# Natural Products Targeting Liver X Receptors or Farnesoid X Receptor

**DOI:** 10.3389/fphar.2021.772435

**Published:** 2022-01-05

**Authors:** Jianglian She, Tanwei Gu, Xiaoyan Pang, Yonghong Liu, Lan Tang, Xuefeng Zhou

**Affiliations:** ^1^ CAS Key Laboratory of Tropical Marine Bio-resources and Ecology, Guangdong Key Laboratory of Marine Materia Medica, South China Sea Institute of Oceanology, Chinese Academy of Sciences, Guangzhou, China; ^2^ College of Earth and Planetary Sciences, University of Chinese Academy of Sciences, Beijing, China; ^3^ NMPA Key Laboratory for Research and Evaluation of Drug Metabolism, Guangdong Provincial Key Laboratory of New Drug Screening, School of Pharmaceutical Sciences, Southern Medical University, Guangzhou, China; ^4^ Southern Marine Science and Engineering Guangdong Laboratory (Guangzhou), Guangzhou, China

**Keywords:** natural products, liver X receptor, farnesoid X receptor, docking, agonists, antagonists

## Abstract

Nuclear receptors (NRs) are a superfamily of transcription factors induced by ligands and also function as integrators of hormonal and nutritional signals. Among NRs, the liver X receptors (LXRs) and farnesoid X receptor (FXR) have been of significance as targets for the treatment of metabolic syndrome-related diseases. In recent years, natural products targeting LXRs and FXR have received remarkable interests as a valuable source of novel ligands encompassing diverse chemical structures and bioactive properties. This review aims to survey natural products, originating from terrestrial plants and microorganisms, marine organisms, and marine-derived microorganisms, which could influence LXRs and FXR. In the recent two decades (2000–2020), 261 natural products were discovered from natural resources such as LXRs/FXR modulators, 109 agonists and 38 antagonists targeting LXRs, and 72 agonists and 55 antagonists targeting FXR. The docking evaluation of desired natural products targeted LXRs/FXR is finally discussed. This comprehensive overview will provide a reference for future study of novel LXRs and FXR agonists and antagonists to target human diseases, and attract an increasing number of professional scholars majoring in pharmacy and biology with more in-depth discussion.

## 1 Introduction

**FIGURE 1:**
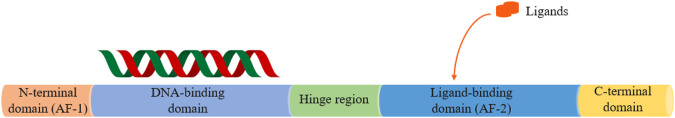
The basic structure of nuclear receptor (NR) domains.

Nuclear receptors (NRs), a superfamily of transcription factors incorporating a group of 48 members in humans and 49 in mice (Gronemeyer et al., 2004; Zhang et al., 2004), are integrators of hormonal and nutritional signals, mediating changes to metabolic pathways within the body (Calkin and Tontonoz, 2012). NRs comprise seven subfamilies, namely, thyroid hormone receptor-like (NR1), retinoid X receptor-like (NR2), estrogen receptor-like (NR3), nerve growth factor IB-like (NR4), steroidogenic factor-like (NR5), germ cell nuclear factor-like (NR6), miscellaneous (NR0), etc. (Zhang et al., 2020c). From N-terminus to C-terminus, NRs share common structure features and are composed of an activation function-1 (AF-1) domain, a DNA-binding domain (DBD), a ligand-binding domain (LBD), a ligand-dependent activation function-2 (AF-2) domain, as well as a hinge region linking the DBD and the LBD (Figure 1) (El-Gendy et al., 2018). NRs activate or repress genes expression required for virtually all aspects of development, reproduction, cell growth, metabolism, immunity, and inflammation by binding with regulatory regions of target genes and acting in concert with coactivators and corepressors. The activity of a large subgroup of NRs depends on small lipophilic, membrane-permeable ligands binding to the LBD, such as fatty acids, oxysterols, and bile acids, functioning as important regulators and ideal drug targets in metabolic syndrome-related diseases for drug discovery and development (Lonard and O’Malley, 2007; McKenna et al., 2009; Hollman et al., 2012).

Among the representative NRs, the liver X receptors (LXRs) were identified as an orphan nuclear receptor in the mid-1990s (Willy et al., 1995; Niesor et al., 2001) and have two isoforms: LXRα (NR1H3, initially named OR-1) and LXRβ (NR1H2, initially named NER and UR) (Shinar et al., 1994; El-Gendy et al., 2018). In humans, LXRα consists of 447 amino acids and is highly expressed in the liver, whereas LXRβ consists of 460 amino acids and is expressed ubiquitously in most tissues and organs (Apfel et al., 1994; Shinar et al., 1994; Willy et al., 1995; Lu et al., 2001; Chinetti-Gbaguidi and Staels, 2009). Both LXRα and LXRβ form obligate heterodimers with 9-cis retinoic acid receptor RXR (retinoid X receptor, NR2B1), and thus, LXRs/RXR might be activated by ligands for either LXRs or RXR, such as endogenous oxysterols for LXRs, which are considered as “permissive” (Chinetti-Gbaguidi et al., El-Gendy et al., 2018). As cholesterol sensors, LXRs have attracted sustaining attention in participating in the regulation of cholesterol, fatty acid, and glucose metabolism, inflammation, and immunity (Janowski et al., 1996; Jakobsson et al., 2012).

The farnesoid X receptor (FXR, NR1H4) identified in 1995 has been known as a member of NRs and a xenobiotic receptor (Niesor et al., 2001; Wang D. et al., 2018). FXR was originally named on the basis of the observation that farnesol and related derivatives possessed the potency of activating FXR (Calkin and Tontonoz, 2012; Zhang J. et al., 2020). In the subsequent years, a series of natural cholesterol metabolites including bile acids were identified as endogenous ligands, meaning that FXR is more appropriately classified as a nuclear bile acid (BA) receptor (Parks et al., 1999; Zhang T. et al., 2020). In accordance with its function as chief sensor of BA, FXR is most abundantly expressed in the liver, intestine, kidneys, and adrenal glands, thus, playing a pivotal role in the regulation of BA metabolism (Lefebvre et al., 2009; Chen et al., 2011). Furthermore, FXR has also been reported to exhibit the regulatory potential of several other physiological processes such as glucose and lipid metabolism (Forman et al., 1995; Parks et al., 1999). Similarly, FXR also forms obligate heterodimers with 9-cis retinoic acid receptor RXR like LXRs. FXR exists as two types of encoded genes, namely, FXRα and FXRβ, although the latter is a pseudogene. Hence, for the purpose of this review, FXRα will be simplified as FXR (Modica et al., 2010).

Natural products are a promising source for bioactive agents and lead compounds for new drug research (Hiebl et al., 2018; Wang D. et al., 2020), which not only include ethnopharmacologically used compounds from plants or herbals but also terrestrial or marine-derived microbial metabolites (Yang et al., 2014). There are plenty of bioactive components in traditional Chinese medicines (TCMs) that might serve as selective ligands for their respective receptors (Qiu, 2007; Cragg and Newman, 2013; Li et al., 2015). Furthermore, the oceans, with their unique environment and huge biodiversity, have the potential as a plentiful source of diverse natural products accompanying pharmacological activities (Carazo et al., 2019). In comparison with synthetic and combinatorial compounds, natural products display a great structural and chemical diversity (Jones et al., 2006; Shen 2015). Beyond that, they cover a large range of biodiversity and profuse functionality because of the capacity of interacting with multiple proteins or other biological targets (Wink, 2003; Jones et al., 2006; Hiebl et al., 2018).

However, there are two main drawbacks to the development of natural products, one of which is that natural products are often in limited supply. Another limitation is the difficulty of separation of trace active compounds and elucidation of unknown compounds (Yang et al., 2014). To date, several technological advances have helped to overcome the above drawbacks, particularly in the developments of high-performance liquid chromatography–electrospray ionization mass spectrometry (HPLC-ESI-MS) and high-resolution nuclear magnetic resonance (NMR) technologies, facilitating the identification and structure elucidation of unknown compounds (Strege, 1999; Koehn and Carter, 2005). Additionally, advances in other subjects, such as metabolic engineering, microbial cultivation, as well as genetic methods, might solve the supply of natural products and contribute to the development of natural product-derived drugs (Ling et al., 2015; Shen, 2015).

This review focuses on natural products targeting LXRs/FXR in the recent two decades (2000–2020). For a brief background of LXRs/FXR, we refer to several recent reviews and give comprehensive overviews regarding this topic, and the docking evaluation of desired natural products targeting LXRs/FXR is finally discussed.

## 2 Physiological functions of liver X receptors and farnesoid X receptor

### 2.1 The role of liver X receptors and farnesoid X receptor in metabolic processes

#### 2.1.1 Cholesterol and lipid metabolism

A stringent control of systemic and cellular cholesterol levels is essential to physiological homeostasis (Bonamassa and Moschetta, 2013; Hong and Tontonoz, 2014). LXRs sense excess cholesterol and trigger various adaptive mechanisms protecting the cells from cholesterol overload. Activation of LXRs results in reverse cholesterol transport (RCT), inhibition of intestinal cholesterol absorption, and suppression of cholesterol synthesis and uptake by the cells (Beltowski, 2008).

ATP-binding cassette transporters A1 (ABCA1) is one of the earliest identified LXR target genes and one of the most highly regulated LXRs targets, required for the ability of LXRs agonists to stimulate cholesterol efflux to apolipoprotein AI (APOAI) (Ishibashi et al., 2013) acceptors. Another transporter ATP-binding cassette transporters G1 (ABCG1) that promotes cholesterol efflux from macrophages is also an LXR target gene (Tarling and Edwards, 2011). RCT is the stimulation of cholesterol removal from the cell, along with transport to the liver, and biliary excretion (Beltowski, 2008). In this process, LXRs upregulate the expression of transporters involved in cholesterol removal from plasma membrane to extracellular acceptors, namely the ABCA1 and ABCG1 (Cavelier et al., 2006), thus, induce cholesterol mobilization from the plasma membrane of nonhepatocyte cell types, cause the formation of high density lipoprotein (HDL) or apolipoproteins, reduce cholesterol expression in the cell membrane, and disrupt lipid rafts formation (Repa et al., 2000; Bradley et al., 2007). LXRs are also involved in the regulation of intracellular cholesterol traffic. LXRs agonists increase the expression of Niemann–Pick C1 (NPC1) and C2 (NPC2) proteins, two carriers mediating transportation from the endosomal compartment to the plasma membrane before efflux, which result in stimulating redistribution of cholesterol from the endosomal compartment to the plasma membrane where it becomes available for efflux to extracellular acceptors (Castrillo et al., 2003; Duval et al., 2006).

In terms of the indispensable interrelation between the intestinal absorption and the regulation of cholesterol levels within the body, LXRs agonists have been verified to stimulate cholesterol recycling from the enterocyte to the intestinal lumen by upregulating ATP-binding cassette transporters G5 (ABCG5) and ATP-binding cassette transporters G8 (ABCG8) (Yu et al., 2003). Additionally, Niemann–Pick C1-like 1 (NPC1L1) protein contained in the apical membranes of enterocytes and attenuated by LXR activation endows a paramount role in intestinal cholesterol absorption (Wang, 2007).

Collectively, cellular and systemic cholesterol homeostasis are maintained by the coordinated actions of sterol-regulatory element-binding proteins (SREBPs) and LXRs. SREBPs are activated in response to low cellular cholesterol levels, whereas LXRs are activated by elevated cholesterol levels (Calkin and Tontonoz, 2012).

FXR activation not only inhibits the uptake and conversion to bile acids of cholesterol, as well as impact the synthesis and excretion of cholesterol, but also promote the expression of hepatic scavenger receptors leading to the enhanced RCT (Neuschwander-Tetri et al., 2015). Growing lines of evidence indicate that FXR activation is an attractive approach for the regulation of lipid homeostasis. FXR-null mice are featured with hypertriglyceridemia, hypercholesterolemia, and growing intestinal cholesterol absorption, in close association with increase in HDL cholesterol and lipoprotein lipase activity, the generation and characterization of which are a breakthrough for uncovering and verifying the importance of FXR action for lipid metabolism (Sinal et al., 2000; Lambert et al., 2003). LXRs regulate the expression of proteins involved in lipid remodeling. The gene cluster of apolipoproteins E, C1, C2, and C4 (APOE, APOC1, APOC2, and APOC4), belonging to LXRs target genes, are implicated in lipid transport and catabolism (Hiebl et al., 2018). Also, FXR has been demonstrated to possess the ability of regulating LXRs-mediated lipogenesis (Han et al., 2016).

#### 2.1.2 Bile acid metabolism

BAs, the end products of cholesterol catabolism in the liver (Fiorucci et al., 2012a), are amphipathic molecules with a steroidal moiety derived from cholesterol (Han et al., 2016). FXR, widely expressing in nonclassical BA target tissues (Wang et al., 2018b), plays a critical role in maintaining BAs homeostasis by controlling their synthesis, transport, and metabolism (Sinal et al., 2000). Take for instance FXR. It protects the liver from the excess BAs by promoting excretion and preventing synthesis and uptake (Jian et al., 2014). Recent researches have demonstrated that BAs-activated FXR decreases BAs *de novo* synthesis in the liver, increases BAs secretion into the small intestine, promotes BA intestinal reabsorption, and inhibits hepatic basolateral BAs reuptake (Gadaleta et al., 2015).

The classic BA synthetic pathway, initiated by the first and rate-limiting enzyme converting cholesterol into BA, cholesterol 7α-hydroxylase (CYP7A1), is considered as the major BA biosynthetic pathway in humans (Russell, 2003). Likewise, FXR activation induces intestinal epithelial expression of fibroblast growth factor 19 (FGF19, also known as FGF15 in rodents), which suppresses BA synthesis by inhibiting CYP7A1 (Inagaki et al., 2005). FXR primarily controls BA synthesis via activating small heterodimer partner (SHP) to inhibit the expression of LXRs, which further suppresses the transcription of CYP7A1 (De Fabiani et al., 2001). As has been stated, CYP7A1 contains a responsive element for the LXRs/RXR heterodimer, proposing that LXRs also might regulate the BA synthetic pathways to a certain degree (Li et al., 2019).

#### 2.1.3 Glucose metabolism

Given the interdependence of lipid and carbohydrate metabolism, it is not surprising that the LXR signaling has affected glucose homeostasis and insulin sensitivity (Goodwin et al., 2008). Glucose transporter type 4 (GLUT4), an insulin-responsive glucose transporter expressed primarily in adipose tissues and skeletal muscle, is usually inhibited in diabetic patients and diabetic mice models. Several studies have illuminated that the expression of GLUT4 in some mice models could be regulated throughout direct interaction with LXR response elements (LXREs) (Mi et al., 2003; Goodwin et al., 2008). Indeed, phosphoenolpyruvate carboxy kinase (PEPCK) and glucose-6-phosphatase (G6P), involved in hepatic gluconeogenesis, are dramatically inhibited in insulin-resistant rats administrated with LXR agonists, resulting in decreasing hepatic glucose, insulin sensitivity, and plasma glucose (Cao et al., 2003).

Another unanticipated area of intense study that recently arose is that FXR are currently under clinical investigation for the pleiotropic role to manage glucose homeostasis. Activation of hepatic FXR decreases plasma glucose levels, downregulates the gluconeogenic pathway, and alters the transcription, either indirectly or directly, of several genes that govern gluconeogenesis and glycolysis. Nevertheless, the underlying mechanisms involved in glycemic response remain controversial.

### 2.2 Liver X receptors and farnesoid X receptor as therapeutic target

**FIGURE 2:**
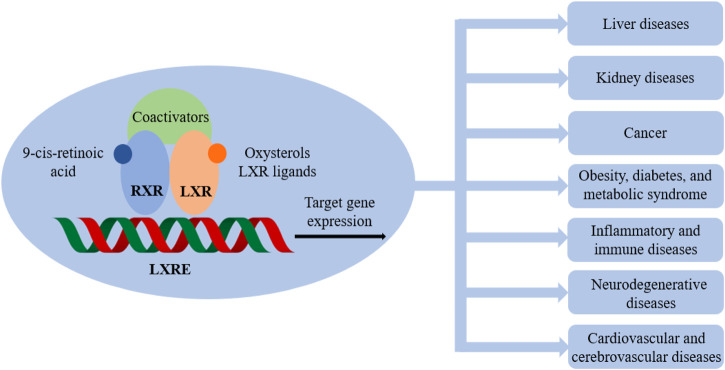
Liver X receptors (LXRs) and related pathological conditions.

**FIGURE 3:**
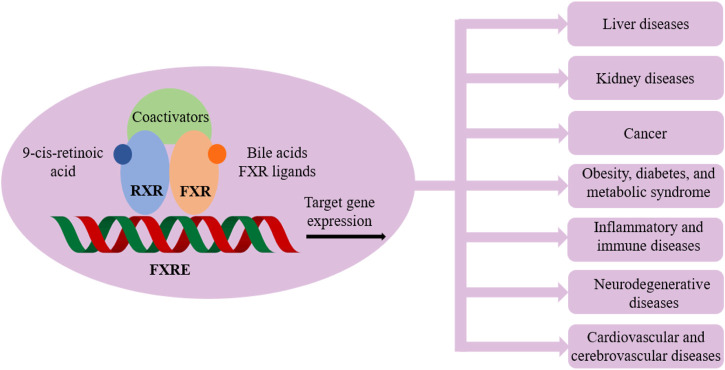
Farnesoid X receptor (FXR) and related pathological conditions.

The fact that LXRs and FXR play a pivotal role in metabolism homeostasis is of relevance for drug research, the functions of which are evidenced in many pathological conditions as illustrated in Figures 2 and 3.

#### 2.2.1 Diabetic mellitus and obesity

It has been covered that human LXRs genes possess potential connections between obesity and diabetes with the advent of genome-wide association studies (Calkin and Tontonoz, 2012). LXR activation promotes triglyceride (TG) accumulation in skeletal muscle cells, probably through the induction of the expression of lipogenic enzymes. Furthermore, LXR agonist treatment improves glucose tolerance in diet-induced diabetic models, involved in glucose metabolism as a potential approach for the treatment of type 2 diabetes (T2D) characterized by high-blood glucose and insulin resistance (Huang, 2014).

The observation that FXR activation results in insulin substrate receptor 1 (IRS-1) phosphorylation on the tyrosine residues in the liver and adipose tissue manifests a prospect application of FXR agonists for the management of patients with insulin resistance to improve insulin sensitivity, indicating that FXR agonists are suitable for T2D (Gadaleta et al., 2015; Han et al., 2016). Intestinal activation of FXR reduces diet-induced weight gain, hepatic glucose production, and steatosis. Meanwhile, it strongly stimulates FGF19 expression in the intestine, reverses high fat-induced diabetes, and enhances the metabolic rate while decreasing adiposity (Cave et al., 2016).

#### 2.2.2 Cancer

LXRs inhibit the proliferation of multiple cancer cell lines from the liver, lung, skin melanoma, prostate, breast, cervical, epidermis, bone, and squamous carcinoma, as well as leukemia T cells (El-Gendy et al., 2018). LXR ligands have shown antiproliferative effects on different cancer cell types, altogether suggesting a ubiquitous and global effect of LXRs on proliferation and apoptosis, not only on cells with a tumor origin (Jakobsson et al., 2012; Boussac et al., 2013).

According to accumulating bibliographic data, FXR may prevent intestinal and hepatocellular tumorigenesis (Gadaleta et al., 2015; Masaoutis and Theocharis, 2018). Emerging evidence supports that FXR seems to possess various antioncogenic and less common prooncogenic attributes. In the case of the pancreas, elevated FXR expression seems to impart better prognosis to adenocarcinoma. In the case of breast cancer, immunohistochemical FXR positivity is also an independent favorable prognostic factor (Masaoutis and Theocharis, 2018).

#### 2.2.3 Inflammation and immunity

Both LXRα and LXRβ are involved in regulating inflammatory responses since they are expressed in a variety of immune cells, and are involved in innate and adaptive immunity and inflammatory responses (Hong and Tontonoz 2008), the activation of which inhibit proinflammatory cytokine production in macrophages derived from wild-type mice rather than from LXRα- and LXRβ-knockout mice (Cave et al., 2016). Moreover, FXR is downregulated in a variety of disease states accompanying inflammation, such as fibrosis, cirrhosis, cardiovascular inflammatory, and cancer (Han et al., 2016). For the therapy of nonalcoholic steatohepatitis (NASH), chronic hepatitis B virus (HBV) infection (Wang et al., 2018c), and inflammatory bowel disease (IBD), FXR has provided a novel target.

Most of the studies conducted to find LXR modulators possessing therapeutic utility have been directed toward LXR agonists. However, the elevation of plasma TG and hepatic steatosis caused by LXRs in the liver, as a major drawback, has impeded its development into commercial drugs. To overcome this undesirable effect, new strategies have been put forward such as developing inverse agonists, LXRβ-selective agonists, and tissue-selective agonists (El-Gendy et al., 2018).

Based on emerging evidence that FXR activation by agonists treatment has a beneficial effect on the treatment of various diseases in animal models, some FXR agonists have been imported into clinical studies for its value as medicinal drugs (Han et al., 2016). At present, some FXR ligands impacted in cholestasis, T2D and metabolic syndrome, nonalcoholic fatty liver disease (NAFLD) or NASH, and primary BA diarrhea are measured in phases I and II clinical trials. Although there is therapeutic benefit of FXR ligands, the risks of various ligands, particularly on the intestines or kidneys, require profound investigation and cautious consideration (Hollman et al., 2012).

## 3 Natural products targeting liver X receptors

In this section, natural products regulating LXRs expression in different models are outlined. We categorized and summarized these natural products in Supplementary Tables S1 and S2.

### 3.1 Natural agonists targeting liver X receptors

Oxysterols, oxygenated derivatives of cholesterol, have been identified as the endogenous agonists of LXRs, including 22*R*-hydroxycholesterol, 24*S*,25-epoxycholesterol, 24*S*-hydroxycholesterol (Janowski et al., 1996; Forman et al., 1997; Lehmann et al., 1997; Huang, 2014), 20*S*-hydroxycholesterol, 25-hydroxycholesterol, and 27-hydroxycholesterol (Huang, 2014; Schroepfer et al., 2000). These oxysterols possess the ability of activating both LXRα and LXRβ. Interestingly, an endogenous ligand, 5α,6α-epoxycholesterol (Janowski et al., 1996, Lehmann et al., 1997; Hiebl et al., 2018) could act as either agonist, inverse agonist, or antagonist depending on the setting. In this review, these endogenous active ingredients are not listed as natural products targeting LXRs.

#### 3.1.1 Terpenes

Geraniol (1), a major constituent of essential oils from aromatic plants, including *Cinnamomum tenuipilum* Kosterm (Lauraceae), *Valeriana officinalis* L. (Caprifoliaceae), and *Panax notoginseng* (Burk.) F.H. Chen, leads to the activation of LXRα and FXR, as assessed in the hyperlipidemic rats, which induced significant and dose-dependent decrease of serum total cholesterol (TC), TG, and low-density lipoprotein cholesterol in hyperlipidemic rats (Ji and Gong, 2007; Galle et al., 2015; Lei et al., 2019). Cineole (2), also named cajeputol, is a monoterpene and a principal constituent of *Mentha longifolia* (L.) L., most eucalyptus oils, teas, rosemary, *Psidium*, and many other essential oils. Treatment with 2 induced remarkable increase in target genes associated with LXRs. It displayed decreased cellular lipid accumulation and reduced cholesterol levels, as evaluated via Oil Red O lipid staining and cholesterol quantification, suggesting practical implications for the development of hypercholesterolemia and atherosclerosis because it not only reduced cholesterol accumulation but also prevented the potential side-effect hepatic steatosis (Jun et al., 2013). Triterpene squalene (3), obtained from dried biomass of *Schizochytrium mangrovei* PQ6, was presented in nutrition, health care, cosmetics, and medicine. A recent study revealed that 3 significantly regulated target genes associated with RCT via stimulating the transactivation of LXRα/β, further confirmed as a potent pharmaceutical agent for the treatment of atherosclerosis and hypercholesterolemia, and the prevention of the potential side effect of hepatic steatosis as well (Hien et al., 2017).

A pterosin sesquiterpenoid named (2*R*,3*S*)-5-hydroxymethylpterosin C (4), isolated from the traditional medicinal plant *Pteris cretica* L., activated LXRα/β in murine 3T3-L1 adipocytes, and exhibited lipid-lowering effect (Luo et al., 2016). Three diterpenes DTP 1 (5), DTP 3 (6), and DTP 5 (7), widespread in plants and insects, are active compounds from traditional folk medicine *Scoparia dulcis* L. for the treatment of inflammation and cancer (Cuadrado et al., 2011; Hu et al., 2018). In RAW cells, gene expression analysis demonstrated that 5–7 could activate either LXRα or LXRβ. Moreover, 5–7 strongly induced the expression of established LXR target genes in macrophages and promoted macrophage cholesterol efflux (Traves et al., 2007).

Two new lanostanoid triterpenes, (3*β*, 24*Z*)-3,27-dihydroxy-lanosta-8, 24-dien-1-one (8) and (3*β*, 23*S*)-3,23-dihydroxy-7,9(11),24-lanostane-triene (9), from the fungus *Rigidoporus microporus* collected in the rubber tree, activated both LXRα and LXRβ. More detailed data revealed that 8 and 9 were dual LXRα/β agonists at 10 µM using a luciferase reporter assay in HEK293T cells (Rincón et al., 2020). TCM plant *Gynostemma pentaphyllum* (Thunb.) Makino has been consumed as tea for numerous beneficial effects, for instance, to treat cough and chronic bronchitis. Gynosaponin TR1 (10), from the aerial parts of *G. pentaphyllum* (Thunb.) Makino, selectively enhanced LXR-mediated transcription activation in HEK293 cells as an LXRα agonist. Notably, 10 possessed higher selectivity for LXRα than LXRβ (Ky et al., 2010; Zhang Y. et al., 2020). Two terpenes, (−)-acanthoic acid (11) isolated from *Rollinia pittieri* Saff. and *R. exsucca* (DC.) A.DC*.*, along with polycarpol (12) isolated from *Unonopsis glaucopetala* R.E.Fr. and *Minquartia guianensis* Aubl., showed potent binding affinity for LXRα with IC_50_ of 0.25 and 0.12 µM, respectively. Amazingly, 11 and 12 seemed to be the better LXRα agonists than natural oxysterols, such as 22*R*-hydroxycholesterol and 24*S*,25-epoxycholesterol (Jayasuriya et al., 2005). *Paeonia lactiflora* Pall. is mainly found in Taiwan and frequently used for the treatment of inflammation, hyperlipidemia, and hyperglycemia, a vital natural monoterpene, which is paeoniflorin (13). In a study using mammalian one-hybrid assay with the specificity for LXRα transactivation, 13 transactivated LXRα at 10 mM and was further confirmed as a LXRα agonist (Lin H.-R., 2013).

*Ganoderma capense* has been documented to possess various pharmacological activities, including defending malignant tumors and hepatitis. Ganocalidin A (14), from the fruiting bodies of *G. capense*, could increase LXRα and reduced lipid accumulation as an LXRα agonist (Liao et al., 2019). Ganoboninone E (15) and ganoboninketals A-C (16–18), dominating compounds of the fruiting bodies of *G. boninense*, exhibited remarkable agonistic activity to the transactivation of LXRβ (Ma et al., 2015; Abdullah et al., 2020). *Tripterygium wilfordii* Hook. f*.* has been used as a traditional medicine for multiple pharmacological activities, including anticancer and anti-inflammatory effects. A major ingredient triptolide (19) from *T. wilfordii* Hook. f*.*, influenced either LXRα or FXR through increasing the LXRα protein expression and suppressing FXR protein expression simultaneously, while the clinical application of 19 is greatly constrained by hepatotoxicity (Jiang et al., 2016; Zou et al., 2020).

**Figure:**
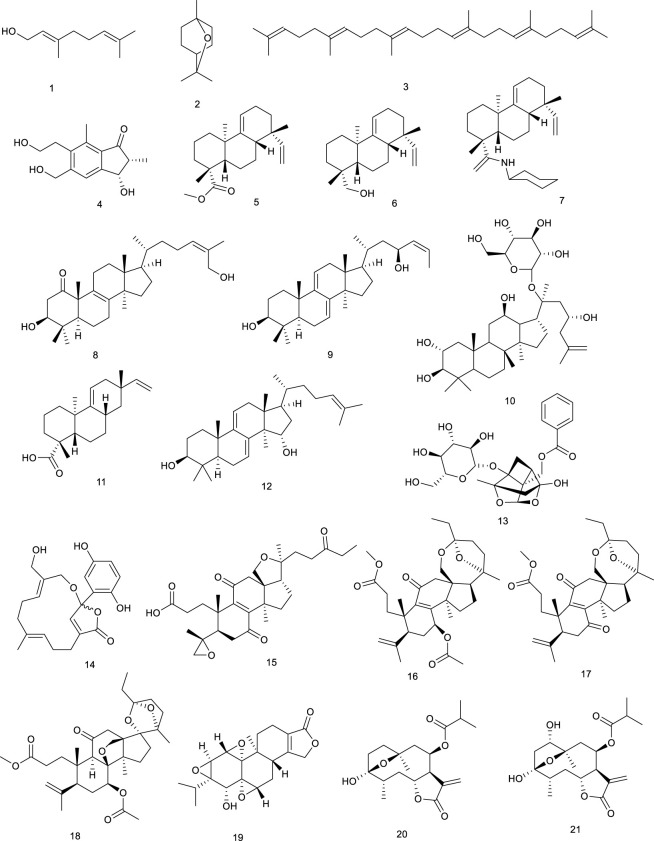


Tirotundin (20) and tagitinin A (21) from *Tithonia diversifolia* (Hemsl.) A. Gray, which are used to treat diabetes and hepatoprotection, also act as LXRα/β and FXR agonists (Lin HR., 2013). Platycodin D (22), a triterpene saponin from *Platycodon grandiflorum* (Jacq.) A.DC., suppressed the generation of proinflammatory cytokine and subsequently inhibited LPS-induced inflammatory response, due to the upregulation of LXRα expression, which is considered as a potential therapeutic drug for mastitis. It exhibited that 22 was capable of attenuating NL-induced inflammatory response in osteoarthritis chondrocyte via LXRα activation (Qu et al., 2016; Wang L. et al., 2017). Saikosaponin A (23), a well-known bioactive component of *Radix bupleuri*, induced cholesterol efflux from lipid rafts through LXRα–ABCA1 signaling pathway, and knockdown of LXRα led to the abrogation of the antiinflammatory effect of 23, suggesting a potency of attenuating oxidative and inflammatory responses (Fu et al., 2015). Viperidone (24), a major ingredient of *Leptocereus quadricostatus* (Bello) Britton & Rose, selectively activated LXRα, and its derivatives also owned the same activity (Jayasuriya et al., 2005; Komati et al., 2017).

**Figure:**
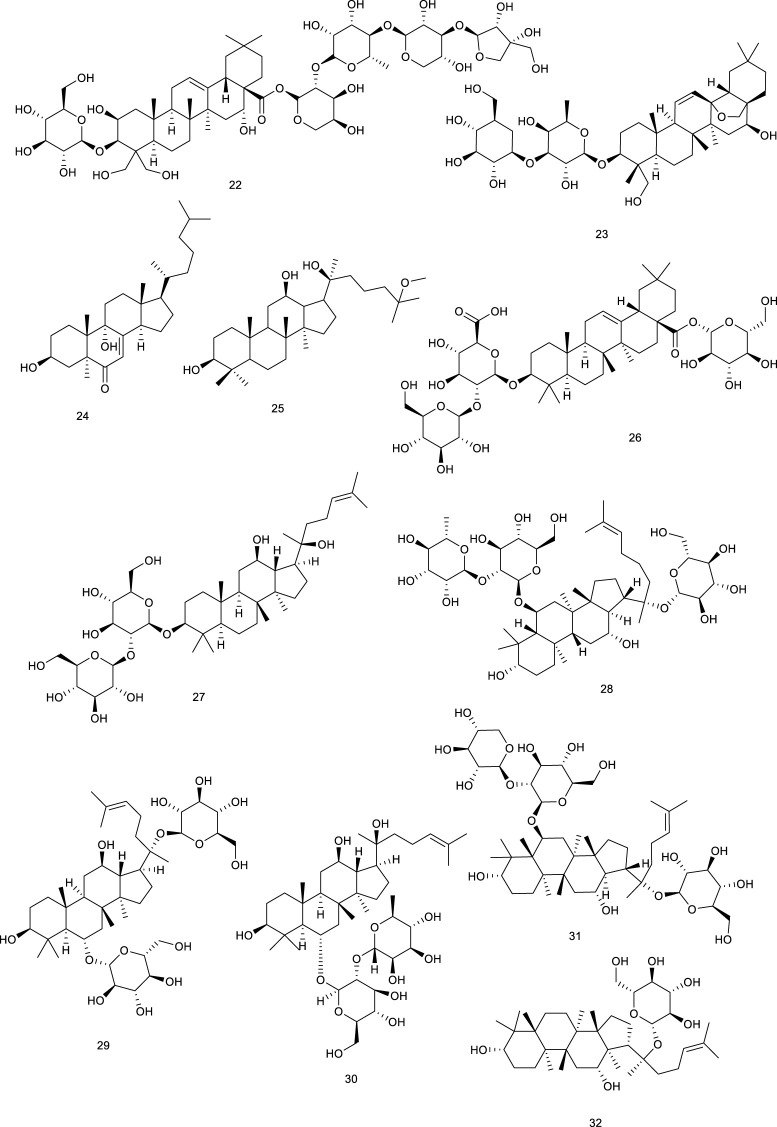


A highly valued TCM *Panax ginseng* C.A.Mey. possesses the efficacy of promoting blood circulation and accelerating metabolism and digestion. A ginsenoside, 25-OCH_3_-PPD (25) from *P. ginseng* C.A.Mey*.*, proved to ameliorate P2X7 receptor-mediated NLRP3 (nucleotide-binding oligomerization domain, leucine-rich repeat, and pyrin domain-containing 3) inflammasome and hepatic fibrosis through inducing LXRα/β pathway in the development of thioacetamide-stimulated hepatic fibrosis mice (Han et al., 2018). Another study indicated that ginsenosides play an important role in cholesterol metabolism since ginsenosides Ro (26), Rg_3_ (27), Re (28), Rg_1_ (29), and Rg_2_ (30) increased LXRα mRNA levels, followed by the upregulation of CYP7A (Kawase et al., 2012). *P. notoginseng* (Burk.) F.H. Chen has been used as a natural remedy in traditional medicine for the treatment of cardiovascular diseases. Terpenes from *P. notoginseng* (Burk.) F.H. Chen, notoginsenoside R1 (31) (Jia et al., 2010; Fan et al., 2012) and ginsenoside CK (32) (Zhang et al., 2020c), could significantly upregulate LXRα functioning as LXRα agonists.

#### 3.1.2 Flavonoids

Cyanidin (33), a very widely distributed flavonoid present in fruits and vegetables, possesses the function of regulating cellular lipid metabolism. It was reported that 33 accelerated LXRα and LXRβ transactivation and induced the recruitment of coactivator peptide with EC_50_ 3.5 and 125.2 μM, respectively. Meanwhile, surface plasmon resonance (SPR) assay demonstrated that 33 directly associated with both LXRα/β with the dissociation equilibrium constant (*K*_*D*_) of 33 to LXRα at 2.16 μM and LXRβ at 73.2 μM, respectively (Jia et al., 2013). Another common flavonoid, hesperetin (34), increased ABCA1 promoter and LXRα enhancer activities, indicating the function of promoting cholesterol efflux (Iio et al., 2012a). Chrysin (35), present in honey, propolis, and plant extracts, significantly enhanced cholesterol efflux and promoted the mRNA level of a set of nuclear receptors including PPAR (peroxisome proliferator-activated receptor) γ, LXRα, ABCA1, and ABCG1 via upregulating the classical PPARγ–LXRα–ABCA1/ABCG1 pathway (Wang et al., 2015). Daidzein (36), widely existing in soybeans, is an indirect modifier of LXRs as it reduced the expression of SREBP-1c through suppressing LXRα, whereas it increased the expression of ABCG8 via activating LXRβ indirectly. Another bioactive flavonoid genistein (37) present in soybeans also was reported to inhibit LXRα activation induced by a synthetic agonist T0901317 while stimulating LXRβ activation. Besides, 37 might prevent NAFLD via the regulation of visceral adipocyte metabolism and adipocytokines (Kim et al., 2010; Gonzalez-Granillo et al., 2012). Naringenin (38), widespread in grapefruits, oranges, and tomatoes, acts as a modulator of LXRα activity. It activated and upregulated LXRα and associated target genes in an AMPK (adenosine 5′-monophosphate-activated protein kinase)-dependent manner, further preventing the deterioration of atherosclerosis and foam cell progression (Wolkow et al., 2010; Saenz et al., 2018). Persimmon tannin is isolated from *Diospyros kaki* L., also known as proanthocyanidins. In a recent study using high-cholesterol diet mice model, persimmon tannin stimulated the expressions of LXRα, PPARα, and PPARγ, and simultaneously, it promoted their associated downstream gene expressions, further accelerating macrophage reverse cholesterol transport (Ge et al., 2017).

Quercetin (39), the pivotal aglycone in *Medicago sativa* L., elevated protein levels of LXRα and PPARγ in THP-1cells. Additionally, it increased cholesterol efflux from THP-1 macrophages and lowered the risk of atherosclerosis. A marked antihepatitis C virus (HCV) activity was exhibited by 39 in replicon-containing cells when combined with interferon α, appearing to be an effective inhibitor of HCV replication (Lee et al., 2013; Pisonero-Vaquero et al., 2014; Ren et al., 2018). Iristectorigenin B (40) derived from *Belamcanda chinensis*, used as a traditional medicine in East Asia for its inflammation property, increased ABCA1 and ABCG1 gene expression function as an agonist for both LXRα and LXRβ significantly. It also stimulated cholesterol efflux in macrophages without inducing hepatic steatosis, providing insights for treating hypercholesterolemia and atherosclerosis (Jun et al., 2012). A flavonoid that is very widespread in food plants like *Phaseolus vulgaris* L. and in medicinal plants like *Cornus alternifolia* L. f. (Cornaceae) is kaempferol-3-*O*-β-D-glucopyranoside (41), as an LXRα/β dual agonist. It exhibited potent LXRα/β agonistic activity with EC_50_ values of 1.8 μM (Dong et al., 2007; He et al., 2012). Interestingly, its aglycone kaempferol (42) selectively activated LXRβ, further suppressing SREBP-1 to regulate metabolic syndrome (Hoang et al., 2015). Formononetin (43), an isoflavone constituent from Brazilian red propolis, enhanced LXR transcription and promoted ABCG1 activity in THP-1 macrophages, indicating agonistic function of both LXRα and LXRβ (Daugsch et al., 2008; Iio et al., 2012b). *Nelumbo nucifera* Gaertn., usually regarded as tea in Japan, has been used in traditional medicine; the leaves of *Nelumbo nucifera* Gaertn. contains quercetin glycosides, especially quercetin-3-*O*-glucuronide (44). It was proven to increase ABCA1 expression via activating LXRα, providing a potential direction on arteriosclerosis prevention (Ohara et al., 2013). *Rhus verniciflua* Stokes is used in TCM for the therapy of gastritis, stomach cancer, and atherosclerosis. One of the effective compounds, butein (45), increased CYP7A1 luciferase activity in AML 12 cells via activating LXRα dependently and upregulating the transcriptional expression level of LXRα, which was borne out using the knockdown of LXRα (Jeong et al., 2015). Scutellarein (46), widely found in *Erigeron breviscapus* (vant.), is a traditional agent for the treatment of inflammation and obesity. Cholesterol output was reduced by 46 by activating the PPARγ–LXRα–ABCA1 pathway, suggesting a potential function in the cholesterol metabolism. Beyond that, treatment with 46 attenuated high-fat diet (HFD)-induced obesity and the associated diseases as it reduced the body weight, inflammatory state, and visceral index as well as improved hyperlipidemia and hepatic dysfunction in C57 mice fed on the HFD-developed obesity (Lin et al., 2019). Puerarin (47), obtained from *Radix Pueraria* or *Pueraria lobate*, attenuated acute lung injury via activating LXRα and suppressed LPS-induced inflammatory response (Li et al., 2017; Wang et al., 2018d). Isoliquiritigenin (48), isolated from *Glycyrrhizae* species, could promote the activation of LXRα and repressed LXRα-dependent hepatic steatosis, further protecting hepatocytes against oxidative injury inflicted caused by fat accumulation (Kim YM. et al., 2010).

**Figure:**
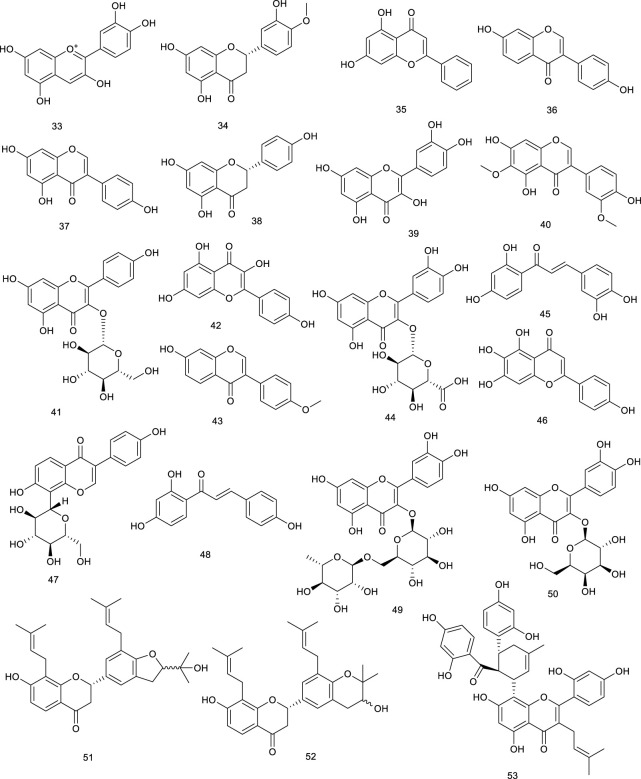


The *n*-butanol fraction isolated from *Zanthoxylum bungeanum* Maxim., widespread in China as an edible aroma and a traditional medicine, mainly including rutin (49) and hyperin (50), was demonstrated to regulate the lipid metabolism in apolipoprotein E knockout mice via promoting the expression of genes involved in RCT, such as CYP27A1, LXRα, and ABCG1 (Wu et al., 2015). SPF1 (51) and SPF2 (52), two flavonoids derived from the root of *Sophora tonkinensis* Gagnep. induced the ABCA1 protein in RAW264.7 cells. A more detailed investigation revealed their neuroprotective effect by activating RXR/LXRs heterodimers as a novel approach to the treatment or prevention of Alzheimer’s disease (Inoue et al., 2014; Wang et al., 2019). A recent study using *APOE* ^*−/−*^ mice model, kuwanon G (53) isolated from the root bark of *Morus alba* L., significantly decreased intracellular lipid accumulation as well as inflammatory cytokines through activating the LXRα–ABCA1/ABCG1 pathway, which upregulated cholesterol efflux-related proteins, ABCA1 and ABCG1, in an LXRα-dependent way (Liu et al., 2018).

#### 3.1.3 Alkaloids

Tetramethylpyrazine (54), an alkaloid diffusely widespread in foods like fermented Japanese food natto and Chinese black vinegar, also present in medicinal plant like *Ligusticum chuanxiong*, could directly regulate the expression of PPAR and LXRα gene by elevating the PPARγ–LXRα–ABCA1 pathway, indicating its potency of improving lipid profiles. In a small cohort of patients with pulmonary arterial hypertension or chronic thromboembolic pulmonary, the therapeutic effects of 54 were accompanied by inhibition of intracellular calcium homeostasis in rat distal pulmonary arterial smooth muscle cells as a novel and inexpensive medication for the treatment of pulmonary hypertension (Chen J. et al., 2017; Chen et al., 2020). Leonurine (55) present in *Herba leonuri* is traditionally used for therapy of gynecological disorders, dysmenorrhea, and menstrual disorders. In a study using Oil Red O staining and liquid scintillation counting assay, 55 mediated cholesterol efflux by promoting ABCA1/G1 induced by PPAR and LXRα, which inhibited lipid accumulation in THP-1 macrophage-derived foam cells via the PPAR and LXRα pathway (Jiang et al., 2017).

A fungus, *Penicillium paxilli* metabolite paxilline (56), was reported as an LXRα/β dual agonist. It activated LXRα and LXRβ with equivalent potency in HEK293 cells, subsequently leading to the expression of ABCA1 and SREBP (Bramlett et al., 2003). Oxepinamides D–G (57–60), four novel oxepin-containing pyrimidines derived from *Aspergillus puniceus* F02Z-1744, were revealed as LXRα selective agonists for their agonistic function of LXRα in chimeric receptor reporter gene assay with the EC_50_ values of 10.6, 12.8, 13.6, and 12.1 μM, respectively (Lu et al., 2011). Oxepinamides H–K (61–64) and four 4-quinazolinone alkaloids, puniceloids A–D (65–68) were obtained from the deep-sea fungus *A. puniceus* SCSIO z021. Compounds 61–68 remarkably activated LXRα with EC_50_ values of 1.7–50 μM, with 67 and 68 exhibiting the most potency (Liang et al., 2019). Scequinadoline D (69), an alkaloid derived from the marine fungus *Scedosporium apiospermum* F41-1, stimulated the mRNA expression of a series target genes, such as LXRα and PPARγ. It was found to facilitate TG accumulation with EC_50_ values of 0.27 ± 0.03 μM as a potent insulin sensitizer targeting adipocytes and a promising potential for the treatment of T2D (Li CJ. et al., 2020).

**Figure:**
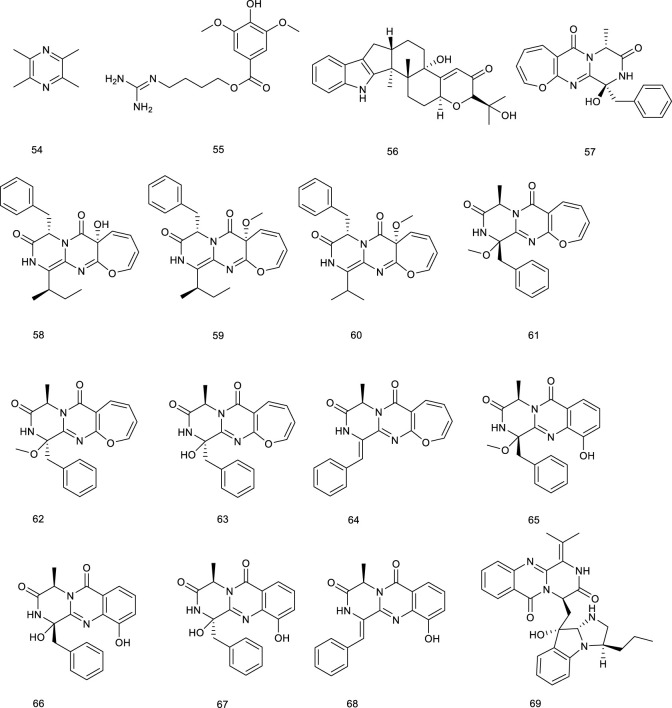


#### 3.1.4 Steroids

Phytosterols, including *β*-sitosterol (70), campesterol (71), sitostanol (72), and YT-32 (73), are equivalent of mammalian cholesterols. Treatment with 70–73 increased the expression of LXRα/β target genes, further supporting subsequent research in intestinal cells (Komati et al., 2017). Two steroids, 24*S*-stigmast-5-ene-3β,24-ols (74) derived from *Ficus pumila* L. and 24*S*-stigmast-5,28-diene-3β,24-ols (75) isolated from genus *Sargassum* were proved to be selectively LXRβ agonists via luciferase assay with GAL-4 chimeric receptors. Meaningfully, their isomers also promoted the expression of LXR target genes, such as ABCA1 and SREBP1c, serving as LXR positive modulators (Castro Navas et al., 2018).

**Figure:**
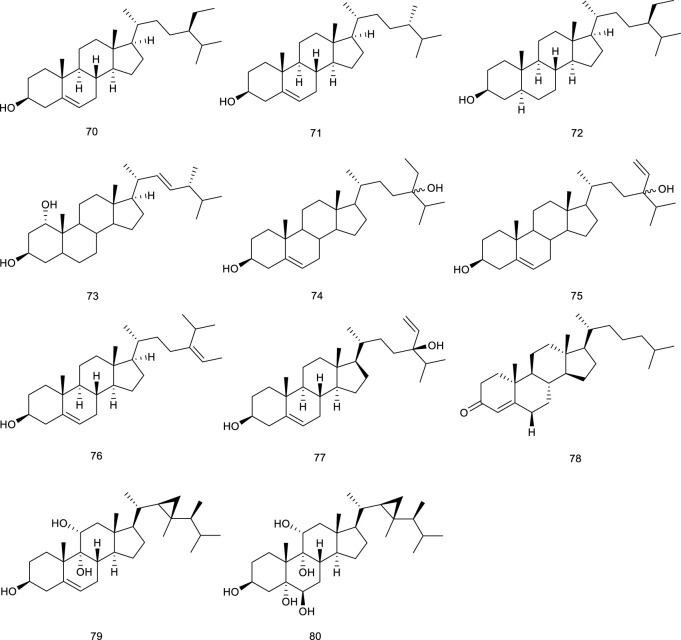


There have been several marine-derived steroids reported as ligands of LXRs. Fucosterol (76), a steroid widespread in marine algae, could promote the transactivation of both LXRα (+155% at 200 μM) and LXRβ (+83% at 200 μM) serving as an LXRα/β dual agonist, further indicating nutritional implications in hypercholesterolemia and atherosclerosis (Hoang et al., 2012a). 24*S*-Saringosterol (77) (maybe same as 75) from an edible seaweed *Sargassum fusiforme*, promoted the transactivation of LXRα/β, and stimulated LXRβ by 14.4-fold, higher than LXRα by 3.8-fold, acting as a selective LXRβ agonist, the discovery of which also further confirmed that phytosterols in *Sargassum fusiforme* contributed to the well-known antiarteriosclerosis. Beyond that, 77 was in the observed effects on cognition and Aβ plaque load as an attractive option for the treatment of neurodegenerative disorders such as AD (Chen P. et al., 2014; Bogie et al., 2019; Hannan et al., 2020). Present in marine fish and plant roots is 4-Cholesten-3-one (78), but it is also found in a red marine alga *Laurencia papillosa*. Compound 78 at 12.5 μM resulted in a remarkable enhancement of the mRNA expression of LXRβ as well as its associated target genes compared with untreated cells in monocytic cell line THP-1. To confirm this effect, LXRs inverse agonist and LXRβ knockdown were employed, which resulted in the neutralization. Further study showed that 78 decreased the viability of two breast cancer cell lines, namely MCF-7 and MDA-MB-231, exerting promising antibreast cancer activity by altering LXRs-dependent lipid metabolism in breast cancer cells without increasing lipogenesis (Elia et al., 2019). Gorgostane-3β,9α,5α,6β,11α-tetrol (79) and gorgost-5-ene-3β,9α,11α-triol (80) isolated from the *Plexaura* species possessed a better affinity with LXRα than LXRβ with EC_50_ values of 0.45 and 0.05 μM (Jayasuriya et al., 2005).

#### 3.1.5 Phenol derivatives

Ethyl 2,4,6-trihydroxybenzoate (81, ETB) was derived from *Celtis biondii* Pamp., which is traditionally used in the therapy of cardiovascular disease. In a reporter gene assay by time-resolved fluorescence resonance energy transfer as well as SPR analysis, ETB (81) suppressed cellular cholesterol accumulation via activating LXRα/β-responsive transcriptional genes as a LXRα/β dual agonist (Hoang et al., 2012b). Paeonol (82), a phenol present in *Paeonia suffruticosa*, is significantly beneficial for various inflammatory diseases, especially atherosclerosis. Treatment with 82 markedly attenuated cholesterol accumulation and suppressed the formation of foam cells in macrophages and *APOE* ^*−/−*^ mice as it activated LXRα to promote LXRα-ABCA1-dependent cholesterol efflux, providing a novel explanation for the antiatherogenic action of 82 (Zhao et al., 2013). Resveratrol (83), widespread in red wine, berries, and peanuts, elevated LXRα together with the mRNA levels of associated target genes such as ABCA1 and ABCG1. A more detailed investigation revealed that ABCA1-mediated cholesterol efflux and intracellular cholesterol could be mediated by 83 via the PPARγ/LXRα pathway. Pathologic analysis of treatment with 83 (30 mg/kg/day) in HBVX protein transgenic mice showed a therapeutic effect on HBVX protein-induced fatty liver and the early stages of liver damage, considering it as a potential preventive agent for HBV-associated hepatocellular carcinoma (Sevov et al., 2006; Voloshyna et al., 2013). Danshensu (84), rosmarinic acid (85), salvianolic acid A (86), and salvianolic acid B (87), bioactive compounds present in *Salvia miltiorrhiza* Bunge used for the therapy of angina pectoris, myocardial infarction, and stroke, functioned as LXRα/FXR dual agonists via the transactivation assays and improved the lipid profiles in hyperlipidemic rats (Ji and Gong, 2008; Li et al., 2015). Methyl gallate (88) and ethyl gallate (89) were isolated from *Talisia nervosa* Radlk, widespread in the tropical moist and wet forest. The evaluation of LXRα transcriptional activity showed that 88 and 89 were 3.16- and 2.62-fold activation of LXRα at a concentration of 100 µg/ml, indicating their potential utility against metabolic syndrome (Vásqueza et al., 2019).

Riccardin C (90), a natural cyclic bibenzyl derivative found in *Blasia pusilla* L. and *Reboulia hemisphaerica*, interacts with LXRα as an agonist. 90 resulted in a 15-fold increase of the transactivation of the reporter gene at 30 μM. Moreover, the coactivator association and receptor-mediated transactivation assay demonstrated 90 as an LXRβ antagonist through its competition with the synthetic agonist TO-1317 (Tamehiro et al., 2005; Asakawa, 2008; Harada et al., 2013). Several natural ligands isolated from plant resin have also shown activities towards LXRs. For an instance, podocarpic acid (91), and its derivatives podocarpic acid imide (92), podocarpic acid anhydride (93), and podocarpic acid anhydride acetate (94), bound to both LXRα and LXRβ at 1–2 nM serving as LXRα/β dual agonists. Especially for 91, it was over eight-to 10-fold better at activation of LXRs compared with a natural LXRs agonist 22(R)-hydroxycholesterol (Singh et al., 2005; Komati et al., 2017).

The described biological activities of sesamol (95) found in sesame oil of *Sesamum indicum* L. mainly include antiinflammatory and antioxidative effects, protecting against hypertension, atherosclerosis, and aging. In a study using pGL3-TK-PPRE-X3-luciferase reporter assay, 95 prominently enhanced LXRα transcriptional activity for 2.6-fold at 100 μM (Periasamy et al., 2013; Majdalawieh and Ro, 2014). Magnolol (96), a natural lignin isolated from *Magnolia officinalis* Rehder & E.H.Wilson, has proved to activate LXRs and PPARγ target genes and inhibit the NF-κB (nuclear factor kappa-B) and mRNA expression of inflammatory cytokines under Aβ incubation *in vitro* studies to attenuate Aβ-induced AD (Xie et al., 2020). Honokiol (97), another lignin from *M. officinalis* Rehder & E.H.Wilson, activated LXRs transcriptional activity and increased its downstream gene ABCA1 expression. Interestingly, it also functions as a dual activator of LXRs/RXR heterodimer (Jung et al., 2010; Xie et al., 2020). Herniarin (98) is a 7-methoxycoumarin derivative found in the aerial part of *Artemisia dracunculus* L., which is traditionally used to alleviate the symptomatic pain of spasmodic colitis. A detailed research indicated that 98 could upregulate LXRα/β to inhibit the development of breast cancer in Sprague–Dawley rats (Talbi et al., 2016; Pattanayak and Bose, 2019).

Emodin (99) is widespread in the TCM such as *Rheum officinale* Baill*.*, *R. palmatum* L., and *Polygonam cuspidatum*, with its biological activities that range from cardioprotective to antioxidant, anticancer, antibacterial, antifibrotic, and anti-inflammatory effects. Emodin (99) promoted cholesterol efflux from differentiated THP1 macrophages through activating the PPARγ/LXRs/ABCA1/ABCG1 signaling pathway. It prevented atherosclerosis in *ApoE* ^*−/−*^ mouse model, and *in vitro* evidence supported this effect commendably, highlighting the therapeutic potential in atherosclerosis (Luo et al., 2021). Six new octulosonic acid derivatives were obtained from the flower heads of *Chamaemelum nobile* L., while 100 and 101 among them exhibited an increase in LXRα activity at 30 μM (Zhao et al., 2014).

**Figure:**
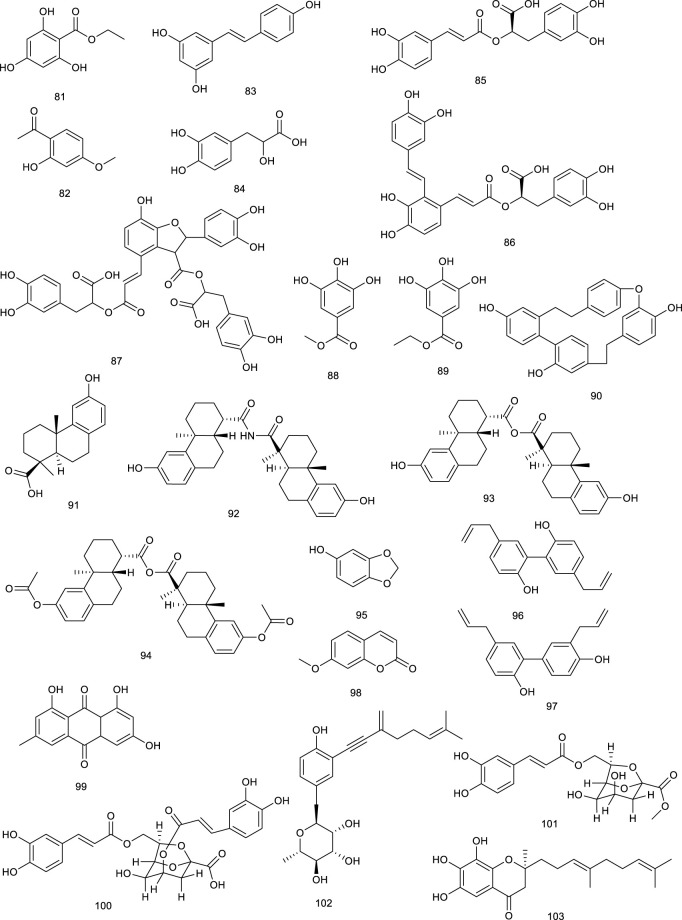


Our previous study revealed eight new ene-yne hydroquinones derived from marine fungus *Pestalotiopsis neglecta* SCSIO41403, one of them, pestalotioquinoside C (102), functioning as a potential LXRα agonist via upregulating the mRNA level of the target gene ABCA1. Using SPR assay, 102 interacted with LXRα dose-dependently with the *K*_*D*_ of 50.0 μM (Wang F. et al., 2020). Further study discovered pestalotiochromones A (103) as a potential LXRs agonist, remarkably upregulated LXRβ and downstream gene ABCA1. Moreover, 103 combined well with LXRα in a dose-dependent manner, the kinetic curves of which resembled those of the potent LXRs agonist GW3965 for binding with LXRα, with *K*_*D*_ of 6.2 μM (Liang et al., 2021).

#### 3.1.6 Others

Betaine (104), a natural trimethyl glycine in common foods, including wheat products, spinach, pretzels, and shrimp, has been used for the therapy of NAFLD via upregulating hepatic expression of LXRα and PPARα, along with attenuating the changes in their associated target genes in fructose-induced rat models. 101 also ameliorated hepatic lipid accumulation, gluconeogenesis, and inflammation through a battery of determinations, further confirming potential mechanisms involved in the treatment of NAFLD (Ge et al., 2016; Chen Q. et al., 2017). Allicin (105) is an essential ingredient of garlic, responsible for its favor, and its pharmacological activities range from anti-inflammatory to antioxidative stress and antihypertensive activities. 105 has also been confirmed to attenuate inflammation via increasing the expression of LXRα in a dose-dependent manner (Zhang et al., 2017). Taurine (106), known as 2-aminoethanesulfonic acid is synthesized in the liver to a small extent, which is also isolated from seafood. Macrophage cells incubated with 106 inhibited cholesterol accumulation and regulated genes expression involved in RCT as an LXRα agonist (Hoang et al., 2012c).

**Figure:**
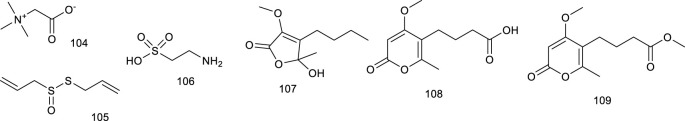


5-Hydroxy-3-methoxy-5-methyl-4-bu-tylfuran-2(5H)-one (107), obtained from marine-derived fungus *Setosphaeria* sp. SCSIO41009, was reported as an LXRα agonist. It decreased ox-low-density lipoprotein-induced lipid accumulation and possessed a greater TG-lowering effect than the positive control T0901317 through targeting LXRα. Taken together, 107 displayed a weak cytotoxicity but had a powerful TC-lowering effect most likely through targeting PPARα, and it exhibited a potential application for the treatment of dyslipidemia (Li et al., 2020b). Setosphapyrone C (108) and D (109) derived from the same fungus, enhanced LXRα/ABC pathways to achieve a lipid-lowering effect, possessing potential application for the treatment of hyperlipidemia (Li et al., 2020c).

In addition, total jiaogulan saponins isolated from *Gynostemma pentaphyllum* were proven to upregulate LXRα and a series of target genes to increase BA and cholesterol excretions (Liu et al., 2016).

### 3.2 Natural antagonists targeting liver X receptors

Polyunsaturated fatty acids (PUFAs) are the dominating source of endogenous LXR antagonists, especially for arachidonic acid identified as a dual antagonist as interacting directly with LXRα and LXRβ, which also could be isolated from traditional medicine *Acanthopanax koreanum* Nakai. (Yoshikawa et al., 2002; Kuang et al., 2012). Prostaglandin F2α, one of the essential metabolites of compound arachidonic acid, has also been verified as a dual antagonist. Notably, prostaglandin F2α could function as an LXRs/RXR heterodimer antagonist (Zhuang et al., 2013). In addition, endogenous antagonist ursodeoxycholic acid, a key secondary BA, has been substantiated to inhibit LXRα-induced lipogenic gene expression as a negative modulator of LXRα signaling (Lee et al., 2014).

#### 3.2.1 Terpenes

Iridoid (110) functions as a predominant compound of *Valeriana jatamansi* Jones. It has been shown that treatment with 110 resulted in the decrease of lipid biochemical indexes in hyperlipidemic rats by decreasing the expressions of SREBP-1c and LXRα (Zhu et al., 2016). Lucidone (111), a natural occurring terpene derivative present in a folk medicine *Lindera erythrocarpa* Makino, has been found to inhibit adipogenesis in 3T3-L1 cells by decreasing adipogenic genes transcription levels, including LXRα, indicating that it is a nutraceutical to guard against obesity and subsequent metabolic disorders (Hsieh and Wang 2013; Wong et al., 2014). Two sesquiterpenoids named paraconiothins C (112) and I (113), isolated from the endophytic fungus *Paraconiothyrium brasiliense* ECN258, were proven to inhibit LXRα at 50 μM (Nakashima et al., 2019).

Ursolic acid (114) is a common pentacyclic triterpenoid in many plants, such as *Cornus officinalis* Siebold & Zucc., and the described pharmacological activities range from anticancer to antioxidant, antiangiogenic, anti-inflammatory, immunoregulatory, hypolipidemic, and hepatoprotective effects. It was demonstrated to be an LXRα antagonist and displays efficacy in treating NAFLD as it significantly decreased TC accumulation and induced steatosis at 20 μM through modulation of LXRα, transcription factor SREBP-1c, and a battery of downstream target genes (Lin et al., 2018). *Potentilla chinensis* Ser. found in oriental countries, has been traditionally used in the therapy of immune disorders and liver diseases. Asiatic acid (115), a pivotal constituent isolated from *P. chinensis* Ser., significantly regulated the key factors associated with lipid metabolism including SREBP-1c and LXRα to restrain the production of hepatocyte lipogenesis. A further study elucidated that 115 effectively alleviated hepatic steatosis and hepatocyte damage, attributed to its ability to alleviate oxidative stress, recruit the antioxidative defense system, inhibit the NF-κB pathway, alleviate hepatocyte apoptosis, and lipid metabolism disorder and, thereby, for the treatment of NAFLD (Wang et al., 2018a). A clerodane diterpenoid borapetoside E (116), derived from *Tinospora crispa* (L.) Hook. f. & Thomson, which is used in traditional medicine for the therapy of diabetes and other diseases, suppressed the mRNA expressions of SREBP and LXRα in the liver, serving a potential therapy for diet-induced T2D. It improved hyperglycemia and oral glucose tolerance, promoted insulin signaling, improved insulin resistance, and improved lipid levels, etc., in high-fat diet-induced obese mice, these beneficial effects *in vivo* further demonstrated the promising therapy (Xu W. et al., 2017). A TCM *Panax ginseng* C. A. Meyer has been regarded as a panacea for centuries in the treatment of various diseases such as metabolic disorders. 20*S*-Protopanaxatriol (117) is present in the root of this plant encompassing antiinflammatory and antioxidative stress bioactive activities. A synthetic agonist T0901317-dependent LXRα target genes and T0901317-induced TG accumulation were inhibited by 117 in primary hepatocytes, further suggesting it as a potential antilipogenic agent to treat NAFLD (Oh et al., 2015a).

**Figure:**
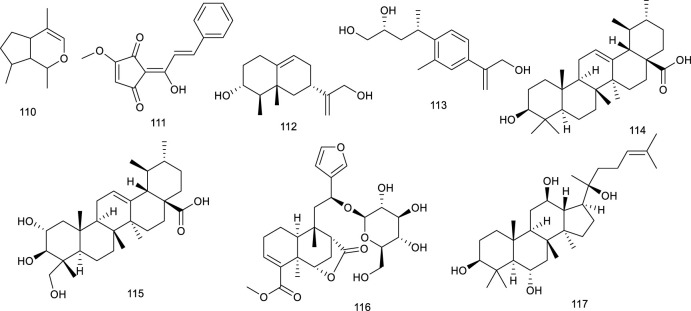


#### 3.2.2 Flavonoids

Luteolin (118), occurring in a broad range of vegetables, fruits, and grains like carrots, peppers, celery, parsley, and spinach, is a common dietary flavonoid exerting numerous biological activities including antioxidant, anticancer, antimicrobial, antiallergic, and antiinflammatory effects. It abrogated agonist-induced LXRα/β transcriptional activity and suppressed the expression of related target genes serving as an LXRα/β antagonist (Francisco et al., 2016). Treatment with 118 inhibited LXR activation in HepG2 cells and eliminated lipid accumulation induced by LXR-SREBP-1c activation, thereby decreasing TG accumulation and primary hepatocytes. Overall, lipid accumulation induced by LXRs-SREBP-1c activation was abolished both *in vivo* and *in vitro* after treatment with 118, indicating the potential as a therapeutic agent for treating NAFLD (Yin et al., 2017). Besides, 118 in combination with cisplatin could potentially be used as a new regimen for the treatment of ovarian cancer (Wang et al., 2018c). It exhibited that 118 could upregulate LXRα and downstream target gene expression to control cholesterol metabolism (Park et al., 2020). Morin (119) present in many plants like mulberry, jackfruit, green tea, also in TCM like *Tartary buckwheat*, could reduce LXRα/β agonism induced by a synthetic agonist GW3965 (Gu et al., 2017a). Licochalcone A (120), present in the licorice root of *Glycyrrhiza* plant, such as *Glycyrrhiza glabra* L. and *G. inflata* Batalin, has been known as “Guolao” in China for thousands of years. The described bioactive properties range from antiparasitic to anticancer, antifungal, antiinflammatory, and osteogenic activities. As an LXRα antagonist, 120 restrained the transcription of lipogenic LXRα and resulted in the diminishment of accumulating TG in primary hepatocytes treated with a synthetic agonist T0901317 (Oh et al., 2015b). Isorhamnetin (121), an active ingredient of *Hippophae rhamnoides* L., downregulated the mRNA levels of LXRα and PPARγ (Lee et al., 2009). It also promoted the protein expression of PPARγ, LXRα, and CYP7A1 in qPCR analysis, indicating it as a potential agent in lipid homeostasis (Xiao et al., 2021). In HFD mice model, administration of alpinetin (122), derived from *Alpinia katsumadai* Hayata, efficiently suppressed LXRα and a series of receptors associated with lipid metabolism. Oral glucose and insulin tolerance tests, immunohistochemical and immunofluorescent analyses manifested that 122 attenuated insulin resistance and alleviated liver injury in HFD-induced mice as an ideal natural product in NAFLD (Zhou et al., 2018).

Sophoricoside (123), an LXRβ antagonist, present in the dried fruit of *Styphnolobium japonicum* (L.) Schott, has been reported to possess an antioxidant property. It could decrease the transcriptional activity of LXRβ concentration dependently (Zhang et al., 2020d). Xanthohumol (124), a critical flavonoid present in the female inflorescences of *Humulus lupulus* L., remarkably attenuated the mRNA expression of a direct target for LXRα transcriptional activation, inducible degrader of the low-density lipoprotein receptor (LDLR) in hepatic cells, which further inferenced a potential role of counteracting LXRs activation (Chen SF. et al., 2017). A flavonolignan silymarin (125), obtained from *Silybum marianum* (L.) Gaertn., has beneficial effects on liver diseases. It has been shown to reduce *de novo* hepatic lipogenesis, recover in insulin sensitivity, and protect against exacerbated myocardial ischemia reperfusion injury. Administration of 125 at 100 and 300 mg/kg depressed the upregulation of LXRβ and related genes expression in the liver of high fructose diet mice (Prakash et al., 2014; Chen et al., 2017b). Citrus fruits contain ample bioactive compounds including flavonoids, carotenoids, and coumarins. Citrange peel extract and citrange flesh and seed extract were proved to decrease LXRα/β and PPARγ level, emerging as a potential candidate for the treatment of obesity and related metabolic disorders (Lu et al., 2013).

**Figure:**
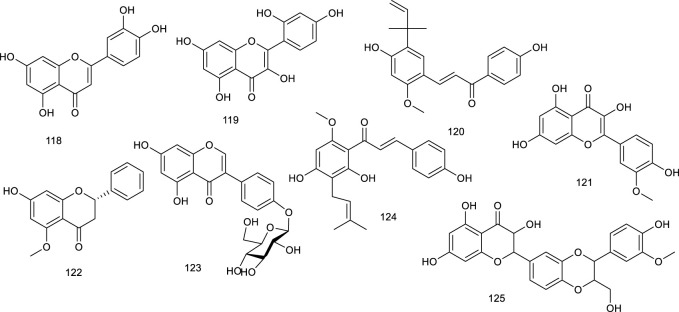


#### 3.2.3 Steroids

Natural steroids antagonists for LXRα principally include ergosterol (126), ergostan-6,8,22-trien-3-ol (127), and ergostan-4,6,8,22-tetraen-3-one (128) from *Tolypocladium niveum*, 129 from *Colletotrichum dematium*, 130 from *Acremonium sordidulum*, and cycloeucalenone (131) from an unidentified fungus. These compounds suppressed LXRα to varying degrees with binding IC_50_ ranging from 0.5 to 6.5 μM (Ondeyka et al., 2005). Diosgenin (132), obtained from *Dioscorea villosa* L., *Rhizoma Dioscorea Nipponicae*, and *Trigonella foenum-graecum* L., has been reported to repress the accumulation of TG and lipogenic genes expression in HepG2 cells at 5 and 10 μmol/L via suppressing LXRα. Compound 132 inhibited an increase in LXRα mRNA in HepG2 cells, which were increased upon high glucose or a synthetic agonist T0901317 treatment. As determined by hematoxylin and eosin and Oil red O staining, along with detection of serum AST and ALT activity, 132 administration obviously ameliorated lipid accumulation in the liver and reduced the elevated serum ALT level in NAFLD rats, suggesting it as a potential agent for preventing NAFLD (Uemura et al., 2011; Cheng et al., 2018).

**Figure:**
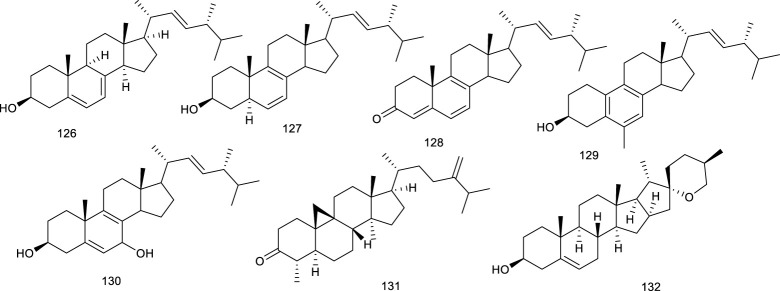


#### 3.2.4 Phenol derivatives

Curcumin (133), an active phenol derivative obtained from *Curcuma longa* L., could suppress the hepatic overexpression of LXRα, PPARγ, and fatty acid synthase. An immunoblot analysis also verified that 133 decreased the protein expression of LXRα and SREBP1c in the liver. Moreover, histological and serum biochemical analyses indicated that 133 apparently attenuated the hepatic lipid accumulation and decreased TG, TC, and nonesterified fatty acid levels in NAFLD mice model on account of the role for the prevention and treatment of NAFLD (Maithilikarpagaselvi et al., 2016; Chen et al., 2017b; Auger et al., 2018).

Meso-dihydroguaiaretic acid (134, MDGA), active dibenzylbutane lignan in *Machilus thunbergia* Siebold & Zucc., appears helpful in inflammation and neurovirulence. In HFD mice model, treatment with 134 reduced LXRα and associated target gene product expression, implying the purpose of attenuating NASH as a selective LXRα antagonist (Sim et al., 2014; Zanellaa et al., 2017). Sesamin (135), a major constituent in *Sesamum indicum* L., exerted antihyperlipidemic effects via decreasing LXRα expression and its downstream target genes, which were induced upon T0901317 treatment (Tai et al., 2019; Majdalawieh et al., 2020). Sauchinone (136), obtained from *Saururus chinensis* (Lour.) Baill., significantly decreased luciferase activity of LXRα activated by T0901317 and further inhibited LXRα-mediated SREBP-1c induction in HepG2 cells, preventing fat accumulation of hepatocytes (Kim YW. et al., 2010). A potential cholesterol-lowering agent alkaloid present in *Piper longum* L. is piperine (137), for which various biological activities, including anti-inflammatory, antiasthmatic and antitumor effects have been described. It inhibited the expression of ABCG5/8 and LXRα in the liver as a potential agent in preventing cholesterol gallstone formation and reduced biliary cholesterol secretion induced by lithogenic diet in C57BL/6 mice (Song et al., 2015).

Cyclic bibenzyl derivative riccardin F (138), present in liverworts, such as *Blasia pusilla* L*.*, suppressed LXRE-dependent luciferase transcription in HepG2 cells functioning as an LXRα antagonist as well, and its homologous compound 90 has been described above as an LXRβ antagonist (Tamehiro et al., 2005; Asakawa, 2008). An LXRα antagonist biacetophenone cynandione A (139) found in the dried roots of *Cynanchum wilfordi* is both an endemic tonic and a traditional herbal medicine to promote liver and renal function. In HepG2 cells, treatment with 139 inhibited the mRNA levels of SREBP-1c as well as the expression of each enzyme upregulation promoted by two extrinsic LXRα agonists, GW3954 and T0901317 (Kim et al., 2020). Guttiferone I (140) is a naturally occurring polyisoprenylated benzophenone found in *Garcinia humilis* (Vahl) C.D.Adams. It has been found to bind with LXRα as an LXRα antagonist earlier on (Herath et al., 2005).

Rhein (141) is a key phytochemical of *Rheum palmatum* L., which is used as a TCM for thousands of years to treat obesity, constipation, ulcers, and gastrointestinal hemorrhage. It resulted into LXRs antagonism, dose-dependently interacted with LXRα and LXRβ with *K*_*D*_ values of 46.7 and 31.97 μM in SPR assay, respectively. Moreover, 141 suppressed the transcriptional activity of SREBP-1c and further dose-dependently reduced the transaction of LXRs induced by GW3965. A further detailed study showed that 141 suppressed the expression levels of LXRs target genes. According to a series pharmacological experiment in an HFD-induced obese mouse model such as intraperitoneal glucose tolerance test and serum, fecal, and liver lipid content analysis, 141 showed several beneficial treatment effects on NAFLD in mice model, including decreased body and fat weight, lowered serum and hepatic lipid levels, improved insulin resistance, and reversed hepatic steatosis (Sheng et al., 2011a; Sheng et al., 2011b; Xue et al., 2020).

**Figure:**
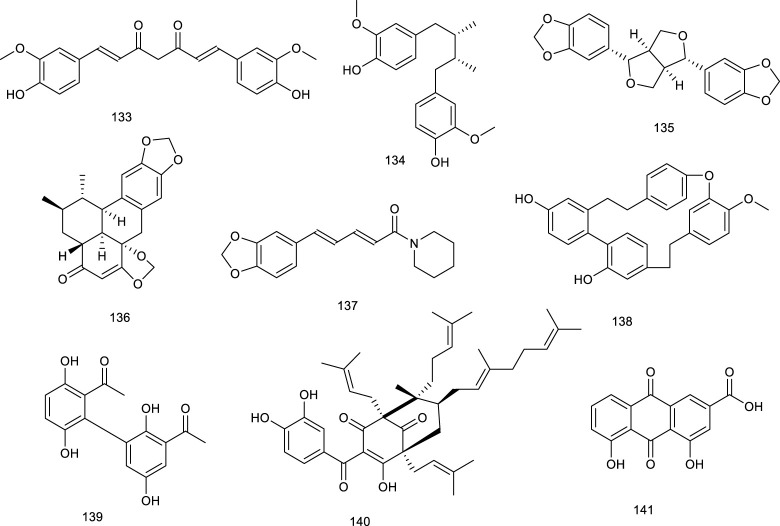


#### 3.2.5 Others

Our recent study revealed that piericidin A (142, PA) and its glycoside glucopiericidin A (143, GPA), isolated from a marine-derived *Streptomyces* strain, could decrease the protein level of LXRα rather than mRNA expression. SPR assay was further employed to explore, along with several other piericidin analogs. As expected, PA presented a similar LXRα protein affinity (K_D_ 3.24 × 10^−5^ M) compared with GW3965 (K_*D*_ 6.97 × 10^−5^ M). Another piericidin aglycone, 10-ketone PA (144), could also serve as an LXRα ligand with a *K*_*D*_ value of 7.22 × 10^−5^ (M). However, the affinity for GPA–LXRα binding was much weaker in nonkinetic simulation binding mode, with the K_D_ value of 3.492 (M). Other piericidin glycosides, such as 13-OH GPA and 6″-Gal GPA, also exhibited no binding activity. The molecular docking analysis also showed the significant differences in the binding effects of glycosides and glycosides with LXRα protein. Intriguingly, PA (142) and GPA (143) have been considered as antirenal cell carcinoma (RCC) drug candidates, whereas on account of LXRα, PA/GPA caused specific hepatotoxicity in RCC xenograft mice-induced ALT to 2.6- and 2.3-fold, as well as the AST to 1.6- and 2.2-fold higher than that of the control group, respectively. Further study demonstrated that PA/GPA aggravated hepatotoxicity in high cholesterol diet-fed LXRα-activated mice while exhibiting no toxicity in chow diet-fed mice, indicating that the accumulation as well as the delay metabolism process of cholesterol resulted in hepatotoxicity and cholestasis. Pharmacokinetic study discovered that GPA existed as a prodrug in the liver and exerted toxic effect due to transforming into PA, different from PA directly combined with LXRα as an inhibitor (Zhou et al., 2019; Liang et al., 2020).

**Figure:**
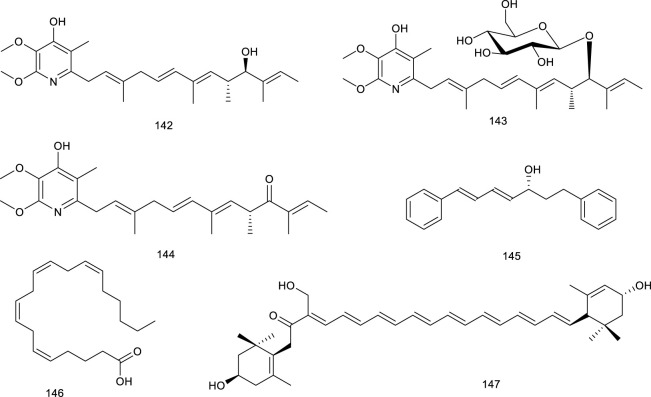


3*R*-1,7-Diphenyl-4*E*,6*E*-4,6-heptadien-3-ol (145), namely DPHD, is present in an indigenous medicinal herb *Curcuma comosa* Roxb. 145 upregulated mRNA and protein expressions of LXRα, SREBP1c, and their downstream targets in bilateral ovariectomy rats, exerting a lipid-lowering effect (Sutjarit et al., 2017). An endogenous LXR antagonist, arachidonic acid (146) (Ou et al., 2001) was also reported as a natural product from traditional medicine *Acanthopanax koreanum* Nakai. (Yoshikawa et al., 2002; Kuang et al., 2012). Another LXRα antagonist siphonaxanthin (147), a carotenoid present in green algae, such as *Codium fragile*, *Umbraulva japonica*, and *Caulerpa lentillifera*, repressed the excess accumulation of triacylglycerol induced by LXRα agonists, this effect was due to the downregulation of SREBP-1C and a series of associated target genes (Zheng J. et al., 2018).

It exhibited that schisandra polysaccharides, isolated from *Schisandra chinensis* (Turcz.) Baill (Wang et al., 2016), phenol, flavonoid, and sugar collected in the fruit of *Abelmoschus esculentus* (Zhang et al., 2020b), and total glycosides, isolated from *Ligustrum lucidum* L. Moench (Zhao and Liu, 2020), have the potency to downregulate LXRα and a set of associated genes, indicating both preventive and therapeutic role in regard to metabolic disorders.

## 4 Natural products targeting farnesoid X receptor

In this section, we gather natural products regulating FXR expression in different models and categories. These are illustrated in Supplementary Tables S3 and S4.

### 4.1 Natural agonists targeting farnesoid X receptor

BAs are endogenous agonists targeting FXR/PXR, with chenodeoxycholic acid (CDCA) being most effective. The activation of BAs targeting FXR follows a rank order of potency as CDCA > deoxycholic acid (DCA) > lithocholic acid (LCA) > cholic acid (Parks et al., 1999; Carottiet al., 2014).

#### 4.1.1 Terpenes and steroids

Forman et al. identified and described an orphan nuclear receptor, farnesoid X-activated receptor, which is activated by a naturally occurring isoprenoid farnesol (148) and related derivatives. Farnesol (148), present in many essential oils, activated FXR-RXR at the concentrations ranging between 5 and 50 μM as an FXR agonist, further inhibiting hepatic TG synthesis via regulating the mRNA expressions of related molecules involved in lipid metabolism (Forman et al., 1995). Further study indicated that 148 activated both PPARα and FXR at similar concentrations (30 or 50 μM) in CV-1 cells (Goto et al., 2011).

Lepidozenolide (149), a sesquiterpenoid isolated from the liverwort *Lepidozia fauriana*, could significantly activate FXR in a dose-dependent manner (Lin, 2015). Cafestol (150), a well-known ingredient of coffee, has been identified as a dual FXR/PXR agonist, as assessed by a double FXR/PXR KO mice model. It increased the expression of FXR-target genes in pathways of BA biosynthesis and intestinal FGF15 via the contribution of FXR/PXR activation (Ricketts et al., 2007). A naturally occurring terpenoid found in *Ferula ovina* Boiss. is tschimgine (151), capable of activating FXR and bind ideally with the LBD of FXR in molecular docking. Interestingly, 151 inhibited the induction of inflammation genes in an FXR-dependent manner (Zheng W. et al., 2017). Dihydroartemisinin (152), the derivative of artemisinin, is present in *Artemisia carvifolia* Buch.-Ham. ex Roxb. and emerged as a potential candidate for the treatment of liver cancer and fibrogenesis. Compound 152 activated FXR to inhibit contractility of cultured hepatic stellate cells as a potential natural product to attenuate portal hypertension in fibrotic rodents (Xu J. et al., 2016; Xu Y. et al., 2017; Ma et al., 2020). *Scrophularia dentatais*, a folk herbal medicine found in Tibet, is emerging as a potential candidate for the treatment of smallpox, measles, high-heat plague, and poisoning. Zhang et al. isolated four compounds from the whole plant of *Scrophularia dentatais* Royle ex Benth., all of them being C_19_-diterpenoids. One is scrodentoids F (153) that dose-dependently activated FXR in dual-luciferase reporter assay system in 293T cells (Zhang et al., 2016). Picroside II (154), the major terpene in *Picrorhiza scrophulariiflora* Pennell, activated FXR via transient transfection in a dual-luciferase reporter assay. It decreased CYP7A1 and increased bile salt export pump and UDP-glucuronosyltransferase 1a1 mRNA expression, the latter two of which were inhibited upon knockdown of FXR. Meanwhile, 154 dose-dependently reversed α-naphthylisothiocyanate (ANIT)-induced alteration in serum markers and thereby exerted protective effect on ANIT-induced cholestasis, possibly through FXR activation (Li et al., 2020d; Li et al., 2020e). Geniposide (155), a pivotal constituent in *Gardenia jasminoides* Ellis, activated FXR, PXR, and SHP. Blood biochemical determination discovered that 155 at 25, 50, or 100 mg/kg increased bile flow rate and reversed the ANIT-induced increases in serum markers for hepatotoxicity and cholestasis in a dose-dependent manner (Wang Y. et al., 2017).

**Figure:**
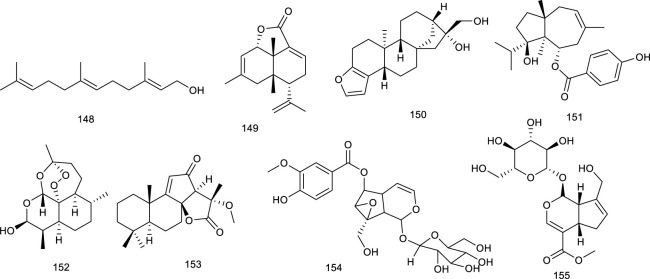


Genet et al. screened betulinic acid (156) from the *Betula* species, oleanolic acid (157) from *Olea europaea* L., and ursolic acid (114) from *Cornus officinalis* Siebold & Zucc., and tested them regarding their ability to activate FXR. In the results, 156, 157, and 114 activated FXR along with potential impact in diabetes (Genet et al., 2010), and 157 also generated frail FXR agonism, along with moderate PXR agonism, which protected against LCA-induced hepatotoxicity and cholestasis (Chen Z. et al., 2014). Hedragonic acid (158), from the stem and root of *Celastrus orbicalatus* Thunb., remarkably activated FXR and possessed therapeutic effects on liver injury and inflammation in acetyl-para-aminophenol-induced mice model as an FXR agonist (Lu et al., 2018). Ergosterol peroxide (159) and four triterpenoids, ganoderiol F (160), ganodermanontriol (161), lucidumol A (162), and ganoderic acid TR (163), are bioactive lanostane metabolites of *Ganoderma lucidum* Karst., which has been used in Asian medicines since 2,000 years against a variety of diseases including tumor, inflammation, obesity, and diabetes. 159–163 were verified as FXR agonists as inducing FXR with the low micromolar range in HEK293 cells (Grienke et al., 2011). Ginsenoside Rg1 (29) from *Panax ginseng* C.A.Mey. is both FXR and LXRs agonist, the function of 29 on LXRs has been described above. Herein, 29 also was reported to activate FXR to promote bile secretion and normalize enzyme activity in the serum in intrahepatic cholestasis mice model (Xiao et al., 2020).

*Astragalus membranaceus* (Fisch.) Bunge, widespread in Europe and Asia, is beneficial to the treatment of diabetes, hyperlipidemia, atherosclerosis, and cancers, with its key constituents like astragaloside IV and cycloastragenol (164). Compound 164 stimulated FXR transcription activities and regulated the expression of FXR target gene in HepG2 cells as a potential candidate for NAFLD. Meanwhile, it improved metabolic profiles, ameliorated hepatic steatosis, altered BA composition, and activated FXR signaling and feedback loops in diet-induced obesity mice, further confirming the promise in ameliorating NAFLD. Besides, 164 also alleviated hepatic steatosis in methionine and choline-deficient L-amino acid diet-induced NASH mice (Gu et al., 2017b). Alisol M 23-acetate (165) and alisol A 23-acetate (166) present in *Alisma orientalis* (Sam.) Juzep. have been described as FXR agonists by transactivating FXR to modulate promoter action (Lin 2012). Another FXR agonist present in *A. orientale* (Sam.) Juzep and *Rhizoma alismatis* is alisol B 23-acetate (167), protecting against NASH and CCl_4_-induced hepatotoxicity via FXR activation (Meng et al., 2015; Meng et al., 2017; Huo et al., 2018). In addition, 167, aisol F (168), aisol A (169), and 25-anhydro alisol A (170) found in *A. orientale* (Sam.) Juzep. activated FXR in a dose-dependent manner (Huo et al., 2018).

**Figure:**
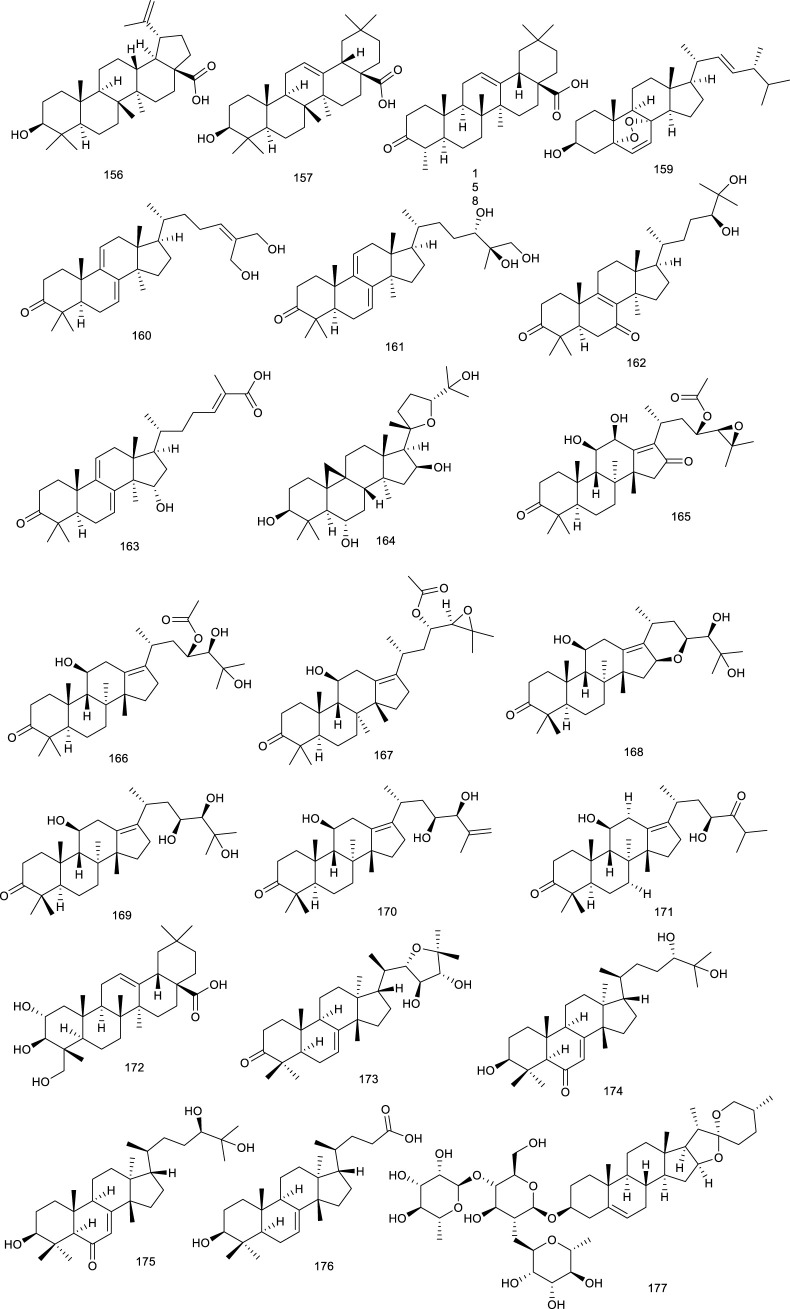


Recently, another protostane-type triterpenoid 23*S*-11β,23-dihydroxy-8α, 9β,14β-dammar-13(17)-ene-3,24-dione (171) from *A. orientale* (Sam.) Juzep. was confirmed with the activities of activating FXR with an EC_50_ value of 90 nM (Luan et al., 2019). An oleanane triterpenoid arjunolic acid (172) is present in the heartwood of *Terminalia arjuna* W. & Arn*.*, a common traditional medicine in India. Compound 172 was reported to upregulate the FXR and PPARα expression, as well as downregulated PPARγ expression in HFD-fed rats as a lead molecule for the therapy of NAFLD (Toppo et al., 2018).

Four tirucallanes xylocarpols E (173), agallochols A (174), B (175), and D (176) isolated from mangrove plants, activated FXR at the concentration of 10.0 µM in a luciferase reporter assay in HepG2 cells (Jiang et al., 2018). *Dioscoreae rhizome*, also known as “Shaoyao,” acts as either a food or a traditional medicine because of its protectivity of the kidneys and liver. Dioscin (177), a steroid saponin from *D. rhizome*, attenuated oxidative stress and inhibited inflammation to improve thioacetamide-induced acute liver injury in Sprague–Dawley rats as upregulating the expression levels of FXR and AMPKα via FXR/AMPK signal pathway, providing a new insight in the treatment of acute liver injury (Zheng L. et al., 2018).

#### 4.1.2 Flavonoids

Calycosin (178), present in the dry roots of *Radix Astragali*, has been demonstrated to improve TG metabolism, mitigate obesity, and hyperlipidemia. It played an emerging role in protecting against liver injury induced by CCl_4_ on account of activating FXR and its target genes as an FXR agonist (Chen et al., 2005b). A more detailed study showed that 178 attenuated TG accumulation and hepatic fibrosis to protect against NASH through FXR activation (Duan et al., 2017). *Cryptocarya chinensis* (Hance) Hemsl. is an endemic famous for its antioxidant, immunological, and antitumor effects in southern China, Japan, and Taiwan. Four naturally occurring tetrahydroflavanone cryptochinones A–D (179–182), obtained from this plant, transactivated FXR dose dependently in a transient transfection reporter assay, semblable to CDCA (Lin et al., 2014). Xanthohumol (124) is not only a counteractor of LXRs activation, but also an FXR modulator. It activated FXR dose-dependently in the transient transfection assay. Furthermore, 124-fed mice possessed lowered levels of SREBP-1c, gluconeogenetic genes, as well as targets involved in fatty acid synthesis in KKA-(y) mice, indicating a promising role in attenuating diabetes (Nozawa, 2005).

A flavanone glycoside abundantly found in lemons and oranges is hesperidin (183), treatment with which prevented cholestatic liver injury and reduced BA toxicity in HepaRG cells via activating FXR. Compound 183 dose-dependently protected against 75 mg/kg dose of ANIT-induced cholestasis and liver injury as reversing increases in the liver index, biliary index, serum AST, ALT, alkaline phosphatase, and total bilirubin, functioning as an effective agent for the prevention and therapy of cholestatic liver disease (Zhang et al., 2020a). *Desmodium styracifolium* has extensive biological activities, ranging from cholesterol level-lowering to cancer prevention and inflammatory inhibition. Schaftoside (184), a flavanone glycoside found in *D. styracifolium*, ameliorated oxidative injury and inflammation as an FXR agonist. Particularly, 184 attenuated acetaminophen-induced hepatotoxicity via restraining NF-κB signaling and fine-tuning the generation of pro- and antiinflammatory eicosanoids, providing a promising agent to alleviate liver injury induced by acetaminophen overdose (Liu et al., 2020a). In another study, HFD-induced lipid accumulation in the liver was decreased after treatment with 184, as indicated by reduced AST and cholesterol in serum, along with intracellular TG levels in the serum and liver tissue, subsequently resulting in attenuation of liver histopathological injury. As assessed by RT-PCR and Western blotting, 184 ameliorated HFD-induced NAFLD probably via FXR–SREBP1 signaling (Liu et al., 2020b). A crucial tea catechin, epigallocatechin-3-gallate (185, EGCG), is well known as a potential tonic for cardiovascular diseases and a variety of cancers. EGCG activated FXR with the EC_50_ value of 2.99 μM, nevertheless, which seemed to be specific because of the nonactivation of other nuclear receptors. It was regarded as a selective SBARM (a selective bile acid receptor modulator) (Li et al., 2012). EGCG possessed the strongest anti-HBV activity toward HBsAg and HBeAg among the crude green tea catechin tests. Meanwhile, the interaction between 185 and FXR has been confirmed to decrease the transcriptional activation of HBV, serving as an FXR antagonist and anti-HBV agent (Xu W. et al., 2016).

*Penthorum chinense* Pursh, also known as “GanHuang-Cao,” has been used as food and Chinese tea for thousands of years, with various biological activities, including antioxidation and antihepatitis virus effects. Notably, Gansu granule made up of the extracts of *P. chinense* has been used in clinics for the treatment of acute hepatitis. Besides, pinocembrin-7-*O*-[2″-*O*-galloyl-4″,6″-hexahydroxydiphenoyl]-β-D-glucose (186) and 2′,6-dihydroxydihydrochalcone-4′-*O*-[2″-*O*-galloyl-4″, 6″-hexahydroxydiphenoyl]-β-D-glucopyranoside (187) present in *P. chinense* activated hepatic FXR to BA homeostasis and regulated lipid metabolism (Zhao et al., 2021).

Total flavonoids of *Astmgali Radix* (TFA) are the essential ingredients of *A. Radix*, which contributes to the pharmacological efficacy including antiinjury, antimutation, and antitumor effects of this herb. TFA reversed the decrease in mRNA and protein levels of FXR induced by dimethylnitrosamine in rats and mitigated liver function in rats with liver cirrhosis (Cheng et al., 2017). Procyanidins is abundant in grapes, apples, red grape juice, and red wine, whose folkloric applications embody in preventing and ameliorating atherosclerosis and other cardiovascular disease. A SBARM grape seed procyanidin extract promoted FXR transcriptional activity induced by BA and FXR dependently resulted in a reduction in TG *in vivo* (Del Bas et al., 2009).

**Figure:**
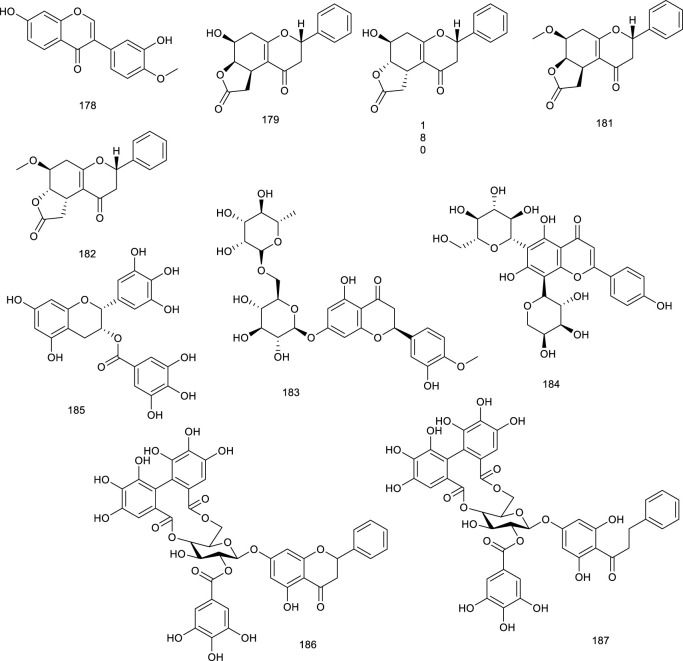


#### 4.1.3 Phenol derivatives

A farnesyl phenolic compound grifolin (188) and ginkgolic acid 15:1 (189) from mushroom or *Ginkgo biloba* L., and geranyl caffeate (190) from Himalayan poplar, activated FXR ranging from 20 to 30 μM in monkey kidney cell line (Suzuki et al., 2006). Marchantins A (191) and E (192), macrocyclic bis-bibenzyls present in the liverworts of *Marchantia paleacea* Bertol., activated FXR at 10 μM (Suzuki et al., 2008; Asakawa and Ludwiczuk, 2018). A fungal metabolite altenusin (193), isolated from *Alternaria* sp. present in the leaves of *polygonumse negalense*, functioned as an FXR agonist with an EC_50_ value of 3.4 μM. Additionally, weight and fat mass, along with blood and glucose serum insulin level were reduced after treatment with 193 in HFD-fed mice. It reversed hepatic lipid droplet accumulation and macrovesicular steatosis, which were abolished in FXR-knockout mice (Zheng Z. et al., 2017).

Papaverine (194) is a bioactive constituent present in *Papaver somniferum* L. and contributes to the pharmacological efficacy of this herb, appearing to be an FXR agonist in a full length FXR transactivation assay (Steri et al., 2012). Another famous natural product berberine (195), beneficial for diarrhea, isolated from the roots of *Coptis chinensi*, regulated BA metabolism and, thus, exerted a lipid-lowering effect, functioning as an intestine-restricted FXR agonist. It prevented diet-induced obesity and TG accumulation in the liver with a significant decrease in serum and hepatic TG by 19% and 47%, respectively. A further mechanism study discovered that the antiobesity and lipid-lowering effects of 195 are primarily due to the activation of intestinal FXR and thereby result into a reduction in the CD36 expression (Sun et al., 2017; Tian et al., 2019).

Three naturally occurring phenylpropanoids, nelumol A (196) and nelumal A (197), from *Ligularia nelumbifolia* Hand. Mazz. (Bruyere et al., 2011), and auraptene (198), from *Citrus aurantium*, exhibited significant agonistic activity of FXR as FXR agonists (Epifano et al., 2012). The activation of FXR via 198 also resulted in hepatoprotective effect against cholestatic liver injury and increasing effect of BA efflux from the liver into the intestine (Gao et al., 2017). Moreover, it showed that 198 significantly attenuated apparent collagen deposition between liver lobules in thioacetamide-induced liver fibrosis mice. Treatment with 198 at 30 mg kg^−1^ significantly lowered the grade of fibrosis compared with the thioacetamide group. Further gene expression involved in hepatic stellate cell activation and fibrosis also confirmed its efficacious potency. Overall, 198 seemed to be beneficial to hepatic fibrosis as reducing toxic BAs and inhibiting hepatic stellate cell activation and inflammation due to FXR activation (Gao et al., 2018). Another phenylpropanoid present in the rhizomes of the *Podophyllum* species, podophyllotoxin (199), could transactivate FXR at a concentration of 10 μM in HeLa cells (Hiebl et al., 2018).

“Se-Ji-Mei-Duo,” recognized as having auspicious and beautiful flowers, is a traditional medicine, *Herpetospermum pedunculosum* (Ser.) C.B. Clarke, for which various biological properties including anti-HBV, anti-inflammatory, and antioxidant effects have been described. Five lignans, herpetotriol (200), spathulatd (201), lariciresinol (202), dehydrodiconiferyl alcohol (203), and herpetrione (204), have emerged with significant FXR agonistic activity *in vitro* and, thus, were identified as the main bioactive constituents in the seeds of *H. pedunculosum* (Ser.) C.B. Clarke (Wei et al., 2020). Furthermore, dehydrodiconiferyl alcohol (205) is also an FXR agonist found in the seeds of *H. dunculosum* (Ser.) C.B. Clarke. To confirm its agonistic activity, knockdown of the expression of FXR in L-02 cells was performed. The mRNA and protein levels of FXR as well as a range of keys downstream has been remarkably upregulated after treatment with 205, which were decreased in Si-FXR cells (Wei et al., 2021).

**Figure:**
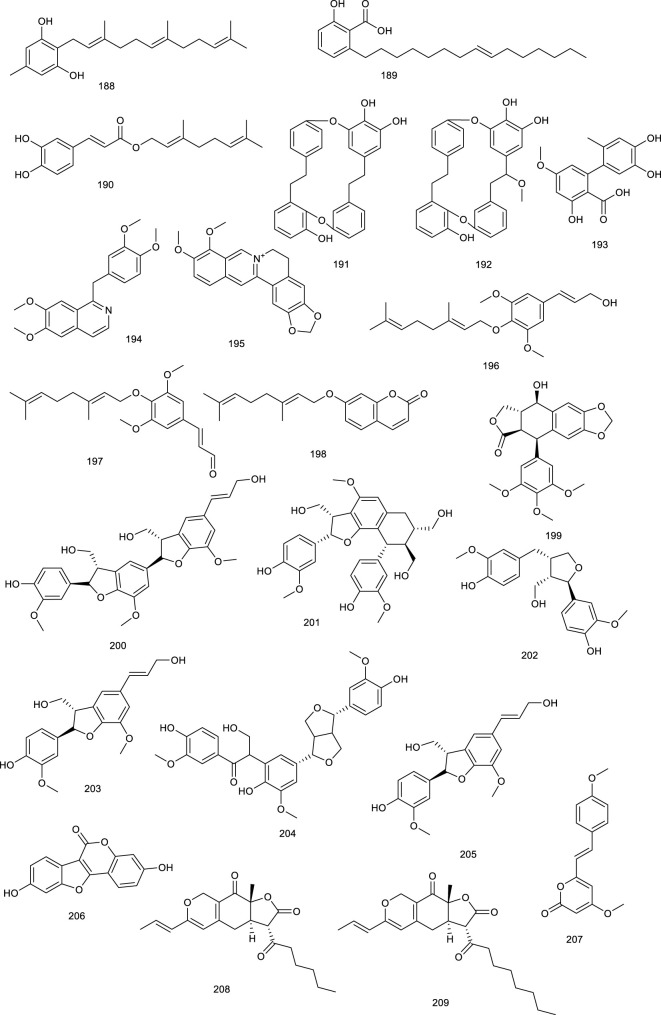


Soybean coumestrol (206) with 50 μM stimulated FXR transcriptional activity to 2.0-fold over the control group in HEK293T cells. ORO staining was employed to investigate the effect of 206 on differentiation of 3T3-L1 preadipocytes into mature adipocytes. It was observed that 206 dose-dependently suppressed MDI (mixture of 3-isobutyl-1-methylxanthine, dexamethasone, and insulin)-induced lipid accumulation in a manner, 206 at 20 or 40 µM decreased MDI-induced lipid accumulation by 38.8% and 77.4%, respectively. Together with RT-PCR analysis, 206 could be considered as an agent to prevent adipogenesis (Takahashi et al., 2008; Jang et al., 2016). Yangonin (207), a kavalactone found in Kava *Piper methysticum*, has been demonstrated to bind with the LBD of FXR and activate FXR to alleviate estrogen-caused cholestasis. Moreover, 207 caused an increase in BA efflux and detoxification as well as a decrease in BA uptake and synthesis via activating FXR, thereby exerting potential hepatoprotective effect. Moreover, in estrogen-induced cholestasis rat model, 17α-ethinylestradiol-induced growth retardation was reduced in a dose-dependent manner by 207, and relative liver weight was significantly decreased after treatment with 207, thereby alleviating estrogen-induced cholestasis (Dong et al., 2019).

#### 4.1.4 Others

Two secondary metabolites collected in *Monascus*-fermented rice, monascin (208) and ankaflavin (209), have been confirmed to upregulate FXR and PPARα expressions and, thus, result in fatty acid oxidation as food supplements to prevent against diabetes and obesity (Hsu et al., 2014).

### 4.2 Natural antagonists targeting farnesoid X receptor

#### 4.2.1 terpenes

Atractylenolides II (210) and III (211) are two naturally occurring sesquiterpenoids of *Atractylodes macrocephala* Koidz. responsible for treating excessive vaginal bleeding and exerting antihyperlipidemic effect. Compounds 210 and 211 at 50 µM exerted approximately 50% antagonistic effect against SHP gene promoter transactivation activity induced by 10 µM CDCA. Intriguingly, 100 µM of 210 and 211 entirely antagonized the transactivation activity of SHP in HepG2 cells at 0.9 and 0.85, respectively, induced by 10 µM of CDCA, which confirmed the function as FXR antagonists (Tsai et al., 2012). Andrographolide (212), a major constituent of “King of Bitte” *Andrographis panniculata* (Burm.f.) Nees, contributes to the pharmacological efficacy of this herb, owning medicinal potencies including anti-inflammation, antidiabetic, and anticancer (Kandanur et al., 2019; Kumar et al., 2020). In a study using transient transfection and FXR luciferase reporter assay in 293T cells, 212 was proven to be an FXR antagonist with the IC_50_ value of 9.7 µM (Liu et al., 2014).

**Figure:**
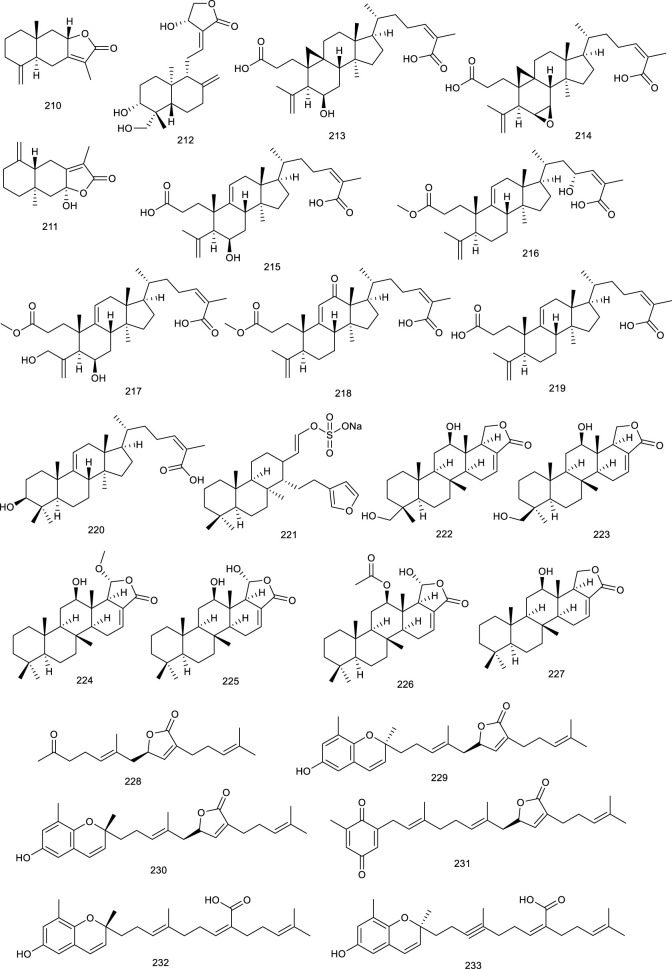


Oleanolic acid (157) not only activates LXRs as a promising treatment for NAFLD but also functions as an antagonist of FXR. It could bind with the LBD of FXR and inhibit the activity of FXR–LBD induced upon an endogenous ligand CDCA (Liu and Wong 2010). It also ameliorated obstructive cholestasis in mice after treatment with bile duct ligation due to FXR antagonism. Further histological and biochemical assessment showed that pretreatment with 20 mg/kg of 157 significantly ameliorated pathological alteration, such as extrahepatic cholestatic liver injury, bile duct hyperplasia, and portal infiltrates along with extensive foci of liver necrosis in bile duct-ligation mice (Chen et al., 2015a). *Schisandra glaucescens* Diels is both a traditional medicine and a food locally, as a potential candidate for the therapy of spontaneous sweat, night sweat, chronic diarrhea, along with neurasthenia in Hubei and Sichuan provinces of China. Cycloartane triterpenoids 6β-hydroxynigranoic acid (213) and schiglausin N (214), from the stems of *S. glaucescens* Diels, exhibited significant inhibitory effect on FXR as antagonists. Compound 213 restrained FXR with an IC_50_ of 1.50 μM, and simultaneously 214 emerged with the inhibitory rate of FXR of more than 20% at 25 μM (Zou et al., 2012a). Other six triterpenoids from *S. glaucescens* Diels, schiglausins D–G (215–218), kadsuric acid (219), and 3β-hydroxy-lanost-9(11),24-dien-26-oic acid (220), revealed antagonistic activity against FXR together with the inhibitory rate higher than 20% at 25 µM (Zou et al., 2012b). An FXR antagonist, platycodin D (22), from *Platycodon grandiflorum* (Jacq.) A.DC., is capable of modulating hepatic lipogenesis in high glucose-exposed HepG2 cells and in HFD-fed rats (Hwang et al., 2013). Treatment with 22 exhibited an antagonistic ratio at 40%–50%, compared with the control value of CDCA in a coactivator recruitment assay for FXR as a novel cholesterol-lowering and anti-atherogenic candidate (Zhao et al., 2006).

Marine organisms, such as sponges and tunicates, have received remarkable interests as a valuable source of novel bioactive natural products encompassing diverse chemical structures and pharmacological activities. Suvanine (221), a sesterterpene present in sponge *Coscinoder mamathewsi*, has been confirmed to be an FXR antagonist, effectively antagonizing FXR transactivation (Di Leva et al., 2013). In addition, 12-*O*-deacetyl-12-epi-19-deoxy-21-hydroxyscalarin (222), 12-*O*-deacetyl-12-epi-19-deoxy-22-hydroxyscalarin (223), 12-*O*-deacetyl- 12-*epi*-19-*O*-methylscalarin (224), 12-*O*-deacetyl-12-epi-scalarin (225), 12-epi-scalarin (226), and 12-*O*-deacetyl-12-*epi*-19-deoxyscalarin (227) found in the genus of *Spongia* also antagonized FXR transactivation. They decreased the affinity of FXR LBD for a coactivator peptide facilitated by CDCA in SPR assay, especially for 224–227, disrupted very strong interaction between FXR and a coactivator peptide (Nam et al., 2006; Nam et al., 2007). An isoprenoid tuberatolide A (228), and five meroterpenoids tuberatolide B (229), 2′-epi-tuberatolide B (230), yezoquinolide (231), *R*-sargachromenol (232), and *S*-sargachromenol (233), were isolated from Korean marine tunicate *Botryllus tuberatus*, and all of them exhibited significant antagonism on FXR with IC_50_ values as low as 1.5 µM. Compounds 228–233 released the coactivator peptide from the CDCA-bound hFXR LBD in cell-free SPR experiments, indicating the characterization of their potent FXR antagonists (Choi et al., 2011).

#### 4.2.2 Steroids

Guggulsterone, existing in two isomeric forms, namely, *E*-guggulsterone (234) and *Z*-guggulsterone (235), is the active agent isolated from the resin of *Commiphora mukul* (Arn.) Bhandari, responsible for antihyperlipidemic effect. *E*/*Z*-guggulsterones 234 and 235 have been identified as FXR antagonists directly, to decrease hepatic cholesterol levels in rodent models. Moreover, *Z*-guggulsterone (235) exhibited remarkable FXR antagonism with an IC_50_ of 1–5 μM in HepG2 cells, increased the cholesterol CYP7A1, and further decreased the circulating cholesterol level (Urizar et al., 2002; Urizar and Moore, 2003; Bhutani et al., 2007; Yu et al., 2009; Singh and Sashidhara, 2017). A very well established FXR antagonist from a natural source is stigmasterol acetate (226), a principal phytosterol extracted from soybeans. Compound 236 inhibited the expression of FXR target genes induced by CDCA in a Gal4-responsive luciferase reporter gene assay in HepG2 cells. Intriguingly, except for FXR, 236 also antagonized the LBD of PXR (Carter et al., 2007).

Ergostane-3,4,21,26-tetrol 3,21-bis (hydrogensulfate) (237) is a sulfated polyhydroxy sterol present in the marine invertebrate *Ophiolepis superba*. It resulted in a potent antagonism of FXR transactivation concentration dependently, and treatment with 237 at 100 μM completely reversed the effect exerted by CDCA on FXR expression. Taken together, 237 might be a potential candidate in regulating BA metabolism (Sepe et al., 2011; Fiorucci et al., 2012b). Some of polyhydroxylated steroids isolated from marine sponge *Theonella swinhoei* have been identified as FXR antagonists.

Swinhosterol B (238), conicasterols I (239), J (240), and conicasterol (241) are polyhydroxylated steroids identified from *Theonella swinhoei*. In a luciferase reporter assay transfected with FXR and RXR, 238 at 50 μM showed the most potential antagonism of 10 μM of CDCA-induced FXR, followed by 239–241. Simultaneously, 238–241 were also endowed with a potent PXR agonistic activity (De Marino et al., 2012; Fiorucci et al., 2012a). 4-Methylene-24-ethylsteroid, also called theonellasterol (242), also isolated from *T. swinhoei*, directly antagonized the activation of FXR at 50 μM and activated PXR as well (Fiorucci et al., 2012a; Renga et al., 2012; Sepe et al., 2014). Other eight polyhydroxylated steroids from *T. swinhoei*, conicasterols B–D (243–245), theonellasterols B, C, and E–G (246–250), functioned as FXR antagonists in the presence of CDCA with 10 μM, but also behaved as PXR agonists (De Marino et al., 2011, Fiorucci et al., 2012). Moreover, conicasterol E (251), from the same sponge, also has been demonstrated as an SBARM endowed with PXR agonistic activity (Sepe et al., 2012). 3-Oxocholest-1, 22-dien-12β-ol (252) and 3-oxocholest-1, 4-dien-20β-ol (253), two sterols from the soft coral *Dendronephthya gigantea*, have been verified as FXR antagonists, as assessed by a cotransfection assay in CV-1 cells, which exhibited notable antagonistic activity against FXR with IC_50_ values of 14 and 15 μM (Shin et al., 2012).

**Figure:**
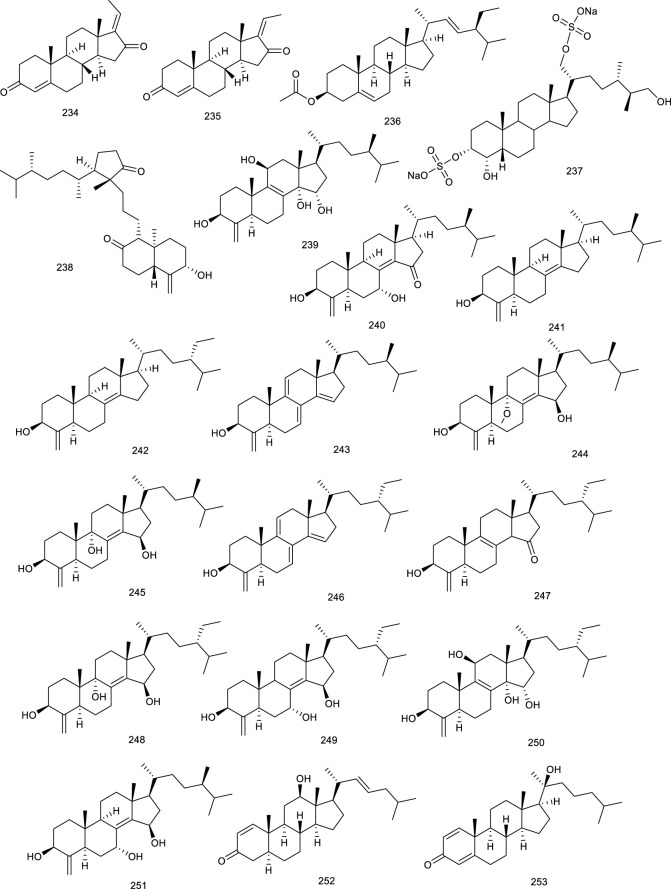


#### 4.2.3 Others

Three PUFAs FXR antagonists, arachidonic acid (146), docosahexaenoic acid (254) from *Crocodylus siamensis*, and linolenic acid (255) from *Perilla frutesccns*, exhibited antagonistic activity of CDCA-activated FXR with IC_50_ ranging from 0.9 to 4.7 μM (Zhao et al., 2004). Naringin (256), one of the most abundant and common flavonoids in citrus fruits and grapefruits, has been reported to relieve atherosclerosis. In ApoE^−/−^mice fed with an HFD, 256 ameliorated atherosclerosis, lowered the serum and liver cholesterol levels by 24.04% and 28.37%, increased lipid and BA excretion in feces, and regulated the gut microbiota associated with cholesterol metabolism. Moreover, 256 upregulated CYP7A1 via suppressing FXR/FGF15 pathway, and facilitated BA synthesis, providing insight into the atherosclerosis-amelioration mechanisms (Wang J. et al., 2020). In addition, 256 also could exert hepato- and nephroprotective effect in rats via the role of FXR and renal injury molecule-1 (Adil et al., 2016).

Maninsigins A (257), isolated from *Magnolia officinalis* as a TCM for the therapy of asthma, dyspepsia, and asthmatic cough, suppressed FXR activation induced by CDCA with an IC_50_ value of 55.6 μM as an FXR antagonist (Shang et al., 2013). An isoquinoline alkaloid palmatine (258, PAL) from *Rhizoma coptidis* contributes to the pharmacological efficacy of the herb such as obesity, diabetes mellitus, hyperlipidemia, and hyperglycemia–cardiovascular diseases. PAL-treated rats were characterized by decreased TC, TG, low-density lipoprotein cholesterol levels, serum total bile acid (TBA) levels, and increased fecal TBA and TC levels. It showed that 258 reduced HFD-induced lipid accumulation in the liver. So, it indicated the curative effect of 258 for hyperlipidemia (Ning et al., 2020).

**Figure:**
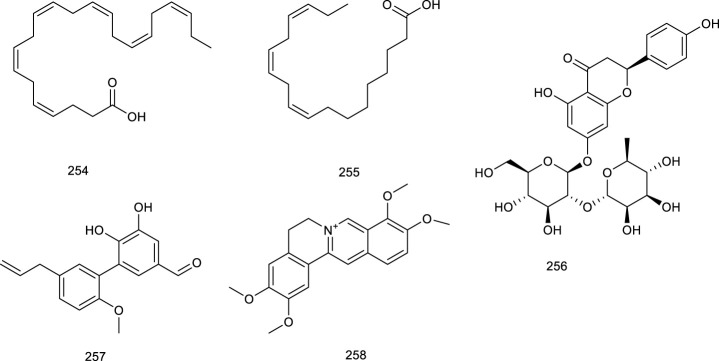


Halicylindramides F (259), A (260), and C (261) are three naturally occurring depsipeptides from a *Petrosia* species marine sponge collected in Korean waters. In a cotransfection assay in CV-1 cells, 259–261 possessed potent inhibitory effects of CDCA-induced hFXR transactivation with IC_50_ values of 6.0, 0.5, and 5.0 μM, respectively. Nevertheless, the function of hFXR antagonists they exerted did not bind directly to hFXR. However, SPR assay showed that 259–261 did not appear to have any significant inhibition of the recruitment of cofactor coactivator peptide to hFXR at concentrations higher than their IC_50_ values determined in a cell-based cotransfection assay. Thereby, the antagonism between FXR and 259–261 might be achieved by an indirect manner, not by a direct binding to LBD of FXR (Hahn et al., 2016).

**Figure:**
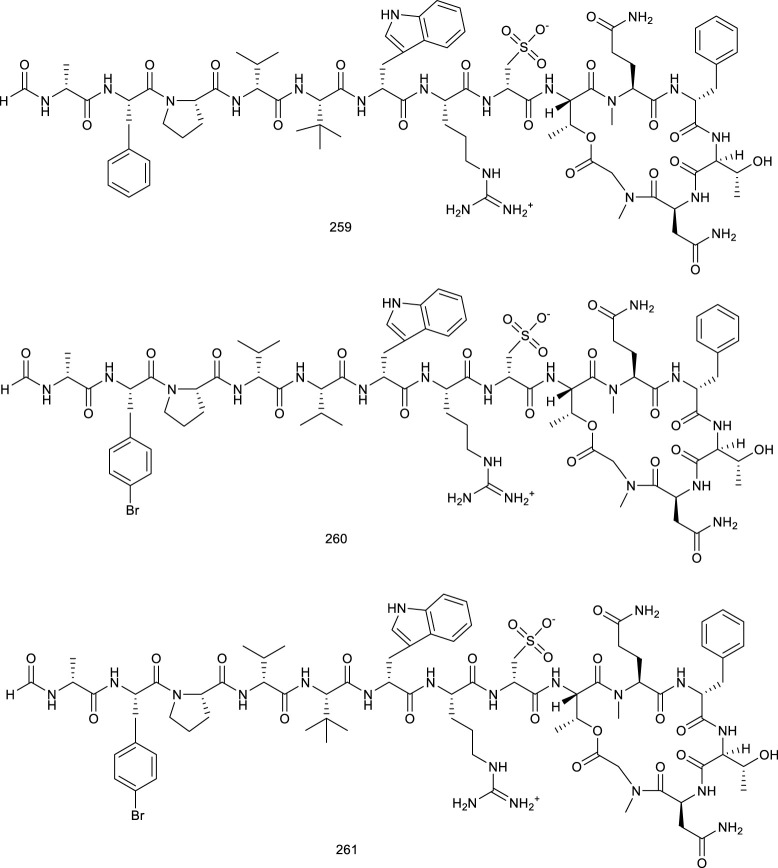


## 5 Molecular docking analysis of liver X receptors and farnesoid X receptor

*In silico* molecular docking is an important virtual screening method in the drug discovery process, which mainly focuses on structures of targets and ligands. It predicts the binding geometries as well as the binding energy of the ligand–target complex. To better understand the above-reported active natural products targeting LXR/FXR, most of them were performed together with molecular docking analyses, using the same method and parameters, although similar work has been done on some compounds in the literatures. As results, some natural compounds, like 7, 47, 54, 58, 60, 76, 77, 92, 93, 94, 136, 145, 156, 170, 189, 223, 226, 230, 232, 236, 238, 240, 244, 249, 250, 257, and 258, exhibited optimal property for targeting LXRs or FXR in molecular docking evaluation, suggesting that these compounds are even more promising.

### 5.1 Molecular docking analysis of liver X receptors

Based on the abovementioned LXR ligands, we have then analyzed, by means of molecular docking calculations, the interactions between LXRs ligands and LXRs proteins to understand and shed light on the binding mechanism of LXRs ligands. The crystal structures of the LXRα LBD in a complex with T0901317 (PDB ID: 1UHL) (Svensson et al., 2014) and GW3965 (PDB ID: 3IPQ) (Fradera et al., 2010), along with the LXRβ LBD in a complex with 2 (PDB ID: 1P8D) (Williams et al., 2003), were selected and subjected to an *in silico* molecular docking analysis, using the induced-fit module in the Schrödinger software suite. Molecular docking results (S values listed in Supplementary Tables S1 and S2) indicated that majority of the compounds fitted comfortably into the binding pocket, and the most potential 2D and 3D binding models of each protein are displayed in Supplementary Figures S1–S3, respectively.

As the docking results shown in Supplementary Figure 1A, the hydroxy in the saccharide moiety of 27 formed hydrogen bonds with the active-site residue SER 228 of 1UHL, the S value of which was −8.779, followed by 130 (−8.770), and 47 (−8.732), comparative with the ligand T0901317 (−8.931). In line with the abovementioned SPR assay of 33, 81, 102, 103, and 141, they could directly combine with LXRα protein 1UHL with S values from −5.763 to −9.187 (Supplementary Figures S1B–F). In the 2D binding models, the hydroxy or carbonyl groups in the hexatomic ring formed hydrogen bonds with the active site residue THR258, HID421, LYS434, THR302, TRP443, SRE264, or ARG305 of LXRα.

The docking model between LXRα 3IPQ and 58 along with 60 (Supplementary Figures S2A,B) exhibited that 58 and 60 formed hydrogen bonds with the active-site residue PHE 257, and the hexatomic ring plays a key role to form a π–π stacking interaction with PHE 315 with the docking score of −10.776 and −10.567 individually. Compounds 33, 81, 102, 103, 141, 142, and 144 also appeared to interact with LXRα protein 3IPQ perfectly with the S values from −5.763 to −9.187 (Supplementary Figures S2C–I), consistent with the corresponding SPR assay. 2D binding models showed that the phenolic hydroxy groups in the hexatomic ring formed hydrogen bonds with the active site residue ARG305, GLU267, MET298, THR302, PHE257, or SER264 of LXRα. Additionally, the aromatic hexatomic ring of 33, 103, and 141 formed a π–π stacking interaction with PHE 315 or PHE 326 of LXRα.

As depicted in Supplementary Figures S3A–D, 94, 76, 27-hydroxycholesterol, and its own ligand 24(S),25-epoxycholesterol all fitted well into LXRβ 1P8D and formed a hydrogen bond with ASN 239. Moreover, 27-hydroxycholesterol contained an additional hydrogen bond with HID 435. In fact, with respect to compounds 76, 77, 92–94, and 125, the S values of which are from −10.056 to −11.457, were better than or comparative to 24(S),25-epoxycholesterol (−9.941). Overall, ASN 239, GLU 281, and HID 435 might be regarded as key residues for the interactions and activity of 1P8D. In accordance with the SPR assay of 33, 81, and 141, they could directly combine with LXRβ protein 1P8D with the S values from −5.578 to −8.715 (Supplementary Figures S3E–G). The phenolic hydroxy groups in the hexatomic ring formed hydrogen bonds with the active site residues ASN239, GLU281, THR316, or MET312 of LXRβ, and there exist the formation of π–π stacking and π cation with PHE239 or PHE 243.

### 5.2 Molecular docking analysis of farnesoid X receptor

To gain an insight into the molecular interactions between FXR ligands and FXR proteins as well as generate a structure and activity relationship at the atomic level, molecular docking calculations were carried out using the crystal structures of the FXR LBD in a complex with obeticholic acid (PDB ID: 1OSV and 1OT7) (Mi et al., 2003) and DB08220 (PDB ID: 3BEJ) (Soisson et al., 2008) to explore the molecular interactions (S values listed in Supplementary Tables S3, S4). The most potential 2D and 3D binding models of each protein are shown in Supplementary Figures S4–S6, respectively.

As illustrated in Supplementary Figures S4A, B, 239 and 250 occupied the LBD similar to obeticholic acid (the S value −11.769) with similar interaction (Supplementary Figure S4B3), which bound to 1OSV with the S values of −10.917 and −10.415, respectively. The hydroxy groups in the steroidal ring moiety of these molecules formed hydrogen bonds with the active-site residue LEU 284 and SER 329. Overall, the docking models 235 also showed the most potency with a docking score over −10. Compounds 222–233 were a series of steroids isolated from the genus of *Spongia*. SPR assay indicated that they all decreased the affinity of FXR LBD for a coactivator peptide facilitated by CDCA. In line with SPR, docking indicated that they fitted comfortably into the binding pocket for the agonist obeticholic acid with the similar binding positions, with the S values from −8.941 to −9.179, except for 225 with the S value of −5.849 (Supplementary Figures S4C–N). The ester and phenolic hydroxyl groups formed hydrogen bonds with the active-site residue ARG 328 or TYR358 of FXR. Furthermore, both 226 and 227 formed π–π stacking interaction with TRP451 and TYR328.

Compound 168 interacted with FXR 1OT7 with the highest S value of −10.884, comparative to the ligand obeticholic acid (docking score −11.569) and CDCA (docking score −10.610). The hydroxy groups formed hydrogen bonds with the active-site residue TYR 358, HID 444, LEU 284, and ARG 328 (Supplementary Figure 5A). Meanwhile, 163, 218, and 168 also ideally fitted into the LBD of 1OT7 along with a docking score over −10. 2D binding models of 222–233 with 1OT7 supporting the corresponding SPR results excellently with the S values from −7.906 to −9.352, except for 225 with the S value of −5.899 (Supplementary Figures S5B–M). The ester and phenolic hydroxyl groups of 226, 232, and 236 formed hydrogen bonds with the active-site residue ARG 328 or TYR358 of FXR.

Compound 233 docked into FXR 3BEJ with the highest score of −11.025, though only inferior to DB08220 (the S value of −12.625), both formed hydrogen bonds with the active-site residue THR 288 and ARG 331. Differently, there is a salt bridge between DB08220 and ARG 331 of 3BEJ. The docking models of 233, 204, 244, 252, 228, 230, 253, and 255 predicted the significant role of the active-site residue THR 288 and ARG 331. Interestingly, direct binding of 233, along with 222–232 to the LBD of FXR, was monitored by SPR assay. In molecular docking, they also fitted comfortably into the binding pocket for the agonist obeticholic acid with the similar binding positions as well, with the S values from −8.894 to −10.113, except for 228 with the S value of −5.861 (Supplementary Figures S6A–K). In the 2D binding models, the ester and hydroxyl groups formed hydrogen bonds with the active-site residue THR288, ARG331 or HID447 of FXR.

## 6 Conclusion and perspective

Concurrent with our increasing knowledge of the roles of LXRs/FXR in cholesterol, lipid, bile acid, and glucose metabolism, selective and potent LXRs/FXR ligands have gained significant ground for shedding light on the functions of LXRs and FXR in metabolic syndrome-related diseases as promising chemical biology tools (Jian et al., 2014; Pu et al., 2019).

**FIGURE 4:**
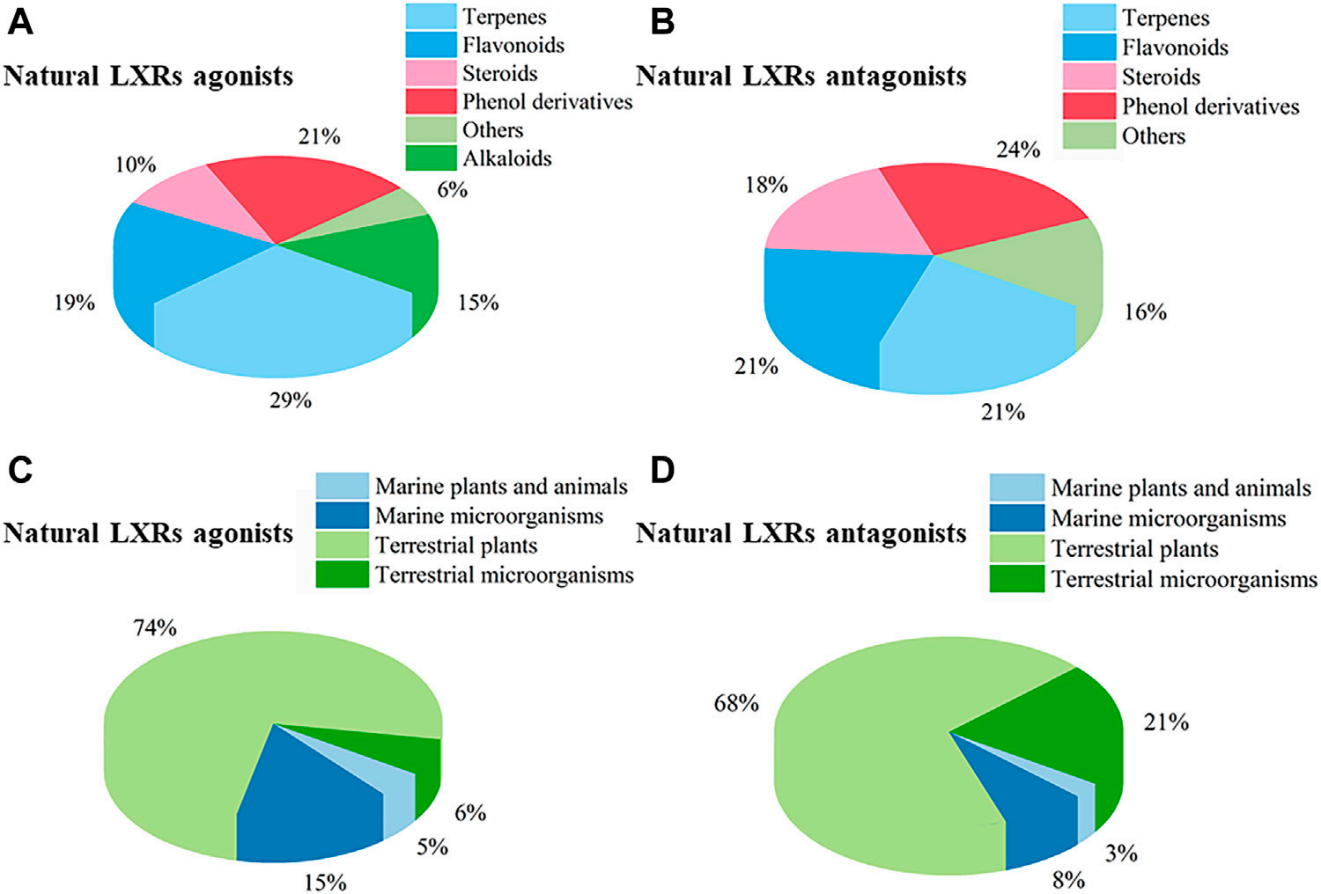
Chemical types **(A, B)** and resources **(C, D)** of natural-derived LXRs agonists and antagonists.

**FIGURE 5:**
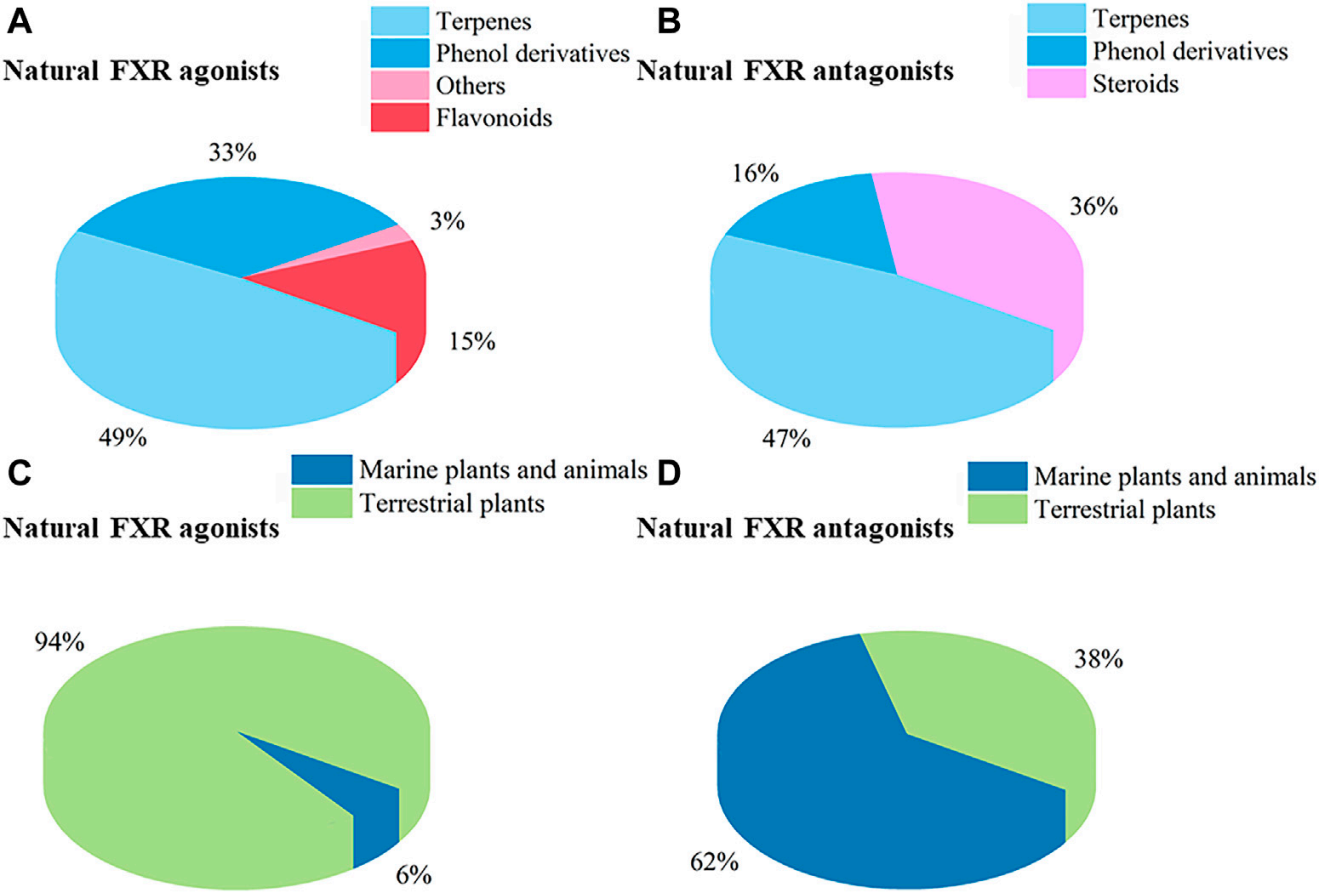
Chemical types **(A, B)** and resources **(C, D)** of natural-derived FXR agonists and antagonists.

In the recent two decades (2000–2020), 261 natural products were discovered from natural resources, such as LXRs/FXR modulators, 109 agonists and 38 antagonists targeting LXRs, while 72 agonists and 55 antagonists are targeting FXR. As shown in Figure 4 and Figure 5, LXR ligands exhibited higher chemical diversity than FXR ligands, terpenes, flavonoids, phenol derivatives, and steroids constituted 29%, 19%, 21%, and 10% of the reported LXR agonists, constituted 21%, 21%, 24%, and 18% of the reported LXR antagonists, respectively, while for FXR ligands, terpenoids accounted for a large proportion, close to half, for 49% and 47% of the reported FXR agonists and antagonists, respectively. Sterols, primary type of FXR antagonists, the reason of which is a series of endogenous BAs that possess distinguishing potency of targeting BA-receptor FXR. It is a remarkable fact that several of the mentioned natural products are food constituents, such as compounds 41, 42, 54, 95, 96, 104, 106, 125, 127, 208, 210–212, and 214–220, thus, potential candidates for dietary interventions for the prevention and treatment of corresponding diseases. Terrestrial plants are a promising source for the discovery of new drug leads, especially for TCMs having a history of more than 2,000 years. In recent years, marine organisms and microorganisms have gradually become a vital source of LXRs/FXR modulators with the development of marine natural product research. For instance, 38% of FXR antagonists originated from marine organisms. Especially for 222–227 and 228–233, a series of steroids isolated from the genus of *Spongia* have been verified as FXR antagonists via various representative assays, including SPR, indicating the significance of discovering marine natural steroids associated with FXR in the genus of *Spongia*. Overall, it is believed and prospective that LXR/FXR modulators from marine sources, especially marine microbial sources, will take a greater proportion in the future.

LXRs and FXR are highly expressed in the livers of patients with NAFLD, increasing with the severity of NASH (Zheng Z. et al., 2017, Mi et al., 2019). Some natural compounds especially for 2, 3, 37, 42, 83, 104, 106, 114, 115, 117, 118, 122, 132–134, 141, 157, 164, 167, 178, and 193, proved effective in ameliorating NAFLD and NASH by targeting LXR/FXR or through the LXR/FXR pathway, presenting a promising therapeutic prospect. Risk of cardiovascular diseases has been reported to be associated with cellular cholesterol levels, that is, with excess cellular cholesterol often increasing the risk of cardiovascular diseases (Jun et al., 2013). Our review discovered that 3, 22, 39, 40, 44, 54, 76, 77, 82, 83, 99, 107–109, 152, 210, 211, 256, and 258 played a vital role in hypercholesterolemia, hypertension, or atherosclerosis, indicating the prospect of further exploration in cardiovascular diseases focusing on LXR/FXR ligands. Additionally, 25, 38, 47, 48, 83, 152, 154, 155, 157, 177, and 183–185 have represented tremendous potential for the treatment of various liver diseases including HBV, HCV, hepatic fibrosis, acute lung injury, cholestatic liver injury, liver cancer, and cholestasis. Previous reports have elicited that the ligands targeting the LXR/FXR might be one of the most effective ways to intervene with diabetes and obesity (Pu et al., 2019). It is suggested that 46, 114, 116, 124, 156, 157, 195, 208, and 209 could be developed as prospective candidates to reduce the risk of diabetes and obesity. Intriguingly, 51, 52, 77, and 96 have proved to be beneficial for the treatment of AD; meanwhile, 22, 78, 98, and 99 have contributed to alleviate the development of mastitis and breast cancer. Overall, the abovementioned provides multiple alternative approaches to LXR/FXR-related drug development.

At this point it needs, nevertheless, to be mentioned that most lead compounds failed in preclinical and clinical developments mostly due to toxicological and/or pharmacokinetic issues. For instance, the clinical application of LXRα agonist triptolide (19) is greatly constrained by hepatotoxicity, suggesting the risk of LXRs as toxicity targets. Our recent study revealed that, different from PA (142) directly combined with LXRα as an inhibitor, another anti-RCC drug candidate GPA (143) exists as a prodrug in the liver and exerts a toxic effect due to transformation into PA. Longer survival time of GPA-treated mice indicates that further exploration in anti-RCC drug research should focus on reducing glycosides transformed into PA and concentrating in kidney tumor rather than the liver for lowering hepatotoxicity risk.

Thereby, the therapeutic effects of these on diseases associated with LXRs and FXR expression remain to be further investigated in preclinical or clinical studies. Even if some compounds possessed grand effects on LXRs or FXR at a high concentration without toxicity in cell models, there exists a difference *in vivo*. Besides, overactivation or repression of LXRs and FXR is likely to cause other diseases, the discovery of the right balance between activating and inhibiting LXRs and/or FXR effects will be crucial for the development of targeting agents.

## Glossary

ABCA1: ATP-binding cassette transporters A1
ABCG1: ATP-binding cassette transporters G1
ABCG5: ATP-binding cassette transporters G5
ABCG8: ATP-binding cassette transporters G8
AD: alzheimer’s disease
AF-1: activation function-1
AF-2: activation function-2
ALT: alanine aminotransferase
AMPK: adenosine 5′-monophosphate-activated protein kinase
ANIT: α-naphthylisothiocyanate
APOAI: apolipoprotein AI
APOC1: apolipoproteins C1
APOC2: apolipoproteins C2
APOC4: apolipoproteins C3
APOE: apolipoproteins E
AST: aspartate aminotransferase
BA: bile acid
CDCA: chenodeoxycholic acid
CYP7A1: cholesterol 7α-hydroxylase
DBD: DNA-binding domain
DCA: deoxycholic acid
EC_50_: half maximal effective concentration
FGF19: fibroblast growth factor 19
FXR: farnesoid X receptor
GLUT4: glucose transporter type 4
G6P: glucose-6-phosphatase
GPA: glucopiericidin A
HBV: hepatitis B virus
HCV: hepatitis C virus
HDL: high-density lipoprotein
HFD: high-fat diet
HPLC-ESI-MS: high-performance liquid chromatography-electrospray ionization mass spectrometry
IBD: inflammatory bowel disease
IC_50_: half maximal inhibitory concentration
IRS-1: insulin substrate receptor 1
*K*_*D*_: dissociation equilibrium constant
LBD: ligand-binding domain
LCA: lithocholic acid
LCAT: cholesterol acyltransferase enzyme
LDL-C: low-density lipoprotein-cholesterol
LDLR: low-density lipoprotein receptor
LPS: lipopolysaccharide
LXRs: liver X receptors
LXREs: LXRs response elements
MDI: mixture of 3-isobutyl-1-methylxanthine, dexamethasone and insulin
NAFLD: nonalcoholic fatty liver disease
NASH: nonalcoholic steatohepatitis
NLRP3: nucleotide-binding oligomerization domain, leucine-rich repeat and pyrin domain-containing 3
NMR: nuclear magnetic resonance
NPC1: Niemann–Pick C1
NPC2: Niemann-Pick C2
NPC1L1: Niemann-Pick C1-like 1
NRs: nuclear receptors
NR0: miscellaneous
NR1: thyroid hormone receptor-like
NR2: retinoid X receptor-like
NR3: estrogen receptor-like
NR4: nerve growth factor IB-like
NR5: steroidogenic factor-like
NR6: germ cell nuclear factor-like
NF-κB: nuclear factor kappa-B
PA: piericidin A
PEPCK: phosphoenolpyruvate carboxy kinase
PPAR: peroxisome proliferator-activated receptor
PUFAs: polyunsaturated fatty acids
qPCR: quantitative polymerase chain reaction
RCC: renal cell carcinoma
RCT: reverse cholesterol transport
RT-qPCR: real-time quantitative polymerase chain reaction
RT-PCR: real-time polymerase chain reaction
RXR: retinoid X receptor
S value: negative-binding free energy value
SBARM: A selective bile acid receptor modulator
SHP: small heterodimer partner
SPR: surface plasmon resonance
SREBPs: sterol-regulatory element-binding proteins
TBA: total bile acid
TC: total cholesterol
TCMs: traditional Chinese medicines
T2D: type 2 diabetes
TG: triglyceride
TFA: total flavonoids of *Astmgali Radix*

## References

[B1] Abdullah S. Jang S. E. Kwak M. K. Chong K. (2020). Ganoderma Boninense Mycelia for Phytochemicals and Secondary Metabolites with Antibacterial Activity. J. Microbiol. 58, 1054–1064. 10.1007/s12275-020-0208-z 33263896

[B2] Adil M. Kandhare A. D. Ghosh P. Venkata S. Raygude K. S. Bodhankar S. L. (2016). Ameliorative Effect of Naringin in Acetaminophen-Induced Hepatic and Renal Toxicity in Laboratory Rats: Role of FXR and KIM-1. Ren. Fail. 38, 1007–1020. 10.3109/0886022X.2016.1163998 27050864

[B3] Apfel R. Benbrook D. Lernhardt E. Ortiz M. A. Salbert G. Pfahl M. (1994). A Novel Orphan Receptor Specific for a Subset of Thyroid Hormone-Responsive Elements and its Interaction with the Retinoid/thyroid Hormone Receptor Subfamily. Mol. Cel. Biol. 14, 7025–7035. 10.1128/mcb.14.10.7025 PMC359232 7935418

[B4] Asakawa Y. Ludwiczuk A. (2018). Chemical Constituents of Bryophytes: Structures and Biological Activity. J. Nat. Prod. 81, 641–660. 10.1021/acs.jnatprod.6b01046 29019405

[B5] Asakawa Y. (2008). Recent Advances of Biologically Active Substances from the Marchantiophyta. Nat. Product. Commun. 3, 1934578X0800300–92. 10.1177/1934578X0800300116

[B6] Auger F. Martin F. Pétrault O. Samaillie J. Hennebelle T. Trabelsi M. S. (2018). Risperidone-induced Metabolic Dysfunction Is Attenuated by Curcuma Longa Extract Administration in Mice. Metab. Brain Dis. 33, 63–77. 10.1007/s11011-017-0133-y 29034440

[B7] Bełtowski J. (2008). Liver X Receptors (LXR) as Therapeutic Targets in Dyslipidemia. Cardiovasc. Ther. 26, 297–316. 10.1111/j.1755-5922.2008.00062.x 19035881

[B8] Bhutani K. K. Birari R. Kapat K. (2007). Potential Anti-obesity and Lipid Lowering Natural Products: A Review. Nat. Product. Commun. 2, 1934578X0700200–348. 10.1177/1934578X0700200316

[B9] Bogie J. Hoeks C. Schepers M. Tiane A. Cuypers A. Leijten F. (2019). Dietary Sargassum Fusiforme Improves Memory and Reduces Amyloid Plaque Load in an Alzheimer’s Disease Mouse Model. Sci. Rep. 9, 4908. 10.1038/s41598-019-41399-4 30894635 PMC6426980

[B10] Bonamassa B. Moschetta A. (2013). Atherosclerosis: Lessons from LXR and the Intestine. Trends Endocrinol. Metab. 24, 120–128. 10.1016/j.tem.2012.10.004 23158108

[B11] Bradley M. N. Hong C. Chen M. Joseph S. B. Wilpitz D. C. Wang X. (2007). Ligand Activation of LXR Beta Reverses Atherosclerosis and Cellular Cholesterol Overload in Mice Lacking LXR Alpha and apoE. J. Clin. Invest. 117, 2337–2346. 10.1172/JCI31909 17657314 PMC1924496

[B12] Bramlett K. S. Houck K. A. Borchert K. M. Dowless M. S. Kulanthaivel P. Zhang Y. (2003). A Natural Product Ligand of the Oxysterol Receptor, Liver X Receptor. J. Pharmacol. Exp. Ther. 307, 291–296. 10.1124/jpet.103.052852 12893846

[B13] Bruyère C. Genovese S. Lallemand B. Ionescu-Motatu A. Curini M. Kiss R. (2011). Growth Inhibitory Activities of Oxyprenylated and Non-prenylated Naturally Occurring Phenylpropanoids in Cancer Cell Lines. Bioorg. Med. Chem. Lett. 21, 4174–4179. 10.1016/j.bmcl.2011.05.089 21696954

[B14] Calkin A. C. Tontonoz P. (2012). Transcriptional Integration of Metabolism by the Nuclear Sterol-Activated Receptors LXR and FXR. Nat. Rev. Mol. Cel Biol. 13, 213–224. 10.1038/nrm3312 PMC3597092 22414897

[B15] Cao G. Liang Y. Broderick C. L. Oldham B. A. Beyer T. P. Schmidt R. J. (2003). Antidiabetic Action of a Liver X Receptor Agonist Mediated by Inhibition of Hepatic Gluconeogenesis. J. Biol. Chem. 278, 1131–1136. 10.1074/jbc.M210208200 12414791

[B16] Carazo A. Mladěnka P. Pávek P. (2019). Marine Ligands of the Pregnane X Receptor (PXR): An Overview. Mar. Drugs 17, 554. 10.3390/md17100554 PMC6836225 31569349

[B17] Carotti A. Marinozzi M. Custodi C. Cerra B. Pellicciari R. Gioiello A. (2014). Beyond Bile Acids: Targeting Farnesoid X Receptor (FXR) with Natural and Synthetic Ligands. Curr. Top. Med. Chem. 14, 2129–2142. 10.2174/1568026614666141112094058 25388537

[B18] Carter B. A. Taylor O. A. Prendergast D. R. Zimmerman T. L. Von Furstenberg R. Moore D. D. (2007). Stigmasterol, a Soy Lipid-Derived Phytosterol, Is an Antagonist of the Bile Acid Nuclear Receptor FXR. Pediatr. Res. 62, 301–306. 10.1203/PDR.0b013e3181256492 17622954

[B19] Castrillo A. Joseph S. B. Marathe C. Mangelsdorf D. J. Tontonoz P. (2003). Liver X Receptor-dependent Repression of Matrix Metalloproteinase-9 Expression in Macrophages. J. Biol. Chem. 278, 10443–10449. 10.1074/jbc.M213071200 12531895

[B20] Castro Navas F. F. Giorgi G. Maggioni D. Pacciarini M. Russo V. Marinozzi M. (2018). C24-hydroxylated Stigmastane Derivatives as Liver X Receptor Agonists. Chem. Phys. Lipids 212, 44–50. 10.1016/j.chemphyslip.2018.01.005 29352964

[B21] Cave M. C. Clair H. B. Hardesty J. E. Falkner K. C. Feng W. Clark B. J. (2016). Nuclear Receptors and Nonalcoholic Fatty Liver Disease. Biochim. Biophys. Acta 1859, 1083–1099. 10.1016/j.bbagrm.2016.03.002 26962021 PMC5149456

[B22] Cavelier C. Lorenzi I. Rohrer L. von Eckardstein A. (2006). Lipid Efflux by the ATP-Binding Cassette Transporters ABCA1 and ABCG1. Biochim. Biophys. Acta 1761, 655–666. 10.1016/j.bbalip.2006.04.012 16798073

[B23] Chen J. Tian J. Ge H. Liu R. Xiao J. (2017a). Effects of Tetramethylpyrazine from Chinese Black Vinegar on Antioxidant and Hypolipidemia Activities in HepG2 Cells. Food Chem. Toxicol. 109, 930–940. 10.1016/j.fct.2016.12.017 28034800

[B24] Chen P. Li J. Fan X. Zeng H. Deng R. Li D. (2015a). Oleanolic Acid Attenuates Obstructive Cholestasis in Bile Duct-Ligated Mice, Possibly via Activation of NRF2-MRPs and FXR Antagonism. Eur. J. Pharmacol. 765, 131–139. 10.1016/j.ejphar.2015.08.029 26297978

[B25] Chen P. Zeng H. Wang Y. Fan X. Xu C. Deng R. (2014a). Low Dose of Oleanolic Acid Protects against Lithocholic Acid-Induced Cholestasis in Mice: Potential Involvement of Nuclear Factor-E2-Related Factor 2-mediated Upregulation of Multidrug Resistance-Associated Proteins. Drug Metab. Dispos. 42, 844–852. 10.1124/dmd.113.056549 24510383

[B26] Chen Q. Wang T. Li J. Wang S. Qiu F. Yu H. (2017b). Effects of Natural Products on Fructose-Induced Nonalcoholic Fatty Liver Disease (NAFLD). Nutrients 9, 96. 10.3390/nu9020096 PMC5331527 28146130

[B27] Chen S. F. Chen P. Y. Hsu H. J. Wu M. J. Yen J. H. (2017c). Xanthohumol Suppresses Mylip/Idol Gene Expression and Modulates Ldlr Abundance and Activity in HepG2 Cells. J. Agric. Food Chem. 65, 7908–7918. 10.1021/acs.jafc.7b02282 28812343

[B28] Chen W. D. Wang Y. D. Meng Z. Zhang L. Huang W. (2011). Nuclear Bile Acid Receptor FXR in the Hepatic Regeneration. Biochim. Biophys. Acta 1812, 888–892. 10.1016/j.bbadis.2010.12.006 21167938

[B307] Chen X. L. Meng Q. Wang C. Y. Liu Q. Sun H. J. Huo X. K. (2015b). Protective Effects of Calycosin Against ccl4-Induced Liver Injury With Activation of fxr and Stat3 in Mice. Pharm. Res. 32, 538–548. 10.1007/s11095-014-1483-3 25143196

[B29] Chen Y. Lu W. Yang K. Duan X. Li M. Chen X. (2020). Tetramethylpyrazine: A Promising Drug for the Treatment of Pulmonary Hypertension. Br. J. Pharmacol. 177, 2743–2764. 10.1111/bph.15000 31976548 PMC7236078

[B30] Chen Z. Liu J. Fu Z. Ye C. Zhang R. Song Y. (2014b). 24(S)-Saringosterol from Edible marine Seaweed Sargassum Fusiforme Is a Novel Selective LXRβ Agonist. J. Agric. Food Chem. 62, 6130–6137. 10.1021/jf500083r 24927286

[B31] Cheng S. Liang S. Liu Q. Deng Z. Zhang Y. Du J. (2018). Diosgenin Prevents High-Fat Diet-Induced Rat Non-alcoholic Fatty Liver Disease through the AMPK and LXR Signaling Pathways. Int. J. Mol. Med. 41, 1089–1095. 10.3892/ijmm.2017.3291 29207101

[B32] Cheng Y. Mai J. Y. Wang M. F. Chen G. F. Ping J. (2017). Antifibrotic Effect of Total Flavonoids of Astmgali Radix on Dimethylnitrosamine-Induced Liver Cirrhosis in Rats. Chin. J. Integr. Med. 23, 48–54. 10.1007/s11655-016-2627-6 27787720

[B33] Chinetti-Gbaguidi G. Staels B. (2009). Lipid Ligand-Activated Transcription Factors Regulating Lipid Storage and Release in Human Macrophages. Biochim. Biophys. Acta 1791, 486–493. 10.1016/j.bbalip.2009.01.009 19416654

[B34] Choi H. Hwang H. Chin J. Kim E. Lee J. Nam S. J. (2011). Tuberatolides, Potent FXR Antagonists from the Korean marine Tunicate Botryllus Tuberatus. J. Nat. Prod. 74, 90–94. 10.1021/np100489u 21142112

[B35] Cragg G. M. Newman D. J. (2013). Natural Products: a Continuing Source of Novel Drug Leads. Biochim. Biophys. Acta 1830, 3670–3695. 10.1016/j.bbagen.2013.02.008 23428572 PMC3672862

[B36] Cuadrado I. Fernández-Velasco M. Boscá L. de Las Heras B. (2011). Labdane Diterpenes Protect against Anoxia/reperfusion Injury in Cardiomyocytes: Involvement of Akt Activation. Cell Death Dis 2, e229. 10.1038/cddis.2011.113 22071634 PMC3223697

[B37] Daugsch A. Moraes C. S. Fort P. Park Y. K. (2008). Brazilian Red Propolis-Cchemical Composition and Botanical Origin. Evid. Based Complement. Alternat Med. 5, 435–441. 10.1093/ecam/nem057 18955226 PMC2586321

[B38] De Boussac H. Alioui A. Viennois E. Dufour J. Trousson A. Vega A. (2013). Oxysterol Receptors and Their Therapeutic Applications in Cancer Conditions. Expert Opin. Ther. Targets 17, 1029–1038. 10.1517/14728222.2013.820708 23875732

[B39] De Fabiani E. Mitro N. Anzulovich A. C. Pinelli A. Galli G. Crestani M. (2001). The Negative Effects of Bile Acids and Tumor Necrosis Factor-Alpha on the Transcription of Cholesterol 7alpha-Hydroxylase Gene (CYP7A1) Converge to Hepatic Nuclear Factor-4: a Novel Mechanism of Feedback Regulation of Bile Acid Synthesis Mediated by Nuclear Receptors. J. Biol. Chem. 276, 30708–30716. 10.1074/jbc.M103270200 11402042

[B40] De Marino S. Ummarino R. D’Auria M. V. Chini M. G. Bifulco G. D’Amore C. (2012). 4-Methylenesterols from Theonella Swinhoei Sponge Are Natural Pregnane-X-Receptor Agonists and Farnesoid-X-Receptor Antagonists that Modulate Innate Immunity. Steroids 77, 484–495. 10.1016/j.steroids.2012.01.006 22285937

[B41] De Marino S. Ummarino R. D’Auria M. V. Chini M. G. Bifulco G. Renga B. (2011). Theonellasterols and Conicasterols from Theonella Swinhoei. Novel marine Natural Ligands for Human Nuclear Receptors. J. Med. Chem. 54, 3065–3075. 10.1021/jm200169t 21428459

[B42] Del Bas J. M. Ricketts M. L. Vaqué M. Sala E. Quesada H. Ardevol A. (2009). Dietary Procyanidins Enhance Transcriptional Activity of Bile Acid-Activated FXR *In Vitro* and Reduce Triglyceridemia *In Vivo* in a FXR-dependent Manner. Mol. Nutr. Food Res. 53, 805–814. 10.1002/mnfr.200800364 19496086 PMC4142053

[B43] Di Leva F. S. Festa C. D’Amore C. De Marino S. Renga B. D’Auria M. V. (2013). Binding Mechanism of the Farnesoid X Receptor marine Antagonist Suvanine Reveals a Strategy to Forestall Drug Modulation on Nuclear Receptors. Design, Synthesis, and Biological Evaluation of Novel Ligands. J. Med. Chem. 56, 4701–4717. 10.1021/jm400419e 23656455

[B44] Dong M. He X. Liu R. H. (2007). Phytochemicals of Black Bean Seed coats: Isolation, Structure Elucidation, and Their Antiproliferative and Antioxidative Activities. J. Agric. Food Chem. 55, 6044–6051. 10.1021/jf070706d 17602653

[B45] Dong R. Wang J. Gao X. Wang C. Liu K. Wu J. (2019). Yangonin Protects against Estrogen-Induced Cholestasis in a Farnesoid X Receptor-dependent Manner. Eur. J. Pharmacol. 857, 172461. 10.1016/j.ejphar.2019.172461 31220436

[B46] Duan X. Meng Q. Wang C. Liu Z. Liu Q. Sun H. (2017). Calycosin Attenuates Triglyceride Accumulation and Hepatic Fibrosis in Murine Model of Non-alcoholic Steatohepatitis via Activating Farnesoid X Receptor. Phytomedicine 25, 83–92. 10.1016/j.phymed.2016.12.006 28190475

[B47] Duval C. Touche V. Tailleux A. Fruchart J. C. Fievet C. Clavey V. (2006). Niemann-Pick C1 like 1 Gene Expression Is Down-Regulated by LXR Activators in the Intestine. Biochem. Biophys. Res. Commun. 340, 1259–1263. 10.1016/j.bbrc.2005.12.137 16414355

[B48] El-Gendy B. E. M. Goher S. S. Hegazy L. S. Arief M. M. H. Burris T. P. (2018). Recent Advances in the Medicinal Chemistry of Liver X Receptors. J. Med. Chem. 61, 10935–10956. 10.1021/acs.jmedchem.8b00045 30004226

[B49] Elia J. Carbonnelle D. Logé C. Ory L. Huvelin J. M. Tannoury M. (2019). 4-cholesten-3-one Decreases Breast Cancer Cell Viability and Alters Membrane Raft-Localized EGFR Expression by Reducing Lipogenesis and Enhancing LXR-dependent Cholesterol Transporters. Lipids Health Dis. 18, 168. 10.1186/s12944-019-1103-7 31477154 PMC6721338

[B50] Epifano F. Genovese S. James Squires E. Gray M. A. (2012). Nelumal A, the Active Principle from Ligularia Nelumbifolia, Is a Novel Farnesoid X Receptor Agonist. Bioorg. Med. Chem. Lett. 22, 3130–3135. 10.1016/j.bmcl.2012.03.057 22472691

[B51] Fan J. S. Liu D. N. Huang G. Xu Z. Z. Jia Y. Zhang H. G. (2012). Panax Notoginseng Saponins Attenuate Atherosclerosis via Reciprocal Regulation of Lipid Metabolism and Inflammation by Inducing Liver X Receptor Alpha Expression. J. Ethnopharmacol. 142, 732–738. 10.1016/j.jep.2012.05.053 22683903

[B52] Fiorucci S. Distrutti E. Bifulco G. D’Auria M. V. Zampella A. (2012a). Marine Sponge Steroids as Nuclear Receptor Ligands. Trends Pharmacol. Sci. 33, 591–601. 10.1016/j.tips.2012.08.004 23000093

[B53] Fiorucci S. Zampella A. Distrutti E. (2012b). Development of FXR, PXR and CAR Agonists and Antagonists for Treatment of Liver Disorders. Curr. Top. Med. Chem. 12, 605–624. 10.2174/156802612799436678 22242859

[B54] Forman B. M. Goode E. Chen J. Oro A. E. Bradley D. J. Perlmann T. (1995). Identification of a Nuclear Receptor that Is Activated by Farnesol Metabolites. Cell 81, 687–693. 10.1016/0092-8674(95)90530-8 7774010

[B55] Forman B. M. Ruan B. Chen J. Schroepfer G. J. Evans R. M. (1997). The Orphan Nuclear Receptor LXRalpha Is Positively and Negatively Regulated by Distinct Products of Mevalonate Metabolism. Proc. Natl. Acad. Sci. U S A. 94, 10588–10593. 10.1073/pnas.94.20.10588 9380679 PMC23411

[B56] Fradera X. Vu D. Nimz O. Skene R. Hosfield D. Wynands R. (2010). X-ray Structures of the LXRalpha LBD in its Homodimeric Form and Implications for Heterodimer Signaling. J. Mol. Biol. 399, 120–132. 10.1016/j.jmb.2010.04.005 20382159

[B57] Francisco V. Figueirinha A. Costa G. Liberal J. Ferreira I. Lopes M. C. (2016). The Flavone Luteolin Inhibits Liver X Receptor Activation. J. Nat. Prod. 79, 1423–1428. 10.1021/acs.jnatprod.6b00146 27135143

[B58] Fu Y. Hu X. Cao Y. Zhang Z. Zhang N. (2015). Saikosaponin a Inhibits Lipopolysaccharide-Oxidative Stress and Inflammation in Human Umbilical Vein Endothelial Cells via Preventing TLR4 Translocation into Lipid Rafts. Free Radic. Biol. Med. 89, 777–785. 10.1016/j.freeradbiomed.2015.10.407 26475038

[B59] Gadaleta R. M. Cariello M. Sabbà C. Moschetta A. (2015). Tissue-specific Actions of FXR in Metabolism and Cancer. Biochim. Biophys. Acta 1851, 30–39. 10.1016/j.bbalip.2014.08.005 25139561

[B60] Galle M. Kladniew B. R. Castro M. A. Villegas S. M. Lacunza E. Polo M. (2015). Modulation by Geraniol of Gene Expression Involved in Lipid Metabolism Leading to a Reduction of Serum-Cholesterol and Triglyceride Levels. Phytomedicine 22, 696–704. 10.1016/j.phymed.2015.04.005 26141755

[B61] Gao X. Fu T. Wang C. Ning C. Kong Y. Liu Z. (2017). Computational Discovery and Experimental Verification of Farnesoid X Receptor Agonist Auraptene to Protect against Cholestatic Liver Injury. Biochem. Pharmacol. 146, 127–138. 10.1016/j.bcp.2017.09.016 28987596

[B62] Gao X. Wang C. Ning C. Liu K. Wang X. Liu Z. (2018). Hepatoprotection of Auraptene from Peels of Citrus Fruits against Thioacetamide-Induced Hepatic Fibrosis in Mice by Activating Farnesoid X Receptor. Food Funct. 9, 2684–2694. 10.1039/c8fo00107c 29721568

[B63] Ge C. X. Yu R. Xu M. X. Li P. Q. Fan C. Y. Li J. M. (2016). Betaine Prevented Fructose-Induced NAFLD by Regulating LXRα/PPARα Pathway and Alleviating ER Stress in Rats. Eur. J. Pharmacol. 770, 154–164. 10.1016/j.ejphar.2015.11.043 26593707

[B64] Ge Z. Zhang M. Deng X. Zhu W. Li K. Li C. (2017). Persimmon Tannin Promoted Macrophage Reverse Cholesterol Transport through Inhibiting ERK1/2 and Activating PPARγ Both *In Vitro* and *In Vivo* . J. Funct. Foods 38, 338–348. 10.1016/j.jff.2017.09.023

[B65] Genet C. Strehle A. Schmidt C. Boudjelal G. Lobstein A. Schoonjans K. (2010). Structure-activity Relationship Study of Betulinic Acid, a Novel and Selective TGR5 Agonist, and its Synthetic Derivatives: Potential Impact in Diabetes. J. Med. Chem. 53, 178–190. 10.1021/jm900872z 19911773

[B66] Goldwasser J. Cohen P. Y. Yang E. Balaguer P. Yarmush M. L. Nahmias Y. (2010). Transcriptional Regulation of Human and Rat Hepatic Lipid Metabolism by the Grapefruit Flavonoid Naringenin: Role of PPARα, PPARγ and LXRα. Plos One 5, e12399. 10.1371/journal.pone.0012399 20811644 PMC2928300

[B67] González-Granillo M. Steffensen K. R. Granados O. Torres N. Korach-André M. Ortíz V. (2012). Soy Protein Isoflavones Differentially Regulate Liver X Receptor Isoforms to Modulate Lipid Metabolism and Cholesterol Transport in the Liver and Intestine in Mice. Diabetologia 55, 2469–2478. 10.1007/s00125-012-2599-9 22739758

[B68] Goodwin B. J. Zuercher W. J. Collins J. L. (2008). Recent Advances in Liver X Receptor Biology and Chemistry. Curr. Top. Med. Chem. 8, 781–791. 10.2174/156802608784535075 18537688

[B69] Goto T. Kim Y. I. Funakoshi K. Teraminami A. Uemura T. Hirai S. (2011). Farnesol, an Isoprenoid, Improves Metabolic Abnormalities in Mice via Both PPARα-dependent and -independent Pathways. Am. J. Physiol. Endocrinol. Metab. 301, E1022–E1032. 10.1152/ajpendo.00061.2011 21862726

[B70] Grienke U. Mihály-Bison J. Schuster D. Afonyushkin T. Binder M. Guan S. H. (2011). Pharmacophore-based Discovery of FXR-Agonists. Part II: Identification of Bioactive Triterpenes from Ganoderma Lucidum. Bioorg. Med. Chem. 19, 6779–6791. 10.1016/j.bmc.2011.09.039 22014750 PMC3254236

[B71] Gronemeyer H. Gustafsson J. A. Laudet V. (2004). Principles for Modulation of the Nuclear Receptor Superfamily. Nat. Rev. Drug Discov. 3, 950–964. 10.1038/nrd1551 15520817

[B72] Gu M. Zhang S. Zhao Y. Huang J. Wang Y. Li Y. (2017a). Cycloastragenol Improves Hepatic Steatosis by Activating Farnesoid X Receptor Signalling. Pharmacol. Res. 121, 22–32. 10.1016/j.phrs.2017.04.021 28428116

[B73] Gu M. Zhang Y. Liu C. Wang D. Feng L. Fan S. (2017b). Morin, a Novel Liver X Receptor α/β Dual Antagonist, Has Potent Therapeutic Efficacy for Nonalcoholic Fatty Liver Diseases. Br. J. Pharmacol. 174 (18), 3032–3044. 10.1111/bph.13933 28646531 PMC5573424

[B74] Hahn D. Kim H. Yang I. Chin J. Hwang H. Won D. H. (2016). The Halicylindramides, Farnesoid X Receptor Antagonizing Depsipeptides from a Petrosia Sp. Marine Sponge Collected in Korea. J. Nat. Prod. 79, 499–506. 10.1021/acs.jnatprod.5b00871 26821210

[B75] Han C. Y. Kim T. H. Koo J. H. Kim S. G. (2016). Farnesoid X Receptor as a Regulator of Fuel Consumption and Mitochondrial Function. Arch. Pharm. Res. 39, 1062–1074. 10.1007/s12272-016-0812-y 27515052

[B76] Han X. Song J. Lian L. H. Yao Y. L. Shao D. Y. Fan Y. (2018). Ginsenoside 25-OCH3-PPD Promotes Activity of LXRs to Ameliorate P2X7R-Mediated NLRP3 Inflammasome in the Development of Hepatic Fibrosis. J. Agric. Food Chem. 66, 7023–7035. 10.1021/acs.jafc.8b01982 29929367

[B77] Hannan M. A. Sohag A. A. M. Dash R. Haque M. N. Mohibbullah M. Oktaviani D. F. (2020). Phytosterols of marine Algae: Insights into the Potential Health Benefits and Molecular Pharmacology. Phytomedicine 69, 153201. 10.1016/j.phymed.2020.153201 32276177

[B78] Harada K. Makino K. Shima N. Okuyama H. Esumi T. Kubo M. (2013). Total Synthesis of Riccardin C and (±)-cavicularin via Pd-Catalyzed Ar-Ar Cross Couplings. Tetrahedron 69, 6959–6968. 10.1016/j.tet.2013.06.064

[B79] He Y. Q. Ma G. Y. Peng J. N. Ma Z. Y. Hamann M. T. (2012). Liver X Receptor and Peroxisome Proliferator-Activated Receptor Agonist from Cornus Alternifolia. Biochim. Biophys. Acta 1820, 1021–1026. 10.1016/j.bbagen.2012.02.004 22353334 PMC3482494

[B80] Herath K. B. Jayasuriya H. Guan Z. Schulman M. Ruby C. Sharma N. (2005). Anthrabenzoxocinones from Streptomyces Sp. As Liver X Receptor Ligands and Antibacterial Agents. J. Nat. Prod. 68, 1437–1440. 10.1021/np050176k 16180833

[B81] Hiebl V. Ladurner A. Latkolik S. Dirsch V. M. (2018). Natural Products as Modulators of the Nuclear Receptors and Metabolic Sensors LXR, FXR and RXR. Biotechnol. Adv. 36, 1657–1698. 10.1016/j.biotechadv.2018.03.003 29548878

[B82] Hien H. T. M. Ha N. C. Thom L. T. Hong D. D. (2017). Squalene Promotes Cholesterol Homeostasis in Macrophage and Hepatocyte Cells via Activation of Liver X Receptor (LXR) α and β. Biotechnol. Lett. 39, 1101–1107. 10.1002/asna.20081115010.1007/s10529-017-2345-y 28492976

[B83] Hoang M. H. Jia Y. Jun H. J. Lee J. H. Hwang K. Y. Choi D. W. (2012b). Taurine Is a Liver X Receptor-α Ligand and Activates Transcription of Key Genes in the Reverse Cholesterol Transport without Inducing Hepatic Lipogenesis. Mol. Nutr. Food Res. 56, 900–911. 10.1002/mnfr.201100611 22707265

[B84] Hoang M. H. Jia Y. Jun H. J. Lee J. H. Lee B. Y. Lee S. J. (2012c). Fucosterol Is a Selective Liver X Receptor Modulator that Regulates the Expression of Key Genes in Cholesterol Homeostasis in Macrophages, Hepatocytes, and Intestinal Cells. J. Agric. Food Chem. 60, 11567–11575. 10.1021/jf3019084 23116181

[B85] Hoang M. H. Jia Y. Jun H. J. Lee J. H. Lee D. H. Hwang B. Y. (2012a). Ethyl 2,4,6-trihydroxybenzoate Is an Agonistic Ligand for Liver X Receptor that Induces Cholesterol Efflux from Macrophages without Affecting Lipid Accumulation in HepG2 Cells. Bioorg. Med. Chem. Lett. 22, 4094–4099. 10.1016/j.bmcl.2012.04.071 22579484

[B86] Hoang M. H. Jia Y. Mok B. Jun H. J. Hwang K. Y. Lee S. J. (2015). Kaempferol Ameliorates Symptoms of Metabolic Syndrome by Regulating Activities of Liver X Receptor-β. J. Nutr. Biochem. 26, 868–875. 10.1016/j.jnutbio.2015.03.005 25959373

[B87] Hollman D. A. Milona A. van Erpecum K. J. van Mil S. W. (2012). Anti-inflammatory and Metabolic Actions of FXR: Insights into Molecular Mechanisms. Biochim. Biophys. Acta 1821, 1443–1452. 10.1016/j.bbalip.2012.07.004 22820415

[B88] Hong C. Tontonoz P. (2008). Coordination of Inflammation and Metabolism by PPAR and LXR Nuclear Receptors. Curr. Opin. Genet. Dev. 18, 461–467. 10.1016/j.gde.2008.07.016 18782619 PMC2641014

[B89] Hong C. Tontonoz P. (2014). Liver X Receptors in Lipid Metabolism: Opportunities for Drug Discovery. Nat. Rev. Drug Discov. 13, 433–444. 10.1038/nrd4280 24833295

[B90] Hsieh Y. H. Wang S. Y. (2013). Lucidone from Lindera Erythrocarpa Makino Fruits Suppresses Adipogenesis in 3T3-L1 Cells and Attenuates Obesity and Consequent Metabolic Disorders in High-Fat Diet C57BL/6 Mice. Phytomedicine 20, 394–400. 10.1016/j.phymed.2012.11.007 23265843

[B91] Hsu W. H. Chen T. H. Lee B. H. Hsu Y. W. Pan T. M. (2014). Monascin and Ankaflavin Act as Natural AMPK Activators with PPARα Agonist Activity to Down-Regulate Nonalcoholic Steatohepatitis in High-Fat Diet-Fed C57BL/6 Mice. Food Chem. Toxicol. 64, 94–103. 10.1016/j.fct.2013.11.015 24275089

[B92] Hu X. Zhang N. Fu Y. (2018). Role of Liver X Receptor in Mastitis Therapy and Regulation of Milk Fat Synthesis. J. Mammary Gland Biol. Neoplasia 24, 73–83. 10.1007/s10911-018-9403-5 30066175

[B93] Huang C. (2014). Natural Modulators of Liver X Receptors. J. Integr. Med. 12, 76–85. 10.1016/S2095-4964(14)60013-3 24666673

[B94] Huang H. Xu Y. Zhu J. Li J. (2014). Recent Advances in Non-steroidal FXR Antagonists Development for Therapeutic Applications. Curr. Top. Med. Chem. 14, 2175–2187. 10.2174/1568026614666141112101840 25388534

[B95] Huo X. K. Liu J. Yu Z. L. Wang Y. F. Wang C. Tian X. G. (2018). Alisma Orientale Extract Exerts the Reversing Cholestasis Effect by Activation of Farnesoid X Receptor. Phytomedicine 42, 34–42. 10.1016/j.phymed.2018.03.017 29655695

[B96] Hwang Y. P. Choi J. H. Kim H. G. Khanal T. Song G. Y. Nam M. S. (2013). Saponins, Especially Platycodin D, from Platycodon Grandiflorum Modulate Hepatic Lipogenesis in High-Fat Diet-Fed Rats and High Glucose-Exposed HepG2 Cells. Toxicol. Appl. Pharmacol. 267, 174–183. 10.1016/j.taap.2013.01.001 23319015

[B97] Iio A. Ohguchi K. Iinuma M. Nozawa Y. Ito M. (2012a). Hesperetin Upregulates ABCA1 Expression and Promotes Cholesterol Efflux from THP-1 Macrophages. J. Nat. Prod. 75 (4), 563–566. 10.1021/np200696r 22429094

[B98] Iio A. Ohguchi K. Maruyama H. Tazawa S. Araki Y. Ichihara K. (2012b). Ethanolic Extracts of Brazilian Red Propolis Increase ABCA1 Expression and Promote Cholesterol Efflux from THP-1 Macrophages. Phytomedicine 19, 383–388. 10.1016/j.phymed.2011.10.007 22305277

[B99] Inagaki T. Choi M. Moschetta A. Peng L. Cummins C. L. McDonald J. G. (2005). Fibroblast Growth Factor 15 Functions as an Enterohepatic Signal to Regulate Bile Acid Homeostasis. Cell Metab 2, 217–225. 10.1016/j.cmet.2005.09.001 16213224

[B100] Inoue M. Tanabe H. Nakashima K. Ishida Y. Kotani H. (2014). Rexinoids Isolated from Sophora Tonkinensis with a Gene Expression Profile Distinct from the Synthetic Rexinoid Bexarotene. J. Nat. Prod. 77, 1670–1677. 10.1021/np5002016 24959987

[B101] Ishibashi M. Filomenko R. Rébé C. Chevriaux A. Varin A. Derangère V. (2013). Knock-down of the Oxysterol Receptor LXRα Impairs Cholesterol Efflux in Human Primary Macrophages: Lack of Compensation by LXRβ Activation. Biochem. Pharmacol. 86, 122–129. 10.1016/j.bcp.2012.12.024 23313547

[B102] Jakobsson T. Treuter E. Gustafsson J. Å. Steffensen K. R. (2012). Liver X Receptor Biology and Pharmacology: New Pathways, Challenges and Opportunities. Trends Pharmacol. Sci. 33, 394–404. 10.1016/j.tips.2012.03.013 22541735

[B103] Jang Y. J. Son H. J. Ahn J. Jung C. H. Ha T. (2016). Coumestrol Modulates Akt and Wnt/β-Catenin Signaling during the Attenuation of Adipogenesis. Food Funct. 7, 4984–4991. 10.1039/c6fo01127f 27868125

[B104] Janowski B. A. Willy P. J. Devi T. R. Falck J. R. Mangelsdorf D. J. (1996). An Oxysterol Signalling Pathway Mediated by the Nuclear Receptor LXR Alpha. Nature 383, 728–731. 10.1038/383728a0 8878485

[B105] Jayasuriya H. Herath K. B. Ondeyka J. G. Guan Z. Borris R. P. Tiwari S. (2005). Diterpenoid, Steroid, and Triterpenoid Agonists of Liver X Receptors from Diversified Terrestrial Plants and marine Sources. J. Nat. Prod. 68, 1247–1252. 10.1002/ibd.2045710.1021/np050182g 16124770

[B106] Jeong S. J. Park J. G. Kim S. Kweon H. Y. Seo S. Na D. S. (2015). Extract of Rhus Verniciflua Stokes Protects the Diet-Induced Hyperlipidemia in Mice. Arch. Pharm. Res. 38, 2049–2058. 10.1007/s12272-015-0579-6 25784057

[B107] Ji W. Gong B. Q. (2008). Hypolipidemic Activity and Mechanism of Purified Herbal Extract of Salvia Miltiorrhiza in Hyperlipidemic Rats. J. Ethnopharmacol. 119, 291–298. 10.1016/j.jep.2008.07.013 18691646

[B108] Ji W. Gong B. Q. (2007). Hypolipidemic Effects and Mechanisms of Panax Notoginseng on Lipid Profile in Hyperlipidemic Rats. J. Ethnopharmacol. 113, 318–324. 10.1016/j.jep.2007.06.022 17681443

[B109] Jia Y. Hoang M. H. Jun H. J. Lee J. H. Lee S. J. (2013). Cyanidin, a Natural Flavonoid, Is an Agonistic Ligand for Liver X Receptor Alpha and Beta and Reduces Cellular Lipid Accumulation in Macrophages and Hepatocytes. Bioorg. Med. Chem. Lett. 23, 4185–4190. 10.1016/j.bmcl.2013.05.030 23769638

[B110] Jia Y. Li Z. Y. Zhang H. G. Li H. B. Liu Y. Li X. H. (2010). Panax Notoginseng Saponins Decrease Cholesterol Ester via Up-Regulating ATP-Binding Cassette Transporter A1 in Foam Cells. J. Ethnopharmacol. 132, 297–302. 10.1016/j.jep.2010.08.033 20727959

[B111] Jiang T. Ren K. Chen Q. Li H. Yao R. Hu H. (2017). Leonurine Prevents Atherosclerosis via Promoting the Expression of ABCA1 and ABCG1 in a Pparγ/Lxrα Signaling Pathway-dependent Manner. Cell Physiol. Biochem. 43, 1703–1717. 10.1159/000484031 29045950

[B112] Jiang Z. Huang X. Huang S. Guo H. Wang L. Li X. (2016). Sex-Related Differences of Lipid Metabolism Induced by Triptolide: The Possible Role of the LXRα/SREBP-1 Signaling Pathway. Front. Pharmacol. 7, 87. 10.3389/fphar.2016.00087 27065871 PMC4814849

[B113] Jiang Z. P. Luan Z. L. Liu R. X. Zhang Q. Ma X. C. Shen L. (2018). Mangrove Tirucallane- and Apotirucallane-type Triterpenoids: Structure Diversity of the C-17 Side-Chain and Natural Agonists of Human Farnesoid/Pregnane⁻X⁻Receptor. Mar. Drugs 16, 488. 10.3390/md16120488 PMC6315889 30563240

[B114] Jones W. P. Chin Y. W. Kinghorn A. D. (2006). The Role of Pharmacognosy in Modern Medicine and Pharmacy. Curr. Drug Targets 7, 247–264. 10.2174/138945006776054915 16515526

[B115] Jun H. J. Hoang M. H. Lee J. W. Yaoyao J. Lee J. H. Lee D. H. (2012). Iristectorigenin B Isolated from Belamcanda Chinensis Is a Liver X Receptor Modulator that Increases ABCA1 and ABCG1 Expression in Macrophage RAW 264.7 Cells. Biotechnol. Lett. 34, 2213–2221. 10.1007/s10529-012-1036-y 23011313

[B116] Jun H. J. Hoang M. H. Yeo S. K. Jia Y. Lee S. J. (2013). Induction of ABCA1 and ABCG1 Expression by the Liver X Receptor Modulator Cineole in Macrophages. Bioorg. Med. Chem. Lett. 23, 579–583. 10.1016/j.bmcl.2012.11.012 23246324

[B117] Jung C. G. Horike H. Cha B. Y. Uhm K. O. Yamauchi R. Yamaguchi T. (2010). Honokiol Increases ABCA1 Expression Level by Activating Retinoid X Receptor Beta. Biol. Pharm. Bull. 33, 1105–1111. 10.1248/bpb.33.1105 20606297

[B118] Kandanur S. G. S. Tamang N. Golakoti N. R. Nanduri S. (2019). Andrographolide: A Natural Product Template for the Generation of Structurally and Biologically Diverse Diterpenes. Eur. J. Med. Chem. 176, 513–533. 10.1016/j.ejmech.2019.05.022 31151068

[B119] Kawase A. Yamada A. Gamou Y. Tahara C. Takeshita F. Murata K. (2012). Increased Effects of Ginsenosides on the Expression of Cholesterol 7α-Hydroxylase but Not the Bile Salt export Pump Are Involved in Cholesterol Metabolism. J. Nat. Med. 67, 545–553. 10.1007/s11418-012-0713-4 23108811

[B120] Kim M. H. Kang K. S. Lee Y. S. (2010a). The Inhibitory Effect of Genistein on Hepatic Steatosis Is Linked to Visceral Adipocyte Metabolism in Mice with Diet-Induced Non-alcoholic Fatty Liver Disease. Br. J. Nutr. 104, 1333–1342. 10.1017/s0007114510002266 20687969

[B121] Kim S. Yoon Y. Y. Park Y. W. Whang W. K. Park S. Y. Hwang K. W. (2020). Cynandione A from Cynanchum Wilfordii Inhibits Hepatic De Novo Lipogenesis by Activating the LKB1/AMPK Pathway in HepG2 Cells. J. Nat. Med. 74, 142–152. 10.1007/s11418-019-01356-x 31463669

[B122] Kim Y. M. Kim T. H. Kim Y. W. Yang Y. M. Ryu D. H. Hwang S. J. (2010b). Inhibition of Liver X Receptor-α-dependent Hepatic Steatosis by Isoliquiritigenin, a Licorice Antioxidant Flavonoid, as Mediated by JNK1 Inhibition. Free Radic. Biol. Med. 49, 1722–1734. 10.1016/j.freeradbiomed.2010.09.001 20840863

[B123] Kim Y. W. Kim Y. M. Yang Y. M. Kim T. H. Hwang S. J. Lee J. R. (2010c). Inhibition of SREBP-1c-Mediated Hepatic Steatosis and Oxidative Stress by Sauchinone, an AMPK-Activating Lignan in Saururus Chinensis. Free Radic. Biol. Med. 48, 567–578. 10.1016/j.freeradbiomed.2009.12.006 20005944

[B124] Koehn F. E. Carter G. T. (2005). The Evolving Role of Natural Products in Drug Discovery. Nat. Rev. Drug Discov. 4, 206–220. 10.1038/nrd1657 15729362

[B125] Komati R. Spadoni D. Zheng S. Sridhar J. Riley K. E. Wang G. (2017). Ligands of Therapeutic Utility for the Liver X Receptors. Molecules 22, 88. 10.3390/molecules22010088 PMC5373669 28067791

[B126] Kuang Y. L. Paulson K. E. Lichtenstein A. H. Lamon-Fava S. (2012). Regulation of the Expression of Key Genes Involved in HDL Metabolism by Unsaturated Fatty Acids. Br. J. Nutr. 108, 1351–1359. 10.1017/s0007114511006854 22221450

[B127] Kumar G. Singh D. Tali J. A. Dheer D. Shankar R. (2020). Andrographolide: Chemical Modification and its Effect on Biological Activities. Bioorg. Chem. 95, 103511. 10.1016/j.bioorg.2019.103511 31884143

[B128] Ky P. T. Huong P. T. My T. K. Anh P. T. Kiem P. V. Minh C. V. (2010). Dammarane-type Saponins from Gynostemma Pentaphyllum. Phytochemistry 71, 994–1001. 10.1016/j.phytochem.2010.03.009 20382401

[B129] Lambert G. Amar M. J. Guo G. Brewer H. B. Gonzalez F. J. Sinal C. J. (2003). The Farnesoid X-Receptor Is an Essential Regulator of Cholesterol Homeostasis. J. Biol. Chem. 278, 2563–2570. 10.1074/jbc.M209525200 12421815

[B130] Lee J. Jung E. Lee J. Kim S. Huh S. Kim Y. (2009). Isorhamnetin Represses Adipogenesis in 3T3-L1 Cells. Obesity (Silver Spring) 17, 226–232. 10.1038/oby.2008.472 18948972

[B131] Lee J. M. Gang G. T. Kim D. K. Kim Y. D. Koo S. H. Lee C. H. (2014). Ursodeoxycholic Acid Inhibits Liver X Receptor α-mediated Hepatic Lipogenesis via Induction of the Nuclear Corepressor SMILE. J. Biol. Chem. 289, 1079–1091. 10.1074/jbc.M113.491522 24265317 PMC3887176

[B132] Lee S. M. Moon J. Cho Y. Chung J. H. Shin M. J. (2013). Quercetin Up-Regulates Expressions of Peroxisome Proliferator-Activated Receptor γ, Liver X Receptor α, and ATP Binding Cassette Transporter A1 Genes and Increases Cholesterol Efflux in Human Macrophage Cell Line. Nutr. Res. 33, 136–143. 10.1016/j.nutres.2012.11.010 23399664

[B133] Lefebvre P. Cariou B. Lien F. Kuipers F. Staels B. (2009). Role of Bile Acids and Bile Acid Receptors in Metabolic Regulation. Physiol. Rev. 89, 147–191. 10.1152/physrev.00010.2008 19126757

[B134] Lehmann J. M. Kliewer S. A. Moore L. B. Smith-Oliver T. A. Oliver B. B. Su J. L. (1997). Activation of the Nuclear Receptor Lxr by Oxysterols Defines a New Hormone Response Pathway. J. Biol. Chem. 272, 3137–3140. 10.1074/jbc.272.6.3137 9013544

[B135] Lei Y. Fu P. Jun X. Cheng P. (2019). Pharmacological Properties of Geraniol – A Review. Planta. Med. 85, 48–55. 10.1055/a-0750-6907 30308694

[B136] Li C. Li Y. Gai Z. (2019). Bile Acids and Farnesoid X Receptor: Novel Target for the Treatment of Diabetic Cardiomyopathy. Curr. Protein Pept. Sci. 20, 976–983. 10.2174/1389203720666190726152847 31362653

[B137] Li C. H. Gong D. Chen L. Y. Zhang M. Xia X. D. Cheng H. P. (2017). Puerarin Promotes ABCA1-Mediated Cholesterol Efflux and Decreases Cellular Lipid Accumulation in THP-1 Macrophages. Eur. J. Pharmacol. 811, 74–86. 10.1016/j.ejphar.2017.05.055 28576406

[B138] Li C. J. Chen P. N. Li H. J. Mahmud T. Wu D. L. Xu J. (2020a). Potential Antidiabetic Fumiquinazoline Alkaloids from the marine-derived Fungus Scedosporium Apiospermum F41-1. J. Nat. Prod. 83, 1082–1091. 10.1021/acs.jnatprod.9b01096 32130008

[B139] Li G. Lin W. Araya J. J. Chen T. Timmermann B. N. Guo G. L. (2012). A tea Catechin, Epigallocatechin-3-Gallate, Is a Unique Modulator of the Farnesoid X Receptor. Toxicol. Appl. Pharmacol. 258, 268–274. 10.1016/j.taap.2011.11.006 22178739 PMC3259191

[B140] Li L. Bonneton F. Chen X. Y. Laudet V. (2015). Botanical Compounds and Their Regulation of Nuclear Receptor Action: the Case of Traditional Chinese Medicine. Mol. Cel Endocrinol. 401, 221–237. 10.1016/j.mce.2014.10.028 25449417

[B141] Li T. Hu S. M. Pang X. Y. Wang J. F. Yin J. Y. Li F. H. (2020b). The marine-derived Furanone Reduces Intracellular Lipid Accumulation *In Vitro* by Targeting LXRα and PPARα. J. Cel Mol. Med. 24, 3384–3398. 10.1111/jcmm.15012 PMC7131916 31981312

[B142] Li T. Xu L. Zheng R. Wang X. Li L. Ji H. (2020c). Picroside II Protects against Cholestatic Liver Injury Possibly through Activation of Farnesoid X Receptor. Phytomedicine 68, 153153. 10.1016/j.phymed.2019.153153 32018210

[B143] Li T. Yin J. Ji Y. Lin P. Li Y. Yang Z. (2020d). Setosphapyrone C and D Accelerate Macrophages Cholesterol Efflux by Promoting LXRα/ABCA1 Pathway. Arch. Pharm. Res. 43, 788–797. 10.1007/s12272-020-01255-w 32779151

[B144] Li T. Zheng R. Xu L. Zhou M. Wang X. Guo Q. (2020e). Picroside II Alleviates Liver Injury Induced by Alpha-Naphthylisothiocyanate through AMPK-FXR Pathway. Toxicol. Appl. Pharmacol. 408, 115248. 10.1016/j.taap.2020.115248 32976922

[B145] Liang X. Zhang X. Lu X. Zheng Z. Ma X. Qi S. (2019). Diketopiperazine-Type Alkaloids from a Deep-Sea-Derived Aspergillus puniceus Fungus and Their Effects on Liver X Receptor α. J. Nat. Prod. 82, 1558–1564. 10.1021/acs.jnatprod.9b00055 31095389

[B146] Liang Z. Gu T. Wang J. She J. Ye Y. Cao W. (2021). Chromene and Chromone Derivatives as Liver X Receptors Modulators from a marine-derived Pestalotiopsis Neglecta Fungus. Bioorg. Chem. 112, 104927. 10.1016/j.bioorg.2021.104927 33932772

[B147] Liang Z. Chen Y. Gu T. She J. Dai F. Jiang H. (2021). LXR-mediated Regulation of Marine-Derived Piericidins Aggravates High-Cholesterol Diet-Induced Cholesterol Metabolism Disorder in Mice. J. Med. Chem. 64, 9943–9959. 10.1021/acs.jmedchem.1c00175 34251816

[B148] Liao G. F. Wu Z. H. Liu Y. Yan Y. M. Lu R. M. Cheng Y. X. (2019). Ganocapenoids A-D: Four New Aromatic Meroterpenoids from Ganoderma Capense. Bioorg. Med. Chem. Lett. 29, 143–147. 10.1016/j.bmcl.2018.12.011 30527867

[B149] Lin H.-R. (2013a). Identification of Liver X Receptor and Farnesoid X Receptor Dual Agonists from Tithonia Diversifolia. Med. Chem. Res. 22, 3270–3281. 10.1007/s00044-012-0359-5

[B150] Lin H. R. Chou T. H. Huang D. W. Chen I. S. (2014). Cryptochinones from Cryptocarya Chinensis Act as Farnesoid X Receptor Agonists. Bioorg. Med. Chem. Lett. 24, 4181–4186. 10.1016/j.bmcl.2014.07.045 25127166

[B151] Lin H. R. (2015). Lepidozenolide from the Liverwort Lepidozia Fauriana Acts as a Farnesoid X Receptor Agonist. J. Asian Nat. Prod. Res. 17, 149–158. 10.1080/10286020.2014.964689 25315435

[B152] Lin H. R. (2013b). Paeoniflorin Acts as a Liver X Receptor Agonist. J. Asian Nat. Prod. Res. 15, 35–45. 10.1080/10286020.2012.742510 23281636

[B153] Lin H. R. (2012). Triterpenes from Alisma Orientalis Act as Farnesoid X Receptor Agonists. Bioorg. Med. Chem. Lett. 22, 4787–4792. 10.1016/j.bmcl.2012.05.057 22683342

[B154] Lin Y. Ren N. Li S. Chen M. Pu P. (2019). Novel Anti-obesity Effect of Scutellarein and Potential Underlying Mechanism of Actions. Biomed. Pharmacother. 117, 109042. 10.1016/j.biopha.2019.109042 31228804

[B155] Lin Y. N. Wang C. C. N. Chang H. Y. Chu F. Y. Hsu Y. A. Cheng W. K. (2018). Ursolic Acid, a Novel Liver X Receptor α (LXRα) Antagonist Inhibiting Ligand-Induced Nonalcoholic Fatty Liver and Drug-Induced Lipogenesis. J. Agric. Food Chem. 66, 11647–11662. 10.1021/acs.jafc.8b04116 30359008

[B156] Ling L. L. Schneider T. Peoples A. J. Spoering A. L. Engels I. Conlon B. P. (2015). Erratum: A New Antibiotic Kills Pathogens without Detectable Resistance. Nature 520, 388–459. 10.1038/nature1409810.1038/nature14303 25731174

[B157] Liu J. Li Y. Shi H. Wang T. Wu X. Sun X. (2016). Components Characterization of Total Tetraploid Jiaogulan ( Gynostemma Pentaphyllum ) Saponin and its Cholesterol-Lowering Properties. J. Funct. Foods 23, 542–555. 10.1016/j.jff.2016.03.013

[B158] Liu M. Zhang G. Song M. Wang J. Shen C. Chen Z. (2020a). Activation of Farnesoid X Receptor by Schaftoside Ameliorates Acetaminophen-Induced Hepatotoxicity by Modulating Oxidative Stress and Inflammation. Antioxid. Redox Signal. 33, 87–116. 10.1089/ars.2019.7791 32037847

[B159] Liu M. Zhang G. Wu S. Song M. Wang J. Cai W. (2020b). Schaftoside Alleviates HFD-Induced Hepatic Lipid Accumulation in Mice via Upregulating Farnesoid X Receptor. J. Ethnopharmacol. 255, 112776. 10.1016/j.jep.2020.112776 32205261

[B160] Liu W. Wong C. (2010). Oleanolic Acid Is a Selective Farnesoid X Receptor Modulator. Phytother. Res. 24, 369–373. 10.1002/ptr.2948 19653193

[B161] Liu X. X. Zhang X. W. Wang K. Wang X. Y. Ma W. L. Cao W. (2018). Kuwanon G Attenuates Atherosclerosis by Upregulation of LXRα-Abca1/abcg1 and Inhibition of NFκB Activity in Macrophages. Toxicol. Appl. Pharmacol. 341, 56–63. 10.1016/j.taap.2018.01.007 29355567

[B162] Liu Z. Law W.-K. Wang D. Nie X. Sheng D. Song G. (2014). Synthesis and Discovery of Andrographolide Derivatives as Non-steroidal Farnesoid X Receptor (FXR) Antagonists. RSC Adv. 4, 13533–13545. 10.1039/c3ra46715e

[B163] Lonard D. M. O’Malley B. W. (2007). Nuclear Receptor Coregulators: Judges, Juries, and Executioners of Cellular Regulation. Mol. Cel. 27, 691–700. 10.1016/j.molcel.2007.08.012 17803935

[B164] Lu T. T. Repa J. J. Mangelsdorf D. J. (2001). Orphan Nuclear Receptors as eLiXiRs and FiXeRs of Sterol Metabolism. J. Biol. Chem. 276, 37735–37738. 10.1074/jbc.R100035200 11459853

[B165] Lu X.-H. Shi Q.-W. Zheng Z.-H. Ke A.-B. Zhang H. Huo C.-H. (2011). Oxepinamides: Novel Liver X Receptor Agonists from Aspergillus puniceus. Eur. J. Org. Chem. 2011 (4), 802–807. 10.1002/ejoc.201000812

[B166] Lu Y. Xi W. Ding X. Fan S. Zhang Y. Jiang D. (2013). Citrange Fruit Extracts Alleviate Obesity-Associated Metabolic Disorder in High-Fat Diet-Induced Obese C57BL/6 Mouse. Int. J. Mol. Sci. 14, 23736–23750. 10.3390/ijms141223736 24317433 PMC3876074

[B167] Lu Y. Zheng W. Lin S. Guo F. Zhu Y. Wei Y. (2018). Identification of an Oleanane-type Triterpene Hedragonic Acid as a Novel Farnesoid X Receptor Ligand with Liver Protective Effects and Anti-inflammatory Activity. Mol. Pharmacol. 93, 63–72. 10.1124/mol.117.109900 29162643

[B168] Luan Z. L. Huo X. K. Dong P. P. Tian X. G. Sun C. P. Lv X. (2019). Highly Potent Non-steroidal FXR Agonists Protostane-type Triterpenoids: Structure-Activity Relationship and Mechanism. Eur. J. Med. Chem. 182, 111652. 10.1016/j.ejmech.2019.111652 31494470

[B169] Luo N. Fang J. Wei L. Sahebkar A. Little P. J. Xu S. (2021). Emodin in Atherosclerosis Prevention: Pharmacological Actions and Therapeutic Potential. Eur. J. Pharmacol. 890, 173617. 10.1016/j.ejphar.2020.173617 33010303

[B170] Luo X. Li C. Luo P. Lin X. Ma H. Seeram N. P. (2016). Pterosin Sesquiterpenoids from Pteris Cretica as Hypolipidemic Agents via Activating Liver X Receptors. J. Nat. Prod. 79, 3014–3021. 10.1021/acs.jnatprod.6b00558 28006909

[B171] Ma K. Li L. Bao L. He L. Sun C. Zhou B. (2015). Six New 3,4-Seco-27-Norlanostane Triterpenes from the Medicinal Mushroom Ganoderma Boninense and Their Antiplasmodial Activity and Agonistic Activity to LXRβ. Tetrahedron 71, 1808–1814. 10.1016/j.tet.2015.02.002

[B172] Ma X. Jiang Y. Wen J. Zhao Y. Zeng J. Guo Y. (2020). A Comprehensive Review of Natural Products to Fight Liver Fibrosis: Alkaloids, Terpenoids, Glycosides, Coumarins and Other Compounds. Eur. J. Pharmacol. 888, 173578. 10.1016/j.ejphar.2020.173578 32976828

[B173] Maithilikarpagaselvi N. Sridhar M. G. Swaminathan R. P. Sripradha R. Badhe B. (2016). Curcumin Inhibits Hyperlipidemia and Hepatic Fat Accumulation in High-Fructose-Fed Male Wistar Rats. Pharm. Biol. 54, 2857–2863. 10.1080/13880209.2016.1187179 27241764

[B174] Majdalawieh A. F. Dalibalta S. Yousef S. M. (2020). Effects of Sesamin on Fatty Acid and Cholesterol Metabolism, Macrophage Cholesterol Homeostasis and Serum Lipid Profile: A Comprehensive Review. Eur. J. Pharmacol. 885, 173417. 10.1016/j.ejphar.2020.173417 32750369

[B175] Majdalawieh A. F. Ro H. S. (2014). Sesamol and Sesame (Sesamum indicum) Oil Enhance Macrophage Cholesterol Efflux via Up-Regulation of PPARγ1 and LXRα Transcriptional Activity in a MAPK-dependent Manner. Eur. J. Nutr. 54, 691–700. 10.1007/s00394-014-0747-3 25081501

[B176] Masaoutis C. Theocharis S. (2018). The Farnesoid X Receptor: a Potential Target for Expanding the Therapeutic Arsenal against Kidney Disease. Expert Opin. Ther. Targets 23, 107–116. 10.1080/14728222.2019.1559825 30577722

[B177] McKenna N. J. Cooney A. J. DeMayo F. J. Downes M. Glass C. K. Lanz R. B. (2009). Minireview: Evolution of NURSA, the Nuclear Receptor Signaling Atlas. Mol. Endocrinol. 23, 740–746. 10.1210/me.2009-0135 19423650 PMC2691684

[B178] Meng Q. Chen X. Wang C. Liu Q. Sun H. Sun P. (2015). Protective Effects of Alisol B 23-acetate from Edible Botanical Rhizoma Alismatis against Carbon Tetrachloride-Induced Hepatotoxicity in Mice. Food Funct. 6, 1241–1250. 10.1039/c5fo00082c 25747392

[B179] Meng Q. Duan X. P. Wang C. Y. Liu Z. H. Sun P. Y. Huo X. K. (2017). Alisol B 23-acetate Protects against Non-alcoholic Steatohepatitis in Mice via Farnesoid X Receptor Activation. Acta Pharmacol. Sin. 38, 69–79. 10.1038/aps.2016.119 27773935 PMC5220543

[B180] Mi L. Z. Devarakonda S. Harp J. M. Han Q. Pellicciari R. Willson T. M. (2003). Structural Basis for Bile Acid Binding and Activation of the Nuclear Receptor FXR. Mol. Cel. 11, 1093–1100. 10.1016/S1097-2765(03)00112-6 12718893

[B181] Modica S. Gadaleta R. M. Moschetta A. (2010). Deciphering the Nuclear Bile Acid Receptor FXR Paradigm. Nucl. Recept. Signal. 8, e005. 10.1621/nrs.08005 21383957 PMC3049226

[B182] Nakashima K. I. Tomida J. Hirai T. Kawamura Y. Inoue M. (2019). Paraconiothins A-J: Sesquiterpenoids from the Endophytic Fungus Paraconiothyrium Brasiliense ECN258. J. Nat. Prod. 82, 3347–3356. 10.1021/acs.jnatprod.9b00638 31815465

[B183] Nam S. J. Ko H. Ju M. K. Hwang H. Chin J. Ham J. (2007). Scalarane Sesterterpenes from a marine Sponge of the Genus Spongia and Their Fxr Antagonistic Activity. J. Nat. Prod. 70, 1691–1695. 10.1021/np070024k 17988093

[B184] Nam S. J. Ko H. Shin M. Ham J. Chin J. Kim Y. (2006). Farnesoid X-Activated Receptor Antagonists from a marine Sponge Spongia Sp. Bioorg. Med. Chem. Lett. 16, 5398–5402. 10.1016/j.bmcl.2006.07.079 16905319

[B185] Neuschwander-Tetri B. A. Loomba R. Sanyal A. J. Lavine J. E. Van Natta M. L. Abdelmalek M. F. (2015). Farnesoid X Nuclear Receptor Ligand Obeticholic Acid for Non-cirrhotic, Non-alcoholic Steatohepatitis (FLINT): a Multicentre, Randomised, Placebo-Controlled Trial. Lancet 385, 956–965. 10.1016/s0140-6736(14)61933-4 25468160 PMC4447192

[B187] Niesor E. J. Flach J. Lopes-Antoni I. Perez A. Bentzen C. L. (2001). The Nuclear Receptors FXR and LXRalpha: Potential Targets for the Development of Drugs Affecting Lipid Metabolism and Neoplastic Diseases. Curr. Pharm. Des. 7, 231–259. 10.2174/1381612013398185 11254888

[B188] Ning Y. Xu F. Xin R. Yao F. (2020). Palmatine Regulates Bile Acid Cycle Metabolism and Maintains Intestinal flora Balance to Maintain Stable Intestinal Barrier. Life Sci. 262, 118405. 10.1016/j.lfs.2020.118405 32926925

[B189] Nozawa H. (2005). Xanthohumol, the Chalcone from Beer Hops (Humulus Lupulus L.), Is the Ligand for Farnesoid X Receptor and Ameliorates Lipid and Glucose Metabolism in KK-A(y) Mice. Biochem. Biophys. Res. Commun. 336, 754–761. 10.1016/j.bbrc.2005.08.159 16140264

[B190] Oh G. S. Lee G. G. Yoon J. Oh W. K. Kim S. W. (2015b). Selective Inhibition of Liver X Receptor α-mediated Lipogenesis in Primary Hepatocytes by Licochalcone A. Chin. Med. 10, 8. 10.1186/s13020-015-0037-x 25937827 PMC4416341

[B191] Oh G. S. Yoon J. Lee G. G. Oh W. K. Kim S. W. (2015a). 20(S)-protopanaxatriol Inhibits Liver X Receptor α-mediated Expression of Lipogenic Genes in Hepatocytes. J. Pharmacol. Sci. 128, 71–77. 10.1016/j.jphs.2015.05.007 26109499

[B192] Ohara K. Wakabayashi H. Taniguchi Y. Shindo K. Yajima H. Yoshida A. (2013). Quercetin-3-O-glucuronide Induces ABCA1 Expression by LXRα Activation in Murine Macrophages. Biochem. Biophys. Res. Commun. 441, 929–934. 10.1016/j.bbrc.2013.10.168 24216107

[B193] Ondeyka J. G. Jayasuriya H. Herath K. B. Guan Z. Schulman M. Collado J. (2005). Steroidal and Triterpenoidal Fungal Metabolites as Ligands of Liver X Receptors. J. Antibiot. (Tokyo) 58, 559–565. 10.1038/ja.2005.76 16320760

[B194] Ou J. Tu H. Shan B. Luk A. DeBose-Boyd R. A. Bashmakov Y. (2001). Unsaturated Fatty Acids Inhibit Transcription of the Sterol Regulatory Element-Binding Protein-1c (SREBP-1c) Gene by Antagonizing Ligand-dependent Activation of the LXR. Proc. Natl. Acad. Sci. U S A. 98, 6027–6032. 10.1073/pnas.111138698 11371634 PMC33416

[B195] Park H. S. Lee K. Kim S. H. Hong M. J. Jeong N. J. Kim M. S. (2020). Luteolin Improves Hypercholesterolemia and Glucose Intolerance through LXRα-dependent Pathway in Diet-Induced Obese Mice. J. Food Biochem. 44, e13358. 10.1111/jfbc.13358 32598492

[B196] Parks D. J. Blanchard S. G. Bledsoe R. K. Chandra G. Consler T. G. Kliewer S. A. (1999). Bile Acids: Natural Ligands for an Orphan Nuclear Receptor. Science 284, 1365–1368. 10.1126/science.284.5418.1365 10334993

[B197] Pattanayak S. Bose P. (2019). Herniarin, a Natural Coumarin, Inhibits Mammary Carcinogenesis by Modulating Liver X Receptor-Α/β-Pi3k-Akt-Maf1 Pathway in prague-dawley Rats. Phcog Mag. 15, 510. 10.4103/pm.pm_264_19

[B198] Periasamy S. Hsu D. Z. Chandrasekaran V. R. Liu M. Y. (2013). Sesame Oil Accelerates Healing of 2,4,6-trinitrobenzenesulfonic Acid-Induced Acute Colitis by Attenuating Inflammation and Fibrosis. JPEN J. Parenter. Enteral Nutr. 37, 674–682. 10.1177/0148607112468768 23243149

[B199] Pisonero-Vaquero S. García-Mediavilla M. V. Jorquera F. Majano P. L. Benet M. Jover R. (2014). Modulation of PI3K-lxrα-dependent Lipogenesis Mediated by Oxidative/nitrosative Stress Contributes to Inhibition of HCV Replication by Quercetin. Lab. Invest. 94, 262–274. 10.1038/labinvest.2013.156 24492281

[B200] Prakash P. Singh V. Jain M. Rana M. Khanna V. Barthwal M. K. (2014). Silymarin Ameliorates Fructose Induced Insulin Resistance Syndrome by Reducing De Novo Hepatic Lipogenesis in the Rat. Eur. J. Pharmacol. 727, 15–28. 10.1016/j.ejphar.2014.01.038 24486395

[B201] Pu S. Wu X. Yang X. Zhang Y. Dai Y. Zhang Y. (2019). The Therapeutic Role of Xenobiotic Nuclear Receptors against Metabolic Syndrome. Curr. Drug Metab. 20, 15–22. 10.2174/1389200219666180611083155 29886826

[B202] Qiu J. (2007). Traditional Chinese Medicine and Western Science Face Almost Irreconcilable differences.Can Systems Biology Bring Them Together? Nature 448, 126–128. 10.1038/448126a 17625539

[B203] Qu Y. Zhou L. Wang C. (2016). Effects of Platycodin D on IL-1β-induced Inflammatory Response in Human Osteoarthritis Chondrocytes. Int. Immunopharmacol. 40, 474–479. 10.1016/j.intimp.2016.09.025 27743553

[B204] Ren K. Jiang T. Zhao G. J. (2018). Quercetin Induces the Selective Uptake of HDL-Cholesterol via Promoting SR-BI Expression and the Activation of the PPARγ/LXRα Pathway. Food Funct. 9, 624–635. 10.1039/c7fo01107e 29292466

[B205] Renga B. Mencarelli A. D’Amore C. Cipriani S. D’Auria M. V. Sepe V. (2012). Discovery that Theonellasterol a marine Sponge Sterol Is a Highly Selective FXR Antagonist that Protects against Liver Injury in Cholestasis. Plos One 7, e30443. 10.1371/journal.pone.0030443 22291955 PMC3264597

[B206] Repa J. J. Turley S. D. Lobaccaro J. A. Medina J. Li L. Lustig K. (2000). Regulation of Absorption and ABC1-Mediated Efflux of Cholesterol by RXR Heterodimers. Science 289, 1524–1529. 10.1126/science.289.5484.1524 10968783

[B207] Ricketts M. L. Boekschoten M. V. Kreeft A. J. Hooiveld G. J. Moen C. J. Müller M. (2007). The Cholesterol-Raising Factor from Coffee Beans, Cafestol, as an Agonist Ligand for the Farnesoid and Pregnane X Receptors. Mol. Endocrinol. 21, 1603–1616. 10.1210/me.2007-0133 17456796

[B208] Rincón Y. A. Siless G. E. Amado L. D. Dansey M. V. Grassi E. Schenone N. (2020). Lanostanoid Triterpenes from the Fungus Rigidoporus Microporus. Nat. Prod. Res. 35, 1–10. 10.1080/14786419.2020.1752205 32308028

[B209] Russell D. W. (2003). The Enzymes, Regulation, and Genetics of Bile Acid Synthesis. Annu. Rev. Biochem. 72, 137–174. 10.1146/annurev.biochem.72.121801.161712 12543708

[B210] Saenz J. Santa-María C. Reyes-Quiroz M. E. Geniz I. Jiménez J. Sobrino F. (2018). Grapefruit Flavonoid Naringenin Regulates the Expression of LXRα in THP-1 Macrophages by Modulating AMP-Activated Protein Kinase. Mol. Pharm. 15, 1735–1745. 10.1021/acs.molpharmaceut.7b00797 29140707

[B308] Schroepfer G. J. (2000). Oxysterols: Modulators of Cholesterol Metabolism and Other Processes. Physiol. Rev. 80, 361–554. 10.1152/physrev.2000.80.1.361 10617772

[B211] Sepe V. Bifulco G. Renga B. D’Amore C. Fiorucci S. Zampella A. (2011). Discovery of Sulfated Sterols from marine Invertebrates as a New Class of marine Natural Antagonists of Farnesoid-X-Receptor. J. Med. Chem. 54, 1314–1320. 10.1021/jm101336m 21309576

[B212] Sepe V. D’Amore C. Ummarino R. Renga B. D’Auria M. V. Novellino E. (2014). Insights on Pregnane-X-Receptor Modulation. Natural and Semisynthetic Steroids from Theonella marine Sponges. Eur. J. Med. Chem. 73, 126–134. 10.1016/j.ejmech.2013.12.005 24388834

[B213] Sepe V. Ummarino R. D’Auria M. V. Chini M. G. Bifulco G. Renga B. (2012). Conicasterol E, a Small Heterodimer Partner Sparing Farnesoid X Receptor Modulator Endowed with a Pregnane X Receptor Agonistic Activity, from the marine Sponge Theonella Swinhoei. J. Med. Chem. 55, 84–93. 10.1021/jm201004p 22126372

[B214] Sevov M. Elfineh L. Cavelier L. B. (2006). Resveratrol Regulates the Expression of LXR-Alpha in Human Macrophages. Biochem. Biophys. Res. Commun. 348, 1047–1054. 10.1016/j.bbrc.2006.07.155 16901463

[B215] Shang S. Z. Kong L. M. Yang L. P. Jiang J. Huang J. Zhang H. B. (2013). Bioactive Phenolics and Terpenoids from Manglietia Insignis. Fitoterapia 84, 58–63. 10.1016/j.fitote.2012.10.010 23103294

[B216] Shen B. (2015). A New golden Age of Natural Products Drug Discovery. Cell 163, 1297–1300. 10.1016/j.cell.2015.11.031 26638061 PMC5070666

[B217] Sheng X. Wang M. Lu M. Xi B. Sheng H. Zang Y. Q. (2011a). Rhein Ameliorates Fatty Liver Disease through Negative Energy Balance, Hepatic Lipogenic Regulation, and Immunomodulation in Diet-Induced Obese Mice. Am. J. Physiol. Endocrinol. Metab. 300, E886–E893. 10.1152/ajpendo.00332.2010 21364120

[B218] Sheng X. Zhu X. Zhang Y. Cui G. Peng L. Lu X. (2011b). Rhein Protects against Obesity and Related Metabolic Disorders through Liver X Receptor-Mediated Uncoupling Protein 1 Upregulation in Brown Adipose Tissue. Int. J. Biol. Sci. 8, 1375–1384. 10.7150/ijbs.4575 PMC3492795 23139635

[B219] Shin K. Chin J. Hahn D. Lee J. Hwang H. Won D. H. (2012). Sterols from a Soft Coral, Dendronephthya Gigantea as Farnesoid X-Activated Receptor Antagonists. Steroids 77 (5), 355–359. 10.1016/j.steroids.2011.12.027 22266736

[B220] Shinar D. M. Endo N. Rutledge S. J. Vogel R. Rodan G. A. Schmidt A. (1994). Ner, a New Member of the Gene Family Encoding the Human Steroid Hormone Nuclear Receptor. Gene 147, 273–276. 10.1016/0378-1119(94)90080-9 7926814

[B221] Sim W. C. Park S. Lee K. Y. Je Y. T. Yin H. Q. Choi Y. J. (2014). LXR-α Antagonist Meso-Dihydroguaiaretic Acid Attenuates High-Fat Diet-Induced Nonalcoholic Fatty Liver. Biochem. Pharmacol. 90, 414–424. 10.1016/j.bcp.2014.06.013 24955981

[B222] Sinal C. J. Tohkin M. Miyata M. Ward J. M. Lambert G. Gonzalez F. J. (2000). Targeted Disruption of the Nuclear Receptor FXR/BAR Impairs Bile Acid and Lipid Homeostasis. Cell 102, 731–744. 10.1016/S0092-8674(00)00062-3 11030617

[B223] Singh S. B. Ondeyka J. G. Liu W. Chen S. Chen T. S. Li X. (2005). Discovery and Development of Dimeric Podocarpic Acid Leads as Potent Agonists of Liver X Receptor with HDL Cholesterol Raising Activity in Mice and Hamsters. Bioorg. Med. Chem. Lett. 15, 2824–2828. 10.1016/j.bmcl.2005.03.100 15911262

[B224] Singh S. P. Sashidhara K. V. (2017). Lipid Lowering Agents of Natural Origin: An Account of Some Promising Chemotypes. Eur. J. Med. Chem. 140, 331–348. 10.1016/j.ejmech.2017.09.020 28987600

[B225] Soisson S. M. Parthasarathy G. Adams A. D. Sahoo S. Sitlani A. Sparrow C. (2008). Identification of a Potent Synthetic Fxr Agonist with an Unexpected Mode of Binding and Activation. Proc. Natl. Acad. Sci. U S A. 105, 5337–5342. 10.1073/pnas.0710981105 18391212 PMC2291122

[B226] Song X. Y. Xu S. Hu J. F. Tang J. Chu S. F. Liu H. (2015). Piperine Prevents Cholesterol Gallstones Formation in Mice. Eur. J. Pharmacol. 751, 112–117. 10.1016/j.ejphar.2015.01.038 25645812

[B227] Steri R. Achenbach J. Steinhilber D. Schubert-Zsilavecz M. Proschak E. (2012). Investigation of Imatinib and Other Approved Drugs as Starting Points for Antidiabetic Drug Discovery with FXR Modulating Activity. Biochem. Pharmacol. 83, 1674–1681. 10.1016/j.bcp.2012.02.027 22414727

[B228] Strege M. A. (1999). High-performance Liquid Chromatographic-Electrospray Ionization Mass Spectrometric Analyses for the Integration of Natural Products with Modern High-Throughput Screening. J. Chromatogr. B. Biomed. Sci. Appl. 725, 67–78. 10.1016/S0378-4347(98)00553-2 10226878

[B229] Sun R. Yang N. Kong B. Cao B. Feng D. Yu X. (2017). Orally Administered Berberine Modulates Hepatic Lipid Metabolism by Altering Microbial Bile Acid Metabolism and the Intestinal FXR Signaling Pathway. Mol. Pharmacol. 91, 110–122. 10.1124/mol.116.106617 27932556 PMC5267522

[B230] Sutjarit N. Sueajai J. Boonmuen N. Sornkaew N. Suksamrarn A. Tuchinda P. (2017). Curcuma Comosa Reduces Visceral Adipose Tissue and Improves Dyslipidemia in Ovariectomized Rats. J. Ethnopharmacol. 215, 167–175. 10.1016/j.jep.2017.12.027 29273438

[B231] Suzuki T. Nishimaki-Mogami T. Kawai H. Kobayashi T. Shinozaki Y. Sato Y. (2006). Screening of Novel Nuclear Receptor Agonists by a Convenient Reporter Gene Assay System Using green Fluorescent Protein Derivatives. Phytomedicine 13, 401–411. 10.1016/j.phymed.2005.09.003 16716909

[B232] Suzuki T. Tamehiro N. Sato Y. Kobayashi T. Ishii-Watabe A. Shinozaki Y. (2008). The Novel Compounds that Activate Farnesoid X Receptor: the Diversity of Their Effects on Gene Expression. J. Pharmacol. Sci. 107, 285–294. 10.1254/jphs.08006fp 18603830

[B233] Svensson S. Ostberg T. Jacobsson M. Norström C. Stefansson K. Hallén D. (2014). Crystal Structure of the Heterodimeric Complex of LXRalpha and RXRbeta Ligand-Binding Domains in a Fully Agonistic Conformation. EMBO J. 22, 4625–4633. 10.1093/emboj/cdg456 PMC212723 12970175

[B234] Tai T. S. Tien N. Shen H. Y. Chu F. Y. Wang C. C. N. Lu C. H. (2019). Sesamin, a Naturally Occurring Lignan, Inhibits Ligand-Induced Lipogenesis through Interaction with Liver X Receptor Alpha (LXRα) and Pregnane X Receptor (PXR). Evid. Based. Complement. Alternat. Med. 2019, 9401648. 10.1155/2019/9401648 31976003 PMC6959160

[B235] Takahashi M. Kanayama T. Yashiro T. Kondo H. Murase T. Hase T. (2008). Effects of Coumestrol on Lipid and Glucose Metabolism as a Farnesoid X Receptor Ligand. Biochem. Biophys. Res. Commun. 372, 395–399. 10.1016/j.bbrc.2008.04.136 18457666

[B236] Talbi M. Saadali B. Boriky D. Bennani L. Elkouali M. Ainane T. (2016). Two Natural Compounds – a Benzofuran and a Phenylpropane – from Artemisia Dracunculus. J. Asian Nat. Prod. Res. 18, 724–729. 10.1080/10286020.2016.1158708 26982075

[B237] Tamehiro N. Sato Y. Suzuki T. Hashimoto T. Asakawa Y. Yokoyama S. (2005). Riccardin C: a Natural Product that Functions as a Liver X Receptor (LXR)alpha Agonist and an LXRbeta Antagonist. FEBS Lett. 579, 5299–5304. 10.1016/j.febslet.2005.08.054 16182288

[B238] Tarling E. J. Edwards P. A. (2011). ATP Binding Cassette Transporter G1 (ABCG1) Is an Intracellular Sterol Transporter. Proc. Natl. Acad. Sci. U S A. 108, 19719–19724. 10.1073/pnas.1113021108 22095132 PMC3241749

[B239] Tian Y. Cai J. Gui W. Nichols R. G. Koo I. Zhang J. (2019). Berberine Directly Affects the Gut Microbiota to Promote Intestinal Farnesoid X Receptor Activation. Drug Metab. Dispos. 47, 86–93. 10.1124/dmd.118.083691 30409838 PMC6323626

[B240] Toppo E. Sylvester Darvin S. Esakkimuthu S. Buvanesvaragurunathan K. Ajeesh Krishna T. P. Antony Caesar S. (2018). Curative Effect of Arjunolic Acid from Terminalia Arjuna in Non-alcoholic Fatty Liver Disease Models. Biomed. Pharmacother. 107, 979–988. 10.1016/j.biopha.2018.08.019 30257410

[B241] Traves P. G. Hortelano S. Zeini M. Chao T. H. Lam T. Neuteboom S. T. (2007). Selective Activation of Liver X Receptors by Acanthoic Acid-Related Diterpenes. Mol. Pharmacol. 71, 1545–1553. 10.1124/mol.106.031906 17329499

[B242] Tsai C. J. Liang J. W. Lin H. R. (2012). Sesquiterpenoids from Atractylodes Macrocephala Act as Farnesoid X Receptor and Progesterone Receptor Modulators. Bioorg. Med. Chem. Lett. 22, 2326–2329. 10.1016/j.bmcl.2012.01.048 22365756

[B243] Uemura T. Goto T. Kang M. S. Mizoguchi N. Hirai S. Lee J. Y. (2011). Diosgenin, the Main Aglycon of Fenugreek, Inhibits LXRα Activity in HepG2 Cells and Decreases Plasma and Hepatic Triglycerides in Obese Diabetic Mice. J. Nutr. 141, 17–23. 10.3945/jn.110.125591 21106928

[B244] Urizar N. L. Liverman A. B. Dodds D. T. Silva F. V. Ordentlich P. Yan Y. (2002). A Natural Product that Lowers Cholesterol as an Antagonist Ligand for FXR. Science 296, 1703–1706. 10.1126/science.1072891 11988537

[B245] Urizar N. L. Moore D. D. (2003). GUGULIPID: A Natural Cholesterol-Lowering Agent. Annu. Rev. Nutr. 23, 303–313. 10.1146/annurev.nutr.23.011702.073102 12626688

[B246] Vásquez Y. Zhao J. Khan S. I. Gupta M. P. Khan I. A. (2019). Constituents of Talisia Nervosa with Potential Utility against Metabolic Syndrome. Nat. Product. Commun. 14, 1934578X1901400–54. 10.1177/1934578X1901400114

[B247] Voloshyna I. Hai O. Littlefield M. J. Carsons S. Reiss A. B. (2013). Resveratrol Mediates Anti-atherogenic Effects on Cholesterol Flux in Human Macrophages and Endothelium via PPARγ and Adenosine. Eur. J. Pharmacol. 698, 299–309. 10.1016/j.ejphar.2012.08.024 23041272

[B248] Wang C. M. Yuan R. S. Zhuang W. Y. Sun J. H. Wu J. Y. Li H. (2016). Schisandra Polysaccharide Inhibits Hepatic Lipid Accumulation by Downregulating Expression of SREBPs in NAFLD Mice. Lipids Health Dis. 15, 195. 10.1186/s12944-016-0358-5 27852305 PMC5112637

[B249] Wang D. Hiebl V. Xu T. Ladurner A. Atanasov A. G. Heiss E. H. (2020a). Impact of Natural Products on the Cholesterol Transporter ABCA1. J. Ethnopharmacol. 249, 112444. 10.1016/j.jep.2019.112444 31805338

[B250] Wang D. Lao L. Pang X. Qiao Q. Pang L. Feng Z. (2018a). Asiatic Acid from Potentilla Chinensis Alleviates Non-alcoholic Fatty Liver by Regulating Endoplasmic Reticulum Stress and Lipid Metabolism. Int. Immunopharmacology 65, 256–267. 10.1016/j.intimp.2018.10.013 30340105

[B251] Wang D. Q. (2007). Regulation of Intestinal Cholesterol Absorption. Annu. Rev. Physiol. 69, 221–248. 10.1146/annurev.physiol.69.031905.160725 17002594

[B252] Wang F. Zhao C. Tian G. Wei X. Ma Z. Cui J. (2020b). Naringin Alleviates Atherosclerosis in ApoE-/- Mice by Regulating Cholesterol Metabolism Involved in Gut Microbiota Remodeling. J. Agric. Food Chem. 68, 12651–12660. 10.1021/acs.jafc.0c05800 33107729

[B253] Wang H. He Q. Wang G. Xu X. Hao H. (2018b). FXR Modulators for Enterohepatic and Metabolic Diseases. Expert Opin. Ther. Pat. 28, 765–782. 10.1080/13543776.2018.1527906 30259754

[B254] Wang H. Luo Y. Qiao T. Wu Z. Huang Z. (2018c). Luteolin Sensitizes the Antitumor Effect of Cisplatin in Drug-Resistant Ovarian Cancer via Induction of Apoptosis and Inhibition of Cell Migration and Invasion. J. Ovarian. Res. 11, 93. 10.1021/acs.jafc.0c0580010.1186/s13048-018-0468-y 30454003 PMC6241043

[B255] Wang J. Liang Z. Li K. Yang B. Liu Y. Fang W. (2020c). Ene-yne Hydroquinones from a Marine-derived Strain of the Fungus Pestalotiopsis Neglecta with Effects on Liver X Receptor Alpha. J. Nat. Prod. 83, 1258–1264. 10.1021/acs.jnatprod.0c00050 32283019

[B256] Wang L. Wu G. Wu F. Jiang N. Lin Y. (2017a). Geniposide Attenuates ANIT-Induced Cholestasis through Regulation of Transporters and Enzymes Involved in Bile Acids Homeostasis in Rats. J. Ethnopharmacol. 196, 178–185. 10.1016/j.jep.2016.12.022 27988401

[B257] Wang S. Zhang X. Liu M. Luan H. Ji Y. Guo P. (2015). Chrysin Inhibits Foam Cell Formation through Promoting Cholesterol Efflux from RAW264.7 Macrophages. Pharm. Biol. 53, 1481–1487. 10.3109/13880209.2014.986688 25857322

[B258] Wang W. Nakashima K. I. Hirai T. Inoue M. (2019). Correction to: Neuroprotective Effect of Naturally Occurring RXR Agonists Isolated from Sophora Tonkinensis Gagnep. On Amyloid-β-Induced Cytotoxicity in PC12 Cells. J. Nat. Med. 73, 683–684. 10.1007/s11418-018-1257-z10.1007/s11418-019-01305-8 30968309

[B259] Wang X. Yan J. Xu X. Duan C. Xie Z. Su Z. (2018d). Puerarin Prevents LPS-Induced Acute Lung Injury via Inhibiting Inflammatory Response. Microb. Pathog. 118, 170–176. 10.1016/j.micpath.2018.03.033 29571724

[B260] Wang Y. Zhang X. Wei Z. Wang J. Zhang Y. Shi M. (2017b). Platycodin D Suppressed LPS-Induced Inflammatory Response by Activating LXRα in LPS-Stimulated Primary Bovine Mammary Epithelial Cells. Eur. J. Pharmacol. 814, 138–143. 10.1016/j.ejphar.2017.07.037 28736281

[B261] Wei X. Fan X. Feng Z. Ma Y. Lan X. Chen M. (2020). Ethyl Acetate Extract of Herpetospermum Pedunculosum Alleviates α-naphthylisothiocyanate-induced Cholestasis by Activating the Farnesoid X Receptor and Suppressing Oxidative Stress and Inflammation in Rats. Phytomedicine 76, 153257. 10.1016/j.phymed.2020.153257 32534360

[B262] Wei X. Ma Y. Dong Z. Wang G. Lan X. Liao Z. (2021). Dehydrodiconiferyl Alcohol, a Lignan from Herpetospermum Pedunculosum, Alleviates Cholestasis by Activating Pathways Associated with the Farnesoid X Receptor. Phytomedicine 80, 153378. 10.1016/j.phymed.2020.153378 33113499

[B263] Williams S. Bledsoe R. K. Collins J. L. Boggs S. Lambert M. H. Miller A. B. (20032003). X-ray crystal Structure of the Liver X Receptor Beta Ligand Binding Domain: Regulation by a Histidine-Tryptophan Switch. J. Biol. Chem. 278, 27138–27143. 10.1074/jbc.M302260200 12736258

[B264] Willy P. J. Umesono K. Ong E. S. Evans R. M. Heyman R. A. Mangelsdorf D. J. (1995). LXR, a Nuclear Receptor that Defines a Distinct Retinoid Response Pathway. Genes Dev. 9, 1033–1045. 10.1101/gad.9.9.1033 7744246

[B265] Wink M. (2003). Evolution of Secondary Metabolites from an Ecological and Molecular Phylogenetic Perspective. Phytochemistry 64, 3–19. 10.1016/S0031-9422(03)00300-5 12946402

[B266] Wong C. P. Kaneda T. Morita H. (2014). Plant Natural Products as an Anti-lipid Droplets Accumulation Agent. J. Nat. Med. 68, 253–266. 10.1007/s11418-014-0822-3 24550097 PMC3948524

[B267] Wu T. Zhong L. Hong Z. Li Y. Liu X. Pan L. (2015). The Effects of Zanthoxylum Bungeanum Extract on Lipid Metabolism Induced by Sterols. J. Pharmacol. Sci. 127, 251–259. 10.1016/j.jphs.2014.12.002 25837921

[B268] Xiao P. T. Liu S. Y. Kuang Y. J. Jiang Z. M. Lin Y. Xie Z. S. (2021). Network Pharmacology Analysis and Experimental Validation to Explore the Mechanism of Sea Buckthorn Flavonoids on Hyperlipidemia. J. Ethnopharmacol. 264, 113380. 10.1016/j.jep.2020.113380 32918994

[B269] Xiao Q. Zhang S. Ren H. Du R. Li J. Zhao J. (2020). Ginsenoside Rg1 Alleviates ANIT-Induced Intrahepatic Cholestasis in Rats via Activating Farnesoid X Receptor and Regulating Transporters and Metabolic Enzymes. Chem. Biol. Interact. 324, 109062. 10.1016/j.cbi.2020.109062 32198087

[B270] Xie Z. Zhao J. Wang H. Jiang Y. Yang Q. Fu Y. (2020). Magnolol Alleviates Alzheimer’s Disease-like Pathology in Transgenic *C. elegans* by Promoting Microglia Phagocytosis and the Degradation of Beta-Amyloid through Activation of PPAR-γ. Biomed. Pharmacother. 124, 109886. 10.1016/j.biopha.2020.109886 32000045

[B271] Xu J. Gu W. Li C. Li X. Xing G. Li Y. (2016a). Epigallocatechin Gallate Inhibits Hepatitis B Virus via Farnesoid X Receptor Alpha. J. Nat. Med. 70, 584–591. 10.1007/s11418-016-0980-6 26968537

[B272] Xu W. Lu C. Zhang F. Shao J. Yao S. Zheng S. (2017a). Dihydroartemisinin Counteracts Fibrotic portal Hypertension via Farnesoid X Receptor-dependent Inhibition of Hepatic Stellate Cell Contraction. FEBS. J. 284, 114–133. 10.1111/febs.13956 27896916

[B273] Xu W. Lu C. Zhang F. Shao J. Zheng S. (2016b). Dihydroartemisinin Restricts Hepatic Stellate Cell Contraction via an FXR-S1PR2-dependent Mechanism. IUBMB Life 68, 376–387. 10.1002/iub.1492 27027402

[B274] Xu Y. Niu Y. Gao Y. Wang F. Qin W. Lu Y. (2017b). Borapetoside E, a Clerodane Diterpenoid Extracted from Tinospora Crispa, Improves Hyperglycemia and Hyperlipidemia in High-Fat-Diet-Induced Type 2 Diabetes Mice. J. Nat. Prod. 80, 2319–2327. 10.1021/acs.jnatprod.7b00365 28742368

[B275] Xue X. Quan Y. Gong L. Gong X. Li Y. (2020). A Review of the Processed Polygonum Multiflorum (Thunb.) for Hepatoprotection: Clinical Use, Pharmacology and Toxicology. J. Ethnopharmacol. 261, 113121. 10.1016/j.jep.2020.113121 32693115

[B276] Yang C. Li Q. Li Y. (2014). Targeting Nuclear Receptors with marine Natural Products. Mar. Drugs 12, 601–635. 10.3390/md12020601 24473166 PMC3944506

[B277] Yin Y. Gao L. Lin H. Wu Y. Han X. Zhu Y. (2017). Luteolin Improves Non-alcoholic Fatty Liver Disease in Db/db Mice by Inhibition of Liver X Receptor Activation to Down-Regulate Expression of Sterol Regulatory Element Binding Protein 1c. Biochem. Biophys. Res. Commun. 482, 720–726. 10.1016/j.bbrc.2016.11.101 27888103

[B278] Yoshikawa T. Shimano H. Yahagi N. Ide T. Amemiya-Kudo M. Matsuzaka T. (2002). Polyunsaturated Fatty Acids Suppress Sterol Regulatory Element-Binding Protein 1c Promoter Activity by Inhibition of Liver X Receptor (LXR) Binding to LXR Response Elements. J. Biol. Chem. 277 (3), 1705–1711. 10.1074/jbc.M105711200 11694526

[B279] Yu B. Z. Kaimal R. Bai S. El Sayed K. A. Tatulian S. A. Apitz R. J. (2009). Effect of Guggulsterone and Cembranoids of Commiphora Mukul on Pancreatic Phospholipase A(2): Role in Hypocholesterolemia. J. Nat. Prod. 72, 24–28. 10.1021/np8004453 19102680

[B280] Yu L. York J. von Bergmann K. Lutjohann D. Cohen J. C. Hobbs H. H. (2003). Stimulation of Cholesterol Excretion by the Liver X Receptor Agonist Requires ATP-Binding Cassette Transporters G5 and G8. J. Biol. Chem. 278, 15565–15570. 10.1074/jbc.M301311200 12601003

[B281] Zanella I. Biasiotto G. Holm F. di Lorenzo D. (2017). Cereal Lignans, Natural Compounds of Interest for Human Health? Nat. Product. Commun. 12, 1934578X1701200–146. 10.1177/1934578X1701200139 30549848

[B282] Zhang G. Sun X. Wen Y. Shi A. Zhang J. Wei Y. (2020a). Hesperidin Alleviates Cholestasis via Activation of the Farnesoid X Receptor *In Vitro* and *In Vivo* . Eur. J. Pharmacol. 885, 173498. 10.1016/j.ejphar.2020.173498 32841642

[B283] Zhang J. Zhao Y. Ren D. Yang X. (2020b). Effect of Okra Fruit Powder Supplementation on Metabolic Syndrome and Gut Microbiota Diversity in High Fat Diet-Induced Obese Mice. Food Res. Int. 130, 108929. 10.1016/j.foodres.2019.108929 32156377

[B284] Zhang L.-q. Zhao Y.-y. Huang C. Chen K.-x. Li Y.-m. (2016). Scrodentoids F-I, Four C19-Norditerpenoids from Scrophularia Dentata. Tetrahedron 72, 8031–8035. 10.1016/j.tet.2016.10.035

[B285] Zhang M. Pan H. Xu Y. Wang X. Qiu Z. Jiang L. (2017). Allicin Decreases Lipopolysaccharide-Induced Oxidative Stress and Inflammation in Human Umbilical Vein Endothelial Cells through Suppression of Mitochondrial Dysfunction and Activation of Nrf2. Cel Physiol. Biochem. 41, 2255–2267. 10.1159/000475640 28456799

[B286] Zhang T. Zhong S. Li T. Zhang J. (2020c). Saponins as Modulators of Nuclear Receptors. Crit. Rev. Food Sci. Nutr. 60, 94–107. 10.1080/10408398.2018.1514580 30582348

[B288] Zhang Y. Li F. Jiang X. Jiang X. Wang Y. Zhang H. (2020d). Sophoricoside Is a Selective LXRβ Antagonist with Potent Therapeutic Effects on Hepatic Steatosis of Mice. Phytother. Res. 34, 3168–3179. 10.1002/ptr.6747 32592532

[B289] Zhang Z. Burch P. E. Cooney A. J. Lanz R. B. Pereira F. A. Wu J. (2004). Genomic Analysis of the Nuclear Receptor Family: New Insights into Structure, Regulation, and Evolution from the Rat Genome. Genome Res. 14, 580–590. 10.1101/gr.2160004 15059999 PMC383302

[B290] Zhao A. Yu J. Lew. J. L. Huang L. Wright S. D. Cui J. (2004). Polyunsaturated Fatty Acids Are FXR Ligands and Differentially Regulate Expression of FXR Targets. DNA Cel Biol 23, 519–526. 10.1089/1044549041562267 15307955

[B291] Zhao H. L. Cho K. H. Ha Y. W. Jeong T. S. Lee W. S. Kim Y. S. (2006). Cholesterol-lowering Effect of Platycodin D in Hypercholesterolemic ICR Mice. Eur. J. Pharmacol. 537, 166–173. 10.1016/j.ejphar.2006.03.032 16626693

[B292] Zhao J. Khan S. I. Wang M. Vasquez Y. Yang M. H. Avula B. (2014). Octulosonic Acid Derivatives from Roman Chamomile (Chamaemelum Nobile) with Activities against Inflammation and Metabolic Disorder. J. Nat. Prod. 77, 509–515. 10.1021/np400780n 24471493

[B293] Zhao J. F. Jim Leu S. J. Shyue S. K. Su K. H. Wei J. Lee T. S. (2013). Novel Effect of Paeonol on the Formation of Foam Cells: Promotion of LXRα-ABCA1-dependent Cholesterol Efflux in Macrophages. Am. J. Chin. Med. 41, 1079–1096. 10.1142/s0192415x13500730 24117070

[B294] Zhao W. W. Guo W. W. Guo J. F. Wang X. Chen X. Q. Wu X. (2021). Three New Flavonoids from Penthorum Chinense Pursh and Their Docking Studies. Nat. Prod. Res. 35, 49–56. 10.1080/14786419.2019.1613394 31342796

[B295] Zhao X. Liu J. (2020). Chemical Constituents from the Fruits ofLigustrum Lucidum W.T.Aitonand Their Role on the Medicinal Treatment. Nat. Product. Commun. 15, 1934578X2092233. 10.1177/1934578x20922338

[B296] Zheng J. Li Z. Manabe Y. Kim M. Goto T. Kawada T. (2018a). Siphonaxanthin, a Carotenoid from Green Algae, Inhibits Lipogenesis in Hepatocytes via the Suppression of Liver X Receptor α Activity. Lipids 53, 41–52. 10.1002/lipd.12002 29446839

[B297] Zheng L. Yin L. Xu L. Qi Y. Li H. Xu Y. (2018b). Protective Effect of Dioscin against Thioacetamide-Induced Acute Liver Injury via FXR/AMPK Signaling Pathway *In Vivo* . Biomed. Pharmacother. 97, 481–488. 10.1016/j.biopha.2017.10.153 29091898

[B298] Zheng W. Lu Y. Lin S. Wang R. Qiu L. Zhu Y. (2017a). A Novel Class of Natural FXR Modulators with a Unique Mode of Selective Co-regulator Assembly. Chembiochem 18, 721–725. 10.1002/cbic.201700059 28186695

[B299] Zheng Z. Zhao Z. Li S. Lu X. Jiang M. Lin J. (2017b). Altenusin, a Nonsteroidal Microbial Metabolite, Attenuates Nonalcoholic Fatty Liver Disease by Activating the Farnesoid X Receptor. Mol. Pharmacol. 92, 425–436. 10.1124/mol.117.108829 28739572 PMC5588546

[B300] Zhou X. Liang Z. Li K. Fang W. Tian Y. Luo X. (2019). Exploring the Natural Piericidins as Anti-renal Cell Carcinoma Agents Targeting Peroxiredoxin 1. J. Med. Chem. 62, 7058–7069. 10.1021/acs.jmedchem.9b00598 31298537

[B301] Zhou Y. Ding Y. L. Zhang J. L. Zhang P. Wang J. Q. Li Z. H. (2018). Alpinetin Improved High Fat Diet-Induced Non-alcoholic Fatty Liver Disease (NAFLD) through Improving Oxidative Stress, Inflammatory Response and Lipid Metabolism. Biomed. Pharmacother. 97, 1397–1408. 10.1016/j.biopha.2017.10.035 29156529

[B302] Zhu J. Xu K. Zhang X. Cao J. Jia Z. Yang R. (2016). Studies on the Regulation of Lipid Metabolism and its Mechanism of the Iridoids Rich Fraction in Valeriana Jatamansi Jones. Biomed. Pharmacother. 84, 1891–1898. 10.1016/j.biopha.2016.10.099 27832992

[B303] Zhuang J. Zhang H. Zhou R. Chen L. Chen J. Shen X. (2013). Regulation of Prostaglandin F2α against β Amyloid Clearance and its Inflammation Induction through LXR/RXR Heterodimer Antagonism in Microglia. Prostaglandins Other Lipid Mediat 106, 45–52. 10.1016/j.prostaglandins.2013.09.002 24076168

[B304] Zou J. Jiang J. Diao Y. Y. Yang L. B. Huang J. Li H. L. (2012a). Cycloartane Triterpenoids from the Stems of Schisandra Glaucescens and Their Bioactivity. Fitoterapia 83, 926–931. 10.1016/j.fitote.2012.04.007 22537642

[B305] Zou J. Yang L. B. Jiang J. Diao Y. Y. Li X. N. Huang J. (2012b). Lanostane Triterpenoids from the Stems of Schisandra Glaucescens. Planta. Med. 78, 472–479. 10.1055/s-0031-1298214 22281717

[B306] Zou M. Nong C. Yu Z. Cai H. Jiang Z. Xue R. (2020). The Role of Invariant Natural Killer T Cells and Associated Immunoregulatory Factors in Triptolide-Induced Cholestatic Liver Injury. Food Chem. Toxicol. 146, 111777. 10.1016/j.fct.2020.111777 32987112

