# Supplementary material for: Natural Products Targeting Liver X Receptors or Farnesoid X Receptor
Source: Front Pharmacol. 2022 Jan 5;12:772435. doi: 10.3389/fphar.2021.772435 (PMC8766425; doi:10.3389/fphar.2021.772435)
Supplement: Supplementary file 1 [file DataSheet1.PDF]

Supporting information

Supplementary Table 1| Natural agonists targeted LXRα and LXRβ.

(#: The docking between ligands with not completely determined configuration and proteins is not done. -: The ligands could not fit into proteins.)

No.	Name	Natural product	Source	Classification	Activation/ Inhibition	S values of 1UHL (LXRα)	S values of 3IPQ (LXRα)	S values of 1P8D (LXRβ)
**1**	Geraniol	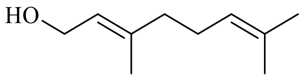	Essential oils of aromatic plants including *Cinnamomum tenuipilum* Kosterm (Lauraceae), *Valeriana officinalis* L. (Caprifoliaceae) and n-butanol extract (NE3) of *Panax notoginseng* (Burk.) F.H. Chen	Terpene	LXRα/FXR dual agonist	-4.476	-4.462	

No.	Name	Natural product	Source	Classification	Activation/ Inhibition	S values of 1UHL (LXRα)	S values of 3IPQ (LXRα)	S values of 1P8D (LXRβ)
**2**	Cineole	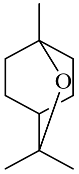	*Mentha longifolia* L. Most eucalyptus oils, teas rosemary, *Psidium* and many other essential oils	Terpene	LXRα/β dual agonist	-5.719	-6.644	-6.438
**3**	Squalene	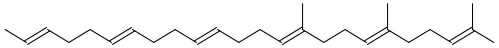	*Schizochytrium mangrovei* PQ6	Terpene	LXRα/β dual agonist	-7.307	-7.410	-8.938
**4**	(2R,3S)-5-hydroxymethylpterosin C	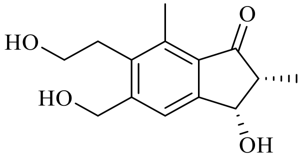	*Pteris cretica* L.	Terpene	LXRα/β dual agonist	-7.415	-7.579	-6.278
**5**	DTP1	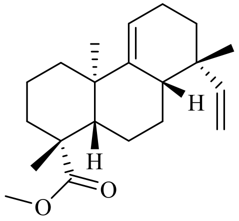	*Scoparia dulcis* L.	Terpene	LXRα/β dual agonist	-7.209	-8.059	-8.227

No.	Name	Natural product	Source	Classification	Activation/ Inhibition	S values of 1UHL (LXRα)	S values of 3IPQ (LXRα)	S values of 1P8D (LXRβ)
**6**	DTP3	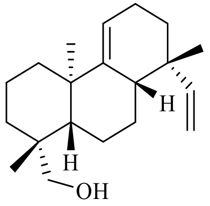	*Scoparia dulcis* L.	Terpene	LXRα/β dual agonist	-7.442	-7.930	-7.561
**7**	DTP5	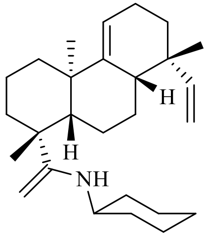	*Scoparia dulcis* L.	Terpene	LXRα/β dual agonist	-8.016	-8.446	-10.161
**8**	(3β, 24Z)-3,27-dihydroxy-lanosta-8,24-dien-1-one	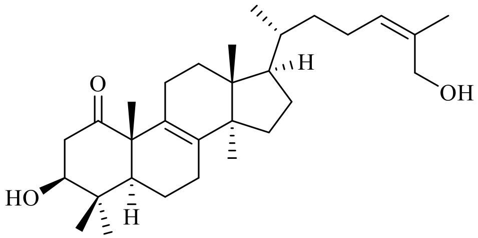	*Rigidoporus microporus*	Terpene	LXRα/β dual agonist	-8.266	-9.275	-9.774

No.	Name	Natural product	Source	Classification	Activation/ Inhibition	S values of 1UHL (LXRα)	S values of 3IPQ (LXRα)	S values of 1P8D (LXRβ)
**9**	(3β, 23S)-3,23-dihydroxy-7,9(11),24-lanostane-triene	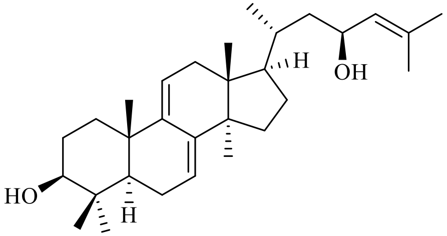	*Rigidoporus microporus*	Terpene	LXRα/β dual agonist	-8.276	-7.361	-9.174
**10**	Gynosaponin TR1	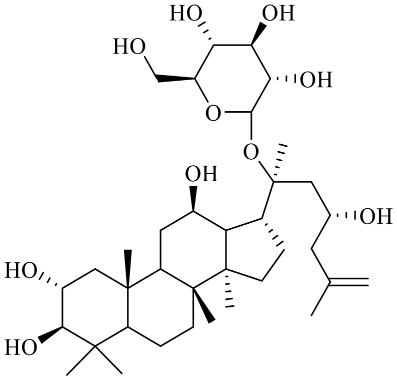	*Gynostemma pentaphyllum* (Thunb.) Makino	Terpene	LXRα agonist	#	#	
**11**	(-)-Acanthoic acid	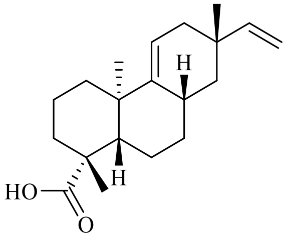	*Rollinia pittieri* Saff. and *Rollinia exsucca* (DC.) A.DC.	Terpene	LXRα agonist	-7.448	-8.394	

No.	Name	Natural product	Source	Classification	Activation/ Inhibition	S values of 1UHL (LXRα)	S values of 3IPQ (LXRα)	S values of 1P8D (LXRβ)
**12**	Polycarpol	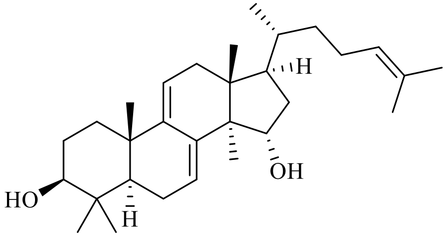	*Unonopsis glaucopetala* R.E.Fr. and *Minquartia guianensis* Aubl.	Terpene	LXRα agonist	-7.932	-7.011	
**13**	Paeoniflorin	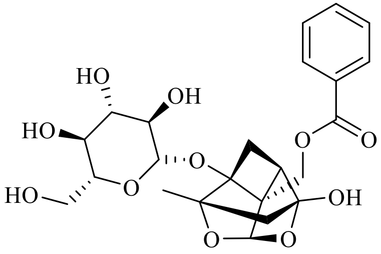	*Paeonia lactiflora* Pall.	Terpene	LXRα agonist	-8.222	-8.485	
**14**	Ganocalidin A	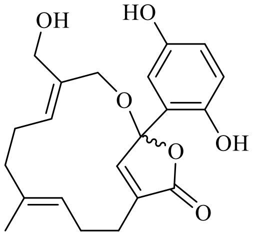	*Ganoderma capense*	Terpene	LXRα agonist	#	#	

No.	Name	Natural product	Source	Classification	Activation/ Inhibition	S values of 1UHL (LXRα)	S values of 3IPQ (LXRα)	S values of 1P8D (LXRβ)
**15**	Ganoboninones E	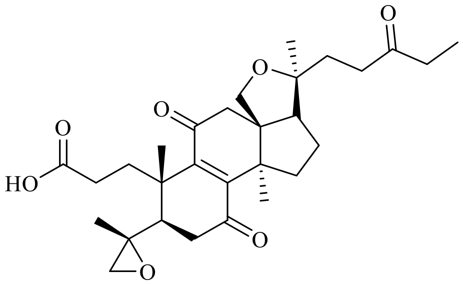	*Ganoderma boninense*	Terpene	LXRβ agonist			-8.122
**16**	Ganoboninketals A	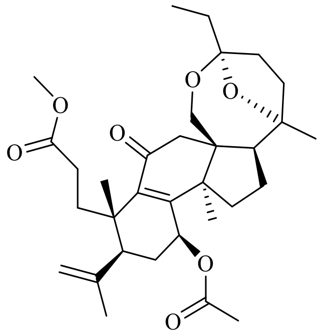	*Ganoderma boninense*	Terpene	LXRβ agonist			-

No.	Name	Natural product	Source	Classification	Activation/ Inhibition	S values of 1UHL (LXRα)	S values of 3IPQ (LXRα)	S values of 1P8D (LXRβ)
**17**	Ganoboninketals B	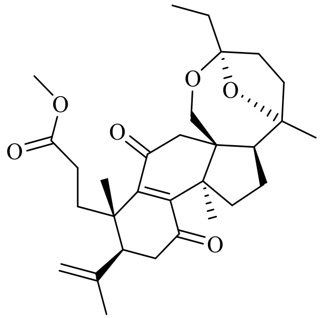	*Ganoderma boninense*	Terpene	LXRβ agonist			-5.784
**18**	Ganoboninketals C	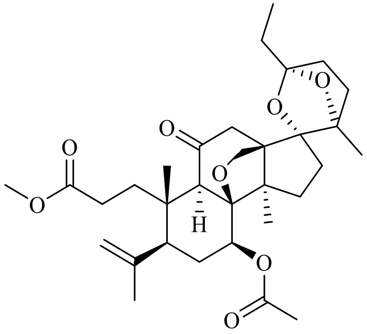	*Ganoderma boninense*	Terpene	LXRβ agonist			-
**19**	Triptolide	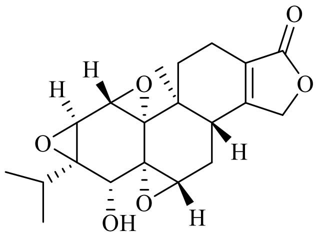	*Tripterygium wilfordii* Hook. f.	Terpene	Increase the LXRα protein expression and suppress FXR	-7.398	-8.722	

No.	Name	Natural product	Source	Classification	Activation/ Inhibition	S values of 1UHL (LXRα)	S values of 3IPQ (LXRα)	S values of 1P8D (LXRβ)
					protein expression			
**20**	Tirotundin	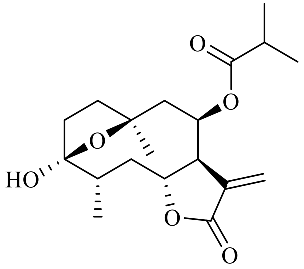	*Tithonia diversifolia* (Hemsl.) A. Gray	Terpene	LXRα/β and FXR dual agonists	-7.301	-8.441	-7.741
**21**	Tagitinin A	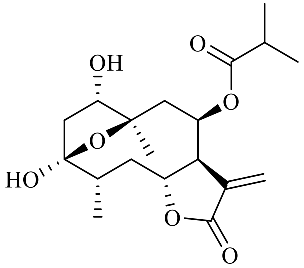	*Tithonia diversifolia* (Hemsl.) A. Gray	Terpene	LXRα/β and FXR dual agonists	-7.427	-8.476	-7.965
**22**	Platycodin D	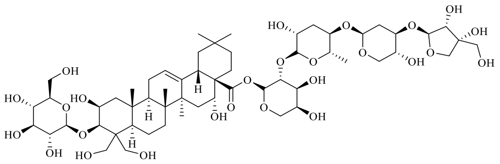	*Platycodon grandiflorum* (Jacq.) A.DC.	Terpene	Activate LXRα and FXR antagonist	-	-	

No.	Name	Natural product	Source	Classification	Activation/ Inhibition	S values of 1UHL (LXRα)	S values of 3IPQ (LXRα)	S values of 1P8D (LXRβ)
**23**	Saikosaponin a	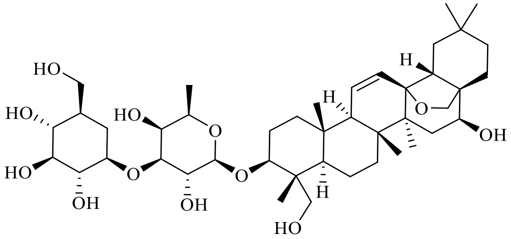	*Radix bupleuri*	Terpene	Activate LXRα	#	#	
**24**	Viperidone	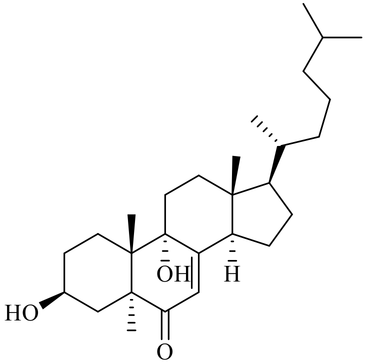	*Leptocereus quadricostatus* (Bello) Britton & Rose	Terpene	Activate LXRα	-7.268	-	
**25**	25-OCH3-PPD	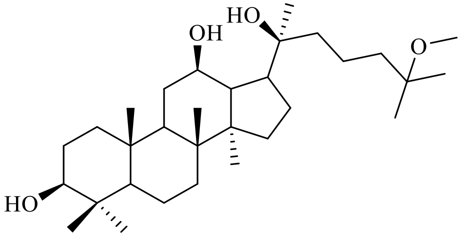	*Panax ginseng* C.A.Mey.	Terpene	Activate LXRα/β	#	#	#

No.	Name	Natural product	Source	Classification	Activation/ Inhibition	S values of 1UHL (LXRα)	S values of 3IPQ (LXRα)	S values of 1P8D (LXRβ)
**26**	Ginsenoside Ro	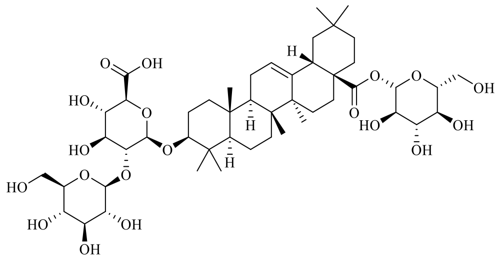	*Panax ginseng* C.A.Mey.	Terpene	Increase LXRα mRNA level	-	-	
**27**	Ginsenoside Rg_3	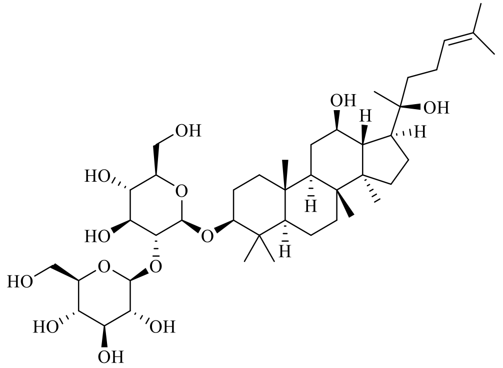	*Panax ginseng* C.A.Mey.	Terpene	Increase LXRα mRNA level	-8.779	-	
**28**	Ginsenoside Re	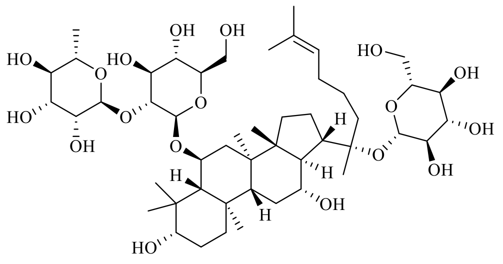	*Panax ginseng* C.A.Mey.	Terpene	Increase LXRα mRNA level	-	-	

No.	Name	Natural product	Source	Classification	Activation/ Inhibition	S values of 1UHL (LXRα)	S values of 3IPQ (LXRα)	S values of 1P8D (LXRβ)
**29**	Ginsenoside Rg_1	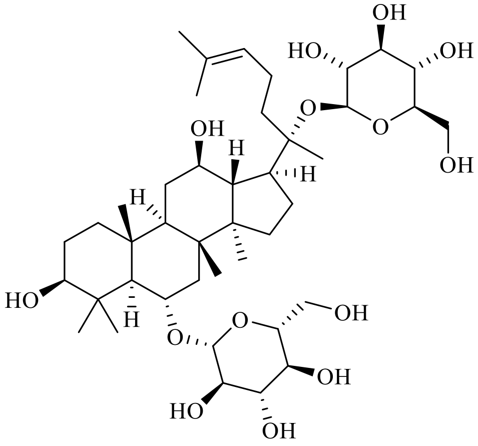	*Panax ginseng* C.A.Mey.	Terpene	Increase LXRα mRNA level and FXR agonist	-	-	
**30**	Ginsenoside Rg_2	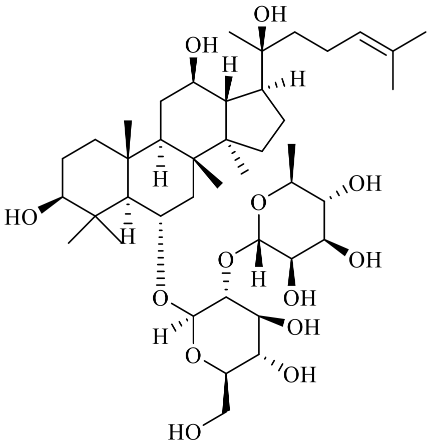	*Panax ginseng* C.A.Mey.	Terpene	Increase LXRα mRNA level	-	-	

No.	Name	Natural product	Source	Classification	Activation/ Inhibition	S values of 1UHL (LXRα)	S values of 3IPQ (LXRα)	S values of 1P8D (LXRβ)
**31**	Notoginsenoside R1	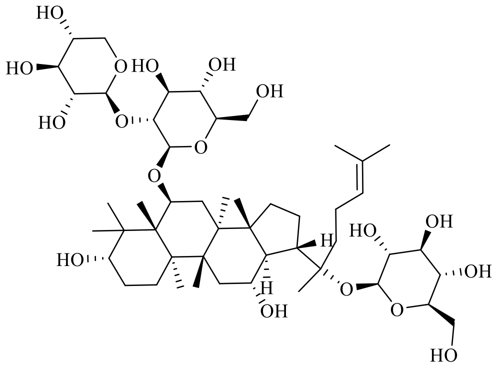	*Panax Notoginseng* (Burkill) F.H.Chen	Terpene	LXRα agonist	-	-	
**32**	Ginsenoside CK	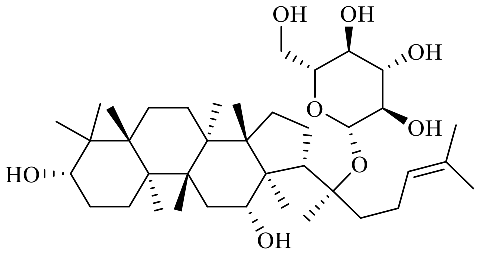	*Panax notoginseng* (Burkill) F.H.Chen	Terpene	LXRα agonist	-	-	
**33**	Cyanidin	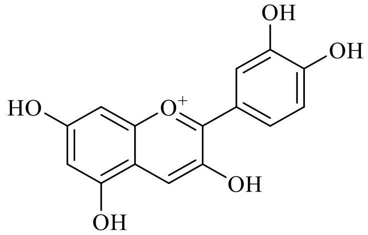	Fruits and vegetables	Flavonoid	LXRα/β dual agonist	-8.455	-9.187	-8.715

No.	Name	Natural product	Source	Classification	Activation/ Inhibition	S values of 1UHL (LXRα)	S values of 3IPQ (LXRα)	S values of 1P8D (LXRβ)
**34**	Hesperetin	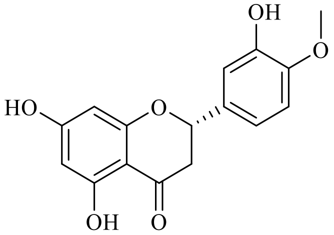	Citrus fruits such as oranges and grapefruit	Flavonoid	Activate LXRα	-8.456	-7.986	
**35**	Chrysin	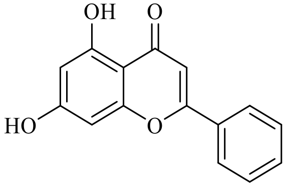	Honey, propolis, and plant extracts	Flavonoid	Increase LXRα mRNA level	-7.915	-7.701	
**36**	Daidzein	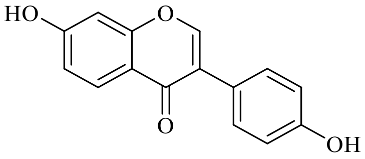	Soybeans	Flavonoid	Data indicative of indirect modulation of LXRα/β	-8.229	-8.010	-7.706
**37**	Genistein	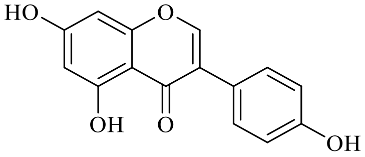	Soybeans	Flavonoid	Data indicative of indirect modulation of	-7.250	-8.088	-7.692

No.	Name	Natural product	Source	Classification	Activation/ Inhibition	S values of 1UHL (LXRα)	S values of 3IPQ (LXRα)	S values of 1P8D (LXRβ)
					LXRα/β			
**38**	Quercetin	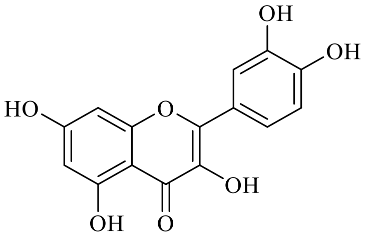	*Medicago sativa* L.	Flavonoid	Activate LXRα	-6.048	-7.434	
**39**	Naringenin	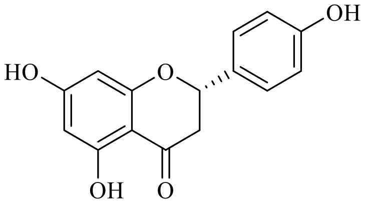	Grapefruits, oranges, and tomatoes	Flavonoid	Activate LXRα	-7.966	-7.339	
**40**	Iristectorigenin B	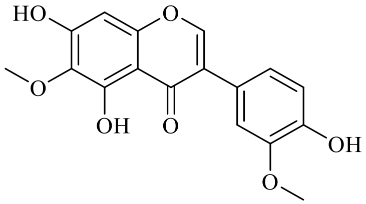	*Belamcanda chinensis*	Flavonoid	LXRα/β dual agonist	-7.738	-7.371	-7.448

No.	Name	Natural product	Source	Classification	Activation/ Inhibition	S values of 1UHL (LXRα)	S values of 3IPQ (LXRα)	S values of 1P8D (LXRβ)
**41**	Kaempferol-3-O-β-D-glucopyranoside	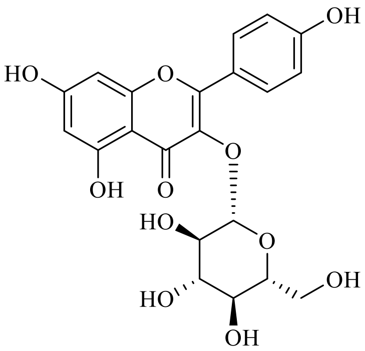	*Cornus alternifolia* L. f. (Cornaceae), as well as in food plants like black beans (*Phaseolus vulgaris* L.)	Flavonoid	LXRα/β dual agonist	-7.321	-10.108	-8.282
**42**	Kaempferol	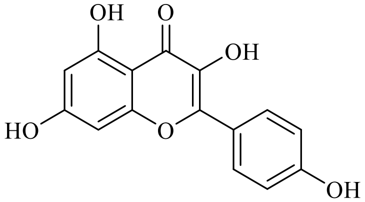	Plant-based foods	Flavonoid	LXRβ agonist			-6.578
**43**	Formononetin	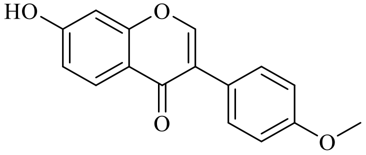	*Dalbergia ecastophyllum* (L.) Taub.	Flavonoid	LXRα/β dual agonist	-8.353	-8.168	-7.436

No.	Name	Natural product	Source	Classification	Activation/ Inhibition	S values of 1UHL (LXRα)	S values of 3IPQ (LXRα)	S values of 1P8D (LXRβ)
**44**	Quercetin-3-O-glucuronide	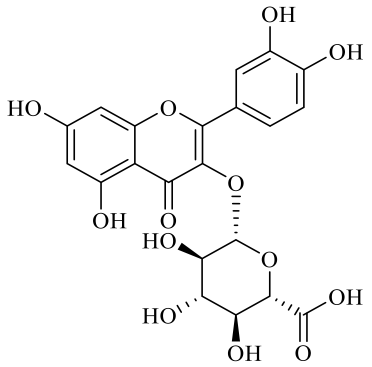	*Nelumbo nucifera* Gaertn.	Flavonoid	LXRα agonist	-6.494	-	
**45**	Butein	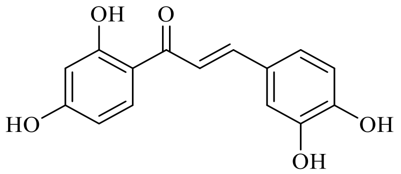	*Rhus verniciflua* Stokes	Flavonoid	Activate LXRα	-7.401	-7.608	
**46**	Scutellarein (Sc)	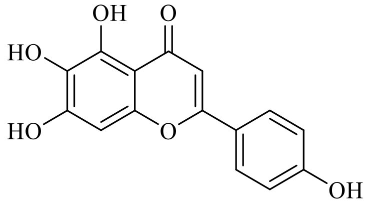	Erigeron *breviscapus* (vant.)	Flavonoid	Activate LXRα	-7.511	-7.581	

No.	Name	Natural product	Source	Classification	Activation/ Inhibition	S values of 1UHL (LXRα)	S values of 3IPQ (LXRα)	S values of 1P8D (LXRβ)
**47**	Puerarin	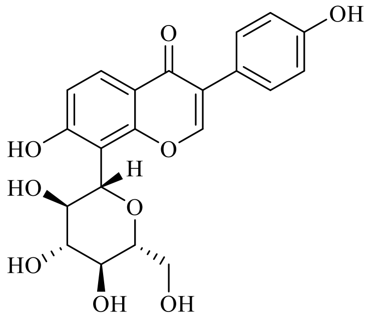	*Radix Pueraria* or *Pueraria lobate*	Flavonoid	Activate LXRα	-8.732	-7.829	
**48**	Isoliquiritigenin	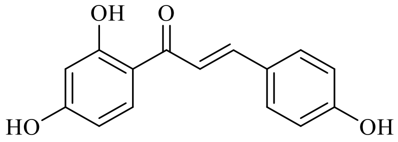	*Glycyrrhizae* species such as *Glycyrrhizae glabra*	Flavonoid	Activate LXRα	-7.654	-7.740	
**49**	Rutin	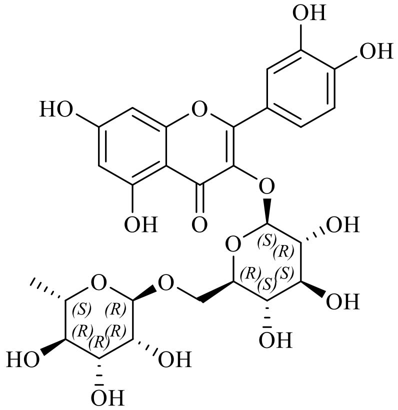	*Zanthoxylum bungeanum* Maxim.	Flavonoid	Promote LXRα expression	-8.216	-	

No.	Name	Natural product	Source	Classification	Activation/ Inhibition	S values of 1UHL (LXRα)	S values of 3IPQ (LXRα)	S values of 1P8D (LXRβ)
**50**	Hyperin	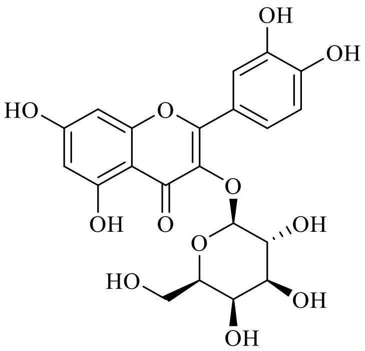	*Zanthoxylum bungeanum* Maxim.	Flavonoid	Promote LXRα expression	-7.011	-8.665	
**51**	SPF1	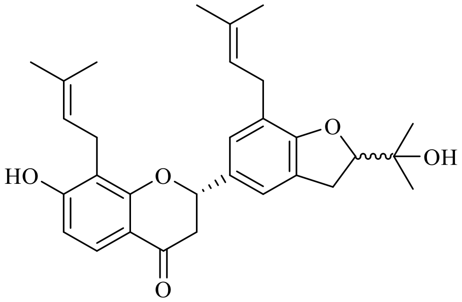	*Sophora tonkinensis* Gagnep.	Flavonoid	Activate RXR/LXR	#	#	#

No.	Name	Natural product	Source	Classification	Activation/ Inhibition	S values of 1UHL (LXRα)	S values of 3IPQ (LXRα)	S values of 1P8D (LXRβ)
**52**	SPF2	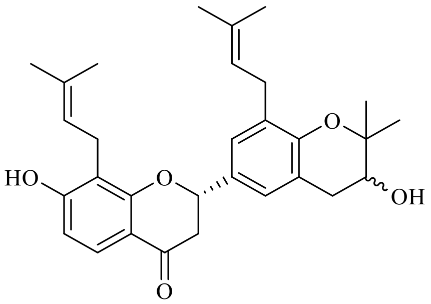	*Sophora tonkinensis* Gagnep.	Flavonoid	Activate RXR/LXR	#	#	#
**53**	Kuwanon G	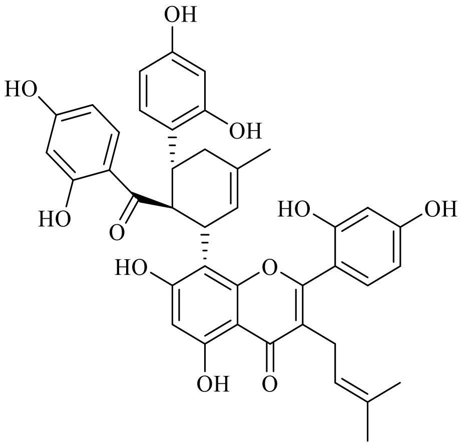	*Morus alba* L.	Flavonoid	Increase LXRα expression	-	-	
**54**	Tetramethylpyrazine (TMP)	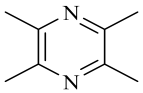	*Ligusticum chuanxiong*	Alkaloid	Activate LXRα	-5.523	-6.076	

No.	Name	Natural product	Source	Classification	Activation/ Inhibition	S values of 1UHL (LXRα)	S values of 3IPQ (LXRα)	S values of 1P8D (LXRβ)
**55**	Leonurine	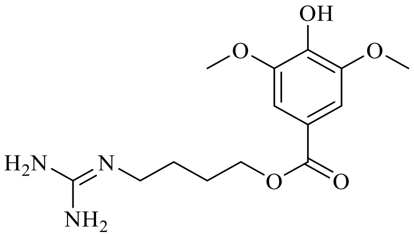	*Herba leonuri*	Alkaloid	Increase LXRα expression	-5.380	-6.137	
**56**	Paxilline	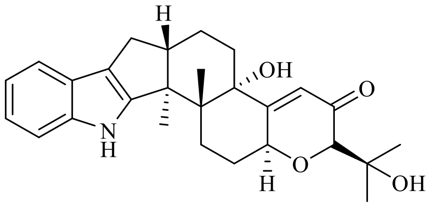	*Penicillium paxilli*	Alkaloid/ Steroid	LXRα/β dual agonist	-8.250	-7.477	-9.633
**57**	Oxepinamides D	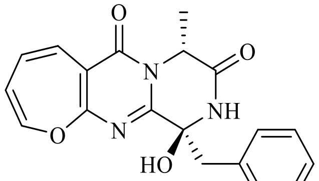	*Aspergillus puniceus* F02Z-1744	Alkaloid	LXRα agonist	-8.339	-9.913	

No.	Name	Natural product	Source	Classification	Activation/ Inhibition	S values of 1UHL (LXRα)	S values of 3IPQ (LXRα)	S values of 1P8D (LXRβ)
**58**	Oxepinamides E	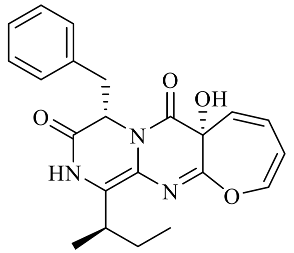	*Aspergillus puniceus* F02Z-1744	Alkaloid	LXRα agonist	-6.076	-10.567	
**59**	Oxepinamides F	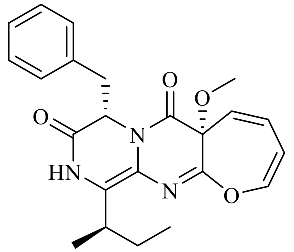	*Aspergillus puniceus* F02Z-1744	Alkaloid	LXRα agonist	-8.607	-9.487	
**60**	Oxepinamides G	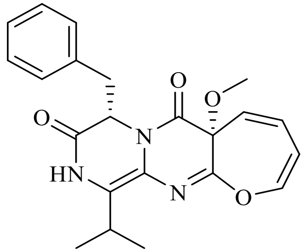	*Aspergillus puniceus* F02Z-1744	Alkaloid	LXRα agonist	-8.480	-10.775	

No.	Name	Natural product	Source	Classification	Activation/ Inhibition	S values of 1UHL (LXRα)	S values of 3IPQ (LXRα)	S values of 1P8D (LXRβ)
**61**	Oxepinamides H	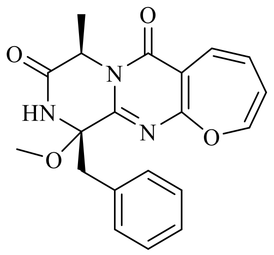	*Aspergillus puniceus* SCSIO z021	Alkaloid	LXRα agonist	-8.420	-9.495	
**62**	Oxepinamides I	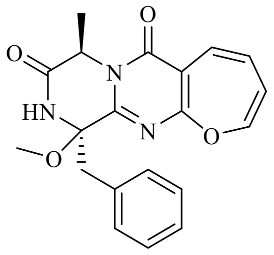	*Aspergillus puniceus* SCSIO z021	Alkaloid	LXRα agonist	-8.452	-9.902	
**63**	Oxepinamides J	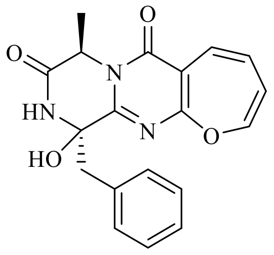	*Aspergillus puniceus* SCSIO z021	Alkaloid	LXRα agonist	-7.166	-9.673	

No.	Name	Natural product	Source	Classification	Activation/ Inhibition	S values of 1UHL (LXRα)	S values of 3IPQ (LXRα)	S values of 1P8D (LXRβ)
**64**	Oxepinamides K	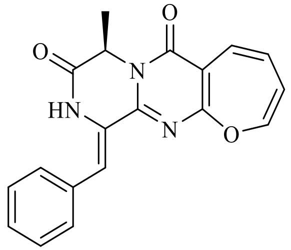	*Aspergillus puniceus* SCSIO z021	Alkaloid	LXRα agonist	-8.440	-9.365	
**65**	Puniceloids A	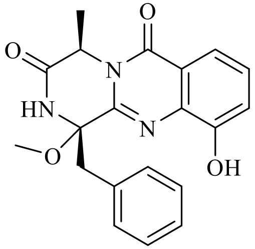	*Aspergillus puniceus* SCSIO z021	Alkaloid	LXRα agonist	-6.668	-9.823	
**66**	Puniceloids B	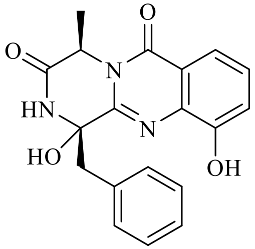	*Aspergillus puniceus* SCSIO z021	Alkaloid	LXRα agonist	-8.251	-9.140	

No.	Name	Natural product	Source	Classification	Activation/ Inhibition	S values of 1UHL (LXRα)	S values of 3IPQ (LXRα)	S values of 1P8D (LXRβ)
**67**	Puniceloids C	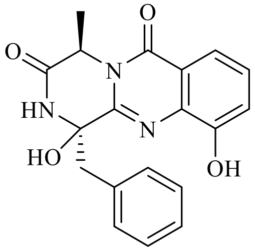	*Aspergillus puniceus* SCSIO z021	Alkaloid	LXRα agonist	-8.379	-9.095	
**68**	Puniceloids D	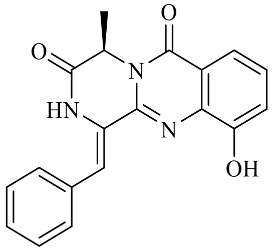	*Aspergillus puniceus* SCSIO z021	Alkaloid	LXRα agonist	-8.292	-8.920	
**69**	Scequinadoline D	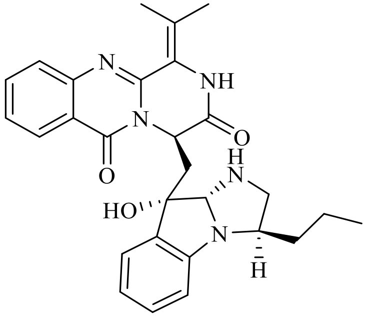	*Scedosporium apiospermum* F41-1	Alkaloid	Stimulate LXRα mRNA expression	-	-	

No.	Name	Natural product	Source	Classification	Activation/ Inhibition	S values of 1UHL (LXRα)	S values of 3IPQ (LXRα)	S values of 1P8D (LXRβ)
**70**	β-sitosterol	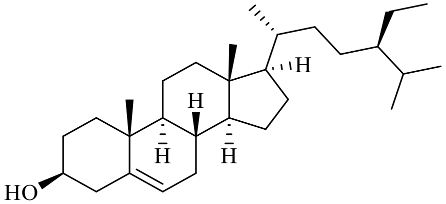	Plant oils and nuts	Steroid	Increase LXRα/β expression	-	-	-
**71**	Campesterol	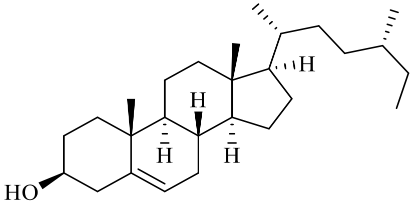	Plant oils and nuts	Steroid	Increase LXRα/β expression	-	-	-
**72**	Sitostanol	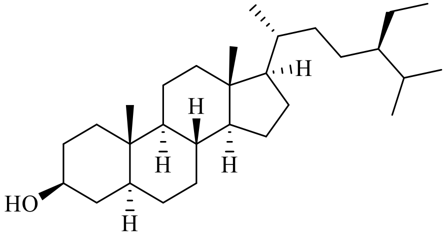	Plant oils and nuts	Steroid	Increase LXRα/β expression	-7.701	-7.196	-9.611
**73**	YT-32	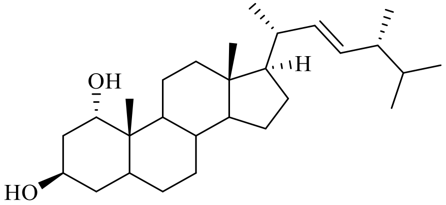	Plant oils and nuts	Steroid	Increase LXRα/β expression	#	#	#

No.	Name	Natural product	Source	Classification	Activation/ Inhibition	S values of 1UHL (LXRα)	S values of 3IPQ (LXRα)	S values of 1P8D (LXRβ)
**74**	24(S)-stigmast-5-ene-3β,24-ols	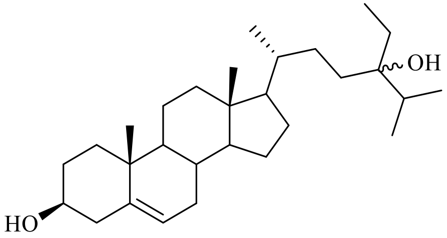	*Ficus pumila* L.	Steroid	LXRβ agonist			#
**75**	24(S)-stigmast-5,28-diene-3β,24-ols	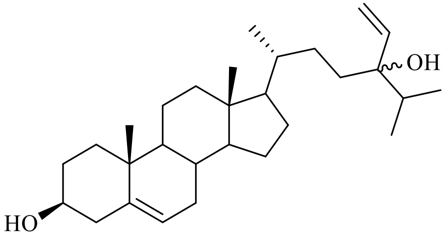	Genus *Sargassum*	Steroid	LXRβ agonist			#
**76**	Fucosterol	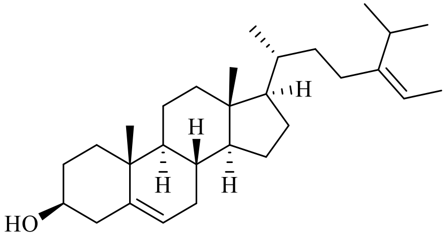	Marine algae	Steroid	LXRα/β dual agonist	-8.409	-	-10.056

No.	Name	Natural product	Source	Classification	Activation/ Inhibition	S values of 1UHL (LXRα)	S values of 3IPQ (LXRα)	S values of 1P8D (LXRβ)
**77**	24(S)-Saringosterol	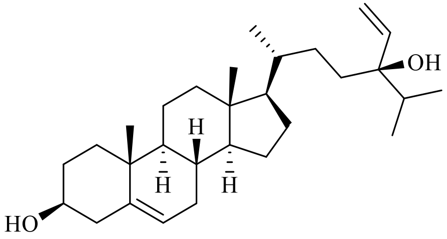	*Sargassum fusiforme*	Steroid	LXRβ agonist			-10.449
**78**	4-cholesten-3-one	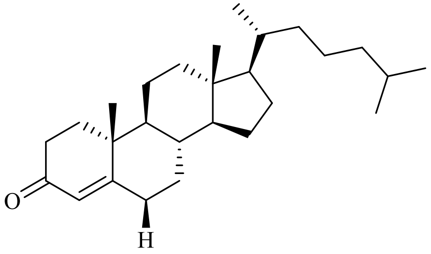	*Laurencia papillosa,* marine algae, marine fish, and plant roots	Steroid	LXRβ agonist			-8.880
**79**	Gorgostane-3β,9α,5α,6β,11α-tetrol	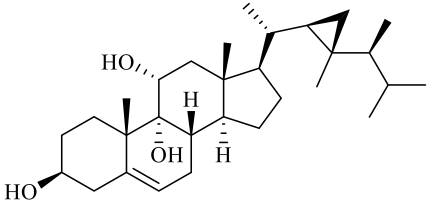	*Plexaura species*	Steroid	Activate LXRα	-	-	

No.	Name	Natural product	Source	Classification	Activation/ Inhibition	S values of 1UHL (LXRα)	S values of 3IPQ (LXRα)	S values of 1P8D (LXRβ)
**80**	Gorgost-5-ene-3β,9α,11α-triol	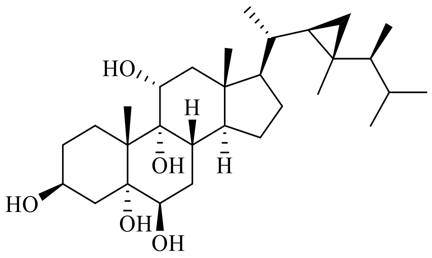	*Plexaura species*	Steroid	Activate LXRα	-	-	
**81**	Ethyl 2,4,6-trihydroxybenzoate (ETB)	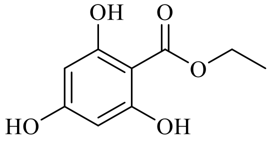	*Celtis biondii* Pamp.	Phenol	LXRα/β dual agonist	-5.380	-5.763	-5.578
**82**	Paeonol	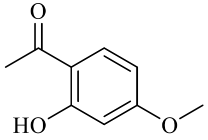	*Paeonia suffruticosa*	Phenol	LXRα agonist	-5.410	-7.074	
**83**	Resveratrol	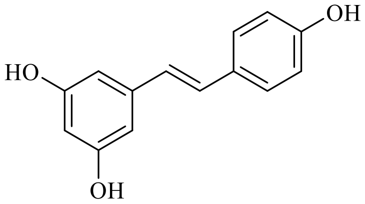	Red wine, berries and peanuts	Phenol	Upregulate LXRα	-7.929	-7.928	

No.	Name	Natural product	Source	Classification	Activation/ Inhibition	S values of 1UHL (LXRα)	S values of 3IPQ (LXRα)	S values of 1P8D (LXRβ)
**84**	Danshensu	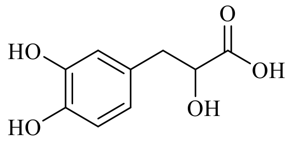	*Salvia miltiorrhiza* Bunge	Phenol	LXRα/FXR dual agonist	-5.952	-6.746	
**85**	Rosmarinic acid	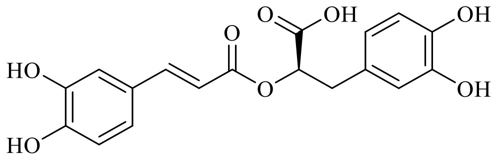	*Salvia miltiorrhiza* Bunge	Phenol	LXRα/FXR dual agonist	-6.972	-9.361	
**86**	Salvianolic acid A	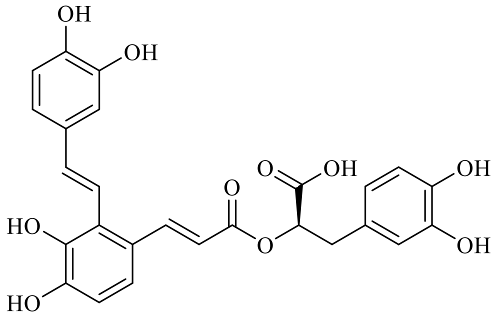	*Salvia miltiorrhiza* Bunge	Phenol	LXRα/FXR dual agonist	-7.429	-9.733	

No.	Name	Natural product	Source	Classification	Activation/ Inhibition	S values of 1UHL (LXRα)	S values of 3IPQ (LXRα)	S values of 1P8D (LXRβ)
**87**	Salvianolic acid B	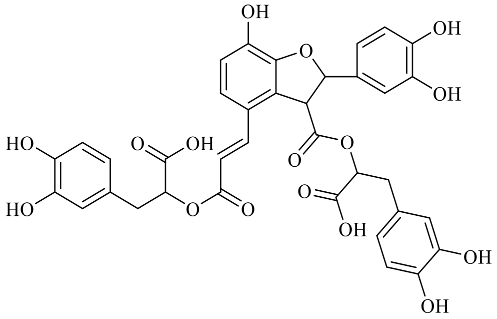	*Salvia miltiorrhiza* Bunge	Phenol	LXRα/FXR dual agonist	-	-	
**88**	Methyl gallate	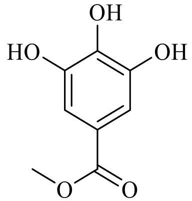	*Talisia nervosa* Radlk	Phenol	Activate LXRα	-5.877	-6.125	
**89**	Ethyl gallate	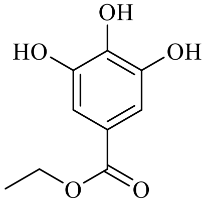	*Talisia nervosa* Radlk	Phenol	Activate LXRα	-5.448	-5.823	
**90**	Riccardin C	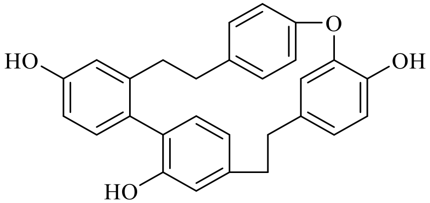	*Blasia pusilla* L. and *Reboulia hemisphaerica*	Phenol	LXRα agonist and LXRβ antagonist	-	-	-

No.	Name	Natural product	Source	Classification	Activation/ Inhibition	S values of 1UHL (LXRα)	S values of 3IPQ (LXRα)	S values of 1P8D (LXRβ)
**91**	Podocarpic acid	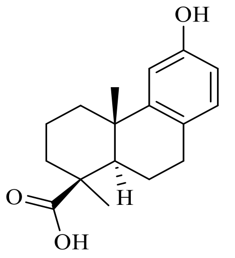	Plant resin	Phenol	LXRα/β dual agonist	-6.796	-8.281	-7.337
**92**	Podocarpic acid imide	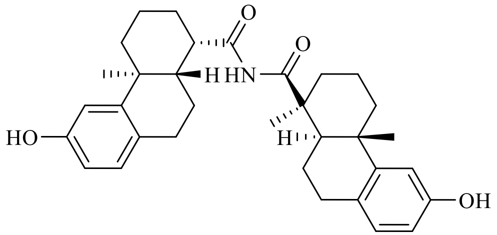	Plant resin	Phenol	LXRα/β dual agonist	-7.640	-	-10.158
**93**	Podocarpic acid anhydride	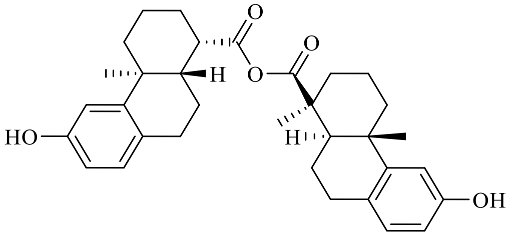	Plant resin	Phenol	LXRα/β dual agonist	-7.269	-	-10.462

No.	Name	Natural product	Source	Classification	Activation/ Inhibition	S values of 1UHL (LXRα)	S values of 3IPQ (LXRα)	S values of 1P8D (LXRβ)
**94**	Podocarpic acid anhydride acetate	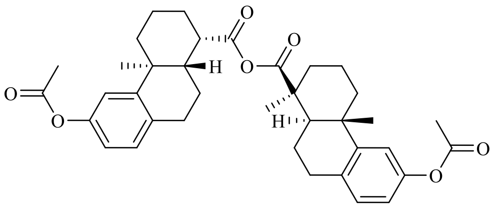	Plant resin	Phenol	LXRα/β dual agonist	-7.899	-	-11.457
**95**	Sesamol	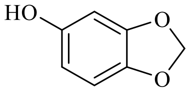	*Sesamum indicum* L.	Phenylpropanoid	LXRα/β dual agonist	-6.382	-6.169	-5.447
**96**	Magnolol	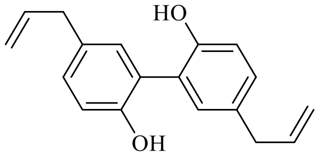	*Magnolia officinalis* Rehder & E.H.Wilson	Phenylpropanoid	LXRα/β dual agonist	-7.532	-7.758	-7.134
**97**	Honokiol	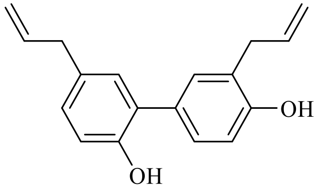	*Magnolia officinalis* Rehder & E.H.Wilson	Phenylpropanoid	LXR/RXR dual agonist	-6.942	-8.207	-7.174
**98**	Herniarin	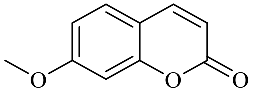	*Artemisia dracunculus* L.	Phenylpropanoid	Upregulate LXRα/β	-6.832	-6.647	-5.896

No.	Name	Natural product	Source	Classification	Activation/ Inhibition	S values of 1UHL (LXRα)	S values of 3IPQ (LXRα)	S values of 1P8D (LXRβ)
**99**	Emodin	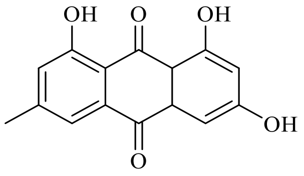	*Rheum officinale* Baill., *Rheum palmatum* L. and *Polygonam cuspidatum*	Anthraquinone	Activate PPARγ/LXR/ ABCA1/ABCG1 signaling pathway	-8.017	-8.399	-7.856
**100**	Octulosonic acid derivative	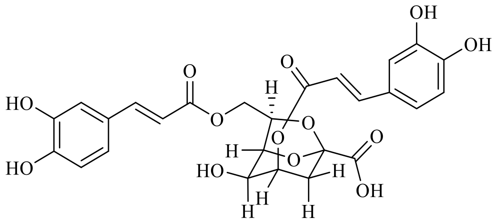	*Chamaemelum nobile* L.	Octulosonic acid derivative	LXRα agonist	-8.229	-	
**101**	Octulosonic acid derivative	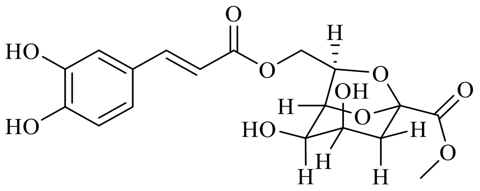	*Chamaemelum nobile* L.	Octulosonic acid derivative	LXRα agonist	-7.652	-8.121	

No.	Name	Natural product	Source	Classification	Activation/ Inhibition	S values of 1UHL (LXRα)	S values of 3IPQ (LXRα)	S values of 1P8D (LXRβ)
**102**	Pestalotioquinoside C	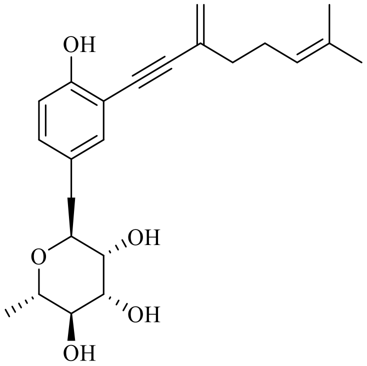	*Pestalotiopsis neglecta* SCSIO41403	Ene-yne hydroquinones	LXRα agonist	-8.253	-8.113	
**103**	Pestalotiochromones A	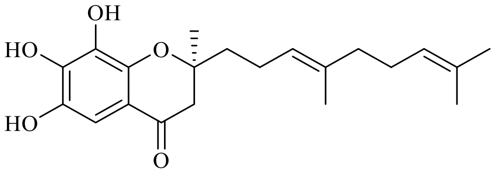	*Pestalotiopsis neglecta* SCSIO41403	Phenol derivative	LXRα agonist	-6.766	-8.208	
**104**	Betaine	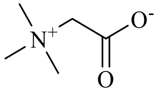	Wheat products, spinach, pretzels and shrimp	Trimethyl glycine	Increase LXRα expression	-4.511	-5.066	
**105**	Allicin	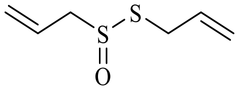	Garlic	Sulfoxide	Increase LXRα expression	-3.004	-4.092	

No.	Name	Natural product	Source	Classification	Activation/ Inhibition	S values of 1UHL (LXRα)	S values of 3IPQ (LXRα)	S values of 1P8D (LXRβ)
**106**	Taurine (2-aminoethanesulfonic acid)	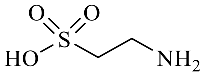	Seafood	Aminoethanesulfonic acid	LXRα agonist	-4.530	-4.120	
**107**	5-hydroxy-3-methoxy-5-methyl-4-butylfuran-2(5H)-one	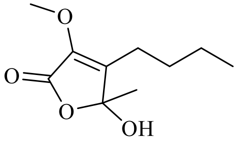	*Setosphaeria* sp. SCSIO41009	Furanone	LXRα agonist	-4.753	-4.946	
**108**	Setosphapyrone C	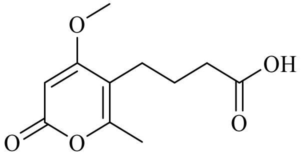	*Setosphaeria* sp. SCSIO41009	Other	Promote LXRα/ABCA1 pathway	-6.051	-6.492	
**109**	Setosphapyrone D	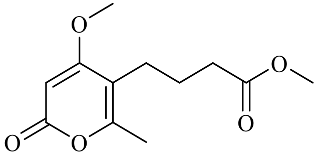	*Setosphaeria* sp. SCSIO41009	Other	Promote LXRα/ABCA1 pathway	-5.264	-6.414	

Supplementary Table 2| Natural antagonists targeted LXRα and LXRβ.
(#: The docking between ligands with not completely determined configuration and proteins is not done. -: The ligands could not fit into proteins.)

No.	Name	Natural product	Source	Classification	Activation/ Inhibition	S values of 1UHL (LXRα)	S values of 3IPQ (LXRα)	S values of 1P8D (LXRβ)
**110**	Iridoid	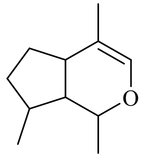	*Valeriana jatamansi* Jones	Terpene	Inhibit LXRα	-6.171	-6.828	
**111**	Lucidone	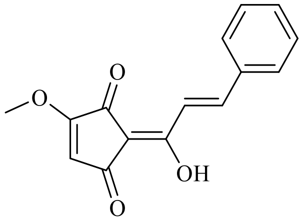	*Lindera erythrocarpa* Makino	Terpene	Inhibit LXRα expression	-8.056	-7.377	
**112**	Paraconiothins C	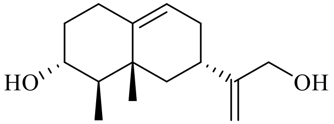	*Paraconiothyrium brasiliense* ECN258	Terpene	LXRα antagonist	-7.741	-7.240	
**113**	Paraconiothins I	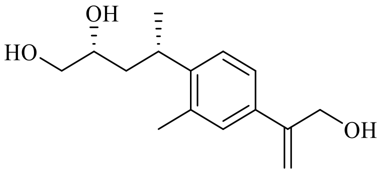	*Paraconiothyrium brasiliense* ECN258	Terpene	LXRα antagonist	-4.385	-5.854	

No.	Name	Natural product	Source	Classification	Activation/ Inhibition	S values of 1UHL (LXRα)	S values of 3IPQ (LXRα)	S values of 1P8D (LXRβ)
**114**	Ursolic acid	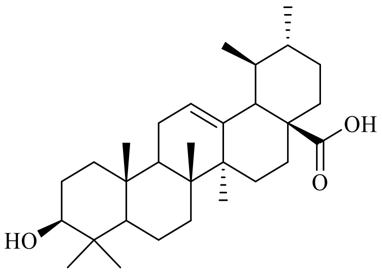	*Cornus officinalis* Siebold & Zucc. and other plants	Terpene	LXRα antagonist and activate FXR	-7.930	-	
**115**	Asiatic acid	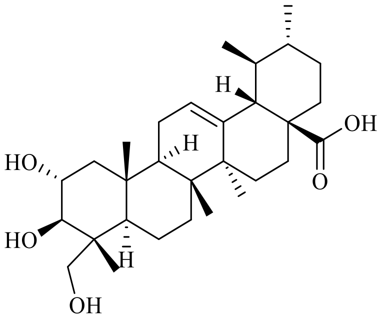	*Potentilla chinensis* Ser.	Terpene	Downregulate LXRα	-7.113	-	
**116**	Borapetoside E	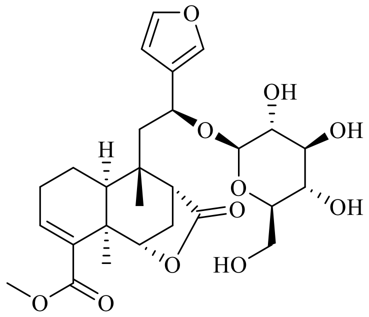	*Tinospora crispa* (L.) Hook. f. & Thomson	Terpene	Downregulate LXRα	-7.919	-	

No.	Name	Natural product	Source	Classification	Activation/ Inhibition	S values of 1UHL (LXRα)	S values of 3IPQ (LXRα)	S values of 1P8D (LXRβ)
**117**	20(S)-protopanaxatriol (PPT)	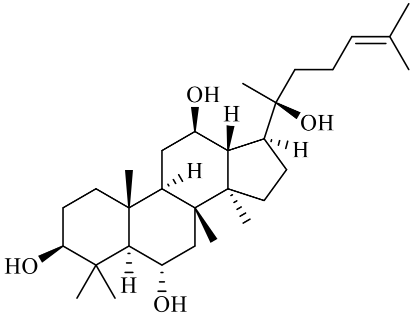	*Panax ginseng* C. A. Meyer	Terpene	LXRα antagonist	-8.285	-	
**118**	Luteolin	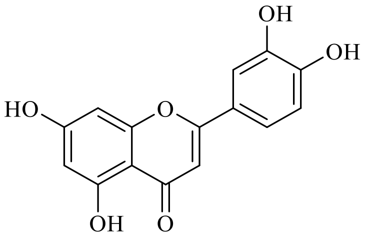	Carrots, peppers, celery, parsley, and spinach	Flavonoid	LXRα/β dual antagonist or LXRα agonist	-8.056	-7.377	-6.636
**119**	Morin	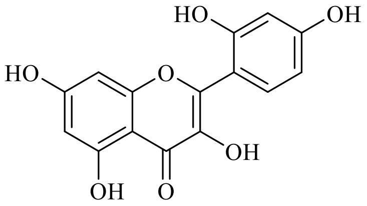	*Tartary buckwheat*, mulberry, jackfruit, green tea, osage orange and many other plants	Flavonoid	LXRα/β dual antagonist	-6.683	-7.578	-6.508

No.	Name	Natural product	Source	Classification	Activation/ Inhibition	S values of 1UHL (LXRα)	S values of 3IPQ (LXRα)	S values of 1P8D (LXRβ)
**120**	Licochalcone A	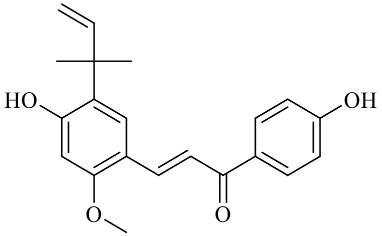	*Glycyrrhiza* plant (Leguminosae) (e.g., *Glycyrrhiza glabra* L. or *Glycyrrhiza inflata* Batalin)	Flavonoid	LXRα antagonist	-8.573	-9.207	
**121**	Isorhamnetin	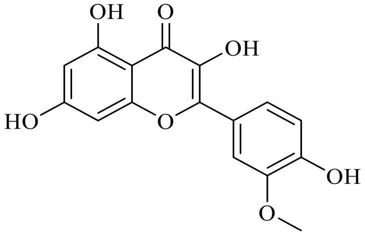	*Hippophae rhamnoides* L.	Flavonoid	Inhibit or increase LXRα expression	-7.23	-7.346	
**122**	Alpinetin	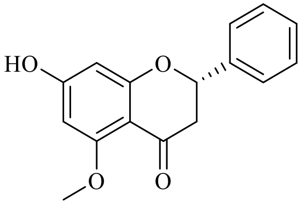	*Alpinia katsumadai* Hayata	Flavonoid	Inhibit LXRα expression	-8.158	-7.642	

No.	Name	Natural product	Source	Classification	Activation/ Inhibition	S values of 1UHL (LXRα)	S values of 3IPQ (LXRα)	S values of 1P8D (LXRβ)
**123**	Sophoricoside	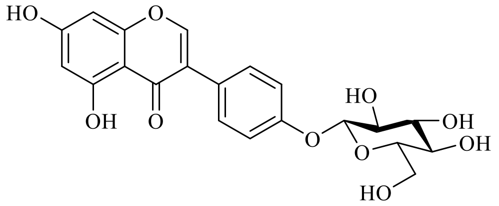	*Styphnolobium Japonicum* (L.) Schott	Flavonoid	LXRβ antagonist			-8.889
**124**	Xanthohumol	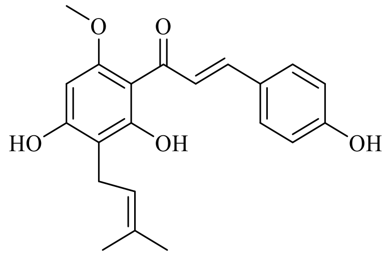	*Humulus lupulus* L.	Flavonoid	Counteract LXRα activation and a selective bile acid receptor modulator (SBARM)	-8.676	-9.127	
**125**	Silymarin	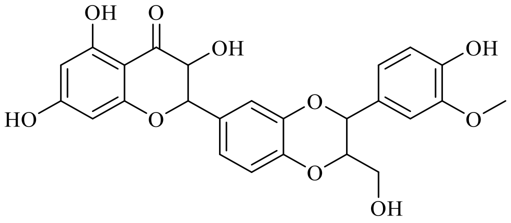	*Silybum marianum* (L.) Gaertn.	Flavonolignan	Inhibit LXRβ upregulation			-10.155

No.	Name	Natural product	Source	Classification	Activation/ Inhibition	S values of 1UHL (LXRα)	S values of 3IPQ (LXRα)	S values of 1P8D (LXRβ)
**126**	Ergosterol	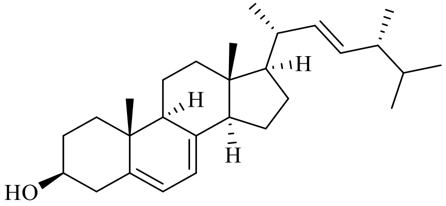	*Tolypocladium niveum*	Steroid	LXRα antagonist	-7.930	-	
**127**	Ergostan-6,8,22-trien-3-ol	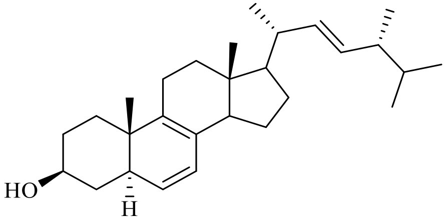	*Tolypocladium niveum*	Steroid	LXRα antagonist	#	#	
**128**	Ergostan-4,6,8,22-tetraen-3-one	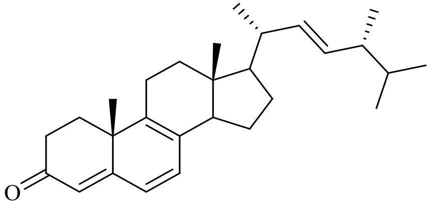	*Tolypocladium niveum*	Steroid	LXRα antagonist	#	#	
**129**		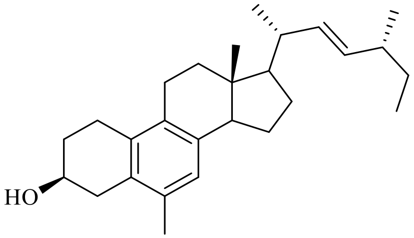	*Colletotrichum dematium*	Steroid	LXRα antagonist	#	#	

No.	Name	Natural product	Source	Classification	Activation/ Inhibition	S values of 1UHL (LXRα)	S values of 3IPQ (LXRα)	S values of 1P8D (LXRβ)
**130**		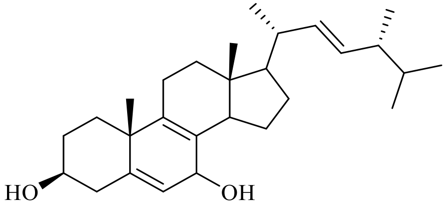	*Acremonium sordidulum*	Steroid	LXRα antagonist	#	#	
**131**	Cycloeucalenone	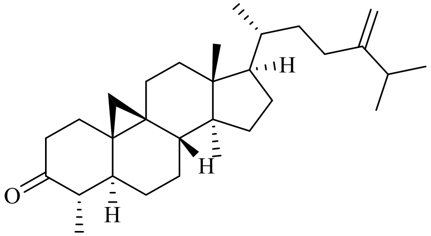	Coelomycete JP4567	Steroid	LXRα antagonist	-8.379	-	
**132**	Diosgenin	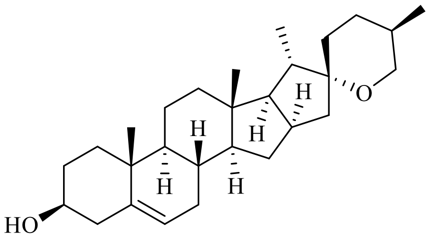	*Dioscorea villosa* L., *Rhizoma Dioscorea Nipponicae*, *Trigonella foenum-graecum* L.	Steroid	Inhibit LXRα	-8.696	-	
**133**	Curcumin	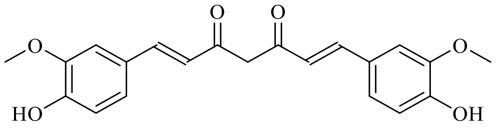	*Curcuma longa* L.	Phenol	Inhibit LXRα protein expression	-5.764	-8.894	

No.	Name	Natural product	Source	Classification	Activation/ Inhibition	S values of 1UHL (LXRα)	S values of 3IPQ (LXRα)	S values of 1P8D (LXRβ)
**134**	Meso-dihydroguaiaretic acid (MDGA)	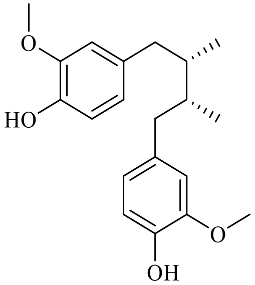	*Machilus thunbergia* Siebold & Zucc.	Lignin Phenylpropanoid	LXRα antagonist	-6.963	-8.285	
**135**	Sesamin	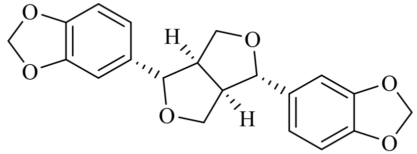	*Sesamum indicum* L.	Lignan Phenylpropanoid	LXRα antagonist	-8.013	-9.764	
**136**	Sauchinone	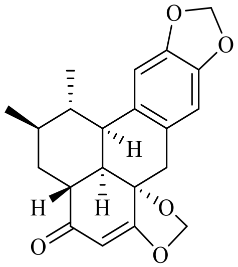	*Saururus chinensis* (Lour.) Baill.	Lignan Phenylpropanoid	Inhibit LXRα	-6.047	-8.199	
**137**	Piperine	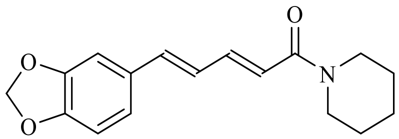	*Piper longum* L.	Alkaloid	Inhibit LXRα/β expression	-8.305	-8.919	-8.204

No.	Name	Natural product	Source	Classification	Activation/ Inhibition	S values of 1UHL (LXRα)	S values of 3IPQ (LXRα)	S values of 1P8D (LXRβ)
**138**	Riccardin F	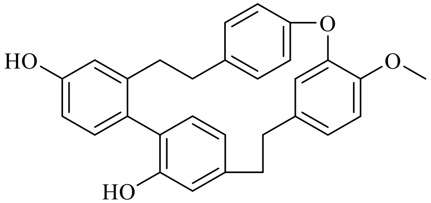	*Blasia pusilla* L.	Non-steroid	LXRα antagonist	-	-	
**139**	Cynandione A	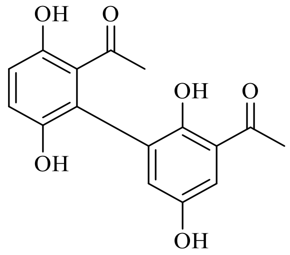	*Cynanchum wilfordii*	Biacetophenone	LXRα antagonist	-7.771	-8.059	
**140**	Guttiferone I	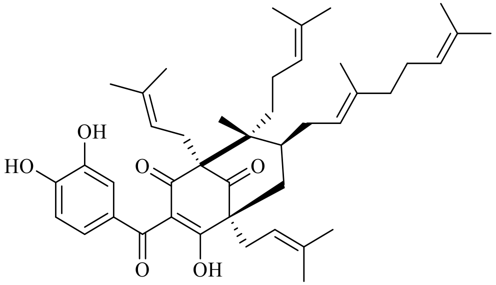	*Garcinia humilis* (Vahl) C.D.Adams	Polyisoprenylated benzophenone	LXRα antagonist	-	-	

No.	Name	Natural product	Source	Classification	Activation/ Inhibition	S values of 1UHL (LXRα)	S values of 3IPQ (LXRα)	S values of 1P8D (LXRβ)
**141**	Rhein	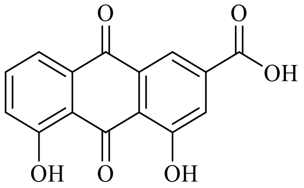	*Rheum palmatum* L.	Anthraquinone	LXRα/β dual antagonist	-7.900	-8.052	-7.227
**142**	Piericidin A (PA)	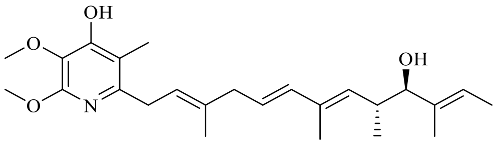	A marine-derived *Streptomyces* strain	Other	LXRα antagonist	-6.505	-9.126	
**143**	Glucopiericidin A (GPA)	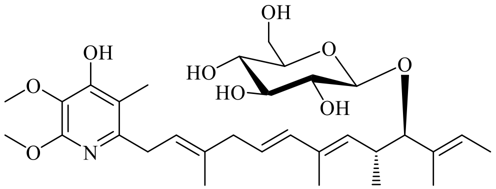	A marine-derived *Streptomyces* strain	Other	LXRα antagonist	-7.895	-	
**144**	10-ketone PA	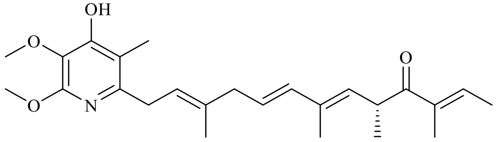	A marine-derived *Streptomyces* strain	Phenol derivative	Inhibit LXRα	-6.878	-8.261	
**145**	(3R)-1,7-diphenyl-(4E,6E)-4,6-heptadien-3-ol	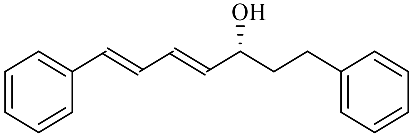	*Curcuma comosa* Roxb.	Phytoestrogen	Downregulate LXRα	-7.810	-8.822	

No.	Name	Natural product	Source	Classification	Activation/ Inhibition	S values of 1UHL (LXRα)	S values of 3IPQ (LXRα)	S values of 1P8D (LXRβ)
	(DPHD)							
**146**	Arachidonic acid	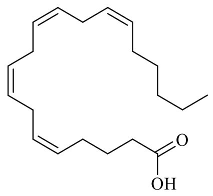	*Acanthopanax koreanum* Nakai.	Non-steroid	LXRα/β dual antagonist and FXR antagonist	-5.144	-7.232	-6.211
**147**	Siphonaxanthin	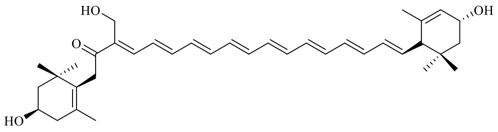	Green algae such as *Codium fragile, Umbraulva japonica,* and *Caulerpa lentillifera*	Carotenoid	LXRα antagonist	-	-	

Supplementary Table 3| Natural agonists targeted FXR.

(#: The docking between ligands with not completely determined configuration and proteins is not done. -: The ligands could not fit into proteins.)

No.	Name	Natural product	Source	Classification	Activation/ Inhibition	Docking score of 1OSV	Docking score of 1OT7	Docking score of 3BEJ
**148**	Farnesol	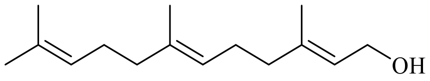	*Anthemis nobilis* L. and *Cananga odorata* (Lam.)	Isoprenoid Terpene	Agonist	-3.411	-4.345	-4.116

No.	Name	Natural product	Source	Classification	Activation/ Inhibition	Docking score of 1OSV	Docking score of 1OT7	Docking score of 3BEJ
			Hook.f. & Thomson					
**149**	Lepidozenolide	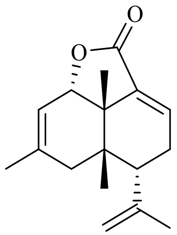	*Lepidozia fauriana*	Terpene	Agonist	-7.371	-7.886	-7.975
**150**	Cafestol	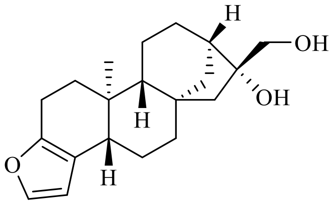	Coffee beans	Terpene	Agonist	-8.473	-8.902	-9.003
**151**	Tschimgine	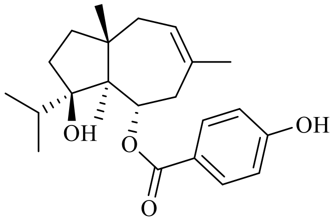	*Ferula ovina* Boiss.	Terpene	Activate FXR	-7.869	-8.567	-8.425

No.	Name	Natural product	Source	Classification	Activation/ Inhibition	Docking score of 1OSV	Docking score of 1OT7	Docking score of 3BEJ
**152**	Dihydroartemisinin	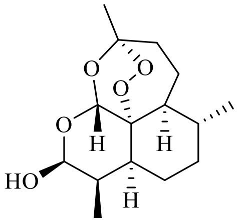	*Artemisia carvifolia* Buch.-Ham. ex Roxb.	Terpene	Activate FXR	-7.748	-7.709	-7.228
**153**	Scrodentoids F	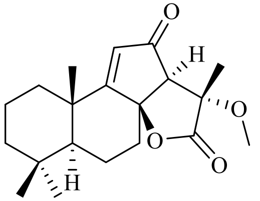	*Scrophularia dentata* Royle ex Benth.	Terpene	Activate FXR	-9.278	-9.169	-9.142
**154**	Picroside II	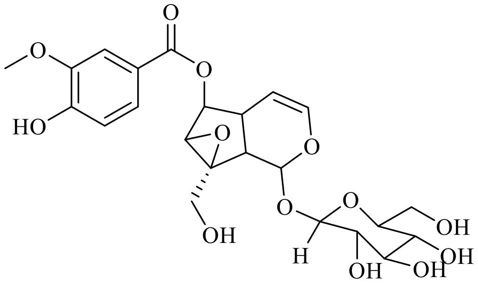	*Picrorhiza scrophulariiflora* Pennell	Terpene	Activate FXR	#	#	#

No.	Name	Natural product	Source	Classification	Activation/ Inhibition	Docking score of 1OSV	Docking score of 1OT7	Docking score of 3BEJ
**155**	Geniposide	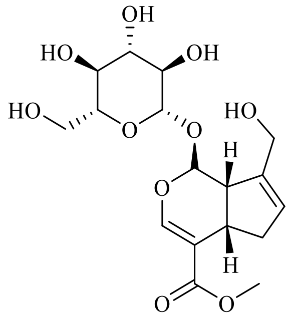	*Gardenia jasminoides* Ellis	Terpene	Activate FXR	-7.211	-6.862	-6.237
**156**	Betulinic acid	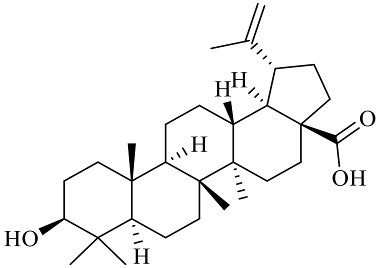	*Betula* species	Terpene	Activate FXR	-7.630	-7.846	-
**157**	Oleanolic acid	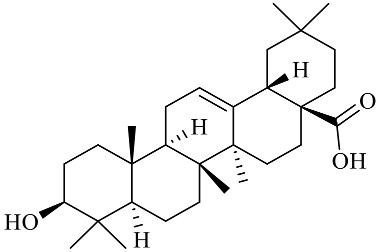	*Olea europaea* L.	Terpene	A selective FXR modulator	-	-	-

No.	Name	Natural product	Source	Classification	Activation/ Inhibition	Docking score of 1OSV	Docking score of 1OT7	Docking score of 3BEJ
**158**	Hedragonic acid	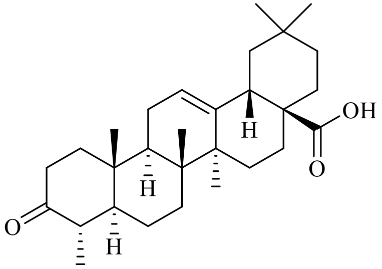	*Celastrus orbicalatus* Thunb.	Terpene	Agonist	-	-	-
**159**	Ergosterol peroxide	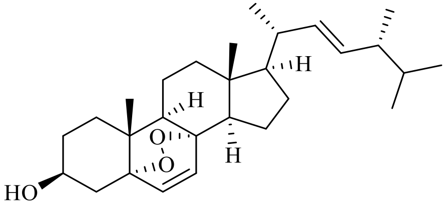	*Ganoderma lucidum* Karst.	Steroid	Agonist	-7.890	-8.017	-9.519
**160**	Ganoderiol F	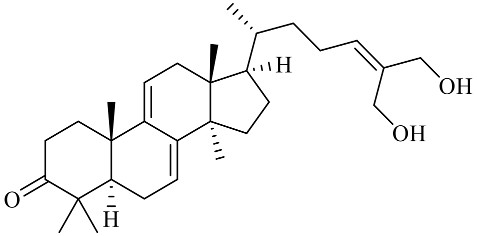	*Ganoderma lucidum* Karst.	Terpene	Agonist	-8.767	-8.396	-9.03

No.	Name	Natural product	Source	Classification	Activation/ Inhibition	Docking score of 1OSV	Docking score of 1OT7	Docking score of 3BEJ
**161**	Ganodermanontriol	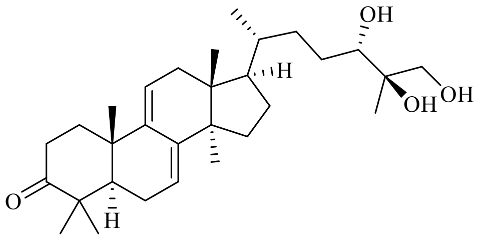	*Ganoderma lucidum* Karst.	Terpene	Agonist	-8.795	-8.509	-9.349
**162**	Lucidumol A	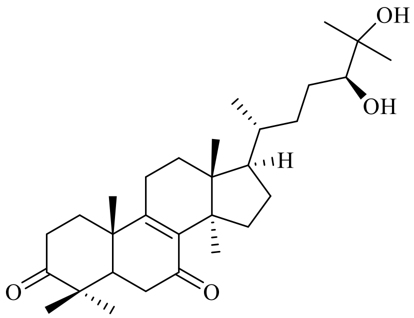	*Ganoderma lucidum* Karst.	Terpene	Agonist	-	-8.667	-
**163**	Ganoderic acid TR	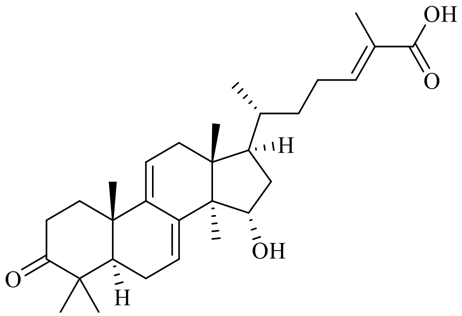	*Ganoderma lucidum* Karst.	Terpene	Agonist	-7.948	-9.078	-

No.	Name	Natural product	Source	Classification	Activation/ Inhibition	Docking score of 1OSV	Docking score of 1OT7	Docking score of 3BEJ
**164**	Cycloastragenol (CAG)	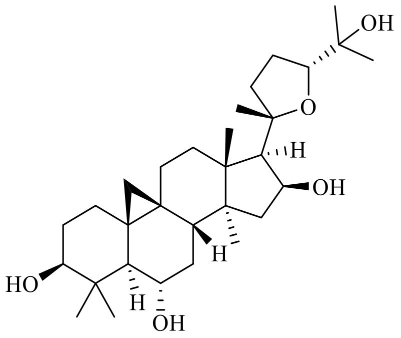	*Astragalus membranaceus* (Fisch.) Bunge	Terpene	Activate FXR	-	-	-
**165**	Alisol M 23-acetate	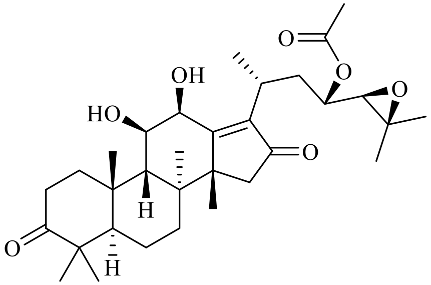	*Alisma orientalis* (Sam.) Juzep.	Terpene	Agonist	-	-	-
**166**	Alisol A 23-acetate	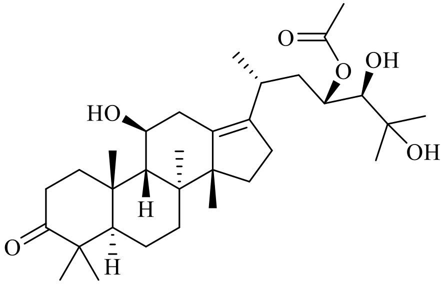	*Alisma orientalis* (Sam.) Juzep.	Terpene	Agonist	-7.928	-	-

No.	Name	Natural product	Source	Classification	Activation/ Inhibition	Docking score of 1OSV	Docking score of 1OT7	Docking score of 3BEJ
**167**	Alisol B 23-acetate	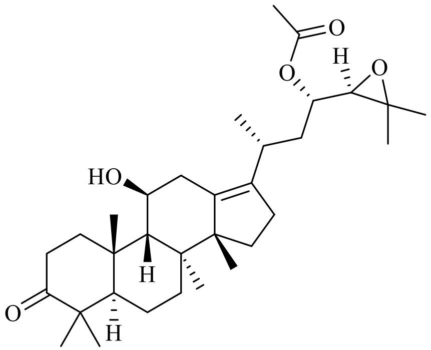	*Rhizoma alismatis* and *Alisma orientale* (Sam.) Juzep.	Terpene	Agonist	-7.928	-10.218	-
**168**	Aisol F	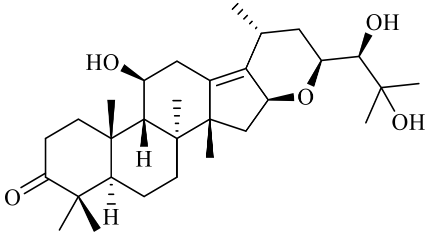	*Alisma orientale* (Sam.) Juzep.	Terpene	Agonist	-	-10.937	-
**169**	Aisol A	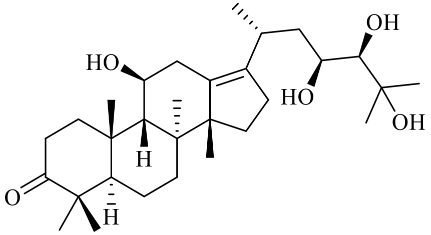	*Alisma orientale* (Sam.) Juzep.	Terpene	Agonist	-	-8.390	-

No.	Name	Natural product	Source	Classification	Activation/ Inhibition	Docking score of 1OSV	Docking score of 1OT7	Docking score of 3BEJ
**170**	25-anhydro alisol A	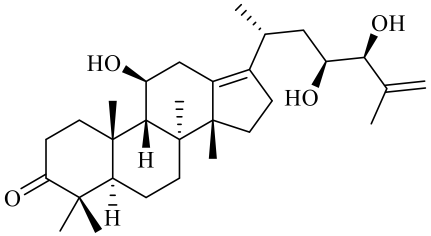	*Alisma orientale* (Sam.) Juzep.	Terpene	Agonist	-8.205	-8.322	-
**171**	(23S)-11β,23-dihydroxy-8α,9β,14β-dammar-13 (17)-ene-3,24-dione	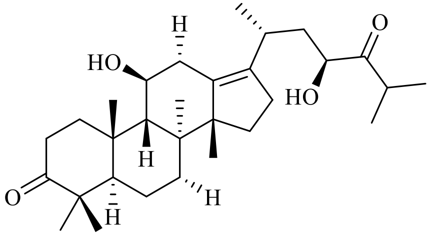	*Alisma orientale* (Sam.) Juzep.	Terpene	Agonist	-9.466	-9.461	-
**172**	Arjunolic acid	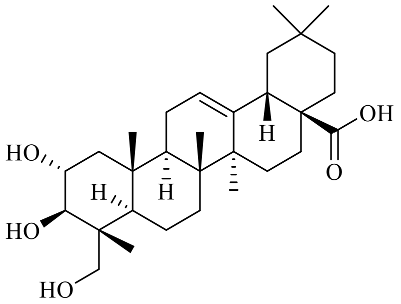	*Terminalia arjuna* W. & Arn.	Terpene	Upregulate FXR expression	-7.443	-	-8.839

No.	Name	Natural product	Source	Classification	Activation/ Inhibition	Docking score of 1OSV	Docking score of 1OT7	Docking score of 3BEJ
**173**	Xylocarpols E	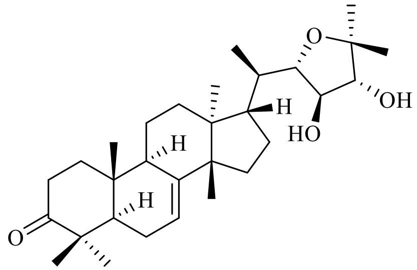	Mangrove plants *Xylocarpus granatum* J.Koenig, *Xylocarpus moluccensis* (Lam.) M.Roem., and *Excoecaria agallocha* L.	Terpene	Agonist	-8.684	-	-
**174**	Agallochols A	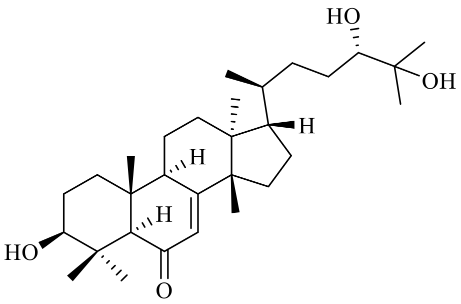	Mangrove plants *Xylocarpus granatum* J.Koenig, *Xylocarpus moluccensis* (Lam.) M.Roem., and *Excoecaria agallocha* L.	Terpene	Agonist	-9.395	-9.911	-

No.	Name	Natural product	Source	Classification	Activation/ Inhibition	Docking score of 1OSV	Docking score of 1OT7	Docking score of 3BEJ
**175**	Agallochols B	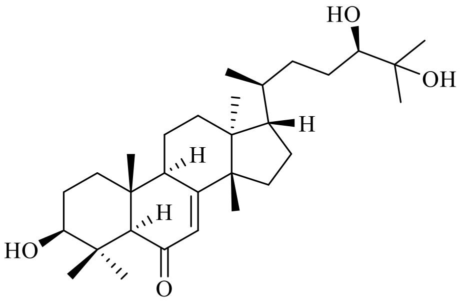	Mangrove plants *Xylocarpus granatum* J.Koenig, *Xylocarpus moluccensis* (Lam.) M.Roem., and *Excoecaria agallocha* L.	Terpene	Agonist	-9.243	-9.448	-
**176**	Agallochols D	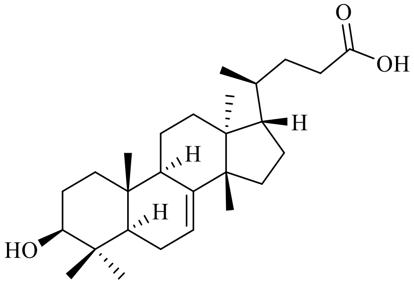	Mangrove plants *Xylocarpus granatum* J.Koenig, *Xylocarpus moluccensis* (Lam.) M.Roem., and *Excoecaria agallocha* L.	Terpene	Agonist	-9.045	-9.465	-9.125

No.	Name	Natural product	Source	Classification	Activation/ Inhibition	Docking score of 1OSV	Docking score of 1OT7	Docking score of 3BEJ
**177**	Dioscin	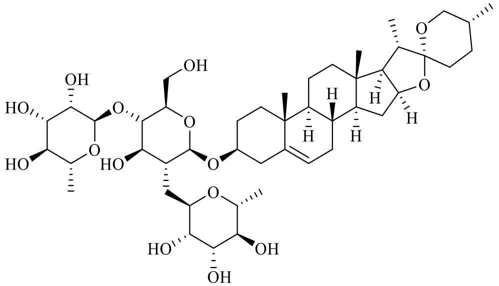	*Dioscoreae rhizoma*	Steroid	Increase FXR expression	-	-	-
**178**	Calycosin	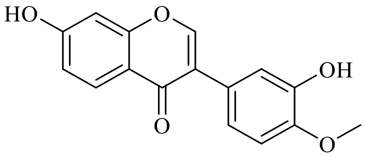	*Radix Astragali*	Flavonoid	Agonist	-8.408	-8.257	-7.318
**179**	Cryptochinones A	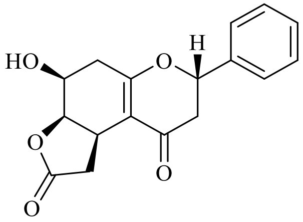	*Cryptocarya chinensis* (Hance) Hemsl.	Tetrahydroflavanone Flavonoid	Agonist	-9.512	-9.898	-8.876
**180**	Cryptochinones B	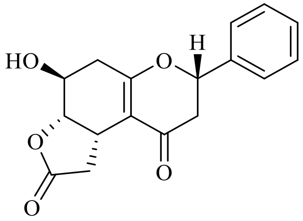	*Cryptocarya chinensis* (Hance) Hemsl.	Tetrahydroflavanone Flavonoid	Agonist	-8.836	-9.186	-8.714

No.	Name	Natural product	Source	Classification	Activation/ Inhibition	Docking score of 1OSV	Docking score of 1OT7	Docking score of 3BEJ
**181**	Cryptochinones C	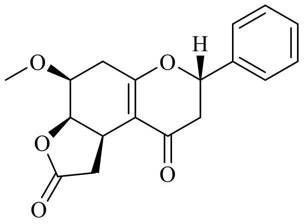	*Cryptocarya chinensis* (Hance) Hemsl.	Tetrahydroflavanone Flavonoid	Agonist	-9.547	-9.201	-8.106
**182**	Cryptochinones D	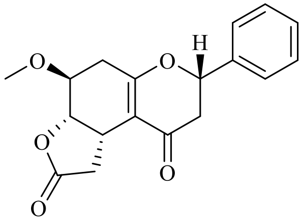	*Cryptocarya chinensis* (Hance) Hemsl.	Tetrahydroflavanone Flavonoid	Agonist	-8.764	-9.287	-8.885
**183**	Hesperidin	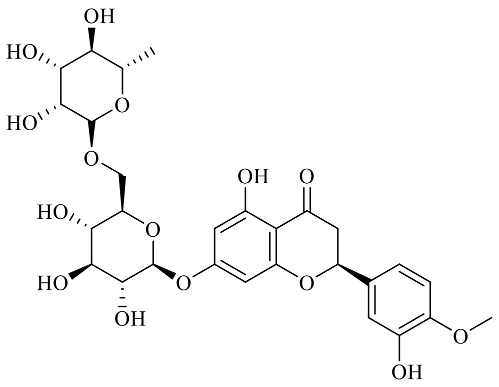	Lemons and oranges	Favonoid	Agonist	-	-	-

No.	Name	Natural product	Source	Classification	Activation/ Inhibition	Docking score of 1OSV	Docking score of 1OT7	Docking score of 3BEJ
**184**	Schaftoside	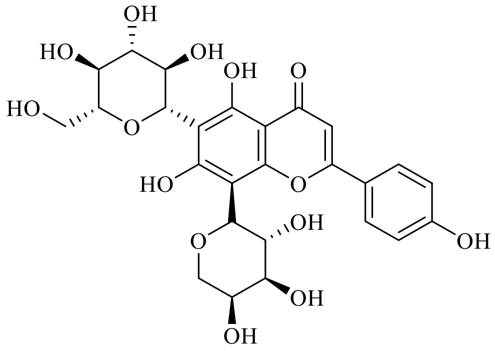	*Desmodium styracif olium*	Flavonoid	Agonist	-	--	-
**185**	Epigallocatechin-3-gallate (EGCG)	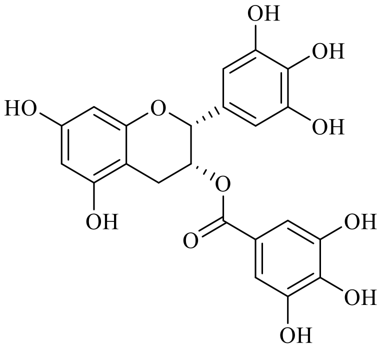	*Camellia sinensis* (L.) Kuntze	Flavonoid	SBARM	-6.459	-	-

No.	Name	Natural product	Source	Classification	Activation/ Inhibition	Docking score of 1OSV	Docking score of 1OT7	Docking score of 3BEJ
**186**	Pinocembrin-7-O-[2”-O-galloyl-4”, 6”-hexahydroxydiphenoyl]-β-D-glucose	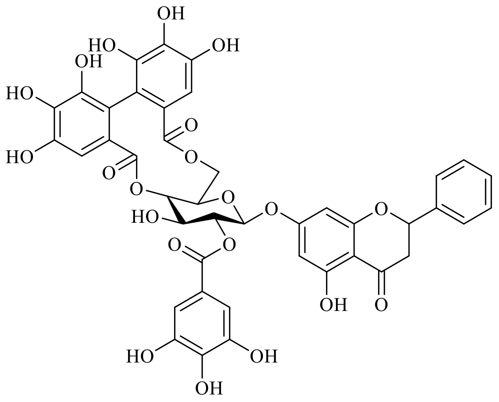	*Penthorum chinense* Pursh	Flavonoid	Activate FXR	#	#	#
**187**	2’,6’-dihydroxydihydrochalcone-4’-O-[2’’-O-galloyl-4’’,6’’-hexahydroxydiphenoyl]-β-D-glucopyranoside	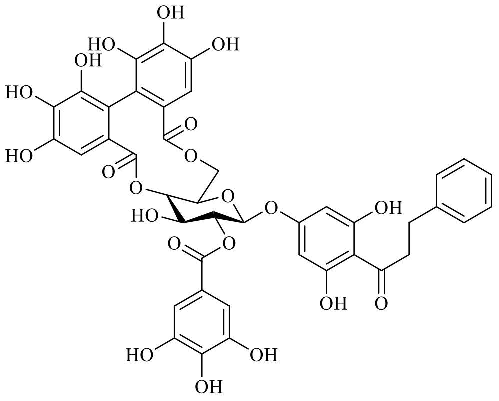	*Penthorum chinense* Pursh	Flavonoid	Activate FXR	-	-	-

No.	Name	Natural product	Source	Classification	Activation/ Inhibition	Docking score of 1OSV	Docking score of 1OT7	Docking score of 3BEJ
**188**	Grifolin	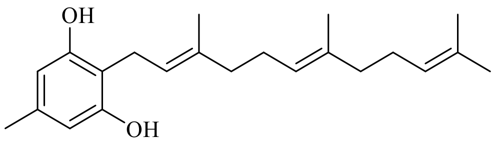	Mushroom and *Ginkgo biloba* L.	Non-steroid	Agonist	-7.482	-8.556	-8.32
**189**	Ginkgolic acid 15:1	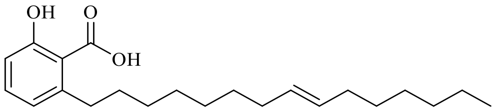	Mushroom and *Ginkgo biloba* L.	Non-steroid	Agonist	-7.793	-7.821	-7.945
**190**	Geranyl caffeate	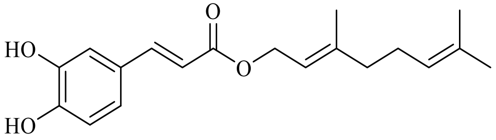	Himalayan poplar	Non-steroid	Agonist	-7.694	-7.147	-7.052
**191**	Marchantin A	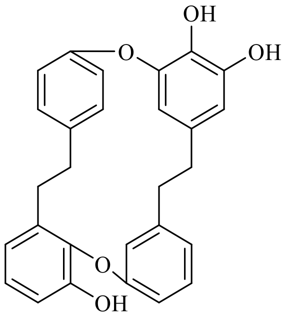	*Marchantia paleacea* Bertol.	Non-steroid	Agonist	-	-	-

No.	Name	Natural product	Source	Classification	Activation/ Inhibition	Docking score of 1OSV	Docking score of 1OT7	Docking score of 3BEJ
**192**	Marchantin E	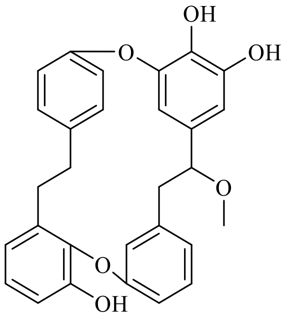	*Marchantia paleacea* Bertol.	Non-steroid	Agonist	-	-	-
**193**	Altenusin	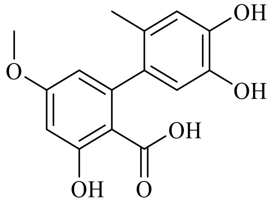	*Alternaria sp.*	Non-steroid	Agonist	-7.231	-7.342	-8.522
**194**	Papaverine	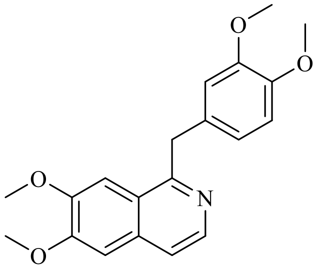	*Papaver somniferum* L.	Alkaloid	Agonist	-7.950	-8.028	-8.316

No.	Name	Natural product	Source	Classification	Activation/ Inhibition	Docking score of 1OSV	Docking score of 1OT7	Docking score of 3BEJ
**195**	Berberine	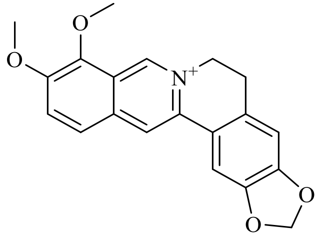	*Coptis chinensi*	Alkaloid	Agonist	-8.731	-9.500	-8.528
**196**	Nelumol A	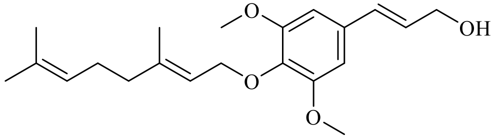	*Ligularia nelumbifolia* Hand. Mazz.	Phenylpropanoid	Agonist	-6.629	-8.077	-8.751
**197**	Nelumal A	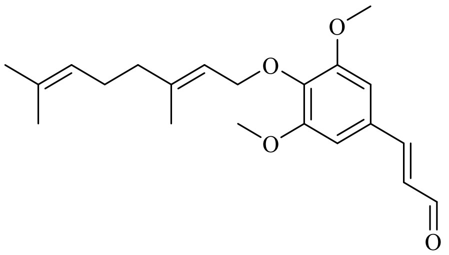	*Ligularia nelumbifolia* Hand. Mazz.	Phenylpropanoid	Agonist	-7.141	-7.613	-8.236
**198**	Auraptene (AUR)	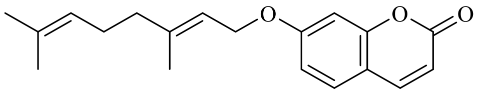	*Citrus aurantium*	Coumarin Phenylpropanoid	Agonist	-7.464	-7.681	-8.194

No.	Name	Natural product	Source	Classification	Activation/ Inhibition	Docking score of 1OSV	Docking score of 1OT7	Docking score of 3BEJ
**199**	Podophyllotoxin	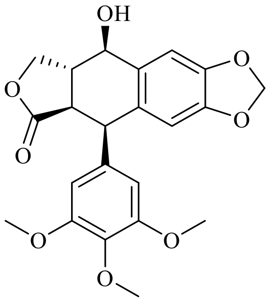	*Podophyllum* species	Lignan Phenylpropanoid	Agonist	-	-3.895	-
**200**	Herpetotriol	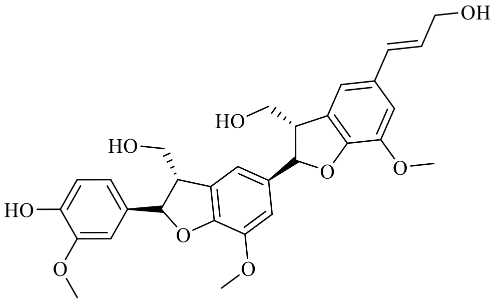	*Herpetospermum pedunculosum* (Ser.) C.B. Clarke	Lignan Phenylpropanoid	Agonist	-	-	-

No.	Name	Natural product	Source	Classification	Activation/ Inhibition	Docking score of 1OSV	Docking score of 1OT7	Docking score of 3BEJ
**201**	Spathulatd	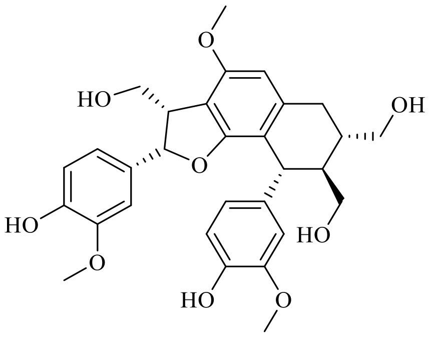	*Herpetospermum pedunculosum* (Ser.) C.B. Clarke	Lignan Phenylpropanoid	Agonist	-	-	-
**202**	Lariciresinol	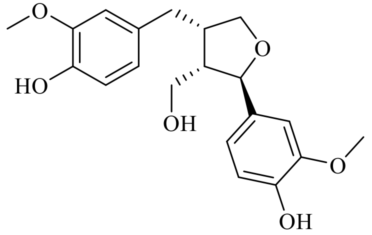	*Herpetospermum pedunculosum* (Ser.) C.B. Clarke	Lignan Phenylpropanoid	Agonist	-8.284	-8.320	-9.127
**203**	Dehydrodiconiferyl alcohol	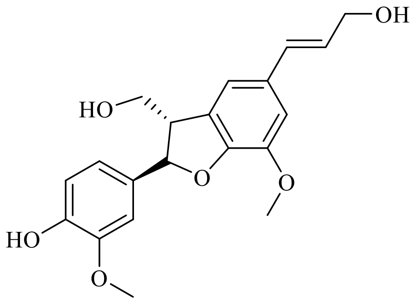	*Herpetospermum pedunculosum* (Ser.) C.B. Clarke	Lignan Phenylpropanoid	Agonist	-8.442	-8.282	-9.263

No.	Name	Natural product	Source	Classification	Activation/ Inhibition	Docking score of 1OSV	Docking score of 1OT7	Docking score of 3BEJ
**204**	Herpetrione	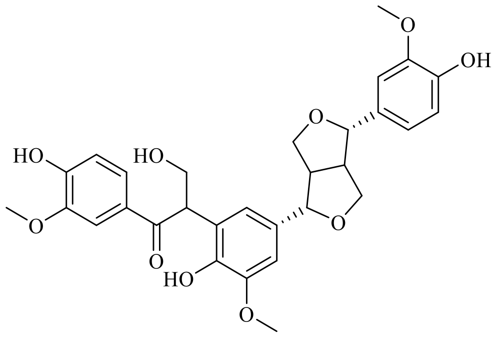	*Herpetospermum pedunculosum* (Ser.) C.B. Clarke	Lignan Phenylpropanoid	Agonist	#	#	#
**205**	Dehydrodiconiferyl alcohol	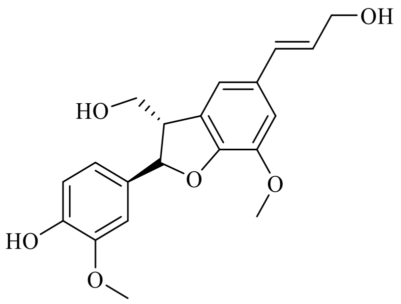	*Herpetospermum pedunculosum* (Ser.) C.B. Clarke	Lignan Phenylpropanoid	Agonist	-8.519	-	-9.028
**206**	Coumestrol	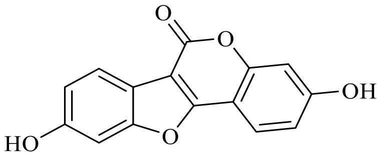	Red clover and soybeans	Phytoestrogens Steroid	Agonist	-8.042	-8.059	-8.181

No.	Name	Natural product	Source	Classification	Activation/ Inhibition	Docking score of 1OSV	Docking score of 1OT7	Docking score of 3BEJ
**207**	Yangonin	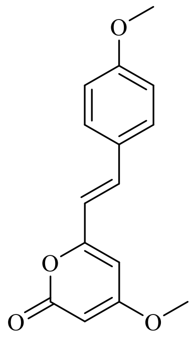	Kava *Piper methysticum*	Kavalactone	Activate FXR	-7.027	-7.056	-7.232
**208**	Monascin	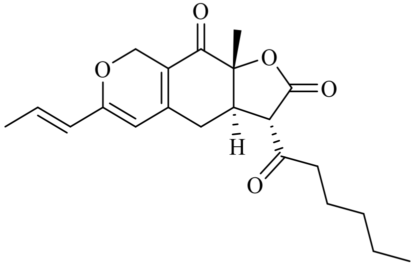	*Monascus*-fermented rice	Other	Upregulate FXR	-7.657	-6.766	-7.720

No.	Name	Natural product	Source	Classification	Activation/ Inhibition	Docking score of 1OSV	Docking score of 1OT7	Docking score of 3BEJ
**209**	Ankaflavin	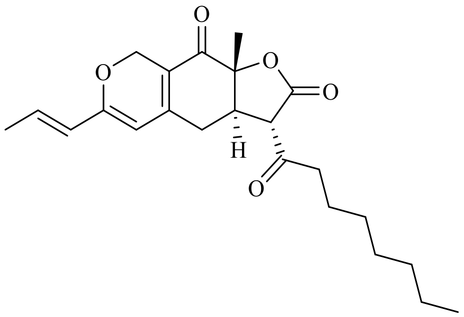	*Monascus*-fermented rice	Other	Upregulate FXR	-8.036	-8.280	-8.094
**1**	Geraniol	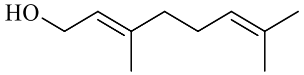	Essential oils of aromatic plants including *Cinnamomum tenuipilum* Kosterm (Lauraceae), *Valeriana officinalis* L. (Caprifoliaceae) and n-butanol extract (NE3) of *Panax notoginseng* (Burk.) F.H. Chen	Terpene	LXRα/FXR dual agonist	-	-	-

No.	Name	Natural product	Source	Classification	Activation/ Inhibition	Docking score of 1OSV	Docking score of 1OT7	Docking score of 3BEJ
**20**	Tirotundin	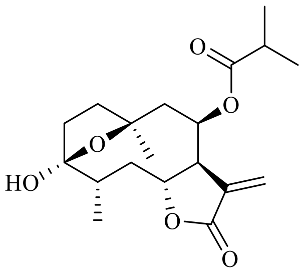	*Tithonia diversifolia* (Hemsl.) A. Gray	Terpene	LXRα/β and FXR dual agonists	-8.401	-9.231	-8.015
**21**	Tagitinin A	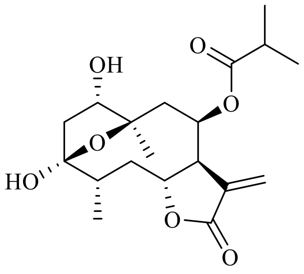	*Tithonia diversifolia* (Hemsl.) A. Gray	Terpene	LXRα/β and FXR dual agonists	-8.346	-8.044	-7.741

No.	Name	Natural product	Source	Classification	Activation/ Inhibition	Docking score of 1OSV	Docking score of 1OT7	Docking score of 3BEJ
**29**	Ginsenoside Rg_1	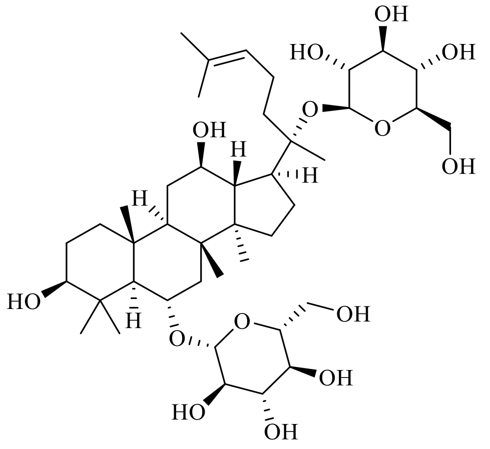	*Panax ginseng* C.A.Mey.	Terpene	Increase LXRα mRNA level and FXR agonist	-	-	-
**84**	Danshensu	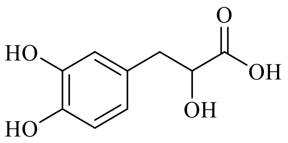	*Salvia miltiorrhiza* Bunge	Phenol	LXRα/FXR dual agonist	-	-	-
**85**	Rosmarinic acid	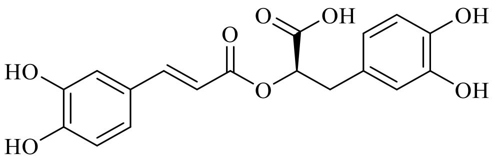	*Salvia miltiorrhiza* Bunge	Phenol	LXRα/FXR dual agonist	-	-	-

No.	Name	Natural product	Source	Classification	Activation/ Inhibition	Docking score of 1OSV	Docking score of 1OT7	Docking score of 3BEJ
**86**	Salvianolic acid A	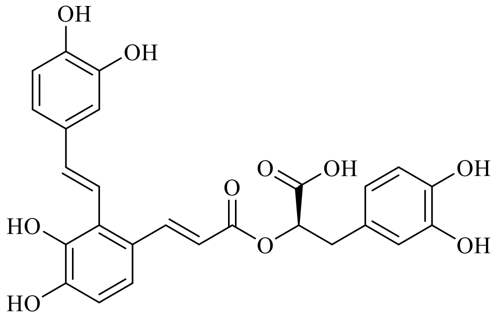	*Salvia miltiorrhiza* Bunge	Phenol	LXRα/FXR dual agonist	-	-	-
**87**	Salvianolic acid B	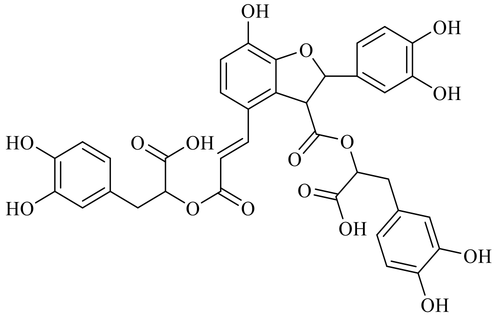	*Salvia miltiorrhiza* Bunge	Phenol	LXRα/FXR dual agonist	-	-	-
**114**	Ursolic acid	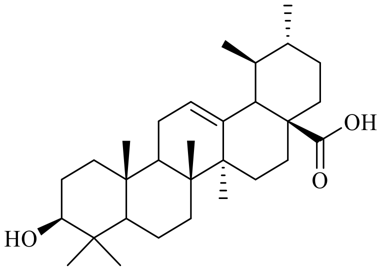	*Cornus officinalis* Siebold & Zucc. and other plants	Terpene	LXRα antagonist and activate FXR	-	-	-

No.	Name	Natural product	Source	Classification	Activation/ Inhibition	Docking score of 1OSV	Docking score of 1OT7	Docking score of 3BEJ
**124**	Xanthohumol	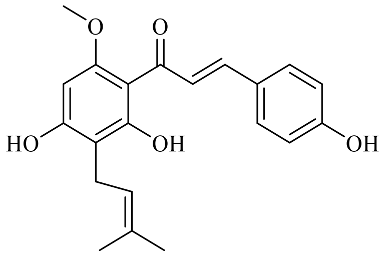	*Humulus lupulus* L.	Flavonoid	Counteract LXRα activation and a selective bile acid receptor modulator (SBARM)	-	-	-

Supplementary Table 4| Natural antagonists targeted FXR.

(#: The docking between ligands with not completely determined configuration and proteins is not done. -: The ligands could not fit into proteins.)

No.	Name	Natural product	Source	Classification	Activation/ Inhibition	Docking score of 1OSV	Docking score of 1OT7	Docking score of 3BEJ
**210**	Atractylenolide II	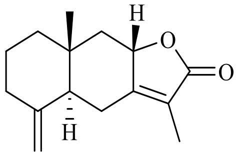	*Atractylodes macrocephala* Koidz.	Terpene	Antagonist	-7.791	-7.745	-7.885

No.	Name	Natural product	Source	Classification	Activation/ Inhibition	Docking score of 1OSV	Docking score of 1OT7	Docking score of 3BEJ
**211**	Atractylenolide III	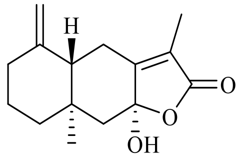	*Atractylodes macrocephala* Koidz.	Terpene	Antagonist	-7.507	-7.818	-8.338
**212**	Andrographolide	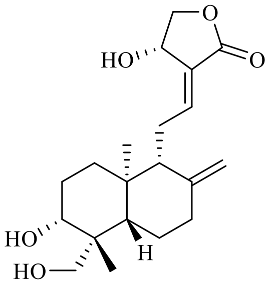	*Andrographis panniculata* (Burm.f.) Nees	Terpene	Antagonist	-8.515	-8.992	-8.153
**213**	6β-hydroxynigranoic acid	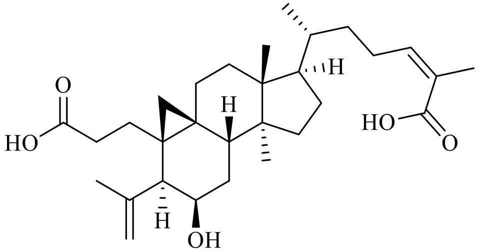	*Schisandra glaucescens* Diels	Terpene	Antagonist	-7.515	-7.934	-7.617

No.	Name	Natural product	Source	Classification	Activation/ Inhibition	Docking score of 1OSV	Docking score of 1OT7	Docking score of 3BEJ
**214**	Schiglausin N	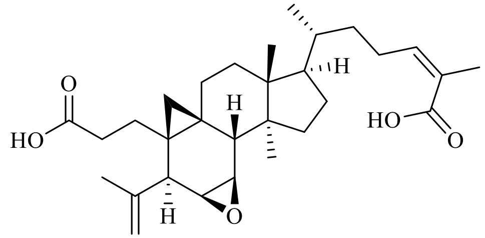	*Schisandra glaucescens* Diels	Terpene	Antagonist	-7.930	-9.525	-8.218
**215**	Schiglausin D	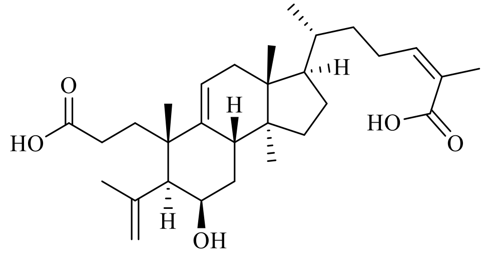	*Schisandra glaucescens*	Terpene	Antagonist	-7.824	-8.743	-8.494
**216**	Schiglausin E	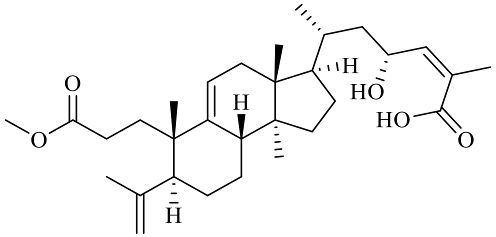	*Schisandra glaucescens* Diels	Terpene	Antagonist	-9.459	-8.826	-

No.	Name	Natural product	Source	Classification	Activation/ Inhibition	Docking score of 1OSV	Docking score of 1OT7	Docking score of 3BEJ
**217**	Schiglausin F	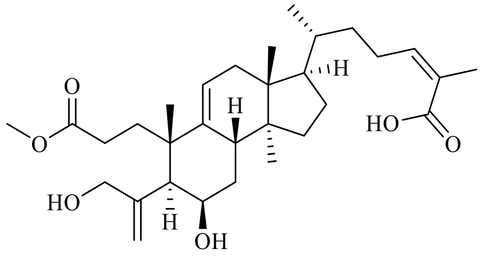	*Schisandra glaucescens* Diels	Terpene	Antagonist	-8.015	-	-8.025
**218**	Schiglausin G	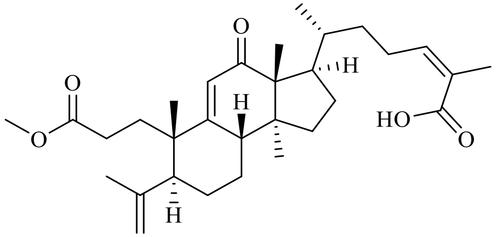	*Schisandra glaucescens* Diels	Terpene	Antagonist	-9.009	-10.399	-8.409
**219**	Kadsuric acid	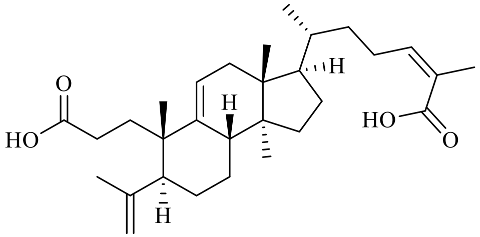	*Schisandra glaucescens* Diels	Terpene	Antagonist	-9.422	-9.880	-9.623

No.	Name	Natural product	Source	Classification	Activation/ Inhibition	Docking score of 1OSV	Docking score of 1OT7	Docking score of 3BEJ
**220**	3β-hydroxy-lanost-9(11),24-dien-26-oic acid	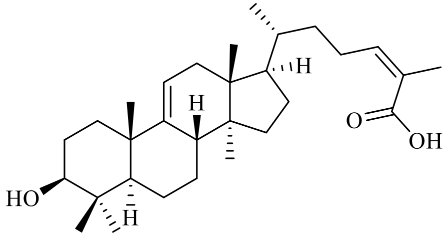	*Schisandra glaucescens* Diels	Terpene	Antagonist	-9.072	-8.806	-7.783
**221**	Suvanine	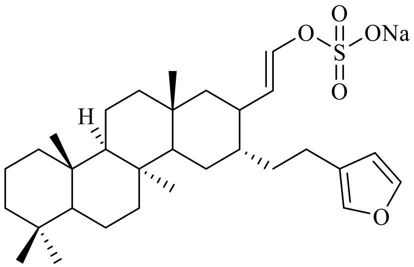	*Coscinoder mamathewsi*	Terpene	Antagonist	#	#	#
**222**	12-*O*-deacetyl-12-*epi*-19-deoxy-21-hydroxyscalarin	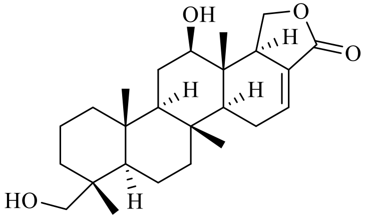	*Spongia* sp.	Terpene	Antagonist	-8.819	-8.561	-9.642

No.	Name	Natural product	Source	Classification	Activation/ Inhibition	Docking score of 1OSV	Docking score of 1OT7	Docking score of 3BEJ
**223**	12-*O*-deacetyl-12-*epi*-19-deoxy-22-hydroxyscalarin	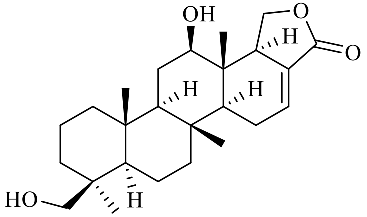	*Spongia* sp.	Terpene	Antagonist	-8.941	-8.672	-9.588
**224**	12-*O*-deacetyl-12-*epi*-19-*O*-methylscalarin	**221** 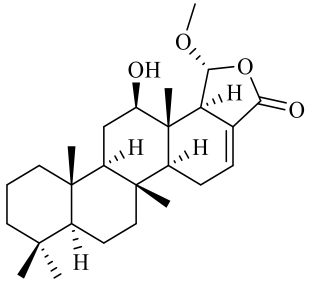	*Spongia* sp.	Terpene	Antagonist	-8.032	-8.614	-9.381
**225**	12-*O*-deacetyl-12-*epi*-scalarin	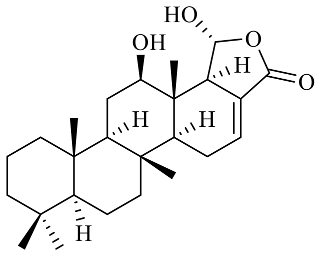	*Spongia* sp.	Terpene	Antagonist	-9.260	-9.352	-9.677

No.	Name	Natural product	Source	Classification	Activation/ Inhibition	Docking score of 1OSV	Docking score of 1OT7	Docking score of 3BEJ
**226**	12-*epi*-scalarin	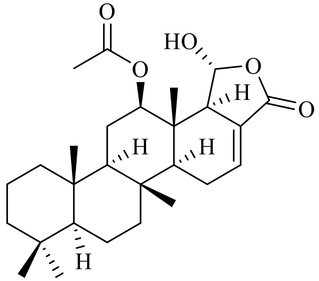	*Spongia* sp.	Terpene	Antagonist	-9.471	-8.771	
**227**	12-*O*-deacetyl-12-*epi*-19-deoxyscalarin	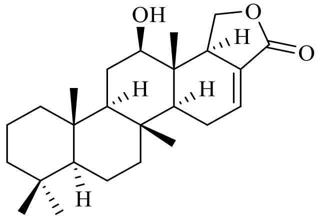	*Spongia* sp.	Terpene	Antagonist	-9.275	-9.152	-10.055
**228**	Tuberatolide A	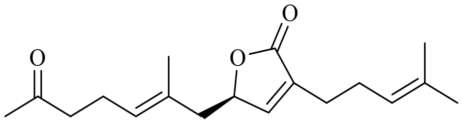	*Botryllus tuberatus*	Terpene	Antagonist	-5.849	-5.899	-5.861
**229**	Tuberatolide B	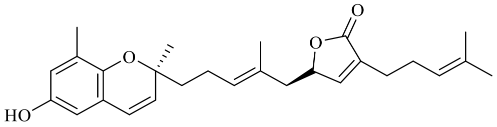	*Botryllus tuberatus*	Terpene	Antagonist	-8.660	-8.759	-10.113

No.	Name	Natural product	Source	Classification	Activation/ Inhibition	Docking score of 1OSV	Docking score of 1OT7	Docking score of 3BEJ
**230**	2'-*epi*-tuberatolide B	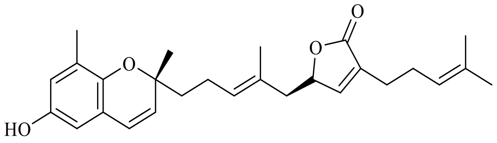	*Botryllus tuberatus*	Terpene	Antagonist	-8.660	-8.759	-10.113
**231**	Yezoquinolide	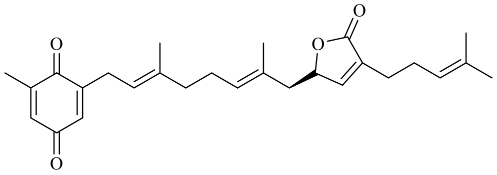	*Botryllus tuberatus*	Terpene	Antagonist	-8.416	-7.906	-8.894
**232**	(R)-sargachromenol	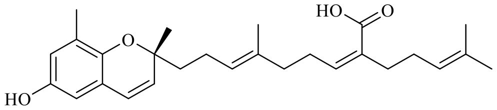	*Botryllus tuberatus*	Terpene	Antagonist	-8.616	-8.812	-9.357
**233**	(S)-sargachromenol	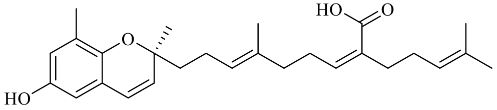	*Botryllus tuberatus*	Terpene	Antagonist	-9.179	-8.801	-11.025
**234**	E-guggulsterone	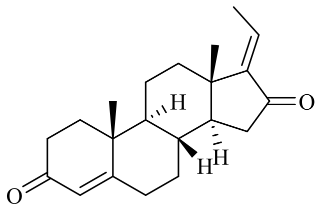	*Commiphora mukul* (Arn.) Bhandari	Steroid	Antagonist	-10.147	-10.154	-9.639

No.	Name	Natural product	Source	Classification	Activation/ Inhibition	Docking score of 1OSV	Docking score of 1OT7	Docking score of 3BEJ
**235**	Z-guggulsterone	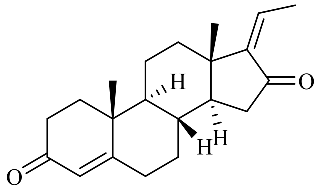	*Commiphora mukul* (Arn.) Bhandari	Steroid	Antagonist	-10.457	-10.146	-9.310
**236**	Stigmasterol acetate	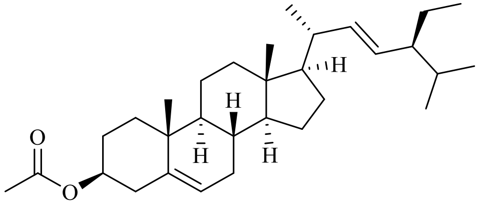	Soybeans	Phytoestrogens Steroid	Antagonist	-7.117	-6.439	-8.446
**237**	Ergostane-3,4,21,26-tetrol, 3,21-bis (hydrogen sulfate), (3α,4α,5β,25S)	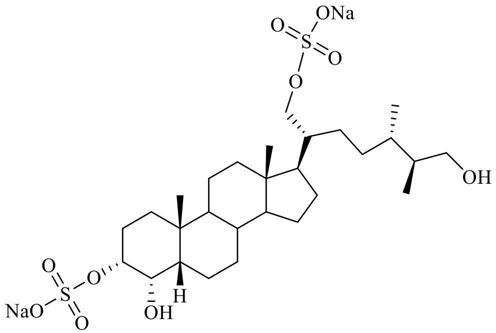	*Ophiolepis superba*	Steroid	Antagonist	-	-	-

No.	Name	Natural product	Source	Classification	Activation/ Inhibition	Docking score of 1OSV	Docking score of 1OT7	Docking score of 3BEJ
**238**	Swinhosterol B	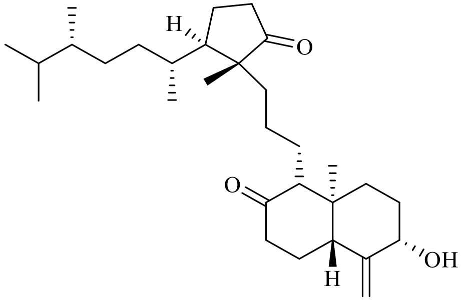	*Theonella swinhoei*	Steroid	Antagonist	-8.818	-7.703	-9.115
**239**	Conicasterol I	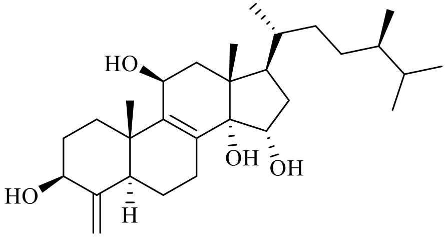	*Theonella swinhoei*	Steroid	Antagonist	-10.917	-8.495	-8.331
**240**	Conicasterol J	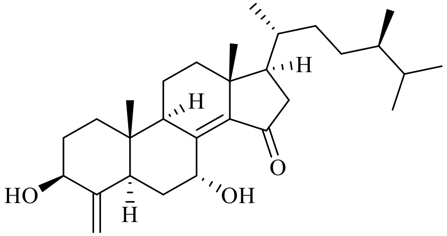	*Theonella swinhoei*	Steroid	Antagonist	-8.897	-8.075	-8.318

No.	Name	Natural product	Source	Classification	Activation/ Inhibition	Docking score of 1OSV	Docking score of 1OT7	Docking score of 3BEJ
**241**	Conicasterol	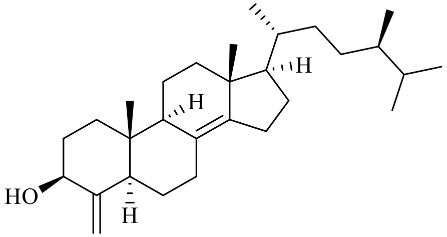	*Theonella swinhoei*	Steroid	Antagonist	-7.841	-8.047	-9.332
**242**	Theonellasterol	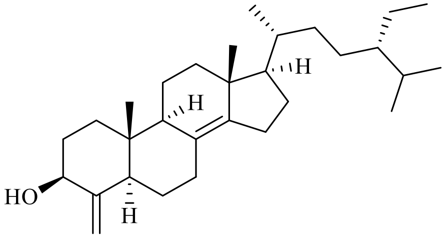	*Theonella swinhoei*	Steroid	Antagonist	-8.345	-8.337	-9.669
**243**	Conicasterol B	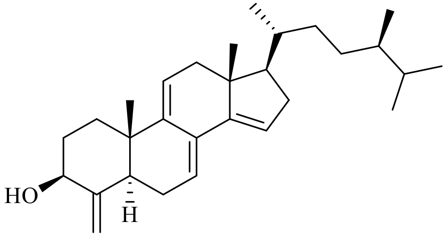	*Theonella swinhoei*	Steroid	Antagonist	-9.041	-8.606	-9.871

No.	Name	Natural product	Source	Classification	Activation/ Inhibition	Docking score of 1OSV	Docking score of 1OT7	Docking score of 3BEJ
**244**	Conicasterol C	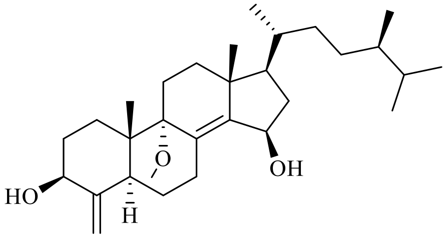	*Theonella swinhoei*	Steroid	Antagonist	-6.980	-	-10.379
**245**	Conicasterol D	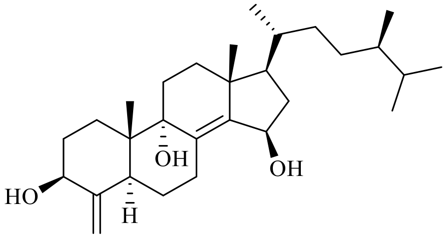	*Theonella swinhoei*	Steroid	Antagonist	-8.695	-7.977	-10.002
**246**	Theonellasterol B	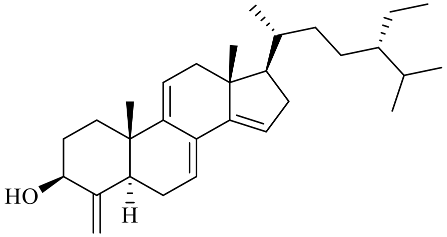	*Theonella swinhoei*	Steroid	Antagonist	-8.900	-8.637	-8.968

No.	Name	Natural product	Source	Classification	Activation/ Inhibition	Docking score of 1OSV	Docking score of 1OT7	Docking score of 3BEJ
**247**	Theonellasterol C	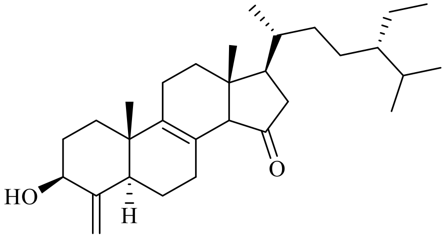 	*Theonella swinhoei*	Steroid	Antagonist	#	#	#
**248**	Theonellasterol E	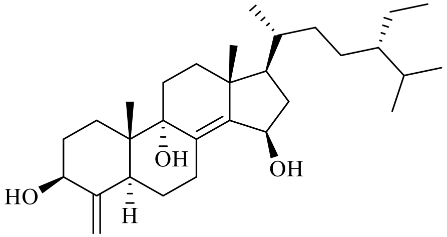 	*Theonella swinhoei*	Steroid	Antagonist	-8.332	-7.887	-9.907
**249**	Theonellasterol F	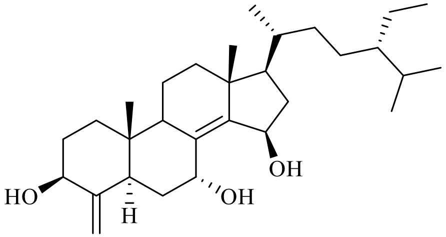 	*Theonella swinhoei*	Steroid	Antagonist	#	#	#

No.	Name	Natural product	Source	Classification	Activation/ Inhibition	Docking score of 1OSV	Docking score of 1OT7	Docking score of 3BEJ
**250**	Theonellasterol G	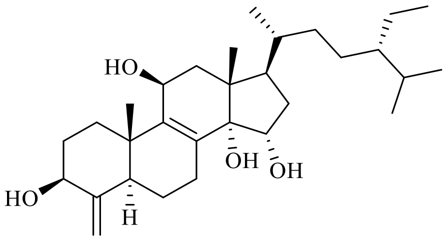	*Theonella swinhoei*	Steroid	A selective bile acid receptor modulator (SBARM)	-10.415	-8.645	-8.523
**251**	Conicasterol E	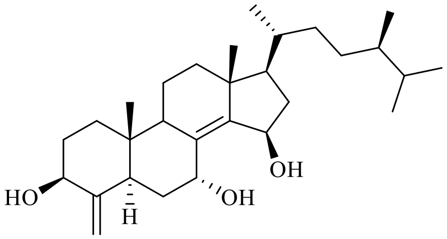	*Theonella swinhoei*	Steroid	A selective bile acid receptor modulator (SBARM)	-8.662	-9.248	-10.047
**252**	3-oxocholest-1,22-dien-12β-ol	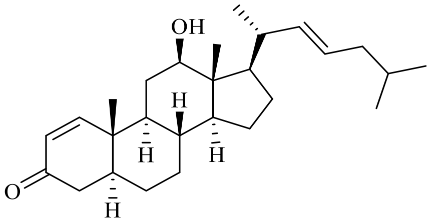	*Dendronephthya gigantea*	Steroid	Antagonist	-9.492	-9.135	-10.323

No.	Name	Natural product	Source	Classification	Activation/ Inhibition	Docking score of 1OSV	Docking score of 1OT7	Docking score of 3BEJ
**253**	3-oxocholest-1,4-dien-20β-ol	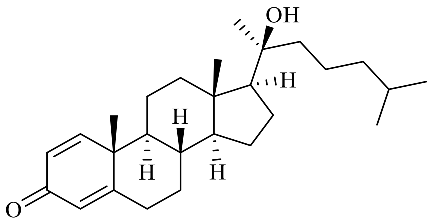	*Dendronephthya gigantea*	Steroid	Antagonist	-8.662	-9.248	-10.047
**254**	Docosahexaenoic acid	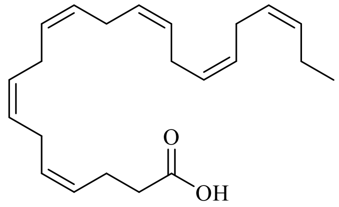	*Crocodylus siamensis*	PUFA, Non-steroid	Antagonist	-	-	-7.963
**255**	Linolenic acid	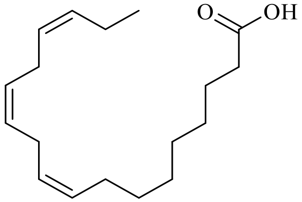	*Perilla frutesccns*	PUFA Non-steroid	Antagonist	-3.428	-3.482	-6.262

No.	Name	Natural product	Source	Classification	Activation/ Inhibition	Docking score of 1OSV	Docking score of 1OT7	Docking score of 3BEJ
**256**	Naringin	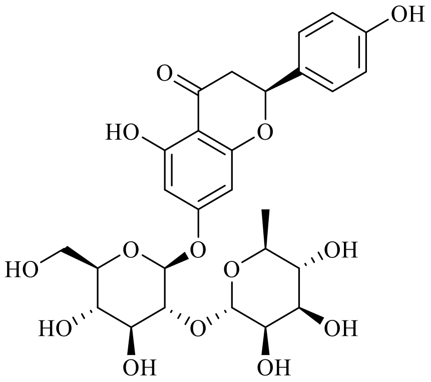	Citrus fruits and grapefruits, particularly in grapefruit peel	Flavonoid	Inhibit FXR	-	-	-
**257**	Maninsigins A	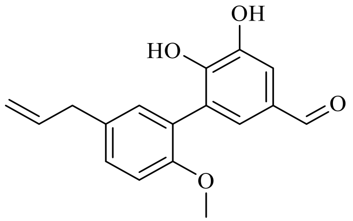	*Magnolia officinalis*	Neolignan Phenylpropanoid	Antagonist	-7.469	-8.062	-7.906
**258**	Palmatine (PAL)	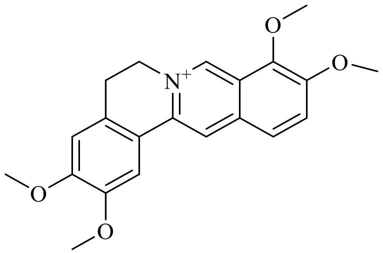	*Rhizoma coptidis*	Alkaloid	Downregulate FXR expression	-8.563	-8.774	-8.092

No.	Name	Natural product	Source	Classification	Activation/ Inhibition	Docking score of 1OSV	Docking score of 1OT7	Docking score of 3BEJ
**259**	Halicylindramides F	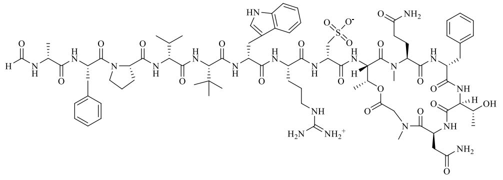	*Petrosia* sp.	Depsipeptide	Antagonist	-	-	-
**260**	Halicylindramides A	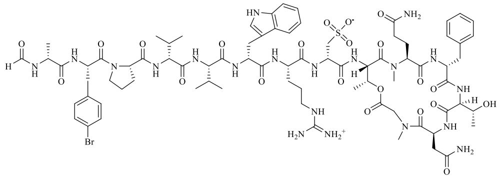	*Petrosia* sp.	Depsipeptide	Antagonist	-	-	-
**261**	Halicylindramides C	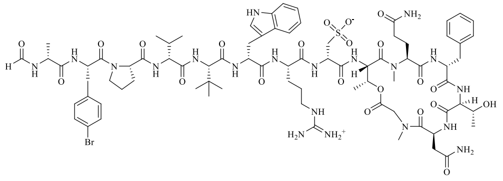	*Petrosia* sp.	Depsipeptide	Antagonist	-	-	-
**22**	Platycodin D	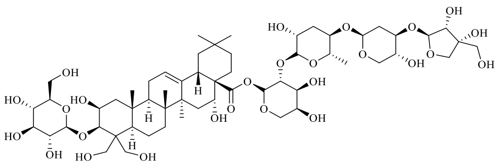	*Platycodon grandiflorum* (Jacq.) A.DC.	Terpene	Activate LXRα and FXR antagonist	-	-	-

No.	Name	Natural product	Source	Classification	Activation/ Inhibition	Docking score of 1OSV	Docking score of 1OT7	Docking score of 3BEJ
**146**	Arachidonic acid	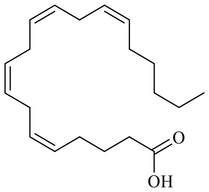	*Acanthopanax koreanum* Nakai.	Non-steroid	LXRα/β dual antagonist and FXR antagonist	-6.415	-6.249	-8.852

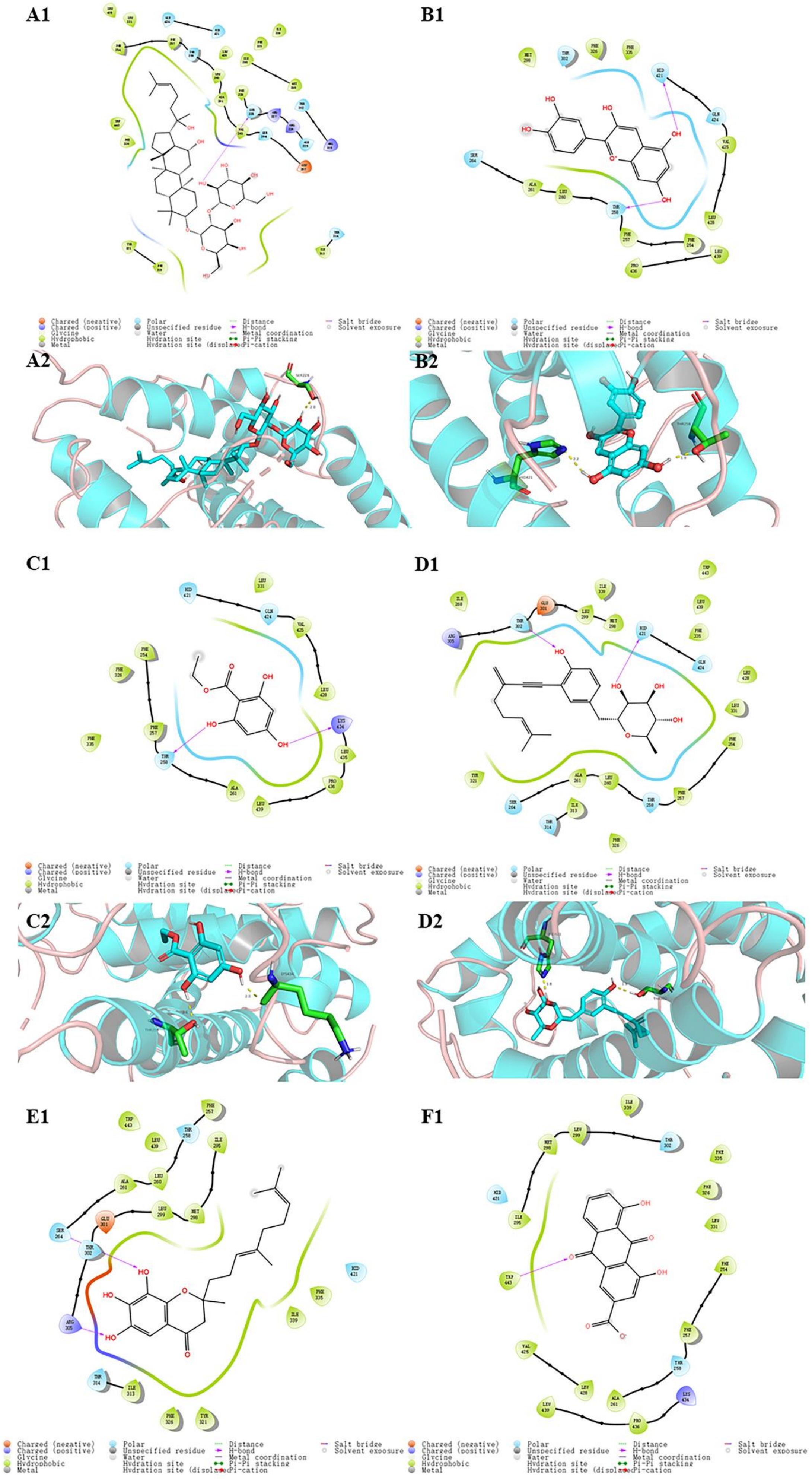
A1
B1
A2
B2
C1
D1
C2
D2
E1
F1
Charged (negative)
Charged (positive)
Glycine
Hydrophobic
Metal
Polar
Unspecified residue
Water
Hydration site
Hydration site (displaced)
Distance
H-bond
Metal coordination
Pi-Pi stacking
Pi-cation
Salt bridge
Solvent exposure

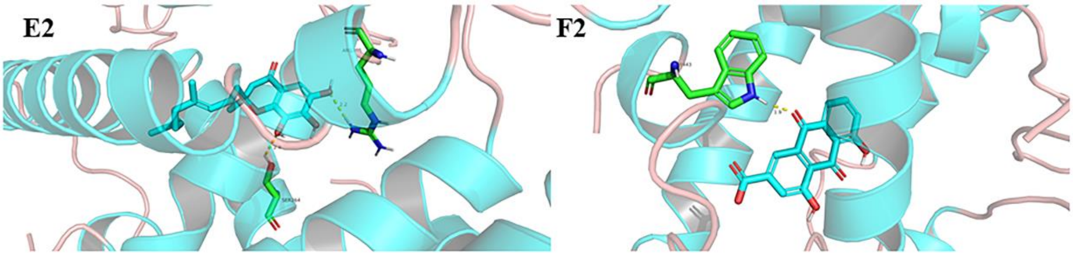

Supplementary Figure 1| 2D and 3D docking modes of **27**, **33**, **81**, **102**, **103**, and **141** with 1UHL (**A**) The 2D (**A1**) and 3D (**A2**) best pose of **27** docking into the LBD with the S value of -8.779 (**B**) The 2D (**B1**) and 3D (**B2**) best pose of **33** docking into the LBD with the S value of -9.187 (**C**) The 2D (**C1**) and 3D (**C2**) best pose of **81** docking into the LBD with the S value of -5.763 (**D**) The 2D (**D1**) and 3D (**D2**) best pose of **102** docking into the LBD with the S value of -8.113 (**E**) The 2D (**E1**) and 3D (**E2**) best pose of **103** docking into the LBD with the S value of -6.766 (**F**) The 2D (**F1**) and 3D (**F2**) best pose of **103** docking into the LBD the S value of -8.502.

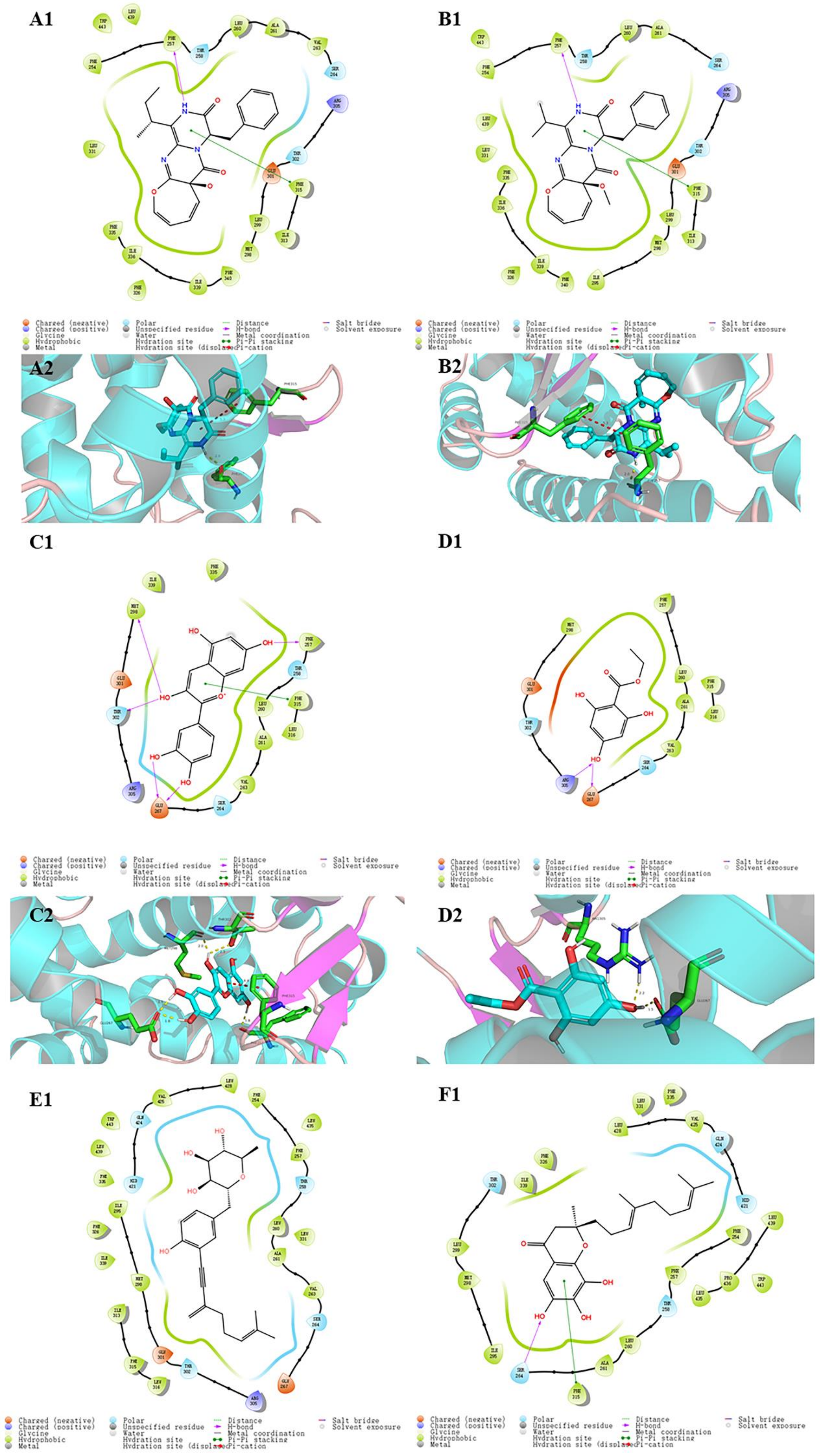
A1
B1
A2
B2
C1
D1
C2
D2
E1
F1
Charged (negative)
Charged (positive)
Glycine
Hydrophobic
Metal
Polar
Unspecified residue
Water
Hydration site
Hydration site (displaced)
Distance
H-bond
Metal coordination
Pi-Pi stacking
Pi-cation
Salt bridge
Solvent exposure

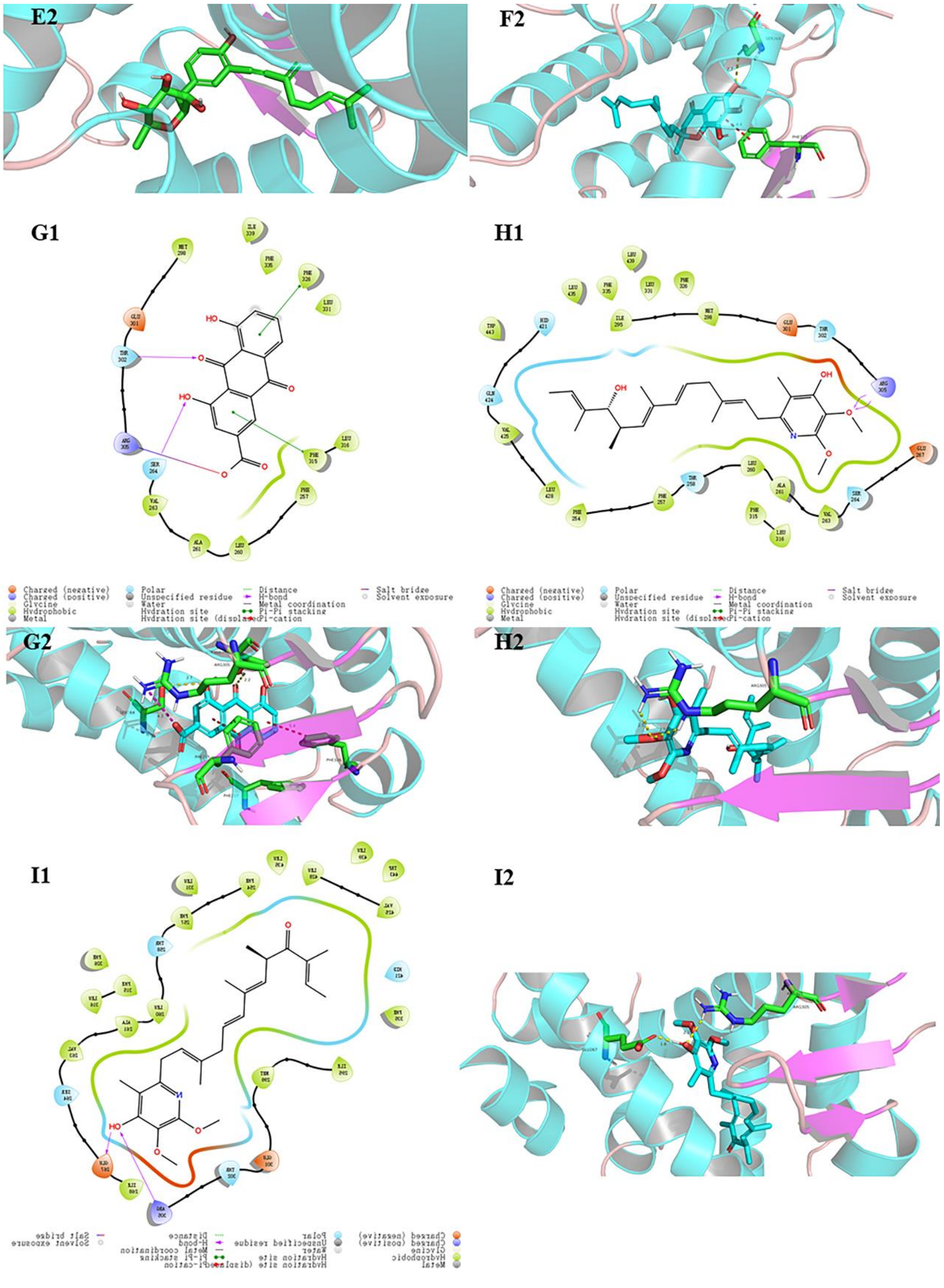

Supplementary Figure 2| 2D and 3D docking modes of **60**, **58**, **33**, **81**, **102**, **103**, **141**, **142**, and **144** with 3IPQ (**A**) The 2D (**A1**) and 3D (**A2**) best pose of **60** docking into the LBD with the S value of -10.776 (**B**) The 2D (**B1**) and 3D (**B2**) best pose of **58** docking into the LBD with the S value of -10.567 (**C**) The 2D (**C1**) and 3D (**C2**) best pose of **33** docking into the LBD with the S value of -8.455 (**D**) The 2D (**D1**) and 3D (**D2**) best pose of **81** docking into the LBD with the S value of -5.380 (**E**) The 2D (**E1**) and 3D (**E2**) best pose of **102** docking into the LBD with the S value of -8.253 (**F**) The 2D (**F1**) and 3D (**F2**) best pose of **103** docking into the LBD with the S value of -8.208 (**G**) The 2D (**G1**) and 3D (**G2**) best pose of **141** docking into the LBD with the S value of -7.900 (**H**) The 2D (**H1**) and 3D (**H2**) best pose of **142** docking into the LBD with the S value

of -9.126. (**I**) The 2D (**I1**) and 3D (**I2**) best pose of **144** docking into the LBD with the S value of -8.261.

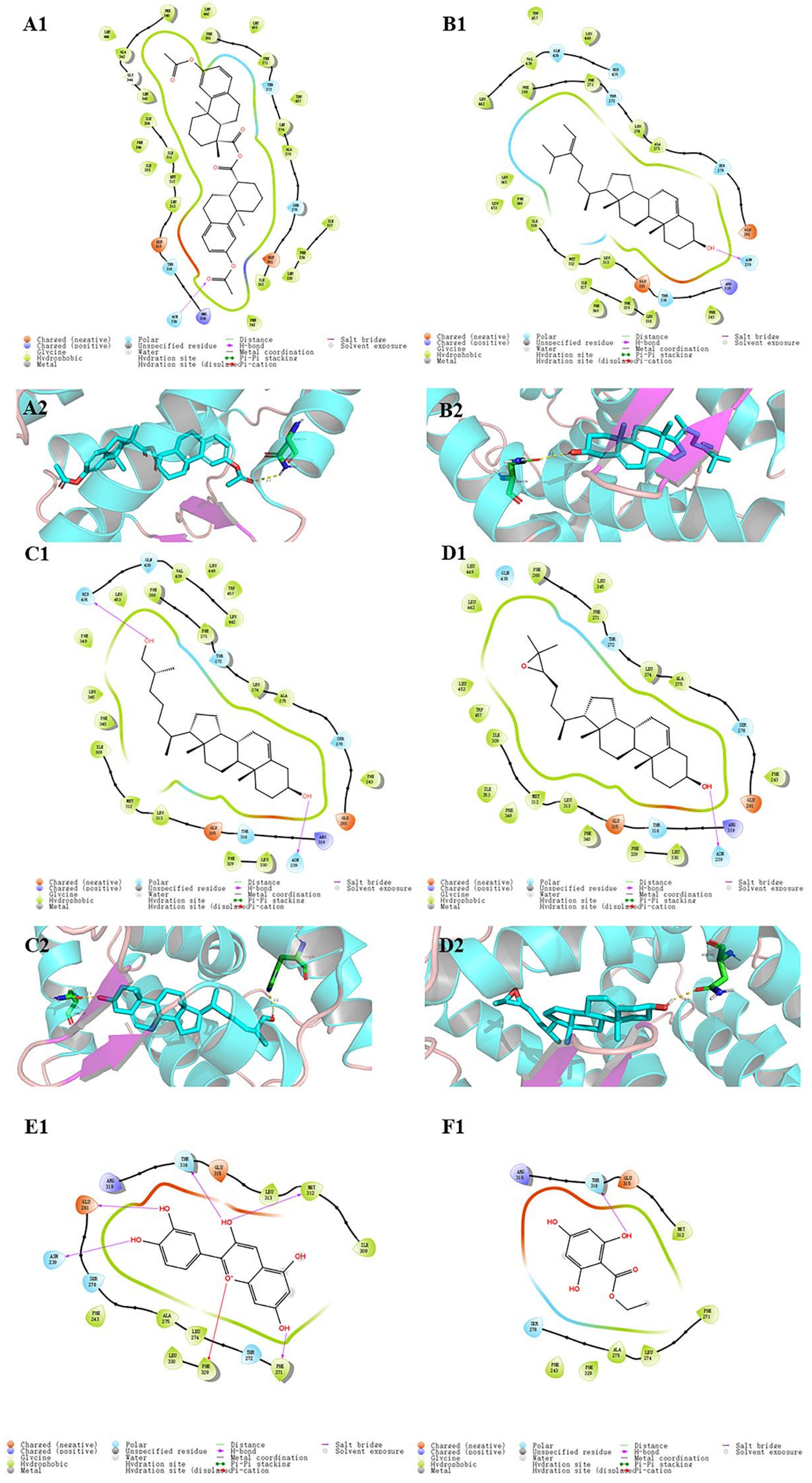
A1
B1
A2
B2
C1
D1
C2
D2
E1
F1
Charged (negative)
Charged (positive)
Glycine
Hydrophobic
Metal
Polar
Unspecified residue
Water
Hydration site
Hydration site (displaced)
Distance
H-bond
Metal coordination
Pi-Pi stacking
Pi-cation
Salt bridge
Solvent exposure

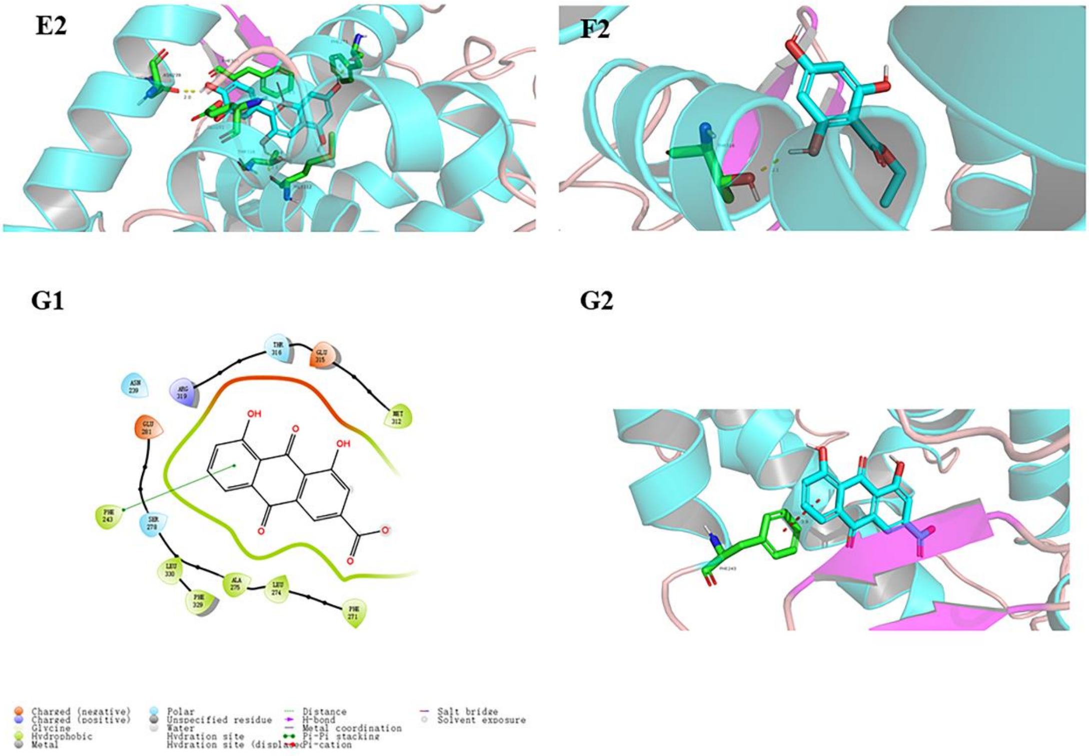

Supplementary Figure 3| 2D and 3D docking modes of **94**, **76**, 27-hydroxycholesterol, 24(S),25-epoxycholesterol, **33**, **81**, and **141** with 1P8D (**A**) The 2D (**A1**) and 3D (**A2**) best pose of **94** docking into the LBD with the S value of -11.457 (**B**) The 2D (**B1**) and 3D (**B2**) best pose of **76** docking into the LBD with the S value of -10.056 (**C**) The 2D (**C1**) and 3D (**C2**) best pose of 27-hydroxycholesterol docking into the LBD with the S value of -9.982 (**D**) The 2D (**D1**) and 3D (**D2**) best pose of 24(S),25-epoxycholesterol docking into the LBD with the S value of -9.941 (**E**) The 2D (**E1**) and 3D (**E2**) best pose of **33** docking into the LBD with the S value of -8.715 (**F**) The 2D (**F1**) and 3D (**F2**) best pose of **81** docking into the LBD with the S value of -5.578 (**G**) The 2D (**G1**) and 3D (**G2**) best pose of **141** docking into the LBD with the S value of -7.227.

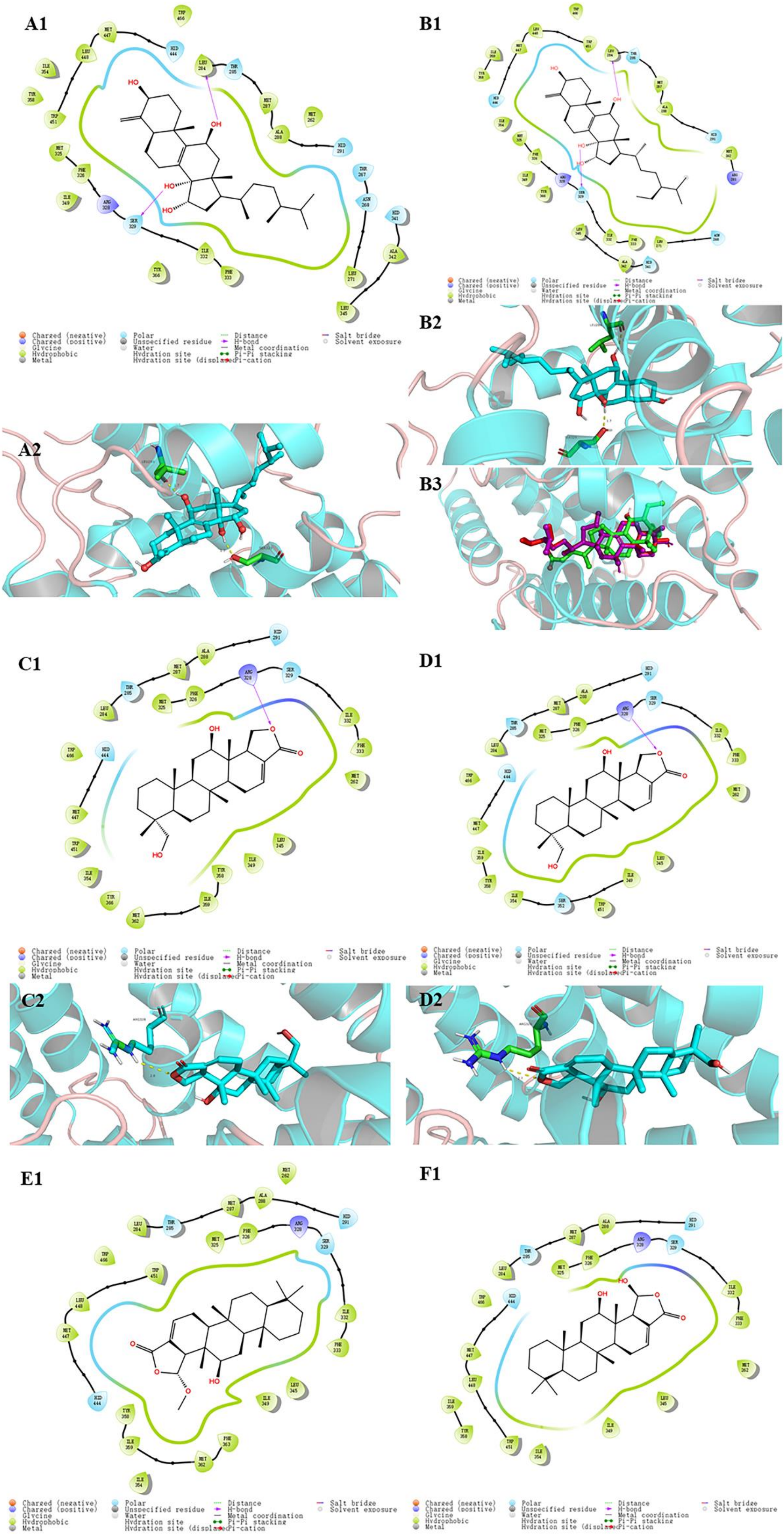
A1
B1
Charged (negative)
Charged (positive)
Glycine
Hydrophobic
Metal
Polar
Unspecified residue
Water
Hydration site
Hydration site (displaced)
Distance
H-bond
Metal coordination
Pi-Pi stacking
Pi-cation
Salt bridge
Solvent exposure
B2
A2
B3
C1
D1
C2
D2
E1
F1

E2

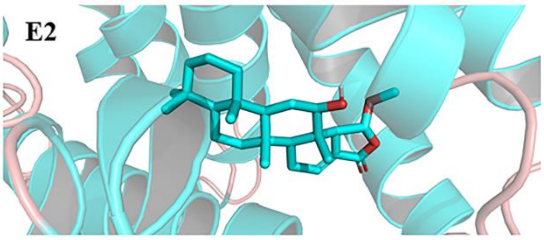

F2

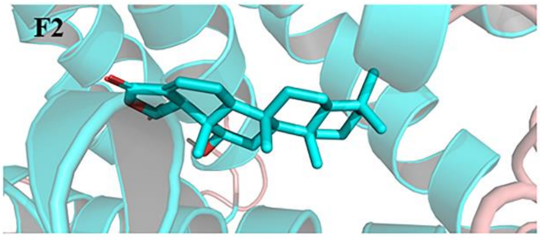

G1

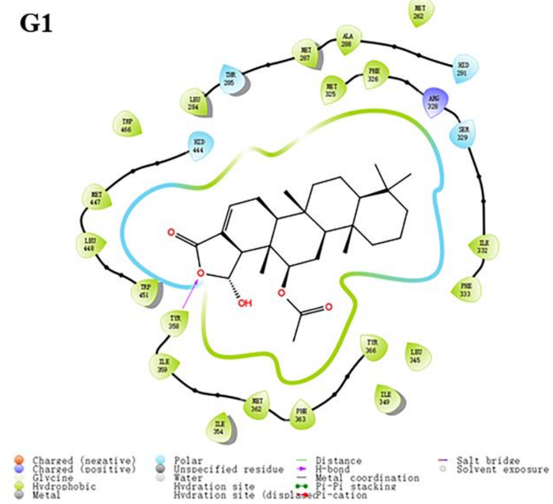

H1

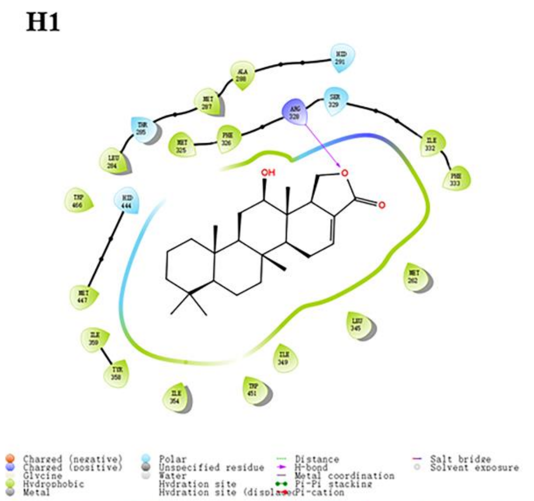

G2

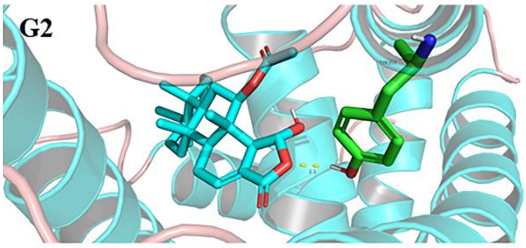

H2

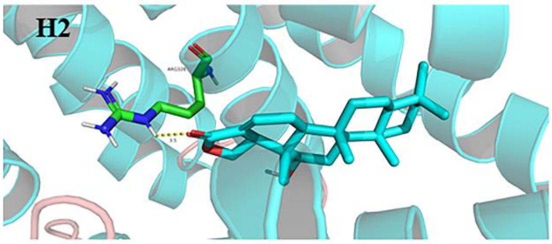

I1

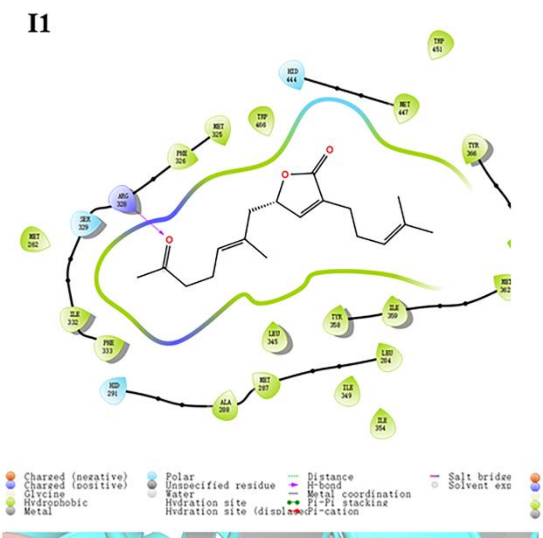

J1

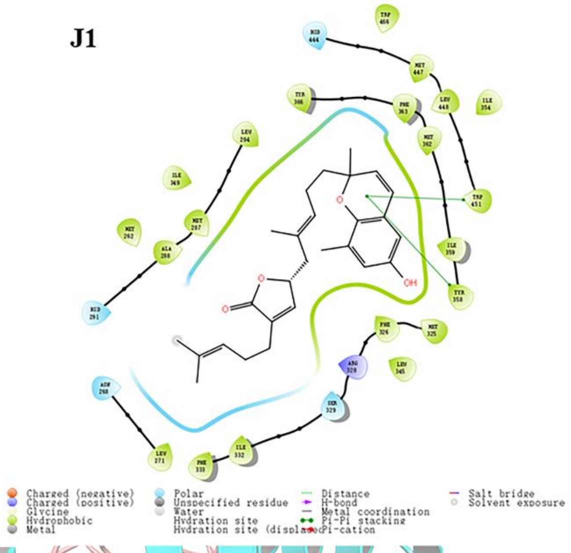

I2

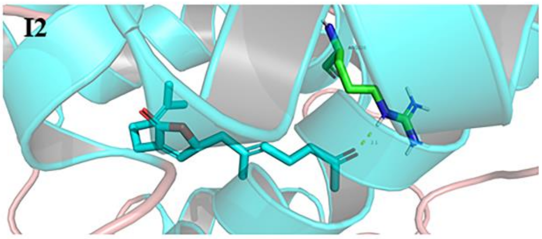

J2

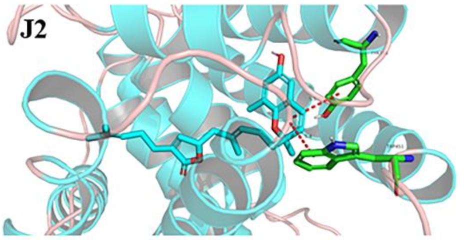

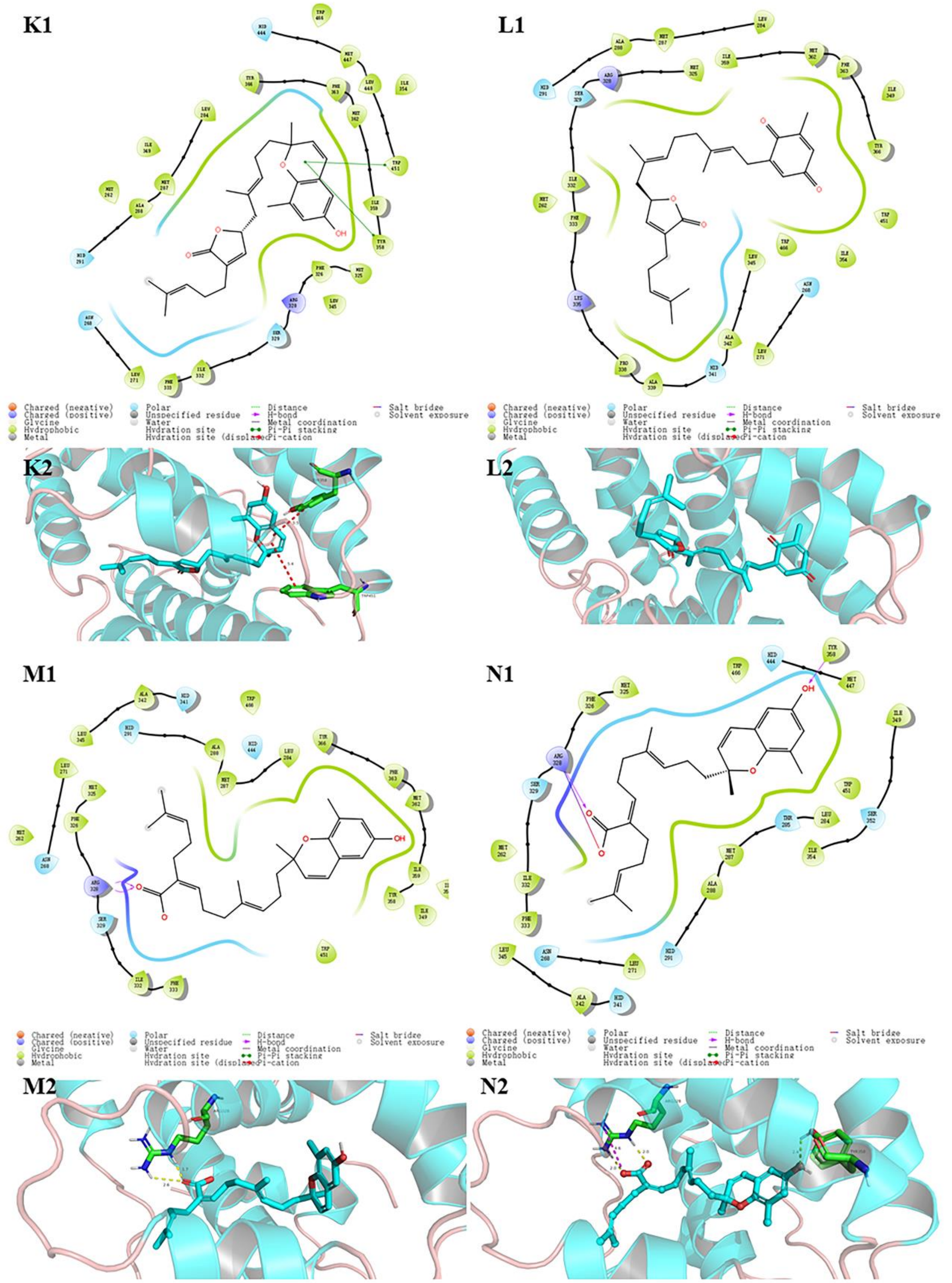

Supplementary Figure 4| 2D and 3D docking modes of **239**, **250**, and **222-233** with 1OSV (**A**) The 2D (**A1**) and 3D (**A2**) best pose of **239** docking into the LBD with the S value of -10.917 (**B**) The 2D (**B1**) and 3D (**B2**) best pose of **250** docking into the LBD with the S value of -10.415 (**B3**) Predicted modes of **233** (in purple), **250** (in red) and obeticholic acid (in green) with 1OSV from molecular docking (**C**) The 2D (**C1**) and 3D (**C2**) best pose of **222** docking into the LBD with the S value of -8.819 (**D**) The 2D (**D1**) and 3D (**D2**) best pose of **223** docking into the LBD with the S value of -8.941 (**E**) The 2D (**E1**) and 3D (**E2**) best pose of **224** docking into the LBD with the S value of -8.032 (**F**) The 2D (**F1**) and 3D (**F2**) best pose of **225** docking into the LBD with the S value of -9.260 (**G**) The 2D (**G1**) and 3D (**G2**) best pose of **226** docking into the LBD

with the S value of -9.471 (**H**) The 2D (**H1**) and 3D (**H2**) best pose of **227** docking into the LBD with the S value of -9.275 (**I**) The 2D (**I1**) and 3D (**I2**) best pose of **228** docking into the LBD with the S value of -5.849 (**J**) The 2D (**J1**) and 3D (**J2**) best pose of **229** docking into the LBD with the S value of -8.66 (**K**) The 2D (**K1**) and 3D (**K2**) best pose of **230** docking into the LBD with the S value of -8.66 (**L**) The 2D (**L1**) and 3D (**L2**) best pose of **231** docking into the LBD with the S value of -8.416 (**M**) The 2D (**M1**) and 3D (**M2**) best pose of **232** docking into the LBD with the S value of -8.616 (**N**) The 2D (**N1**) and 3D (**N2**) best pose of **233** docking into the LBD with the S value of -9.179.

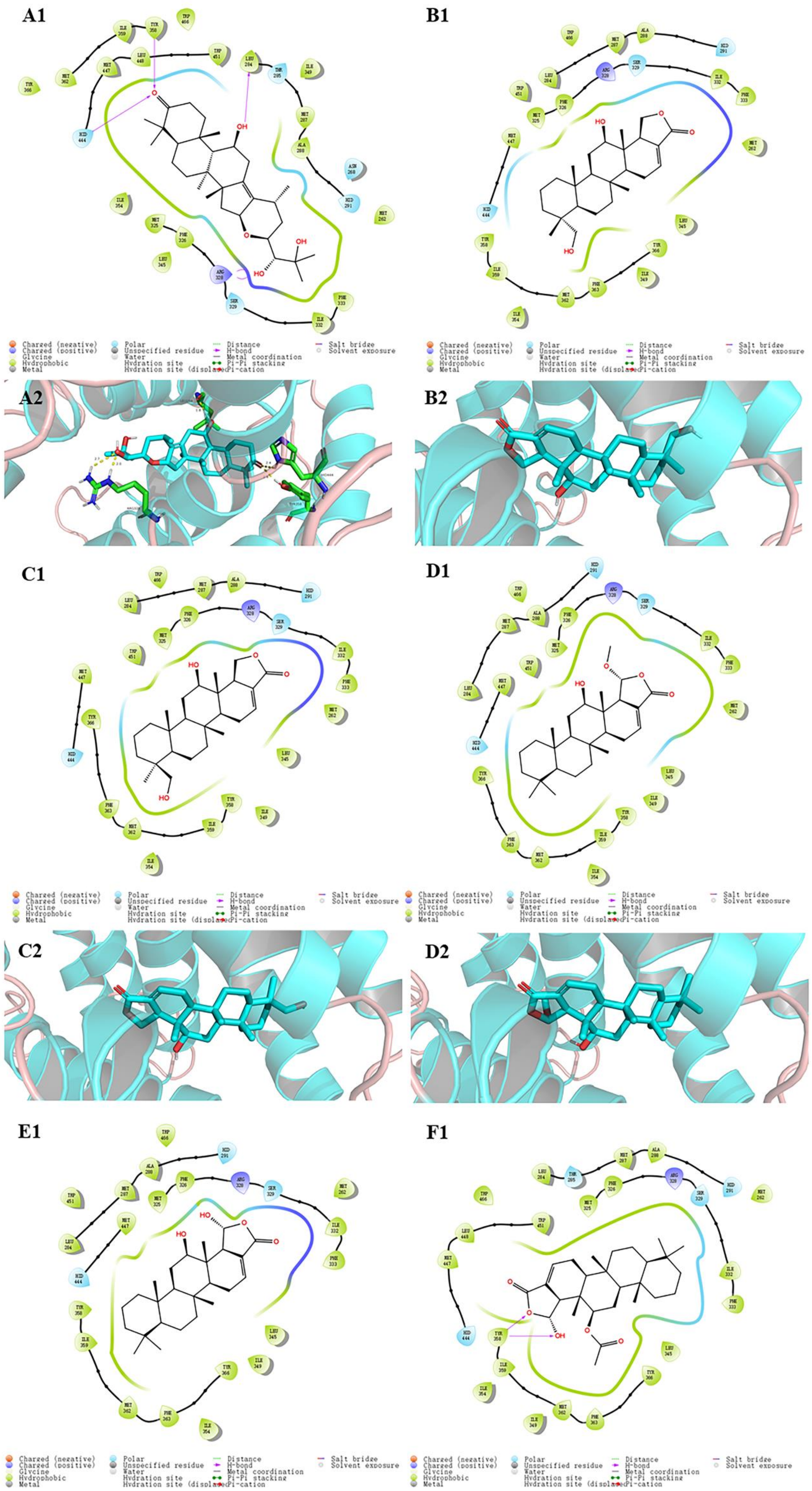
A1
B1
A2
B2
C1
D1
C2
D2
E1
F1
Charged (negative)
Charged (positive)
Glycine
Hydrophobic
Metal
Polar
Unspecified residue
Water
Hydration site
Hydration site (displaced)
Distance
H-bond
Metal coordination
Pi-Pi stacking
Pi-cation
Salt bridge
Solvent exposure

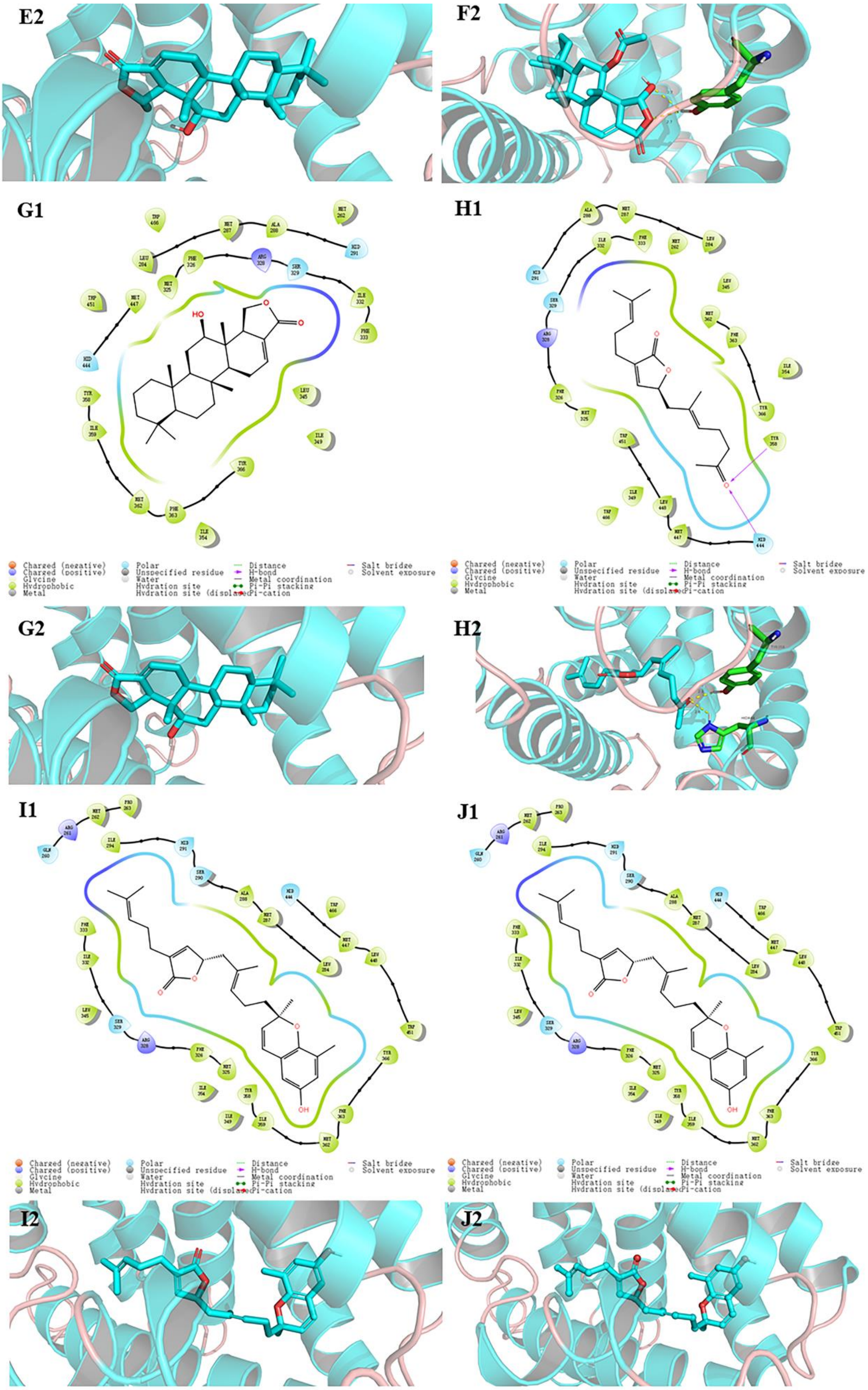
E2
F2
G1
H1
Charged (negative)
Charged (positive)
Glycine
Hydrophobic
Metal
Polar
Unspecified residue
Water
Hydration site
Hydration site (displaced)
Distance
H-bond
Metal coordination
Pi-Pi stacking
Pi-cation
Salt bridge
Solvent exposure
G2
H2
I1
J1
I2
J2

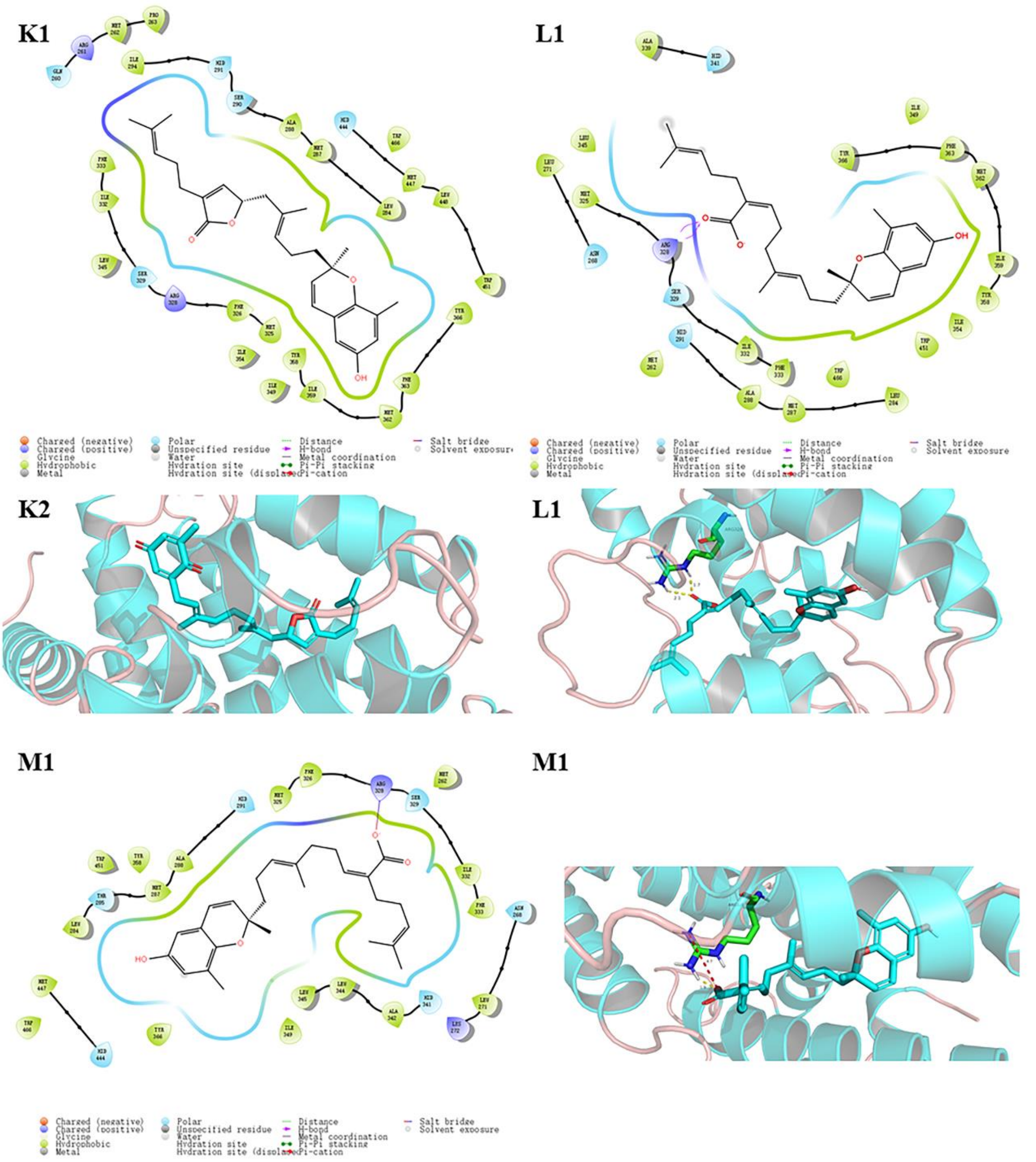

Supplementary Figure 5| 2D and 3D docking modes of **168** and **222-233** with 1OT7 (**A**) The 2D (**A1**) and 3D (**A2**) best pose of **168** docking into the LBD with the S value of -10.937 (**B**) The 2D (**B1**) and 3D (**B2**) best pose of **222** docking into the LBD with the S value of -8.561 (**C**) The 2D (**C1**) and 3D (**C2**) best pose of **223** docking into the LBD with the S value of -8.672 (**D**) The 2D (**D1**) and 3D (**D2**) best pose of **224** docking into the LBD with the S value of -8.614 (**E**) The 2D (**E1**) and 3D (**E2**) best pose of **225** docking into the LBD with the S value of -9.352 (**F**) The 2D (**F1**) and 3D (**F2**) best pose of **226** docking into the LBD with the S value of -8.771 (**G**) The 2D (**G1**) and 3D (**G2**) best pose of **227** docking into the LBD with the S value of -9.152 (**H**) The 2D (**H1**) and 3D (**H2**) best pose of **228** docking into the LBD with the S value of -5.899 (**I**) The 2D (**I1**) and 3D (**I2**) best pose of **229** docking into the LBD with the S value of -8.759 (**J**) The 2D (**J1**) and 3D (**J2**) best pose of **230** docking into the LBD with the S value of -8.759 (**K**) The 2D (**K1**) and 3D (**K2**) best pose of **231** docking into the LBD with the S value of -7.906 (**L**) The 2D (**L1**) and 3D (**L2**) best pose of **232** docking into the LBD

with the S value of -8.812 (**M**) The 2D (**M1**) and 3D (**M2**) best pose of **233** docking into the LBD with the S value of -8.801.

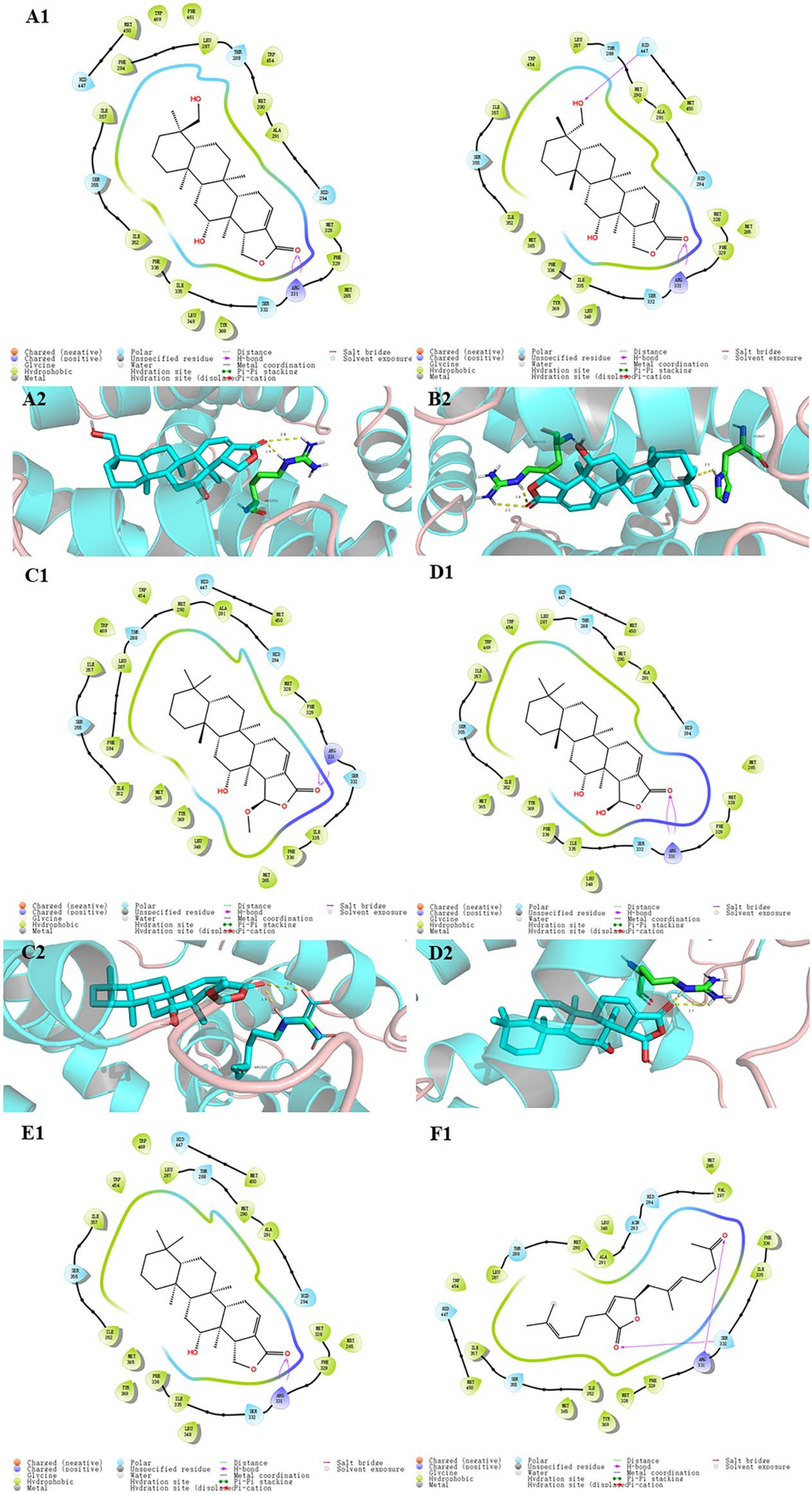
A1
A2
B2
C1
D1
C2
D2
E1
F1
Charged (negative)
Charged (positive)
Glycine
Hydrophobic
Metal
Polar
Unspecified residue
Water
Hydration site
Hydration site (displaced)
Distance
H-bond
Metal coordination
Pi-Pi stacking
Pi-cation
Salt bridge
Solvent exposure

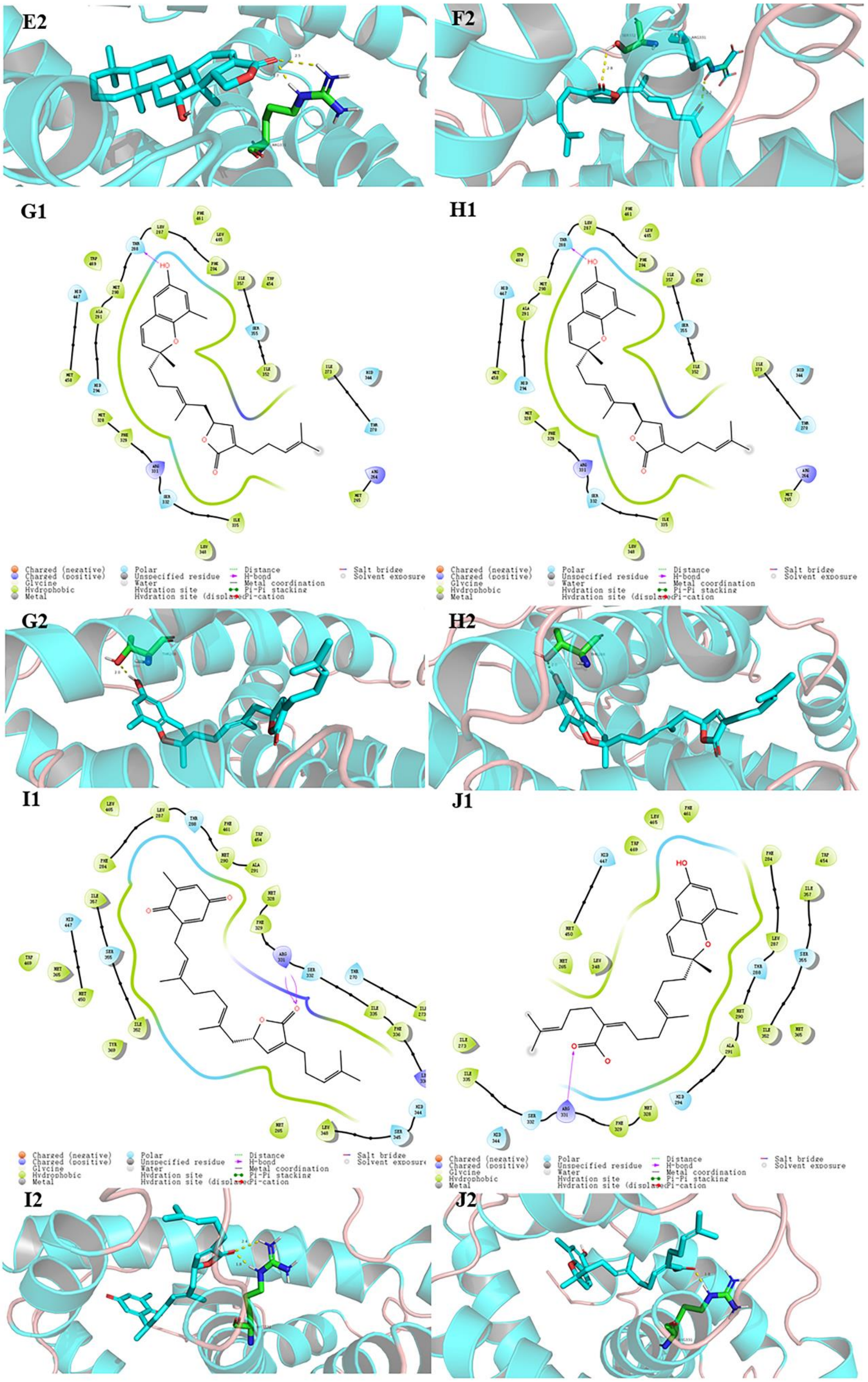
E2
F2
G1
H1
Charged (negative)
Charged (positive)
Glycine
Hydrophobic
Metal
Polar
Unspecified residue
Water
Hydration site
Hydration site (displaced)
Distance
H-bond
Metal coordination
Pi-Pi stacking
Pi-cation
Salt bridge
Solvent exposure
G2
H2
I1
J1
I2
J2

K1

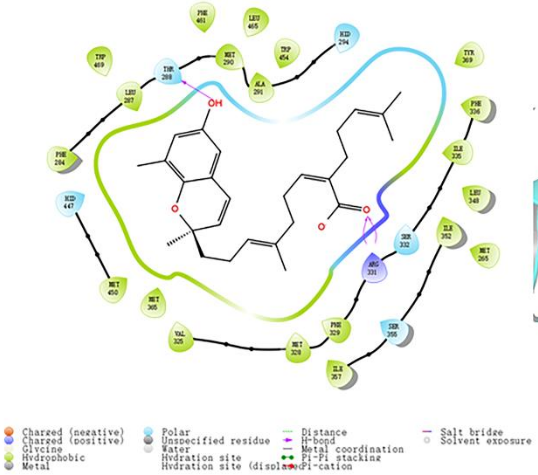

K2

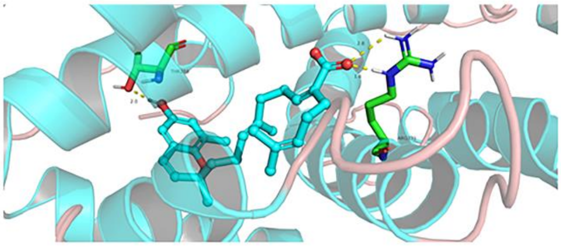

Supplementary Figure 6| 2D and 3D docking modes of **222-225** and **227-233** with 3BEJ (**A**) The 2D (**A1**) and 3D (**A2**) best pose of **222** docking into the LBD with the S value of -9.642 (**B**) The 2D (**B1**) and 3D (**B2**) best pose of **223** docking into the LBD with the S value of -9.588 (**C**) The 2D (**C1**) and 3D (**C2**) best pose of **224** docking into the LBD with the S value of -9.381 (**D**) The 2D (**D1**) and 3D (**D2**) best pose of **225** docking into the LBD with the S value of -9.677 (**E**) The 2D (**E1**) and 3D (**E2**) best pose of **227** docking into the LBD with the S value of -10.055 (**F**) The 2D (**F1**) and 3D (**F2**) best pose of **228** docking into the LBD with the S value of -5.861 (**G**) The 2D (**G1**) and 3D (**G2**) best pose of **229** docking into the LBD with the S value of -10.113 (**H**) The 2D (**H1**) and 3D (**H2**) best pose of **230** docking into the LBD with the S value of -10.113 (**I**) The 2D (**I1**) and 3D (**I2**) best pose of **231** docking into the LBD with the S value of -8.894 (**J**) The 2D (**J1**) and 3D (**J2**) best pose of **232** docking into the LBD with the S value of -9.357 (**K**) The 2D (**K1**) and 3D (**K2**) best pose of **233** docking into the LBD with the S value of -11.025.
